# Prevalence of *Trypanosoma* and *Sodalis* in wild populations of tsetse flies and their impact on sterile insect technique programmes for tsetse eradication

**DOI:** 10.1038/s41598-022-06699-2

**Published:** 2022-02-28

**Authors:** Mouhamadou M. Dieng, Kiswend-sida M. Dera, Percy Moyaba, Gisele M. S. Ouedraogo, Guler Demirbas-Uzel, Fabian Gstöttenmayer, Fernando C. Mulandane, Luis Neves, Sihle Mdluli, Jean-Baptiste Rayaisse, Adrien M. G. Belem, Soumaïla Pagabeleguem, Chantel J. de Beer, Andrew G. Parker, Jan Van Den Abbeele, Robert L. Mach, Marc J. B. Vreysen, Adly M. M. Abd-Alla

**Affiliations:** 1 Insect Pest Control Laboratory, Joint FAO/IAEA Centre of Nuclear Techniques in Food and Agriculture, 1400 Vienna, Austria; 2 Insectarium de Bobo Dioulasso-Campagne d’Eradication de la mouche tsetse et de la Trypanosomose (IBD-CETT), 01 BP 1087, Bobo Dioulasso 01, Burkina Faso; 3 Epidemiology, Vectors and Parasites, Agricultural Research Council-Onderstepoort Veterinary Research, Pretoria, South Africa; 4 University Eduardo Mondlane, Centro de Biotecnologia, Av. de Moçambique Km 1.5, Maputo, Mozambique; 5 Department of Veterinary Tropical Diseases, University of Pretoria, Private Bag X04, Onderstepoort, 0110 South Africa; 6 Epidemiology Unit, Department of Veterinary Services, PO Box 4192, Manzini, Eswatini; 7 Centre International de Recherche-Développement sur l’Elevage en zone Subhumide (CIRDES), 01 BP 454, Bobo-Dioulasso 01, Burkina Faso; 8 Universite´ Nazi Boni (UNB), Bobo-Dioulasso, Burkina Faso; 9 University of Dedougou, B.P. 176, Dédougou 01, Burkina Faso; 10 Roppersbergweg 15, 2381 Laab im Walde, Austria; 11 Institute of Tropical Medicine Antwerp (ITM), Antwerp, Belgium; 12 Institute of Chemical, Environmental, and Bioscience Engineering, Vienna University of Technology, Gumpendorfer Straße 1a, 1060 Vienna, Austria

**Keywords:** Biotechnology, Microbiology, Molecular biology, Systems biology

## Abstract

The sterile insect technique (SIT) is an environment friendly and sustainable method to manage insect pests of economic importance through successive releases of sterile irradiated males of the targeted species to a defined area. A mating of a sterile male with a virgin wild female will result in no offspring, and ultimately lead to the suppression or eradication of the targeted population. Tsetse flies, vectors of African *Trypanosoma,* have a highly regulated and defined microbial fauna composed of three bacterial symbionts that may have a role to play in the establishment of *Trypanosoma* infections in the flies and hence, may influence the vectorial competence of the released sterile males. *Sodalis* bacteria seem to interact with *Trypanosoma* infection in tsetse flies. Field-caught tsetse flies of ten different taxa and from 15 countries were screened using PCR to detect the presence of *Sodalis* and *Trypanosoma* species and analyse their interaction. The results indicate that the prevalence of *Sodalis* and *Trypanosoma* varied with country and tsetse species. Trypanosome prevalence was higher in east, central and southern African countries than in west African countries. Tsetse fly infection rates with *Trypanosoma vivax* and *T. brucei* sspp were higher in west African countries, whereas tsetse infection with *T. congolense* and *T. simiae, T. simiae* (*tsavo*) and *T. godfreyi* were higher in east, central and south African countries. *Sodalis* prevalence was high in *Glossina morsitans morsitans* and *G. pallidipes* but absent in *G. tachinoides.* Double and triple infections with *Trypanosoma* taxa and coinfection of *Sodalis* and *Trypanosoma* were rarely observed but it occurs in some taxa and locations. A significant Chi square value (< 0.05) seems to suggest that *Sodalis* and *Trypanosoma* infection correlate in *G. palpalis gambiensis*, *G. pallidipes* and *G. medicorum*. *Trypanosoma* infection seemed significantly associated with an increased density of *Sodalis* in wild *G. m. morsitans* and *G. pallidipes* flies, however, there was no significant impact of *Sodalis* infection on trypanosome density.

## Introduction

Tsetse flies (Diptera: Glossinidae) are distributed in sub-Saharan Africa between 15° north and 26° south latitude^1^. *Glossina* spp*.* are the cyclic vectors^2^ of unicellular protozoa of the genus *Trypanosoma* that cause African animal trypanosomosis (AAT) or nagana and human African trypanosomosis (HAT) or sleeping sickness^3,4^. Nagana in cattle is mainly caused by *T. congolense*, *T. vivax* and *T. brucei brucei*^5^ and causes annual losses to agriculture estimated at $4.75 billion^6^. In addition, around 35 million doses of trypanocidal drugs are administered to livestock per year for managing AAT^7^. Human African trypanosomosis is fatal without treatment^8^ and is caused by two *Trypanosoma* subspecies, i.e. *T. brucei rhodesiense* responsible for the acute form of HAT in East Africa and *T. b. gambiense* for the chronic form of HAT in western and central Africa^9^. The lack of effective vaccines and the development of resistance to the available trypanocidal drugs makes the control of AAT in the vertebrate host unsustainable^10,11^. Consequently, an effective tool to reduce *Trypanosoma* transmission would be the control of the tsetse vector. One effective method to manage populations of tsetse flies is the sterile insect technique (SIT) when used as part of an area-wide integrated pest management (AW-IPM) approach^12,13^. The SIT method relies on the mass-production and sterilization of male flies by ionizing radiation. The sterile males are released in the target area for mating with wild females and the absence of offspring will gradually reduce the density of the targeted tsetse populations^14^.

The biological transmission of the *Trypanosoma* species requires the parasite to undergo a series of proliferation and differentiation steps in the tsetse alimentary tract and finally mature into an infective form in the mouthparts (*T. congolense*) or salivary glands (*T. brucei* spp.)^15^. However, tsetse flies are refractory to *Trypanosoma* infection meaning that the probability that *Trypanosoma* ingested during a blood meal complete their developmental cycle in the fly to result in a mature infection is rather low^16–18^. The endogenous bacterial microbiome seems important in providing tsetse flies the natural ability to mitigate *Trypanosoma* infections^19^. Three major endosymbiotic bacteria have been identified in tsetse flies, i.e. *Wigglesworthia glossinidia*, *Sodalis glossinidius* (hereafter mentioned as *Sodalis*) and *Wolbachia pipientis*^20^. Some studies suggested that the obligate mutualist *Wigglesworthia* must be present in the larval stage during the development of a mature tsetse fly to properly develop a well-functioning immune system contributing to a refractory phenotype against *Trypanosoma*^5,19^.

*Sodalis,* the second mutualistic symbiont, can be found in the midgut, hemolymph, muscles, fat body, milk glands, and salivary glands of certain tsetse species and is inherited by the progeny through transovarial transmission^21^. The biological role/importance of *Sodalis* for tsetse remain unclear and needs to be clarified^22^. This symbiont might provide some benefits to the host as flies without *Sodalis* have a significantly shorter lifespan as compared with flies with it^23^, however the establishment of a *Sodalis* free colony was feasible^24^. *Sodalis* also presents many ideal characteristics to be used for expressing molecular effectors in paratransgenic tsetse^25^. In addition, previous work suggested that *Sodalis* may modulate the ability of *Trypanosoma* to establish an infection in the tsetse midgut as some studies reported that the elimination of this bacterial endosymbiont results in an increased tsetse fly refractoriness to *Trypanosoma* infection^23,26,27^. Moreover, Geiger et al.,^28^ suggested that specific genotypes of *Sodalis* presents in *G. p. gambiensis* from insectary colonies facilitate *Trypanosoma* infection. Soumana et al.,^29^ revealed that a variation in the *Sodalis* population caused by a hosted prophage can influence the trypanosome infections. In contrast, a recent study demonstrated that the absence or presence of *S. glossinidius* in the tsetse fly does not affect the fly’s susceptibility toward *Trypanosoma* infection^24^. In conclusion, from the above-described results, it is clear that our knowledge on the impact of *Sodalis* on *Trypanosoma* infection in tsetse remains limited and fragmented and is still under debate^23^. Moreover, exploring on a large scale the occurrence and possible association between *Sodalis* and *Trypanosoma* infection in wild flies is highly required. The above described potential impact of *Sodalis* to facilitate *Trypanosoma* infection in tsetse, and the fact that *Sodalis* is found in all laboratory-reared tsetse colonies and some wild populations^21^ indicates that mitigating action, such as feeding the flies 2–3 times on blood supplemented with trypanocidal drugs before release, is required in SIT programs to minimize the risk of disease transmission by the large number of released males that harbour *Sodalis*.

Field studies in two HAT foci in Cameroon used PCR to detect *Trypanosoma* and *Sodalis* in *G. palpalis palpalis* and the results indicate that the presence of *Sodalis* favours *Trypanosoma* infections especially by *T. brucei* s.l.^30^. Furthermore, in the wildlife-livestock-human interface in the Maasai Mara National Reserve in Kenya, it was shown that *G. pallidipes* infected with *Sodalis* was associated with increased *Trypanosoma* infection rates^31^. However, other studies have found no strong association between trypanosome and *Sodalis* in some tsetse species collected in four locations in Kenya^32^. Channumsin et al.,^33^ suggested that the association between *Trypanosoma* infection and the presence of *Sodalis* will vary depending on tsetse and *Trypanosoma* species. Similarly, studies carried out in the Fontem focus in Cameroon did not find a relationship between the endosymbiont and the parasite in *G. p. palpalis*^34^, and no significant *Sodalis*-*Trypanosoma* infection association was found in *G. tachinoides* in two sites of the Faro and Déo Division in Adamawa region of Cameroon^35^. Likewise, no association between the presence of the parasite and *Sodalis* was found in *G. brevipalpis*, *G. m. morsitans* and *G. pallidipes* in the Luambe National Park of Zambia^36^.

The overall objective of this study was to evaluate the prevalence of *Sodalis* and *Trypanosoma* in wild tsetse populations at a continental scale, i.e. Burkina Faso, Democratic Republic of Congo (DRC), Eswatini, Ethiopia, Ghana, Guinea, Kenya, Mali, Mozambique, Senegal, South Africa, Tanzania, Uganda, Zambia, and Zimbabwe and analyse these data in the context of a possible association between the occurrence of *Sodalis* and *Trypanosoma* infection in tsetse. Such information might guide the decision maker for SIT programmes to take the appropriate action, if necessary, to minimize any potential risk of increased transmission.

## Results

### *Trypanosoma* prevalence

Adult tsetse flies (n = 6860) were screened for infection with *T. brucei* sspp (Tz) (T*. b. brucei, T. b. gambiense, T. b. rhodesiense)*, Tc (*T. congolense* savannah; *T. congolense* kilifi; *T. congolense* forest); Tsg (*T. simiae*; *T. simiae* tsavo; *T. godfreyi*) and Tv (*T. vivax*). The results indicate that 1736 (25.3%) adults were infected with one or more *Trypanosoma* taxa (Tables 1, 2 and 3), The *Trypanosoma* prevalence varied significantly between tsetse taxa (*X*^2^ = 750.18, *df* = 9, *P* <<  0.001) and between countries (*X*^2^ = 2038.1, *df* = 14, *P*  <<  0.001). The Permanova analysis indicated as well significant differences between countries (*P* = 0.009) and taxa (*P* = 0.041) (Table 4). As all taxa were not collected from all countries, the interaction between taxa and countries was only analyzed where a taxon was collected from several countries.

**Table 1:** Global prevalence of *Sodalis* and Trypanosomes in tsetse samples analyzed per country.

Region	Country	*Sodalis* prevalence (%)*	Trypanosome prevalence (%)
	Ethiopia	94/459 (20.48)^a,b,e^	92/459 (20.04)^a,d,e^
East, central and southern Africa	Kenya	288/1008 (28.57)^a,b^	448/1008 (44.44)^a,b,e^
	Democratic R. of Congo	4/35 (11.43)^a,b,e^	1/35 (2.86)^a,e^
	Mozambique	7/100 (7.00)^a,b,e^	80/526 (15.21)^a,e^
	South Africa	9/526 (1.71)^a,c,e^	0/30 (0.00)^a,e^
	Eswatini	0/30 (0.00)^a,b,c,e^	8/100 (8.00)^a,e^
	Tanzania	227/338 (67.16)^a,d,c^	128/338 (37.87)^a,e^
	Uganda	91/210 (43.33)^d^	19/210 (9.05)^a,c,e^
	Zambia	11/210 (5.24)^a,b,e^	97/210 (46.19)^a,d,e^
	Zimbabwe	39/211(18.48)^a,b,e^	113/211 (53.55)^a,e^
	Subtotal	770/3127 (24.62)	986/3127 (31.53)
West Africa	Burkina Faso	11/2274 (0.48)^a,e^	498/2274 (21.90^)a,e^
	Ghana	0/234 (0.00)^a,e^	143/234 (61.11)^a,d^
	Guinea	90/314 (28.66)^a,e^	7/314 (2.22)^a,c^
	Mali	0/364 (0.00)^a,e^	25/364 (6.86)^a,c,e^
	Senegal	0/547 (0.00)^a,e^	78/547 (14.25)^a,e^
	Subtotal	101/3733 (2.70)	750/3733 (20.09)
	Total (average)	871/6860 (12.69)	1736/6860 (25.30)

*Values indicated by the same lower-case letter do not differ significantly at the 5% level.

**Table 2:** Global prevalence of *Sodalis* and Trypanosomes in tsetse samples analyzed per tsetse species.

Species	*Sodalis* prevalence (%)*	Trypanosome prevalence (%)
*G. austeni*	5/346 (1.44)^a^	58/346 (16.76)^a^
*G. brevipalpis*	14/350 (4)^a^	34/350 (9.71)^a^
*G. f. fuscipes*	24/183 (13.11)^a,b^	31/183 (16.93)^a^
*G. medicorum*	8/154 (5.2)^a^	61/154 (39.6)^a,b^
*G. m. morsitans*	156/369 (42.27)^b^	152/369 (41.19)^a^
*G. m. submorsitans*	1/343 (0.29)^a^	62/343 (18.07)^a^
*G. pallidipes*	567/1844 (30.74)^b^	711/1844 (38.55)^a,b^
*G. p. gambiensis*	92/2168 (4.24)^a^	343/2168 (15.82)^a^
*G. p. palpalis*	4/35 (11.4)^a,b^	1/ 35 (2.8)^a,b^
*G. tachinoides*	0/1068 (0.0)^a^	283/1068 (26.49)^b^
Total (average)	871/6860 (12.6)	1736/6860 (25.3)

*Values indicated by the same lower-case letter do not differ significantly at the 5% level.

**Table 3:** Global prevalence of *Sodalis* and trypanosomes in tsetse samples analyzed per country and tsetse species.

Species	Country	*Sodalis* prevalence (%)*	Trypanosome prevalence (%)
*G. austeni*	Mozambique	0/50 (0.00)	5/50 (10.00)
	South Africa	2/226 (0.88)	49/226 (21.68)
	Eswatini	0/30 (0.00)	0/30 (0.00)
	Tanzania	3/40 (7.50)	4/40 (10.00)
*G. brevipalpis*	Mozambique	7/50 (14.00)^a^	3/50 (6.00)
	South Africa	7/300 (2.33)^b^	31/300 (10.33)
*G. f. fuscipes*	Kenya	20/89 (22.47)	21/89 (23.60)
	Uganda	4/94 (4.25)	10/ 94 (10.63)
*G. medicorum*	Burkina Faso	8/154 (5.20)	61/154 (39.61)
*G. m. morsitans*	Kenya	54/85 (63.52)^a^	2/ 85 (2.35)
	Tanzania	62/81 (76.54)^a^	43/81 (53.08)
	Zambia	8/64 (12.50)^b^	31/64 (48.43)
	Zimbabwe	32/139 (23.02)^b^	75/139 (53.95)
*G. m. submorsitans*	Burkina Faso	1/343 (0.30)	62/343 (18.07)
*G. pallidipes*	Ethiopia	94/459 (20.48)^a,b,c^	92/459 (20.04)
	Kenya	214/834 (25.65)^a,c^	425/834 (50.95)
	Tanzania	162/217 (74.65)^a,b^	81/217 (37.32)
	Uganda	87/116 (75.00)^a,b^	9/116 (7.75)
	Zimbabwe	7/72 (9.72)^a,c^	38/72 (52.77)
	Zambia	3/146 (2.05)^a,b,c^	66/146 (45.20)
*G. p. palpalis*	Democratic R. of Congo	4/35 (11.42)	1/35 (2.86)
*G. p. gambiensis*	Burkina Faso	2/943 (0.21)	235/943 (24.92)^a^
	Guinea	90/314 (28.66)	7/314 (2.22)^b^
	Mali	0/364 (0.00)	25/364 (6.87)^b,c^
	Senegal	0/547 (0.00)	78/547 (14.25)^c^
*G. tachinoides*	Burkina Faso	0/834 (0.00)	140/834 (16.79)^a^
	Ghana	0/234 (0.00)	143/234 (61.11)^b^
Total (average)		871/6860 (12.69)	1736/6860 (25.30)

*Values indicated by the same lower-case letter do not differ significantly at the 5% level.

**Table 4:** Permanova analysis for Countries and tsetse species for *Sodalis* and trypanosome (single and multiple) infection prevalence.

Source	df	SS	MS	Pseudo-F	P (perm)	Unique perms
Countries	11	13,040	1185.4	2.6004	**0.009**	998
Species	7	7899.8	1128.5	2.4756	**0.041**	999
Residuals	5	2279.3	455.87			
Total	25	34,074				

Within the table, statistically significant differences (*P* < 0.05) can be seen in bold values in countries and tsetse species. Perm(s) = permutations.

**Figure 1:**
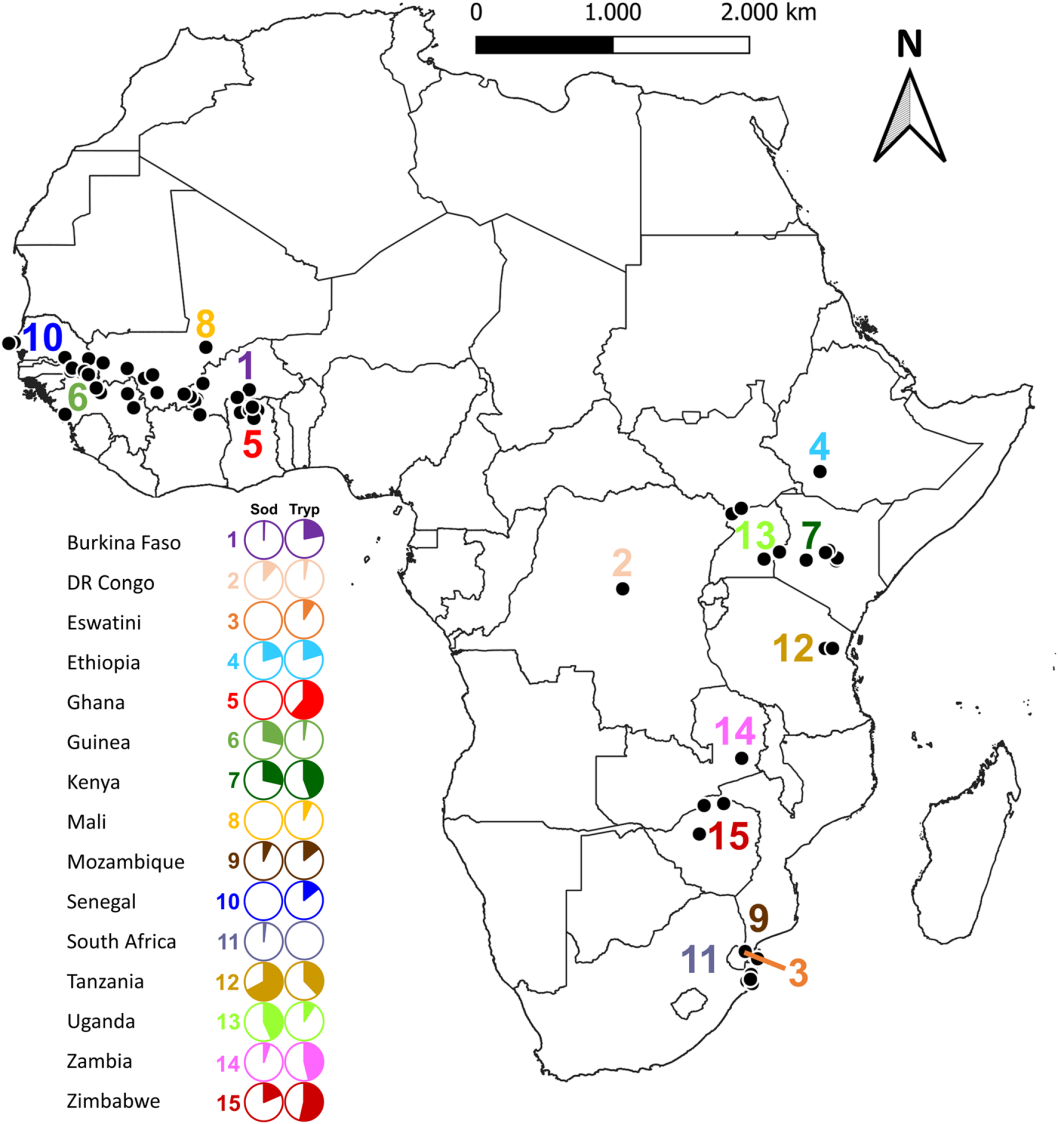
The geographical locations of tsetse samples in Africa. Circles indicate the total prevalence of *Sodalis* and *Trypanosoma* per country. Black dots indicate samples collection site(s) per country.

Regardless of tsetse taxon, in west African countries the average *Trypanosoma* prevalence was 20% (*n* = 3733), with the highest prevalence recorded in Ghana (61%) and the lowest recorded in Guinea (2.2%). The prevalence in Burkina Faso, Mali and Senegal was 21.9, 6.9 and 14.2% respectively (Fig. 1, and Table 1). In east, central and southern African countries, the *Trypanosoma* infection prevalence was a bit higher than in west African countries with an averaged infection of 31.5% (*n* = 3127), with the highest prevalence (53.6%) in Zimbabwe and lowest prevalence (2.9%) in DRC. No *Trypanosoma* infection was detected in Eswatini (Fig. 1 and Table 1). Regardless of the country, *Trypanosoma* prevalence varied from one taxon to another, and *G. m. morsitans* showed the highest *Trypanosoma* prevalence (41%) followed by *G. pallidipes* (38.5%) and the lowest prevalence was detected in *G. brevipalpis* (9.71%) in east, central and southern Africa. In west Africa, *G. medicorum* showed the highest *Trypanosoma* prevalence (39.5%) and the lowest prevalence was detected in *G. p. palpalis* (2.8%) (Table 2).

**Figure 2:**
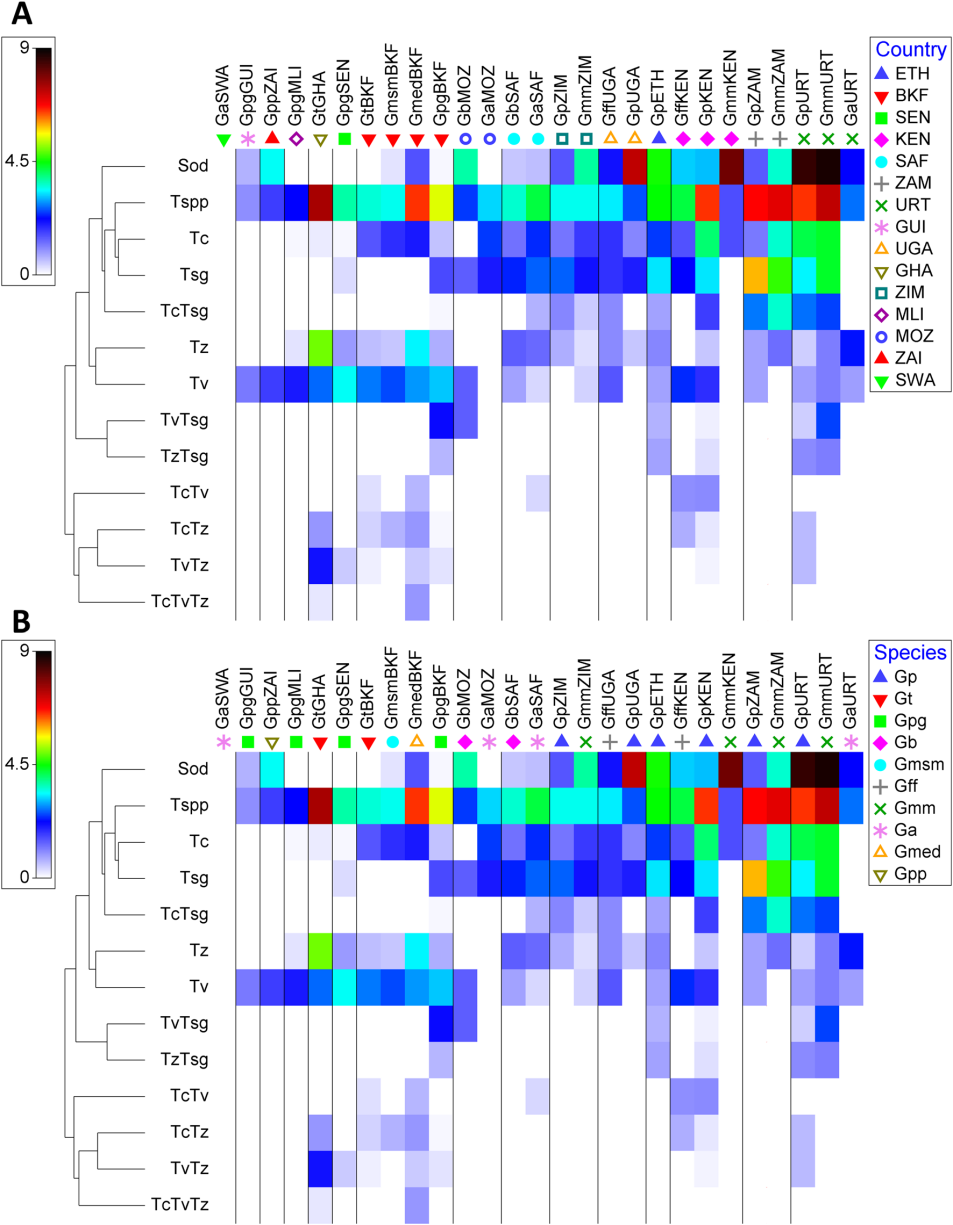
Prevalence of the *Sodalis* and *Trypanosoma* (single and multiple) infections per country (**A**) and tsetse species (**B**). Prevalence data were square root transformed and averaged based on country-species and the matrix display was conducted in PRIMER version 7 + software. Tree on the left of the matrix is the similarity dendrogram based on the similarity index of the square root of the prevalence values. The colour index is the square root of the prevalence values ranged 0–9 which is the square root of 0–81% prevalence. Country abbreviations follow the UNDP list of country codes https://web.archive.org/web/20060713221355/http://refgat.undp.org/genericlist.cfm?entid=82&pagenumber=1&requesttimeout=360 as follows: BKF: Burkina Faso; ETH: Ethiopia; GHA: Ghana; GUI: Guinea; KEN: Kenya; MLI: Mali; MOZ: Mozambique; SAF: South Africa; SWA: Eswatini; ZAI: Democratic Republic of the Congo; ZAM: Zambia; ZIM: Zimbabwe. Tsetse, *Sodalis* and *Trypanosoma* taxa were abbreviated as following: Ga: *Glossina austeni*; Gb: *G. brevipalpis*; Gff: *G. fuscipes fuscipes*, Gmm: *G. morsitans morsitans*; Gmsm: *G. m. submorsitans*; Gpg: *G. palpalis gambiensis*; Gpp: *G. palpalis palpalis*. Sod: *Sodalis*, Tc: *Trypanosoma. congolense* savannah; *T. congolense* kilifi; *T. congolense* forest, Tsg: *T. simiae*; *T. simiae* Tsavo; *T. godfreyi*, Tv: *T. vivax,* Tz: *T. brucei brucei, T. b. gambiense, T. b. rhodesiense.*

Some tsetse taxa were collected from several countries as presented in Fig. 2 and Table 3. The highest *Trypanosoma* prevalence was recorded in *G. tachinoides* in Ghana (61%). This was followed by high prevalence in *G. m. morsitans* collected from Zimbabwe (53.9%), Tanzania (53%) and Zambia (48.4%). *G. pallidipes* from Zimbabwe, Kenya, Zambia and Tanzania also showed high *Trypanosoma* prevalence of 52.7%, 50.9%, 45.2% and 37.3%, respectively. The lowest *Trypanosoma* prevalence was found in *G. p. gambiensis* from Guinea (2.2%). Based on the *Trypanosoma* prevalence presented in Fig. 2 and Table 3, the tested samples can be categorized as: (i) tsetse samples with high prevalence (> 35%) detected in *G. tachinoides* from Ghana; *G. medicorum* from Burkina Faso, *G. pallidipes* from Kenya, Zambia, and Zimbabwe, *G. m. morsitans* from Tanzania, Zambia, and Zimbabwe; (ii) tsetse samples with medium prevalence (10–35%) detected in *G. austeni* from South Africa, *G. f. fuscipes* from Kenya and Uganda, *G. m. submorsitans* from Burkina Faso, *G. p. gambiensis* from Burkina Faso and Senegal and *G. tachinoides* from Burkina Faso; (iii) tsetse samples with low prevalence (< 10%) detected in the rest of the samples listed in Table 3 except the *G. austeni* collected from Eswatini. Despite the difference in *Trypanosoma* prevalence for each tsetse species, the differences were significant only in *G. p. gambiensis* (*X*^2^ = 26.71, *df* = 4, *P* < 0.001) and *G. tachinoides*, (*X*^2^ = 9.38, *df* = 1, 2, *P* = 0.002). In contrast, no significant difference was detected between countries for *G. austeni* (*X*^2^ = 1.47, *df* = 4, *P* = 0.688), *G. brevipalpis* (*X*^2^ = 0.34, *df* = 2, *P* = 0.559), *G. f. fuscipes* (*X*^2^ = 0.15, *df* = 2, *P* = 0.702), *G. m. morsitans* (*X*^2^ = 1.04, *df* = 3, *P* = 0.593) and *G. pallidipes* (*X*^2^ = 4.983, df = 1,6, *P* = 0.418) (Table 3). No *Trypanosoma* infection was recorded in *G. austeni* from Eswatini. The best glm model (lowest AICc) selected for the overall *Trypanosoma* prevalence retained the countries as variables that fitted the data well (AICc = 1521.35) (Supplementary File 1).

### Prevalence of different *Trypanosoma* taxa and mixed infections

The above-mentioned prevalence of *Trypanosoma* infection was comprised of several different *Trypanosoma* species and sub-species. Based on the size of the amplified fragment by PCR, the *Trypanosoma* infection was categorized into four groups: (i) the Tc group including the different forms of *T. congolense*; (ii) Tv group including *T. vivax* infections; (iii) *T. brucei* sspp (Tz) group including *T. b. brucei, T. b. gambiense, T. b. rhodesiense,* and infections; and (iv) Tsg group including the infections with *T. simiae, T. simiae* tsavo and *T. godfreyi*. The screening results revealed that tsetse flies could be infected with single or multiple (double or triple) taxa of *Trypanosoma*, and the proportion of the infections with the different *Trypanosoma* taxa and the mixed infection varied with country (*X*^2^ = 63.56, *df* = 14, P < 0.001) and species (*X*^2^ = 21.86, *df* = 9, P < 0.001) (Supplementary File 1).

The prevalence of the different *Trypanosoma* species with respect to the above-mentioned groups, indicate that infections with the Tsg group was the highest regardless of countries or tsetse species with an average of 7.06%. The infection rate was higher (14.13%) in east, central and southern African countries than in west Africa (1.13%). Tv infection averaged at 6.75% but with higher prevalence in west African countries (10.37%) than in east, central and southern Africa (2.43%). The prevalence of Tc infection was lower than Tv and Tsg group with an average of 4.78% with higher prevalence in central and southern Africa (8.38%) than in west Africa (1.77%). The Tz group had the lowest prevalence with an average of 2.29%. Like Tv infection, the Tz prevalence was higher in west Africa (3.16%) than central and southern Africa (1.25%).

The prevalence of infection by a single *Trypanosoma* group varied significantly from one country to another and from one tsetse species to another. For Tc, Tv, Tz and Tsg the infection prevalence varied significantly with country (*X*^2^ = 47.74, *df* = 14, *P* < 0.001, *X*^2^ = 27.40, *df* = 14, *P* = 0.01705, *X*^2^ = 106.11, *df* = 14, *P* = 0. 001 and, *X*^2^ = 44.74, *df* = 14, *P* = 0.001 respectively). Regardless of tsetse species, the highest infection rate for Tc, Tv, Tz and Tsg was found in Tanzania (14.20%), Ghana (14.10%), Ghana (19.66%) and Zimbabwe (39.81%), respectively (Supplementary Table 1). Similarly, the prevalence of Tc, Tz and Tsg varied significantly with tsetse species (*X*^2^ = 40.364, df = 1.9, *P*  <<  0.001, *X*^2^ = 58.253, df = 1.9, *P* << 0.001 and *X*^2^ = 34.871, df = 1.9, *P* << 0.001, respectively), however no significant difference was found in Tv prevalence between tsetse species (*X*^2^ = 5.475, df = 1.9, *P* = 0.07868). Regardless of the country, the highest infection rate of Tc, Tv, Tz and Tsg was found in *G. pallidipes* (10.68%), *G. tachinoides* (12.92%), *G. medicorum* (13.64%) and *G. m. morsitans* (22.76%), respectively (Supplementary Table 2). No Tc infection was found in samples of *G. austeni* collected from Eswatini and Tanzania, *G. brevipalpis* from Mozambique, *G. p. palpalis* from DRC and *G. p. gambiensis* from Guinea. In addition, no Tv infection was detected in *G. austeni* collected from Eswatini and Mozambique, *G. m. morsitans* from Kenya and Zambia, *G. pallidipes* from Uganda and Zimbabwe. For Tz, *G. austeni* collected from Eswatini and Mozambique, *G. brevipalpis* from Mozambique, *G. f. fuscipes* from Kenya, *G. m. morsitans* from Kenya and Zambia, *G. p. palpalis* from DRC and *G. p. gambiensis* from Guinea did not show any infection (Fig. 2 and Supplementary Table 3).

Mixed infections of *Trypanosoma* groups (double or triple) are rare events with an average prevalence between 0.09 and 1.71% regardless of country or tsetse species. However, double infections seem to be more frequent in some countries than others (*X*^2^ = 35.01, *df* = 14, *P* = 0.001) for Tv–Tz and in some tsetse species than others (*X*^2^ = 21.20, *df* = 9, *P* = 0.012) for Tv–Tz (Supplementary File 1). The highest prevalence of the mixed infections Tv–Tz and Tc–Tz were observed in Ghana with 12.39% and 10.68%, respectively, regardless of tsetse species. Although the average Tc–Tsg prevalence was higher than that of Tv–Tz and Tc–Tz, the highest mixed infection with it was found in Zambia with 9.05%. Regardless of the country, the highest mixed infection of Tc–Tsg detected per tsetse species was ~ 5% in *G. m. morsitans* and *G. pallidipes*. The mixed infection of Tsg with either Tv or Tz or both was lower than 2% regardless of the country or tsetse species. Taking into account both the country and tsetse species, the highest mixed infection of Tc–Tsg (12.5%) was detected in *G. m. morsitans* in Zambia. However, the highest prevalence of Tc–Tz (10.68%) and Tv–Tz (12.39%) was detected in *G. tachinoides* from Ghana. Although the average prevalence of Tv–Tsg was low (0.54%), a relative high infection rate of 6.17% was found in *G. m. morsitans* from Tanzania.

A triple infection of *Trypanosoma* groups (Tc–Tv–Tz) was only detected in *G. medicorum* from Burkina Faso (1.30%) and *G. tachinoides* from Ghana (1.71%) (Fig. 2 and Supplementary Table 3, Supplementary File 1).

### Prevalence of *Sodalis* infection

The prevalence of *Sodalis* infection based on the PCR results varied significantly with country (*X*^2^ = 108.02, df = 1, 14, *P* << 0.001) and tsetse species (*X*^2^ = 69.60, *df* = 9, *P* < 0.001). The best glm model (lowest AICc) selected for the overall *Sodalis* prevalence retained the countries, the species and their interaction (where possible) as variables that fitted the data well (AICc = 1296.12). Similar to the prevalence of *Trypanosoma*, the average *Sodalis* prevalence in east, central and southern Africa (24.6%) was higher than in west Africa (2.70%). Regardless of tsetse species, the highest prevalence of *Sodalis* infection was found in Tanzania (67.1%) followed by Uganda (43.3%), Kenya (28.5%) and Ethiopia (20.48%) (Table 1). The highest prevalence of *Sodalis* infection in west Africa was found in Guinea (28.6%). No *Sodalis* infection was found in Ghana, Mali, Senegal or Eswatini. Regardless of the country, the highest *Sodalis* prevalence per tsetse species was detected in *G. m. morsitans* (42.27%) followed by *G. pallidipes* (30.74%). No *Sodalis* infection was detected in *G. tachinoides.* The prevalence of *Sodalis* infection changed when both the countries and tsetse species are taken into consideration (Table 4). Based on the *Sodalis* prevalence the tsetse samples can be categorized into four groups: (i) samples with high prevalence (> 50%) (ii) samples with medium prevalence (between < 10% and > 50%) (iii) samples with low prevalence (between > 0% and 10%) and (iv) samples with no *Sodalis* infection as shown in Fig. 2 and Table 4. The samples showing high *Sodalis* prevalence includes *G. m. morsitans* from Kenya (63.5%) and Tanzania (76.5%) and *G. pallidipes* from Tanzania (74.6%) and Uganda (75%), however the samples with no *Sodalis* infection includes *G. austeni* from Eswatini, *G. p. gambiensis* from Mali and Senegal and *G. tachinoides* from Burkina Faso and Ghana indicating that there is 95% confidence that the infection rate is less than or equal to 10%, 0.82%, 0.55%, 1.28% and 0.36%, respectively.

### Interactions between *Sodalis* and *Trypanosoma* infections

#### Prevalence of co-infections of *Sodalis* with *Trypanosoma*

**Figure 3:**
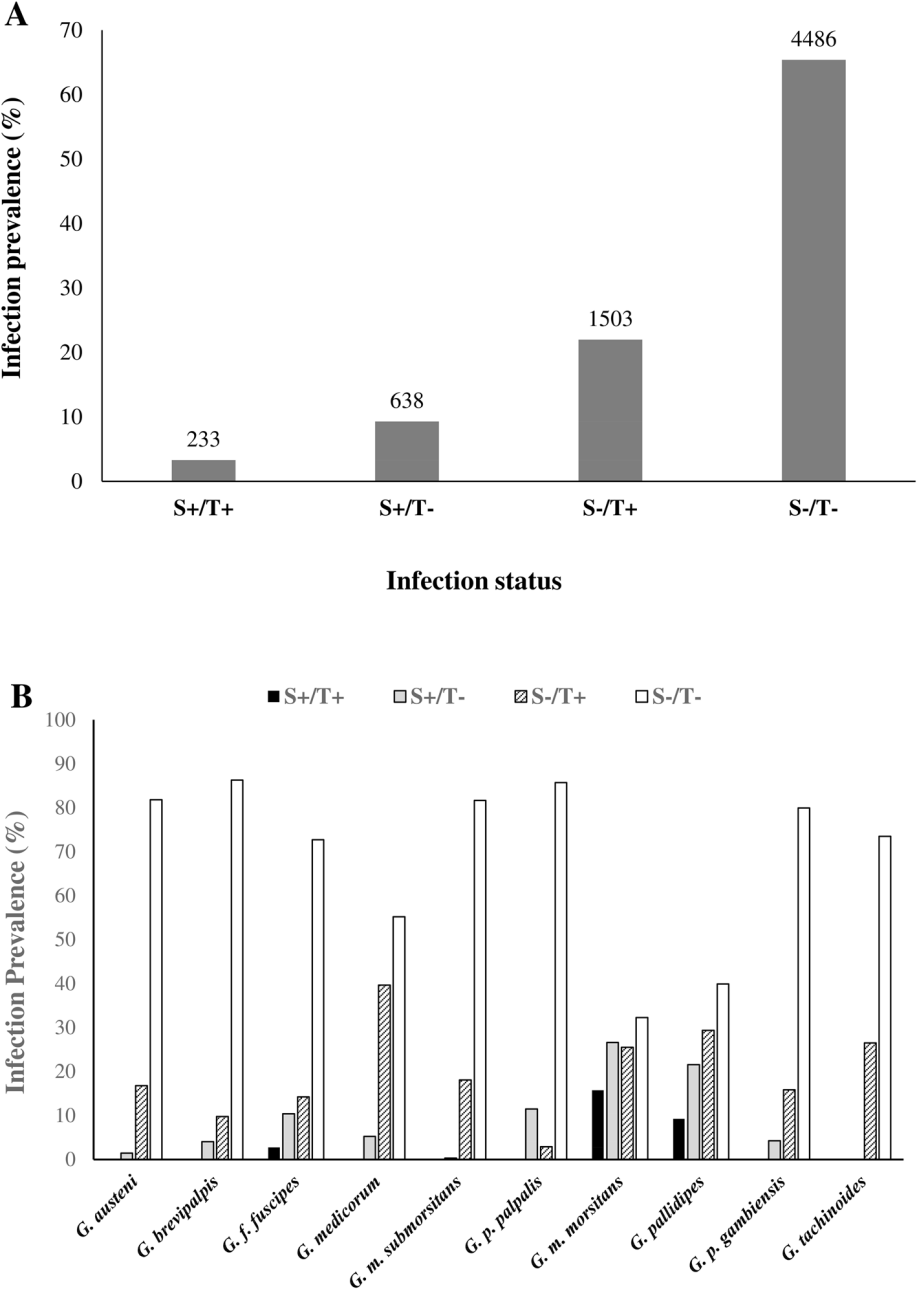
Prevalence of coinfection of *Sodalis* and *Trypanosoma* infection in wild tsetse populations. (**A**) Prevalence of coinfection, (**B**) prevalence of coinfection by tsetse taxa.

The screening results indicated that the single infection rate was 9.3% (n = 638) and 21.9% (n = 1503) for *Sodalis* and *Trypanosoma,* respectively, over all taxa and countries (Fig. 3A). No *Sodalis* infection was found in *G. tachinoides*, and therefore was excluded from the analysis. A Cochran–Mantel–Haenszel test for repeated tests of independence showed that infection with *Sodalis* and *Trypanosoma* did deviate from independence across all taxa (χ^2^_MH_ = 41.73, df = 1, *P* < 0.001) and individual Chi squared tests for independence for each taxon showed significant deviation from independence at the Bonferroni corrected α = 0.00833 in *G. pallidipes* (*P* < 0.001) and *G. p. gambiensis* (*P* < 0.001) (Supplementary Table 4). The prevalence of coinfection of *Sodalis* and *Trypanosoma* in wild tsetse populations varied with tsetse taxon and location. No coinfection was found in many taxa and many locations. The co-infection was found only in *G. f. fuscipes* (2.73%), *G. m. morsitans* (15.72%) and *G. pallidipes* (9.22%) in east, central and southern Africa (Fig. 3B, Table 5 and Supplementary Table 4).

**Table 5:** Distribution of the association between the presence of *Trypanosoma* spp and the presence of *Sodalis* according to the tsetse species and the country.

Glossina taxon	Country (Area, Collection Date)	N	S^+^/T^+^	S^+^/T^−^	S^−^/T^+^	S^−^/T^−^	χ^2^	*P*
*G. austeni*	Tanzania (Jozani, 1997)	4	0	0	1	3		
*G. austeni*	Tanzania (Zanzibar, 1995)	6	0	1	0	5		
*G. austeni*	Tanzania (Uguja Island, 1995)	30	0	2	3	25		
*G. austeni*	South Africa (North eastern Kwazulu Natal, 1999)	39	0	2	2	35		
*G. austeni*	South Africa (Lower Mkhuze, 2018)	53	0	0	23	30		
*G. austeni*	South Africa (Saint Lucia, 2018)	57	0	0	22	35		
*G. austeni*	South Africa (False Bay Park, 2018)	77	0	0	2	75		
*G. austeni*	Mozambique (Reserva Especial de Maputo, 2019)	50	0	0	5	45		
*G. austeni*	Eswatini (Mlawula Nature Reserve, 2019)	30	0	0	0	30		
*G. austeni*	All locations	346	0	5	58	283	1.02	0.31
*G. brevipalpis*	South Africa (North eastern Kwazulu Natal, 1995)	50	0	0	2	48		
*G. brevipalpis*	South Africa (Phinda, 2018)	170	0	7	0	163		
*G. brevipalpis*	South Africa (Saint Lucia, 2018)	30	0	0	13	17		
*G. brevipalpis*	South Africa (Hluhluwe, 2018)	50	0	0	16	34		
*G. brevipalpis*	Mozambique (Reserva Especial de Maputo, 2019)	50	0	7	3	40		
*G. brevipalpis*	All locations	350	0	14	34	302	1.57	0.21
*G. f. fuscipes*	Uganda (Buvuma island, 1994)	94	0	4	10	80		
*G. f. fuscipes*	Kenya (Ikapolock, 2007) ^1^	51	5	15	14	17		
*G. f. fuscipes*	Kenya (Obekai, 2007)	38	0	0	2	36		
*G. f. fuscipes*	All locations	183	5	19	26	133	0.3	0.59
*G. medicorum*	Burkina Faso (Comoe, 2008)	94	0	8	32	54		
*G. medicorum*	Burkina Faso (Folonzo, 2008)	60	0	0	29	31		
*G. medicorum*	All locations	154	0	8	61	85	5.53	0.02
*G. m. submorsitans*	Burkina Faso (Comoe, 2007)	206	0	0	20	186		
*G. m. submorsitans*	Burkina Faso (Folonzo, 2008)	134	0	1	42	91		
*G. m. submorsitans*	Burkina Faso (Sissili, 2008)	3	0	0	0	3		
*G. m. submorsitans*	All locations	343	0	1	62	280	0.22	0.64
*G. p. palpalis*	Democratic Republic of Congo (Zaire, 1995)	35	0	4	1	30		
*G. m. morsitans*	Tanzania (Kwekivu 2, 2005)	81	35	27	9	10		
*G. m. morsitans*	Zambia (Mfuwe, Eastern Zambia, 2007)	64	1	7	30	26		
*G. m. morsitans*	Zimbabwe (Mukondore, 2007)	13	1	2	0	10		
*G. m. morsitans*	Zimbabwe (M. chiuyi, 2007)	9	0	1	0	8		
*G. m. morsitans*	Zimbabwe (Rukomeshi, 2006)	15	0	3	0	12		
*G. m. morsitans*	Zimbabwe (Kemukura, NA)	18	0	4	1	13		
*G. m. morsitans*	Zimbabwe (Mushumbi, 2006)	6	0	0	2	4		
*G. m. morsitans*	Zimbabwe (Makuti, 2006)	78	19	2	52	5		
*G. m. morsitans*	Kenya (Kari, 2006)	85	2	52	0	31		
*G. m. morsitans*	All locations	369	58	98	94	119	1.8	0.18

Glossina taxon	Country (Area, Collection Date)	N	S^+^/T^+^	S^+^/T^−^	S^−^/T^+^	S^−^/T^−^	χ^2^	*P*
*G. pallidipes*	Zambia (Mfuwe, Eastern Zambia, 2007)	146	2	1	64	79		
*G. pallidipes*	Kenya (Mwea, Katotoi, Emsos, Kari, Kiria, Koibos,Meru and Ruma national park, 2007)	834	88	126	337	283		
*G. pallidipes*	Ethiopia (Arba Minch, 2007)	459	15	79	77	288		
*G. pallidipes*	Tanzania (Kwekivu 1, 2005)	217	54	108	27	28		
*G. pallidipes*	Zimbabwe (Mushumbi 2006)	26	1	0	4	21		
*G. pallidipes*	Zimbabwe (Gokwe, 2006)	4	0	0	0	4		
*G. pallidipes*	Zimbabwe (Rukomeshi, 2006)	4	0	0	0	4		
*G. pallidipes*	Zimbabwe (Makuti, 2006)	38	6	0	27	5		
*G. pallidipes*	Uganda (Lira,Omogo, Budaka, Moyo, NA)	116	4	83	5	24		
*G. pallidipes*	All locations	1844	170	397	541	736	25.4	0
*G. p. gambiensis*	Burkina Faso (Lorepeni)	10	0	0	8	2		
*G. p. gambiensis*	Burkina Faso (Bouroum bouroum)	18	0	0	16	2		
*G. p. gambiensis*	Burkina Faso (Kourignon)	24	0	0	10	14		
*G. p. gambiensis*	Burkina Faso (Kampty)	98	0	0	85	13		
*G. p. gambiensis*	Burkina Faso (Ouarkoye)	5	0	0	5	0		
*G. p. gambiensis*	Burkina Faso (Dedougou)	57	0	0	33	24		
*G. p. gambiensis*	Burkina Faso (Bama)	77	0	0	0	77		
*G. p. gambiensis*	Burkina Faso (Comoe)	123	0	0	3	120		
*G. p. gambiensis*	Burkina Faso (Folonzo)	212	0	2	25	185		
*G. p. gambiensis*	Burkina Faso (Kartasso)	136	0	0	0	136		
*G. p. gambiensis*	Burkina Faso (Kenedougou)	41	0	0	0	41		
*G. p. gambiensis*	Burkina Faso (Moussodougou)	142	0	0	49	93		
*G. p. gambiensis*	Guinea (Bafing)	33	0	0	1	32		
*G. p. gambiensis*	Guinea (Dekonkore)	16	0	0	1	15		
*G. p. gambiensis*	Guinea (Kangoliya	126	0	90	0	36		
*G. p. gambiensis*	Guinea (Kerfala	13	0	0	1	12		
*G. p. gambiensis*	Guinea (Kifala)	30	0	0	0	30		
*G. p. gambiensis*	Guinea (Lemonako)	20	0	0	0	20		
*G. p. gambiensis*	Guinea (Mimi)	45	0	0	1	44		
*G. p. gambiensis*	Guinea (Tinkisso)	31	0	0	2	29		
*G. p. gambiensis*	Mali	364	0	0	25	339		
*G. p. gambiensis*	Senegal	547	0	0	79	469		
*G. p. gambiensis*	All locations	2168	0	92	343	1733	18.06	0
*G. tachinoides*	Burkina Faso	834	0	0	140	694		
*G. tachinoides*	Ghana	234	0	0	143	91		
*G. tachinoides*	All locations	1068	0	0	283	785		

#### Impact of co-infection on *Trypanosoma* and *Sodalis* density

**Figure 4:**
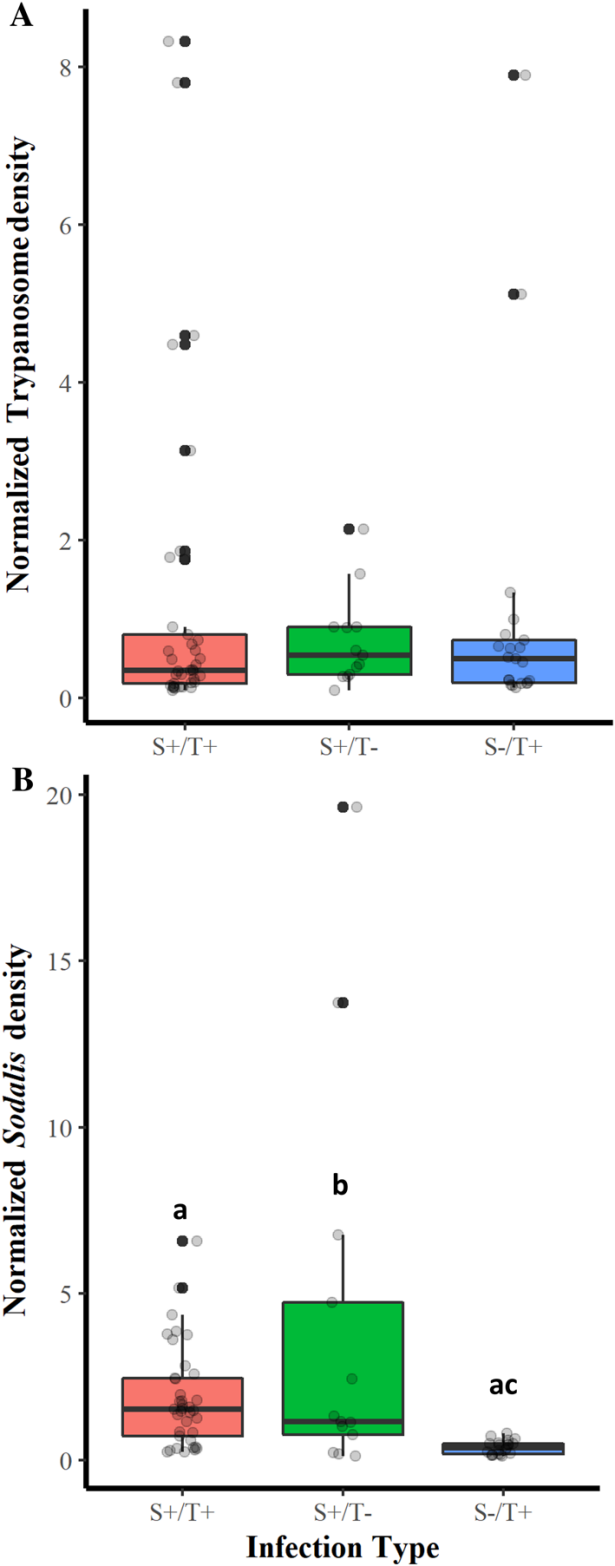
Impact of co-infection with *Trypanosoma* and *Sodalis *on *Trypanosoma* (**A**) and *Sodalis* (**B**) density in *Glossina pallidipes* and *G. m. morsitans*. Bars marked with the same lower-case letter do not differ significantly at the 0.05 level.

Attempts were to assess the density of *Trypanosoma* and *Sodalis* under single (S^−^/T^+^) and (S^+^/T^−^) or double infection (S^+^/T^+^) conducted using qPCR with primers mentioned in Supplementary Table 5. The results show that *Sodalis* infections did not have a significant impact on *Trypanosoma* density (*X*^2^ = 0.648, *df* = 2, *P* = 0.723), however the median value of (S^+^/T^+^) files were slightly (S^−^/T^+^) lower than (S^+^/T^−^) and (S^−^/T^+^) flies and the number of outlier samples with higher trypanosome density (S^−^/T^+^) flies (Fig. 4A). *Trypanosoma* infections significantly reduced the density of *Sodalis* as indicated by comparing (S^+^/T^+^) flies with (S^+^/T^−^) flies (*P* = 0.014) although the median values in (S^+^/T^+^) files is higher than the other samples indicating that the increased of *Sodalis* density in (S^+^/T^−^) might be affected with the outlier flies with high *Sodalis* density (Fig. 4B). No significant different was found in the *Trypanosoma* density determined by qPCR in the flies tested negative (S^+^/T^−^) or positive (S^+^/T^+^) and (S^−^/T^+^) with the standard PCR, however, *Sodalis* density showed significant difference between flies with different infection type (*X*^2^ = 14.54, df = 2, *P* < 0.001) (Fig. 4B). The results showed no correlation between *Sodalis* and *Trypanosoma* density (r = 0.007, t = 0.055, *df* = 69, *P* = 0.9561) Supplementary Fig. 2, Supplementary File 1).

## Discussion

The implementation of the SIT in the context of an AW-IPM strategy to eradicate tsetse flies relies on the release of sterile males in the targeted area. This was successful in eradicating a population of *G. austeni* from Unguja Island of Zanzibar^37^ and significant progress has been made in the eradication programme implemented against *G. p. gambiensis* in the Niayes area of Senegal^38^. However, as both male and female tsetse flies are vectors of *Trypanosoma* species, the release of large numbers of sterile male flies bears a potential risk of temporarily increasing disease transmission during the initial release phase of an SIT programme^39^. Therefore, mitigating measures are required to reduce or eliminate this potential risk, especially in areas where sleeping sickness (HAT) is endemic. To date, to mitigate such risks, sterile males are offered two or three blood meals mixed with the trypanocidal drug isometamidium chloride, before being released which reduces the risk of *Trypanosoma* transmission significantly but does not eliminate it^40,41^. In addition, other approaches were proposed to minimize such risks such as paratransgenesis^42,43^ and combining paratransgenesis with SIT^44^.

The vector competence of tsetse flies for different trypanosome species is highly variable and is suggested to be affected by various factors, amongst which bacterial endosymbionts. Here, the interaction of *Sodalis glossinidius* with tsetse trypanosome infection is still under debate. Several studies reported a potential positive correlation between *Sodalis* and *Trypanosoma* infections^28,30,32,36,45–48^, leading to the hypothesis that *Sodalis* might facilitate the establishment of *Trypanosoma* infections in the tsetse midgut^23,26,27^. However, other studies indicated the lack of correlation between *Sodalis* and *Trypanosoma* infection^34–36^. The presence of *Sodalis* infections in tsetse rearing colonies has been well studied and previous studies indicated that *Sodalis* is more frequently present in colonized tsetse flies than in wild tsetse populations^36,49^ with a prevalence of 80 and 100% in colonized *G. m. morsitans* and *G. p. gambiensis,* respectively^49,50^, which is higher than the symbiont prevalence in wild populations of these tsetse species. This seems to indicate that the rearing process of tsetse flies favours the transmission and spread of *Sodalis* infections within the colonized population. Recently, colonies of *G. pallidipes, G. p. gambiensis, G. f. fuscipes, G. m. morsitans, G. m. centralis* and *G. m. submorsitans* maintained at the FAO/IAEA Insect Pest Control Laboratory were screened for *Sodalis* infections and showed a 100% prevalence of *Sodalis;* only the *G. brevipalpis* colony had a lower prevalence of 95% (data not shown). Taken into consideration that mass-rearing conditions enhances *Sodalis* infections and that *Sodalis* infections might facilitate the establishment of a *Trypanosoma* infection in the midgut, sterile male tsetse flies that are derived from colonies might be effective vectors for different *Trypanosoma* species and, therefore, might increase the trypanosome transmission after flies being released. It is therefore important that the managers and planners of SIT programmes are aware which tsetse species show a positive correlation between *Sodalis* and *Trypanosoma* infections to be able to take the necessary mitigating actions.

Various studies have examined the prevalence of *Sodalis* and *Trypanosoma* species in wild tsetse populations^30,32,35,45,51^, but our study presents for the first time the prevalence of *Sodalis* and *Trypanosoma* species on a continent-wide scale. In addition, the DNA extraction and PCR methods we have used were standardized and were all carried out in one laboratory to avoid discrepancies in the results due to different handling of tsetse samples or to different methods to discriminate trypanosome species in tsetse tissues. Our results indicate that *Sodalis* and *Trypanosoma* prevalence varied with tsetse species and geographical location (with an overall trypanosome prevalence of 23.5%), which agrees with many previous studies^52^. A high *Trypanosoma* prevalence (> 30%) was found in *G. m. morsitans* and *G. pallidipes* from central and east Africa. This finding is in agreement with previous reports on *G. m. morsitans* and *G. m. centralis* from Zambia^36,52^ and *G. m. morsitans* sampled in Malawi^53^. Moreover, a high prevalence of *Trypanosoma* infection in *G. pallidipes* was also previously reported in Tanzania^54^ and Kenya^33^. However, another study in northern Tanzania indicated a lower prevalence of *Trypanosoma* infection (< 10%) both in *G. m. morsitans* and *G. pallidipes*^55^.

Our study showed that the prevalence of different *Trypanosoma* species and or subspecies can be different in different tsetse taxa. In *G. tachinoides* in Ghana, the *Trypanosoma vivax* (Tv) infection was high (> 10%) as well as the infections of the *T. brucei* sspp (Tz) and the *T. simiae/T. godfreyi* (Tsg) group and the mixed infections of Tv–Tsg. In contrast, the prevalence of *T. congolense* was very low. These results are in agreement with the prevalence of *T. brucei* s.l (11%) and *T. congolense* forest type (2.6%) reported in the same tsetse species in Cameroon. The same study reported a prevalence of 13.7% of *T. congolense* savannah type^35^, which was not observed in our study. Our results of trypanosome infection rates in *G. tachinoides* also agree with former studies^56,57^, except for T,c for which a high fly infection rate (31.8%) was previously shown^57^. The Tc infection rates in our study were high in *G. pallidipes* and *G. m. morsitans;* for the latter tsetse fly species, a study in Malawi reported a high prevalence for *T. brucei* (64.4%) but much lower for all other *Trypanosoma* infections(< 10%)^58^. The mixed infection of *Trypanosoma* species/subspecies is in agreement with previous reports^35,52,57,59^.

Likewise, the prevalence of *Sodalis* infection varied significantly with tsetse taxon and location and the highest prevalence was found in *G. m. morsitans* and *G. pallidipes*. Our results agree with the high prevalence of *Sodalis* reported in *G. pallidipes* (~ 50%) in one location in Kenya regardless of the fly age^33^; however, the same study reported low *Sodalis* prevalence in another location. In another study in Kenya, Wamwiri et al.,^32^ reported moderate *Sodalis* prevalence in *G. pallidipes* (16%) and low prevalence in *G. austeni* (3.7%), which is in agreement with our results. On the other hand, our results are different from the low prevalence (< 8%) found in *G. m. morsitans* and *G. pallidipes* in Zambia^36^. In another study in Zambia, *Sodalis* prevalence in *G. m. centralis*, was reported to be 15.9% with no significant difference between inter-site prevalence^52^. The prevalence of *Sodalis* in *G. brevipalpis* in our study was found to be low (< 2.3%) which contradicts with the high prevalence (93.7%) reported in this species in Zambia^36^. In the DRC, the global prevalence of *Sodalis* in *G. fuscipes quanzensis* midgut averaged 15.5%, but in certain locations the prevalence exceeded 40%^60^. In Nigeria, *Sodalis* prevalence in *G. p. palpalis* and *G. tachinoides* was 35.7%^61^ which is higher that the prevalence reported in our study for both species.

Our data indicate that the *Trypanosoma* and *Sodalis* infections were very low or absent in some tsetse taxa from certain locations such as *G. austeni* in Eswatini for *Trypanosoma* and *Sodalis* infections and several species in west Africa for *Sodalis*. The lack of *Sodalis* and/or *Trypanosoma* infection in these samples might be due to (i) low number of tested samples (ii) the use of the DNA extracted from the whole body of tsetse adults (iii) the possibility of the collected samples being infected with different strains/genotypes that might not be detected with the primers used and (iv) the infection of *Sodalis* and *Trypanosoma* are under the detection limit of the used PCR. It is important to note that due to the high number of samples tested in our study, the more sensitive nested PCR to detect low infection level was excluded due to logistic reasons.

Our results indicate significant deviation from independence (correlation) of *Sodalis* and *Trypanosoma* infections in *G. medicorum, G. p. gambiensis* and *G. pallidipes*. However, the lack of detection of any tsetse adult with co-infection of *Sodalis* and *Trypanosoma* in *G. medicorum,* and *G. p. gambiensis* might indicate a negative correlation. Such negative trend might be supported by the lower density of *Sodalis* in the flies with co-infection (S^+^/T^+^) compared to these with *Sodalis* infection only (S^+^/T^−^). On other hand the lack of impact of *Sodalis* infection on *Trypanosoma* density does not support the negative trend and agreed with the results of Trappeniers et al.,^24^ reported on colonized flies. This results also agreed with previous results reporting the absence of direct correlation between the presence of *Sodalis* and the acquisition of a *Trypanosoma* infection^63^. However, an inverse correlation was reported between *Sodalis* and the vector competence where the presence of *Sodalis* in both midgut and proboscis of *G. p. gambiensis* was associated with its status as a poor vector, whereas it is not found in the proboscis of *G. m. morsitans* (major vector). It is worth noting that all previous studies of *Sodalis* infection in *G. p. gambiensis* and its interaction with *Trypanosoma* infection was carried out with flies reared under laboratory conditions^28,29,64^. The correlation between *Sodalis* and *Trypanosoma* infection in *G. pallidipes* is positive, evidenced with the relative high number (n = 170) of tsetse with co-infection. This positive correlation was also found in *G. pallidipes* from Kenya although with too few flies with co-infection to enable us to draw a definite conclusion^32^. Although co-infections were found in *G. m. morsitans* and *G. f. fuscipes* in some locations, the global correlation was missing. This is in agreement with the positive correlation found between *Sodalis* and *Trypanosoma* infection in *G. m. centralis* in Zambia, in which there was a 6.2 fold increase in the likelihood of a fly being infected with *Trypanosoma* if *Sodalis* was present^52^. More studies are needed to enhance the potential control interventions mediated by endosymbionts to reduce parasitic infections^61^.

The results of this study clearly indicate that the interaction between *Sodalis* and *Trypanosoma* infection is complex, species-specific and requires further investigation. The prevalence results indicate that *Sodalis* and *Trypanosoma* infections are not independent in some species, such as *G. p. gambiensis* and *G. medicorum* in west Africa and *G. pallidipes* in central and east Africa, In case of a positive correlation between *Sodalis* and *Trypanosoma* infection in these species, additional measures could be suggested when implementing the SIT to reduce the *Sodalis* density in the sterile males released in the targeted area to maximize the safe implementation of the SIT. These measures might include the mixing of *Sodalis* phage(s)^29,65^ with the blood meals to feed the mass-reared flies to reduce the *Sodalis* density in these flies. In addition, the blood meal offered to the males before release can be supplemented with one or more of the following antimicrobial products to reduce *Sodalis* density, i.e. streptozotocin^23^, indolicidin and OaBAC 5 mini^66^. The use of the *Sodalis* phage as well as these antimicrobial agents requires further studies to (1) develop methods to isolate the phage, (2) determine the conditions (e.g. suitable concentration) for its use, and (3) determine the impact on *Sodalis* density, tsetse productivity and survival. For *G. m morsitans* and *G. pallidipes*, our results suggest that *Sodalis* infection does not have an impact on *Trypanosoma* infection so here no additional measures need to be taken during the implementation of SIT against these species.

## Conclusion

*Sodalis* and *Trypanosoma* infection varied with tsetse taxon and location. There is a significant positive correlation between *Sodalis* and *Trypanosoma* infection in *G. medicorum*, *G. p. gambiensis* and *G. pallidipes*; however, no significant correlation was found in other tsetse taxa and locations. The results of this study will enable the decision makers of SIT projects to better plan and take the necessary measures to fine-tune and optimize SIT efficiency and safety.

## Methods

### Tsetse collection and DNA extractions

Tsetse flies were collected in 1995 and between 2005 and 2018 from 95 different geographical locations in fifteen countries in east, central, southern, and western Africa (Table 6, Supplementary Table 6). The tsetse flies were collected with species-specific traps which included the biconical trap^67^, the monoconical trap^68^, the Vavoua trap^69^, the Ngu trap^70,71^, the odour-baited Epsilon trap^72^, the NZI trap^73^, and the odour baited H trap^74^. A total of 6860 tsetse flies, belonging to ten tsetse species, were collected for this study (Table 6). The majority of the samples were collected in Burkina Faso (2274), Kenya (1008), Senegal (547) and South Africa (526). As the distribution of most tsetse species is allopatric (only few species are sympatric), not all tsetse species were collected from each country. Following collection, fly samples were preserved in 95% ethanol or propylene glycol and shipped to the FAO/IAEA Insect Pest Control Laboratory (IPCL) in Seibersdorf, Austria and stored at − 20 °C until analysis. Total DNA was extracted from individual whole fly bodies using the DNeasy tissue kit (QIAGEN Inc., Valencia, CA) following the supplier’s instructions. The DNA quality and concentration were measured by spectrophotometry (Synergy H1 Multi-Mode Reader, BioTek, Instruments, Inc., USA) and subsequently kept at 4 °C until screened for *Sodalis* and *Trypanosoma* infections*.* To verify the quality of the extracted DNA, a set of specific primers amplifying the *Glossina* spp. microsatellite GpCAG133 sequence (Supplementary Table 5) and only the successful samples were included in the analysis^21,75^**.**

**Table 6:** List of collections of tsetse adults with valid DNA screened for *Sodalis* and Trypanosome^a^ infection in wild tsetse population in east, central, southern and west Africa.

Country	No. of locations	No. of collection flies with valid DNA	Collection year
Ethiopia	1	459	2007
Kenya	11	1008	2007, 2008, 2009
Uganda	5	210	2007
Tanzania	5	338	2005, 2009
Democratic R. of Congo	1	35	1995
Zambia	1	210	2007
Zimbabwe	7	211	2006
South Africa	7	526	1995, 2018, 2019
Mozambique	1	100	2019
Eswatini	1	30	2018, 2019
Burkina Faso	14	2274	2008, 2010, 2013, 2015, 2018, 2019
Ghana^a^	11	234	2008
Guinea^a^	8	314	2008, 2009
Mali^a^	10	364	2008, 2010, 2011, 2012, 2013
Senegal	12	547	2008, 2009
Total	95	6860	

^a^Part of the trypanosome infection in west Africa was screened by Ouedraogo et al*.* 2018.

### *Trypanosoma* prevalence and genotyping

Polymerase chain reaction (PCR), following the method of Njiru et al.^76^ that used the primers ITS1-CF and ITS1-BR (Supplementary Table 5) previously designed to amplify the internal transcribed spacer (ITS1) of the ribosomal DNA, was used to detect *Trypanosoma* infection and *Trypanosoma* species in the fly samples. The PCR was carried out in 25 μl reaction mixtures containing 22.5 µl of 1.1 × Pre-Aliquoted PCR Master Mix (0.625 units Thermoprime Plus DNA Polymerase, 75 mM Tris–HCl (pH 8.8 at 25 °C), 20 mM (NH_4_)2SO_4_, 2.0 mM MgCl_2_, 0.01% (v/v) Tween-20 and 0.2 mM each of the dNTPs (ABgene, UK), 1 µl primers (at 200 nM final concentration of forward and reverse primer) and 1.5 µl of template DNA. PCR cycles were: 94 °C for 15 min, followed by 40 cycles of 94 °C for 30 s, 60 °C for 30 s, 72 °C for 30 s, and final extension 72 °C for 5 min. Interpretation of the results after resolving the amplification products in a 2% agarose gel (Fisher Biotech) stained with SafeGreen or ethidium bromide, was based on the characteristic band size of *Trypanosoma* taxa: all members of the subgenus *T. brucei* sspp (*T. b. brucei*, *T. b. gambiense*, *T. b. rhodesiense*: 480 bp); *T. congolense* savannah (700 bp); *T. congolense* Kilifi (620 bp); *T. congolense* forest (710 bp); *T. simiae* (400 bp); *T. simiae* Tsavo (370 bp); *T. godfreyi* (300 bp) and *T. vivax* (250 bp). The positive control DNA was from *T. congolense* savannah, *T. congolense* forest, *T. b. brucei*, *T. b. gambiense*, *T. b. rhodesiense*, *T. evansi*, and *T. vivax*. DNA samples validated with GpCAG133 primer amplification were screened for trypanosome infection. A tsetse sample was recorded as positive if one or more of the indicated band sizes was detected. *Trypanosoma* infection status and species were recorded for each fly.

### Prevalence of *Sodalis* infection

The detection of *Sodalis* in natural tsetse samples was based on the *Sodalis fliC* (flagellin) gene which results in an amplicon length of about 508 base pairs with the *Sodalis* specific primers Sod-fliC-F and Sod-fliC-R (Supplementary Table 5)^77^. These primers were used in single pairs or in multiplex PCR with GpCAG133 primers. For all PCR reactions, 22.5 µl of 1.1 × Pre-Aliquoted PCR Master Mix (ABgene, UK) was used. In a final volume of 25 µl, 1.5 µl of template DNA plus forward and reverse primers were added to a final concentration of 0.2 mM per primer in a volume of 1 µl. Samples were considered *Sodalis*-infected if the expected symbiont PCR product amplicon was detected. Data were accepted only if the control gene GpCAG133 sequence was amplified. The PCR cycling conditions were: 95 °C for 5 min followed by 34 cycles of 95 °C for 30 s, 52.5 °C for 30 s, 72 °C for 30 s and lastly at 72 °C for 10 min; PCR products were separated by agarose (2%) gel electrophoresis and SafeGreen or ethidium bromide staining.

### Analysis of the *Trypanosoma* and *Sodalis* infection in wild tsetse populations

#### Co-infection of tsetse adults with *Sodalis* and Trypanosoma infection

The co-infection of *Sodalis* and *Trypanosoma* infection was evaluated based on the PCR prevalence. The infection status was divided into four categories *Sodalis* positive and *Trypanosoma* positive (S^+^/T^+^), *Sodalis* positive and *Trypanosoma* negative (S^+^/T^−^), *Sodalis* negative and *Trypanosoma* positive (S^−^/T^+^) and *Sodalis* negative and *Trypanosoma* negative (S^−^/T^−^).

#### Analysis of the *Trypanosoma *and *Sodalis* density

Samples showing *Trypanosoma* infection (not *T. vivax*) with *Sodalis* (S^+^/T^+^) and samples not infected with *Trypanosoma* but infected with *Sodalis* (S^+^/T^−^) were evaluated with quantitative PCR (qPCR) to assess the impact of *Trypanosoma* infection (regardless the *Trypanosoma* type) on *Sodalis* density. The qPCR was performed using a CFX96 Real Time PCR Detection System (Bio-Rad). The *fliC* gene was amplified with the following primers: sodqPCR-FliCF and sodqPCR-FliCR^78^ (Supplementary Table 5) to assess the density of the symbiont present within *Trypanosoma* infected and noninfected, additional criteria for the selection of the samples was the presence of the two groups (S^+^/T^+^) and (S^+^/T^−^) in a given population. Based on the preceding criteria 96 individual flies (52 and 44 flies with infection status of (S^+^/T^+^) and (S^+^/T^−^), respectively, were selected from the *G. pallidipes* and *G. m. morsitans* collected in Kenya, Tanzania and Zimbabwe*.* In addition, samples with (S^+^/T^+^) and (S^−^/T^+^) were used to assess the impact of *Sodalis* infection on *Trypanosoma* density. *Trypanosomatidae*18S specific primers (18S_Typ_F and18S_Typ_R) (Supplementary Table 5) were used to assess the *Trypanosoma* density in the tested samples. The DNA from all selected samples was diluted to a final concentration of 4 ng/μl and 5 μl of the diluted DNA was used for qPCR to determine *Sodalis* and *Trypanosoma* DNA density normalized to the housekeeping β-tubulin gene. The amplification mixture contained 5 μl of DNA template, 200 nM of each primer, and 7.5 μl iQ™ SYBER Green Supermix (Bio-Rad). qPCR cycling conditions for *Sodalis* were as follows: initial denaturation at 95 °C for 2 min; 39 cycles of 95 °C for 5 s, 55 °C for 30 s, one step at 95 °C for 5 s and a melting curve constructed from 65 °C to 95 °C in increments of 0.5 °C for 5 s. The same conditions were used for *Trypanosoma* except the annealing temperature was at 60 °C. The analysis of the *Sodalis, Trypanosoma* and Tubulin densities was based only on qPCR data with the expected melting curve at 81.5 °C, 85.5 °C and 86 °C, respectively.

### Data analysis

The prevalence data were recorded and analyzed with the general linear model (GLM)^79^. The prevalence of *Sodalis*, *Trypanosoma* species and each *Trypanosoma* species and co-infection were tested for differences between the tsetse taxa and between countries. For each country, the prevalence was assessed again for differences between the localities where the flies were collected and between the tsetse species present in each country. In the absence of PCR detected *Sodalis* or *Trypanosoma* infection, the upper 95% confidence interval for the true rate of infection was calculated following the method of Couey and Chew^62^. *Trypanosoma* prevalence between taxa was compared between species by a pairwise comparison of proportions with a Bonferroni correction and Benjamini–Hochberg correction. The analyses were executed in R v 4.0.5^79^ using RStudio V 1.4.1106^80,81^ with the packages ggplot2 v3.3.2.1^82^, lattice v0.20-41^83^, car^84^, ggthems^85^ and MASS v7.3-51.6^86^ except for the Chi squared tests for independence, Spearman correlation coefficient and Cochran–Mantel–Haenszel test for repeated tests of independence, which were performed using Excel 2013 The R Markdown file is available in Supplementary File 1.

To analyse the qPCR data, normalized density of *Trypanosoma* and *Sodalis* against the house keeping gene (tubulin) was extracted from the CFX Maestro software. Samples giving a valid density (not N/A) for both *Trypanosoma* and *Sodalis* were retained for further statistical analysis in R. Similarities in the structure of *Sodalis* and *Trypanosoma* (single and multiple) infection and the role of different factors such as countries and tsetse taxa, were assessed using the matrix display and metric multidimensional scaling (mMDS) plot with bootstrap averages in PRIMER version 7+. The bootstrap averages plots were displayed with a Bray and Curtis matrix based on the square-root transformation of the *Sodalis* and *Trypanosoma* (single and multiple) infection abundance data^87^. The tests were based on the multivariate null hypothesis via the use of the non-parametric statistical method PERMANOVA^88^. The Permanova test was conducted on the average of the abundance data based on the country-species after excluding the data of Eswatini (low number of tested samples).

## Supplementary Information

Supplementary Information 1.

Supplementary Information 2.

Supplementary Information 3.

Supplementary Information 4.

Supplementary Information 5.

Supplementary Information 6.

Supplementary Information 7.

## Data Availability

Materials described in the paper, including all relevant raw data, are available in this link https://dataverse.harvard.edu/dataset.xhtml?persistentId=doi:10.7910/DVN/WOTAIY).

## Abbreviations

SIT: Sterile insect techniques
qPCR: Quantitative polymerase chain reaction
BKF: Burkina Faso
ETH: Ethiopia
GHA: Ghana
GUI: Guinea
KEN: Kenya
MLI: Mali
MOZ: Mozambique
SAF: South Africa
SWA: Eswatini
ZAI: Democratic Republic of the Congo
ZAM: Zambia
ZIM: Zimbabwe
Ga: *Glossina austeni*
Gb: *G. brevipalpis*
Gff: *G. fuscipes fuscipes*
Gmm: *G. morsitans morsitans*
Gmsm: *G. m. submorsitans*
Gpg: *G. palpalis gambiensis*
Gpp: *G. p. palpalis*
Tc: *Trypanosoma congolense*
Tv: *Trypanosoma vivax*
Tz: *T. brucei* sspp.

## References

[1] Elsen P, Amoudi MA, Leclercq M (1990). First record of *Glossina fuscipes fuscipes* Newstead, 1910 and *Glossina morsitans submorsitans* Newstead, 1910 in southwestern Saudi Arabia. Ann. Soc. Belg. Med. Trop..

[2] Leak, S. G. A. *Tsetse Biology and Ecology: Their Role in the Epidemiology and Control of Trypanosomosis*. (ILRI (aka ILCA and ILRAD), 1999).

[3] Mattioli RC (2004). Tsetse and trypanosomiasis intervention policies supporting sustainable animal-agricultural development. J. Food Agric. Environ..

[4] Cecchi G, Mattioli RC, Slingenbergh J, Rocque SDL (2008). Land cover and tsetse fly distributions in sub-Saharan Africa. Med. Vet. Entomol..

[5] Aksoy, S., Gibson, W. C. & Lehane, M. J. Interactions between tsetse and trypanosomes with implications for the control of trypanosomiasis. In *Advances in Parasitology* vol. 53 1–83 (Academic Press, 2003). 10.1016/s0065-308x(03)53002-0 14587696

[6] Roditi I, Lehane MJ (2008). Interactions between trypanosomes and tsetse flies. Curr. Opin. Microbiol..

[7] Geerts S, Holmes PH (1998). Drug management and parasite resistance in bovine trypanosomiasis in Africa.

[8] Reinhardt, E. Travailler Ensemble: La Mouche Tse´ -tse´ et La Pauvrete Rurale. Accessed Nov 2021. https://www.un.org/french/pubs/chronique/2002/numero2/0202p17_la_mouche_tsetse.html. (2002).

[9] Geiger A (2011). Transcriptomics and proteomics in human African trypanosomiasis: Current status and perspectives. J. Proteom..

[10] Aksoy S, Rio RVM (2005). Interactions among multiple genomes: Tsetse, its symbionts and trypanosomes. Insect Biochem. Mol. Biol..

[11] Delespaux V, Geysen D, Van den Bossche P, Geerts S (2008). Molecular tools for the rapid detection of drug resistance in animal trypanosomes. Trends Parasitol..

[12] Vreysen MJB (2001). Principles of area-wide integrated tsetse fly control using the sterile insect technique. Méd. Trop..

[13] Schofield CJ, Kabayo JP (2008). Trypanosomiasis vector control in Africa and Latin America. Parasit. Vectors.

[14] Molyneux DH, Hopkins DR, Zagaria N (2004). Disease eradication, elimination and control: The need for accurate and consistent usage. Trends Parasitol..

[15] Abbeele JVD, Claes Y, Bockstaele DV, Ray DL, Coosemans M (1999). *Trypanosoma brucei* spp. development in the tsetse fly: Characterization of the post-mesocyclic stages in the foregut and proboscis. Parasitology.

[16] Maudlin I, Welburn SC (1994). Maturation of trypanosome infections in tsetse. Exp. Parasitol..

[17] Moloo, S. K., Asonganyi, T. & Jenni, L. Cyclical development of *Trypanosoma brucei gambiense* from cattle and goats in Glossina. *Cycl. Dev. Trypanos. Brucei Gamb. Cattle Goats Glossina* **43**, 407–408 (1986). 2882668

[18] MacLeod ET, Darby AC, Maudlin I, Welburn SC (2007). Factors affecting trypanosome maturation in tsetse flies. PLoS One.

[19] Weiss BL, Wang J, Maltz MA, Wu Y, Aksoy S (2013). Trypanosome infection establishment in the tsetse fly gut is influenced by microbiome-regulated host immune barriers. PLoS Pathog..

[20] O’Neill SL, Gooding RH, Aksoy S (1993). Phylogenetically distant symbiotic microorganisms reside in Glossina midgut and ovary tissues. Med. Vet. Entomol..

[21] Cheng Q, Aksoy S (1999). Tissue tropism, transmission and expression of foreign genes in vivo in midgut symbionts of tsetse flies. Insect Mol. Biol..

[22] Wang J, Weiss BL, Aksoy S (2013). Tsetse fly microbiota: Form and function. Front. Cell. Infect. Microbiol..

[23] Dale C, Welburn SC (2001). The endosymbionts of tsetse flies: Manipulating host–parasite interactions. Int. J. Parasitol..

[24] Trappeniers K, Matetovici I, Van Den Abbeele J, De Vooght L (2019). The tsetse fly displays an attenuated immune response to its secondary symbiont, *Sodalis glossinidius*. Front. Microbiol..

[25] Medlock J, Atkins KE, Thomas DN, Aksoy S, Galvani AP (2013). Evaluating paratransgenesis as a potential control strategy for African trypanosomiasis. PLoS Negl. Trop. Dis..

[26] Geiger A, Ponton F, Simo G (2015). Adult blood-feeding tsetse flies, trypanosomes, microbiota and the fluctuating environment in sub-Saharan Africa. ISME J..

[27] Maudlin I, Ellis DS (1985). Association between intracellular rickettsial-like infections of midgut cells and susceptibility to trypanosome infection in *Glossina* spp. Z. Für Parasitenkd..

[28] Geiger A (2007). Vector competence of Glossina palpalis gambiensis for *Trypanosoma brucei* s.l. and genetic diversity of the symbiont *Sodalis glossinidius*. Mol. Biol. Evol..

[29] Hamidou Soumana I (2014). The transcriptional signatures of *Sodalis glossinidius*. in the *Glossina palpalis gambiensis* flies negative for *Trypanosoma brucei gambiense* contrast with those of this symbiont in tsetse flies positive for the parasite: Possible involvement of a *Sodalis*-hosted prophage in fly *Trypanosoma* refractoriness?. Infect. Genet. Evol..

[30] Farikou O (2010). Tripartite interactions between tsetse flies, *Sodalis glossinidius* and trypanosomes—An epidemiological approach in two historical human African trypanosomiasis foci in Cameroon. Infect. Genet. Evol..

[31] Makhulu EE (2020). Tsetse blood-meal sources, endosymbionts, and trypanosome infections provide insight into African trypanosomiasis transmission in the Maasai Mara National Reserve, a wildlife-human-livestock interface. bioRxiv.

[32] Wamwiri FN (2013). *Wolbachia*, *Sodalis* and trypanosome co-infections in natural populations of *Glossina austeni* and *Glossina pallidipes*. Parasit. Vectors.

[33] Channumsin M, Ciosi M, Masiga D, Turner CMR, Mable BK (2018). *Sodalis glossinidius* presence in wild tsetse is only associated with presence of trypanosomes in complex interactions with other tsetse-specific factors. BMC Microbiol..

[34] Kanté Tagueu S, Farikou O, Njiokou F, Simo G (2018). Prevalence of *Sodalis glossinidius* and different trypanosome species in *Glossina palpalis palpalis* caught in the Fontem sleeping sickness focus of the southern Cameroon. Parasite.

[35] Kame-Ngasse GI (2018). Prevalence of symbionts and trypanosome infections in tsetse flies of two villages of the “Faro and Déo” division of the Adamawa region of Cameroon. BMC Microbiol..

[36] Dennis JW (2014). *Sodalis glossinidius* prevalence and trypanosome presence in tsetse from Luambe National Park, Zambia. Parasit. Vectors.

[37] Vreysen MJB, Saleh KM, Zhu Z-R, Suleiman FW (2000). Responses of *Glossina austeni* to sticky panels and odours. Med. Vet. Entomol..

[38] Vreysen MJB, Hendrichs J, Pereira R, Vreysen MJB (2021). Area-wide integrated pest management of a *Glossina palpalis gambiensis* population from the Niayes area of Senegal: A review of operational research in support of an operational phased conditional approach. in. Area-Wide Integrated Pest Management: Development and Field Application.

[39] Van Den Abbeele J (2013). Enhancing tsetse fly refractoriness to trypanosome infection—A new IAEA coordinated research project. J. Invertebr. Pathol..

[40] van den Bossche P (2006). Effect of isometamidium chloride treatment on susceptibility of tsetse flies (Diptera: Glossinidae) to trypanosome infections. J. Med. Entomol..

[41] Bouyer J (2008). Does isometamidium chloride treatment protect tsetse flies from trypanosome infections during SIT campaigns?. Med. Vet. Entomol..

[42] De Vooght L, Caljon G, De Ridder K, van den Abbeele J (2014). Delivery of a functional anti-trypanosome Nanobody in different tsetse fly tissues via a bacterial symbiont, *Sodalis glossinidius*. Microb. Cell Factories.

[43] Caljon G, De Vooght L, Van Den Abbeele J (2013). Options for the delivery of anti-pathogen molecules in arthropod vectors. J. Invertebr. Pathol..

[44] Demirbas-Uzel G (2018). Combining paratransgenesis with SIT: Impact of ionizing radiation on the DNA copy number of *Sodalis glossinidius* in tsetse flies. BMC Microbiol..

[45] Aksoy E (2014). Analysis of multiple tsetse fly populations in Uganda reveals limited diversity and species-specific gut microbiota. Appl. Environ. Microbiol..

[46] Soumana IH (2013). The bacterial flora of tsetse fly midgut and its effect on trypanosome transmission. J. Invertebr. Pathol..

[47] Maudlin I, Welburn SC, Mehlitz D (1990). The relationship between rickettsia-like-organisms and trypanosome infections in natural populations of tsetse in Liberia. Trop. Med. Parasitol..

[48] Doudoumis V (2013). Tsetse-*Wolbachia* symbiosis: Comes of age and has great potential for pest and disease control. J. Invertebr. Pathol..

[49] Baker RD, Maudlin I, Milligan PJM, Molyneux DH, Welburn SC (1990). The possible role of Rickettsia-like organisms in trypanosomiasis epidemiology. Parasitology.

[50] Soumana IH (2013). Population dynamics of *Glossina palpalis gambiensis* symbionts, *Sodalis glossinidius*, and *Wigglesworthia glossinidia*, throughout host-fly development. Infect. Genet. Evol..

[51] Alam U (2011). *Wolbachia* symbiont infections induce strong cytoplasmic incompatibility in the tsetse fly *Glossina morsitans*. PLoS Pathog..

[52] Mbewe NJ, Mweempwa C, Guya S, Wamwiri FN (2015). Microbiome frequency and their association with trypanosome infection in male *Glossina morsitans centralis* of Western Zambia. Vet. Parasitol..

[53] Musaya J (2017). Polymerase chain reaction identification of *Trypanosoma brucei* rhodesiense in wild tsetse flies from Nkhotakota Wildlife Reserve, Malawi. Malawi Med. J. J. Med. Assoc. Malawi.

[54] Malele II (2011). Multiple Trypanosoma infections are common amongst Glossina species in the new farming areas of Rufiji district, Tanzania. Parasit. Vectors.

[55] Ngonyoka A (2017). Patterns of tsetse abundance and trypanosome infection rates among habitats of surveyed villages in Maasai steppe of northern Tanzania. Infect. Dis. Poverty.

[56] Lefrançois T (1999). Polymerase chain reaction characterization of trypanosomes in *Glossina morsitans submorsitans* and *G. tachinoides* collected on the game ranch of Nazinga, Burkina Faso. Acta Trop..

[57] Djohan V (2015). Detection and identification of pathogenic trypanosome species in tsetse flies along the Comoé River in Côte d’Ivoire. Parasite Paris Fr..

[58] Nayupe SF (2019). The use of molecular technology to investigate trypanosome infections in tsetse flies at Liwonde Wild Life Reserve. Malawi Med. J. J. Med. Assoc. Malawi.

[59] Karshima SN, Ajogi I, Mohammed G (2016). Eco-epidemiology of porcine trypanosomosis in Karim Lamido, Nigeria: Prevalence, seasonal distribution, tsetse density and infection rates. Parasit. Vectors.

[60] Simo G (2019). Molecular identification of *Wolbachia* and *Sodalis glossinidius* in the midgut of *Glossina fuscipes quanzensis* from the Democratic Republic of Congo. Parasite Paris Fr..

[61] Odeniran PO, Macleod ET, Ademola IO, Welburn SC (2019). Endosymbionts interaction with trypanosomes in Palpalis group of Glossina captured in southwest Nigeria. Parasitol. Int..

[62] Couey HM, Chew V (1986). Confidence limits and sample size in quarantine research. J. Econ. Entomol..

[63] Geiger A, Ravel S, Frutos R, Cuny G (2005). *Sodalis glossinidius* (Enterobacteriaceae) and vectorial competence of *Glossina palpalis gambiensis* and *Glossina morsitans morsitans* for *Trypanosoma congolense* savannah type. Curr. Microbiol..

[64] Geiger A, Cuny G, Frutos R (2005). Two tsetse fly species, *Glossina palpalis gambiensis* and *Glossina morsitans morsitans*, carry genetically distinct populations of the secondary symbiont *Sodalis glossinidius*. Appl. Environ. Microbiol..

[65] Geiger A (2015). Differential expression of midgut proteins in *Trypanosoma brucei gambiense*-stimulated versus non-stimulated *Glossina palpalis gambiensis* flies. Front. Microbiol..

[66] Haines LR, Hancock REW, Pearson TW (2003). Cationic antimicrobial peptide killing of African trypanosomes and *Sodalis glossinidius*, a bacterial symbiont of the insect vector of sleeping sickness. Vector-Borne Zoonotic Dis..

[67] Challier A, Laveissière C (1973). Un nouveau piege pour la capture des glossines (Glossina: Diptera, Muscidae): Description et essais sur le terrain. Cah. ORSTOM Sér. Entomol. Médicale Parasitol..

[68] Lancien J (1981). Description du piege monoconique utilise pour l’elimination des glossines en Republique Populaire du Congo. Cah. ORSTOM Sér. Entomol. Médicale Parasitol..

[69] Laveissière, C. & Grébaut, P. The trapping of tsetse flies (Diptera: Glossinidae). Improvement of a model: The Vavoua trap. *Trop. Med. Parasitol. Off. Organ Dtsch. Tropenmedizinische Ges. Dtsch. Ges. Tech. Zusammenarbeit GTZ* **41**, 185–192 (1990). 2166330

[70] Brightwell B (1987). A new trap to *Glossina pallidipes*. Trop. Pest Manag..

[71] Brightwell R, Dransfield RD, Kyorku C (1991). Development of a low-cost tsetse trap and odour baits for *Glossina pallidipes* and *G. longipennis* in Kenya. Med. Vet. Entomol..

[72] Hargrove JE, Langley PA (1990). Sterilizing tsetse (Diptera: Glossinidae) in the field: A successful trial. Bull. Entomol. Res..

[73] Mihok S (2002). The development of a multipurpose trap (the Nzi) for tsetse and other biting flies. Bull. Entomol. Res..

[74] Kappmeier K (2000). A newly developed odour-baited ‘H trap’ for the live collection of *Glossina brevipalpis* and *Glossina austeni* (Diptera: Glossinidae) in South Africa. Onderstepoort J. Vet. Res..

[75] Baker MD, Krafsur ES (2001). Identification and properties of microsatellite markers in tsetse flies *Glossina morsitans* sensu lato (Diptera: Glossinidae). Mol. Ecol. Notes.

[76] Njiru ZK (2005). The use of ITS1 rDNA PCR in detecting pathogenic African trypanosomes. Parasitol. Res..

[77] Toh H (2006). Massive genome erosion and functional adaptations provide insights into the symbiotic lifestyle of *Sodalis glossinidius* in the tsetse host. Genome Res..

[78] Weiss BL, Maltz M, Aksoy S (2012). Obligate symbionts activate immune system development in the tsetse fly. J. Immunol..

[79] R Core Team. *R: A Language and Environment for Statistical Computing*. (R Foundation for Statistical Computing, 2021).

[80] Baier T, Neuwirth E (2007). Excel: COM: R. Comput. Stat..

[81] RStudio Team. *RStudio: Integrated Development Environment for R*. (RStudio, Inc., 2016).

[82] Wickham H (2016). ggplot2: Elegant Graphics for Data Analysis.

[83] Sarkar, D. *Lattice: Multivariate Data Visualization with R*. (Springer Science & Business Media, 2008).

[84] Fox, J. & Weisberg, S. *An R Companion to Applied Regression*, 2nd ed. (Sage, 2019).

[85] Jeffrey B. Arnold. ggthemes: Extra Themes, Scales and Geoms for ‘ggplot2’. R package version 4.2.4. (2021).

[86] Venables, W. N. & Ripley, B. D. *Modern Applied Statistics with S*. (Springer, 2002).

[87] Clarke, K. R. & Gorley, R. N. Getting started with PRIMER v7. Accessed Nov 2021. http://updates.primer-e.com/primer7/manuals/Getting_started_with_PRIMER_7.pdf. (2016).

[88] Anderson MJ (2001). A new method for non-parametric multivariate analysis of variance. Austral Ecol..

