# Supplementary material for: Prevalence of Trypanosoma and Sodalis in wild populations of tsetse flies and their impact on sterile insect technique programmes for tsetse eradication
Source: Sci Rep. 2022 Feb 28;12:3322. doi: 10.1038/s41598-022-06699-2 (PMC8885713; doi:10.1038/s41598-022-06699-2)
Supplement: Supplementary file 1 — Supplementary Information 1. [file 41598_2022_6699_MOESM1_ESM.docx]

Prevalence of *Trypanosoma* and *Sodalis* in wild populations of tsetse flies and their Impact on Sterile Insect Technique programmes for tsetse eradication

Mouhamadou M. Dieng, Kiswend-sida M. Dera, Percy Moyaba, Gisele M. S. Ouedraogo2, Guler Demirbas-Uzel, Fabian Gstöttenmayer, Fernando C. Mulandane, Luis Neves, Sihle Mdluli, Jean-Baptiste Rayaisse, Adrien M. G. Belem, Soumaïla Pagabeleguem, Chantel J. de Beer, Andrew G. Parker, Jan Van Den Abbeele, Robert L. Mach, Marc J. B. Vreysen, and Adly M. M. Abd-Alla

03/06/2021

library(ggplot2)

## Warning: package 'ggplot2' was built under R version 4.0.5

library(lattice)
library(gcookbook)
library(ggfortify)
library(datasets)
library(MASS)
library(survival)
library(rmarkdown)
library(knitr)
library(coxme)

## Loading required package: bdsmatrix

##
## Attaching package: 'bdsmatrix'

## The following object is masked from 'package:base':
##
## backsolve

library(lme4)

## Loading required package: Matrix

library(nlme)

##
## Attaching package: 'nlme'

## The following object is masked from 'package:lme4':
##
## lmList

library(tidyverse)

## Warning: package 'tidyverse' was built under R version 4.0.5

## -- Attaching packages --------------------------------------- tidyverse 1.3.1 --

## v tibble 3.1.1 v dplyr 1.0.5
## v tidyr 1.1.3 v stringr 1.4.0
## v readr 1.4.0 v forcats 0.5.1
## v purrr 0.3.4

## Warning: package 'tibble' was built under R version 4.0.5

## Warning: package 'dplyr' was built under R version 4.0.5

## -- Conflicts ------------------------------------------ tidyverse_conflicts() --
## x dplyr::collapse() masks nlme::collapse()
## x tidyr::expand() masks Matrix::expand()
## x dplyr::filter() masks stats::filter()
## x dplyr::lag() masks stats::lag()
## x tidyr::pack() masks Matrix::pack()
## x dplyr::select() masks MASS::select()
## x tidyr::unpack() masks Matrix::unpack()

library(gapminder)
library(rcompanion)
library(FSA)

## ## FSA v0.8.32. See citation('FSA') if used in publication.
## ## Run fishR() for related website and fishR('IFAR') for related book.

library(stats)
library(RCA)

## Loading required package: igraph

##
## Attaching package: 'igraph'

## The following objects are masked from 'package:dplyr':
##
## as_data_frame, groups, union

## The following objects are masked from 'package:purrr':
##
## compose, simplify

## The following object is masked from 'package:tidyr':
##
## crossing

## The following object is masked from 'package:tibble':
##
## as_data_frame

## The following objects are masked from 'package:stats':
##
## decompose, spectrum

## The following object is masked from 'package:base':
##
## union

## Loading required package: gplots

## Registered S3 method overwritten by 'gplots':
## method from
## reorder.factor DescTools

##
## Attaching package: 'gplots'

## The following object is masked from 'package:stats':
##
## lowess

library(broom)

## Warning: package 'broom' was built under R version 4.0.5

library(sp)
library(MuMIn)

## Registered S3 methods overwritten by 'MuMIn':
## method from
## formula.coxme coxme
## logLik.coxme coxme
## logLik.lmekin coxme

library(ggpubr)

## Registered S3 methods overwritten by 'car':
## method from
## influence.merMod lme4
## cooks.distance.influence.merMod lme4
## dfbeta.influence.merMod lme4
## dfbetas.influence.merMod lme4

library(AICcmodavg)

##
## Attaching package: 'AICcmodavg'

## The following objects are masked from 'package:MuMIn':
##
## AICc, DIC, importance

## The following object is masked from 'package:lme4':
##
## checkConv

library(car)

## Warning: package 'car' was built under R version 4.0.5

## Loading required package: carData

##
## Attaching package: 'car'

## The following object is masked from 'package:FSA':
##
## bootCase

## The following object is masked from 'package:dplyr':
##
## recode

## The following object is masked from 'package:purrr':
##
## some

library(ggthemes)

## Warning: package 'ggthemes' was built under R version 4.0.5

## prepare the data

data=read.csv("rawdata_statistic_sod_tryp_bio2.csv")
str(data)

## 'data.frame': 157 obs. of 18 variables:
## $ Country : chr "ETH" "BKF" "BKF" "SEN" ...
## $ Localisation: chr "Arba minch, nech SAFr" "Comoe" "Comoe" "Pout" ...
## $ Species : chr "Gp" "Gt" "Gt" "Gpg" ...
## $ Sex : chr "NI" "F" "M" "NI" ...
## $ Sample : int 1 2 3 4 5 6 7 8 9 10 ...
## $ Prev_Sod : num 20.5 0 0 0 42.6 ...
## $ Prev_Tspp : num 20.04 16.54 15 7.04 88.72 ...
## $ Prev_Tc : num 6.1 1.1 3.64 1.51 21.54 ...
## $ Prev_Tv : num 0.65 13.24 11.36 2.01 15.38 ...
## $ Prev_Tz : num 1.09 0.37 0 0.5 0.51 1.03 0 0.68 0.68 0 ...
## $ Prev_Tsg : num 10.46 0 0 3.02 26.15 ...
## $ Prev_TcTv : num 0 0.74 0 0 6.67 1.03 0 0 0 0 ...
## $ Prev_TcTz : num 0 1.1 0 0 1.54 0.51 0 0 0 0.76 ...
## $ Prev_TcTsg : num 0.65 0 0 0 15.9 0.51 0 7.53 0.68 0 ...
## $ Prev_TvTz : num 0 0 0 0 1.03 0 0 0 0 0 ...
## $ Prev_TvTsg : num 0.44 0 0 0 0 0 0 0 0.68 0 ...
## $ Prev_TzTsg : num 0.65 0 0 0 0 1.03 0 0 0.68 0 ...
## $ Prev_TcTvTz : num 0 0 0 0 0 0 0 0 0 0 ...

attach(data)
head(data)

## Country Localisation Species Sex Sample Prev_Sod Prev_Tspp Prev_Tc
## 1 ETH Arba minch, nech SAFr Gp NI 1 20.48 20.04 6.10
## 2 BKF Comoe Gt F 2 0.00 16.54 1.10
## 3 BKF Comoe Gt M 3 0.00 15.00 3.64
## 4 SEN Pout Gpg NI 4 0.00 7.04 1.51
## 5 KEN Katotoi Gp NI 5 42.56 88.72 21.54
## 6 KEN Mwea Gp NI 6 3.08 19.49 7.18
## Prev_Tv Prev_Tz Prev_Tsg Prev_TcTv Prev_TcTz Prev_TcTsg Prev_TvTz Prev_TvTsg
## 1 0.65 1.09 10.46 0.00 0.00 0.65 0.00 0.44
## 2 13.24 0.37 0.00 0.74 1.10 0.00 0.00 0.00
## 3 11.36 0.00 0.00 0.00 0.00 0.00 0.00 0.00
## 4 2.01 0.50 3.02 0.00 0.00 0.00 0.00 0.00
## 5 15.38 0.51 26.15 6.67 1.54 15.90 1.03 0.00
## 6 0.51 1.03 7.69 1.03 0.51 0.51 0.00 0.00
## Prev_TzTsg Prev_TcTvTz
## 1 0.65 0
## 2 0.00 0
## 3 0.00 0
## 4 0.00 0
## 5 0.00 0
## 6 1.03 0

data=na.omit(data)
data

## Country Localisation Species Sex Sample Prev_Sod Prev_Tspp
## 1 ETH Arba minch, nech SAFr Gp NI 1 20.48 20.04
## 2 BKF Comoe Gt F 2 0.00 16.54
## 3 BKF Comoe Gt M 3 0.00 15.00
## 4 SEN Pout Gpg NI 4 0.00 7.04
## 5 KEN Katotoi Gp NI 5 42.56 88.72
## 6 KEN Mwea Gp NI 6 3.08 19.49
## 7 SAF Phinda Gb NI 7 4.12 0.00
## 8 ZAM Mfuwe Gp NI 8 2.05 45.21
## 9 URT URTzania Gp NI 9 78.77 19.86
## 10 BKF Comoe Gmsm F 10 0.00 9.16
## 11 BKF Folonzo Gt F 11 0.00 18.87
## 12 BKF Folonzo Gpg F 12 0.00 7.55
## 13 BKF Folonzo Gpg M 13 1.89 16.04
## 14 BKF Folonzo Gt M 14 0.00 32.63
## 15 GUI Kangoliya Gpg F 15 95.74 0.00
## 16 UGA Buvuma island Gff NI 16 4.26 10.64
## 17 KEN Kari Gp NI 17 89.36 2.13
## 18 SEN Kayar Gpg NI 18 0.00 1.14
## 19 KEN Koibos Gp NI 19 0.00 71.59
## 20 KEN Meru nat. parc Gp NI 20 22.99 70.11
## 21 KEN Kari Gmm NI 21 63.53 2.35
## 22 URT MaSAFng-URTga Gmm NI 22 76.54 53.09
## 23 KEN Ruma nat. parc Gp NI 23 26.25 17.50
## 24 GHA Walewale Gt M 24 0.00 53.85
## 25 BKF Moussodougou Gpg F 25 0.00 44.87
## 26 ZIM Makuti Gmm NI 26 26.92 91.03
## 27 SAF False Bay Park Ga NI 27 0.00 2.60
## 28 BKF Comoe Gmsm M 28 0.00 10.67
## 29 BKF Folonzo Gmsm M 29 0.00 40.00
## 30 BKF Comoe Gpg F 30 0.00 2.82
## 31 URT MaSAFng-URTga Gp NI 31 66.20 73.24
## 32 BKF Kartasso Gpg F 32 0.00 0.00
## 33 BKF Kartasso Gpg M 33 0.00 0.00
## 34 SEN DiackSAFo Peulh Gpg NI 34 0.00 7.69
## 35 BKF Moussodougou Gpg M 35 0.00 21.88
## 37 BKF Comoe Gmed M 37 6.56 24.59
## 38 KEN Emsos Gp NI 38 0.00 93.33
## 39 BKF Folonzo Gmsm F 39 1.69 20.34
## 40 MLI Bani Gpg F 40 0.00 1.72
## 41 SAF SAFint Lucia Ga NI 41 0.00 38.60
## 42 UGA Budaka Gp NI 42 94.55 7.27
## 43 GHA Walewale Gt F 43 0.00 66.04
## 44 BKF Arly Gt F 44 0.00 1.89
## 45 BKF Kampty Gpg F 45 0.00 90.57
## 46 SAF Lower Mkhuze Ga NI 46 0.00 43.40
## 47 BKF Comoe Gpg M 47 0.00 1.92
## 48 MLI SEN Gpg M 48 0.00 7.69
## 49 KEN Ikapolok Gff NI 49 39.22 37.25
## 50 SAF Hluhluwe Gb NI 50 0.00 32.00
## 51 SAF North eastern KwaZulu-Natal Gb NI 51 0.00 4.00
## 52 MOZ Reserva Especial de Maputo Gb NI 52 14.00 6.00
## 53 MOZ Reserva Especial de Maputo Ga NI 53 0.00 10.00
## 54 BKF Bama Gpg F 54 0.00 0.00
## 55 BKF Arly Gt M 55 0.00 0.00
## 56 SEN Tambacounda Gpg F 56 0.00 41.46
## 57 SEN Tambacounda Gpg M 57 0.00 71.79
## 58 SEN SebikoURTe Gpg NI 58 0.00 5.13
## 59 SAF North eastern KwaZulu-Natal Ga NI 59 5.13 5.13
## 60 ZIM Makuti Gp NI 60 15.79 86.84
## 61 KEN Obekai Gff NI 61 0.00 5.26
## 62 MLI SEN Gpg F 62 0.00 0.00
## 63 ZAI ZAI Gpp NI 63 11.43 2.86
## 64 BKF Bama Gpg M 64 0.00 0.00
## 65 MLI Banco Gpg F 65 0.00 20.59
## 66 BKF Dedougou Gpg F 66 0.00 52.94
## 67 MLI Sikasso Gpg F 67 0.00 6.06
## 68 BKF Comoe Gmed F 68 12.12 51.52
## 69 GHA MorURTi Gt M 69 0.00 50.00
## 70 BKF Sissili Gt M 70 0.00 25.00
## 71 GUI Kangoliya Gpg M 71 0.00 0.00
## 72 BKF Folonzo Gmed F 72 0.00 50.00
## 73 BKF Folonzo Gmed M 73 0.00 46.67
## 74 SAF SAFint Lucia Gb NI 74 0.00 43.33
## 75 SWA Mlawula Nature Reserve Ga NI 75 0.00 0.00
## 76 URT Uguja island Ga NI 76 6.67 10.00
## 77 GUI Mini Gpg F 77 0.00 3.45
## 78 SEN Hann Gpg NI 78 0.00 0.00
## 79 UGA Omugo Gp NI 79 100.00 0.00
## 80 BKF Kampty Gpg M 80 0.00 80.77
## 81 ZIM Mushumb Gp NI 81 3.85 19.23
## 82 GUI Kifala Gpg M 82 0.00 0.00
## 83 MLI Sikasso Gpg M 83 0.00 0.00
## 84 MLI SS Gpg F 84 0.00 4.00
## 85 UGA Lira Gp NI 85 0.00 16.00
## 86 BKF Kenedougou Gpg F 86 0.00 0.00
## 87 MLI SS Gpg M 87 0.00 0.00
## 88 BKF Dedougou Gpg M 88 0.00 69.57
## 89 GUI Bafing Gpg F 89 0.00 5.00
## 90 KEN Kiria Gp NI 90 0.00 80.00
## 91 BKF Kampty Gpg NI 91 0.00 84.21
## 92 GUI Tinkisso Gpg M 92 0.00 5.56
## 93 ZIM Kemukura Gmm NI 93 22.22 5.56
## 94 BKF Kenedougou Gpg M 94 0.00 0.00
## 95 GHA Bougouhiya Gt F 95 0.00 18.75
## 96 GUI Dekonkore Gpg F 96 0.00 6.25
## 97 GUI Mini Gpg M 97 0.00 0.00
## 98 SEN Fleuve Gambie Gpg M 98 0.00 43.75
## 99 BKF Sissili Gt F 99 0.00 13.33
## 100 ZIM Rukomeshi Gmm NI 100 20.00 0.00
## 101 KEN Mwea nat. parc Gp NI 101 0.00 13.33
## 102 GUI Bafing Gpg M 102 0.00 0.00
## 103 GUI Tinkisso Gpg F 103 0.00 7.69
## 104 BKF Bouroum bouroum Gpg F 104 0.00 92.31
## 105 ZIM Mukondore Gmm NI 105 23.08 7.69
## 106 GUI Karifale Gpg M 106 0.00 8.33
## 107 GUI Lemonako Gpg F 107 0.00 0.00
## 108 BKF KouriGUIon Gpg F 108 0.00 50.00
## 109 MLI Bani Gpg M 109 0.00 0.00
## 110 MLI Sybi Gpg F 110 0.00 0.00
## 111 MLI Sybi Gpg M 111 0.00 0.00
## 112 GHA MorURTi Gt F 112 0.00 66.67
## 113 SEN Fleueve G Gpg F 113 0.00 11.11
## 114 BKF KouriGUIon Gpg NI 114 0.00 22.22
## 115 ZIM M. chiuyi Gmm NI 115 11.11 0.00
## 116 GHA Fumbissi Gt F 116 0.00 100.00
## 117 GUI Lemonako Gpg M 117 0.00 0.00
## 118 UGA Moyo Gp NI 118 87.50 12.50
## 119 SEN Diaguiri Gpg F 119 0.00 0.00
## 120 MLI Banco Gpg M 120 0.00 28.57
## 121 MLI Baoule Gpg F 121 0.00 42.86
## 122 MLI Baoule Gpg M 122 0.00 42.86
## 123 MLI Bougouni Gpg M 123 0.00 0.00
## 124 BKF Lorepeni Gpg F 124 0.00 71.43
## 125 GHA Fumbissi Gt M 125 0.00 100.00
## 126 GHA Grogro Gt M 126 0.00 100.00
## 127 SEN MousSAFlla Gpg M 127 0.00 0.00
## 128 MLI Baguineda Gpg F 128 0.00 16.67
## 129 MLI Bougouni Gpg F 129 0.00 0.00
## 130 MLI Kita Gpg M 130 0.00 16.67
## 131 ZIM Mushumb Gmm NI 131 0.00 33.33
## 132 URT Zanzibar Ga NI 132 16.67 0.00
## 133 GHA Grogro Gt F 133 0.00 100.00
## 134 GHA Kumpole Gt F 134 0.00 100.00
## 135 GHA Sissili Bidge Gt F 135 0.00 100.00
## 136 GUI Kifala Gpg F 136 0.00 0.00
## 137 BKF Bouroum bouroum Gpg M 137 0.00 80.00
## 138 SEN Fleuve Gambie Gpg F 138 0.00 25.00
## 139 ZIM Rukomeshi Gp NI 139 0.00 0.00
## 140 ZIM Gokwe Gp NI 140 0.00 0.00
## 141 URT Jozani Ga NI 141 0.00 25.00
## 142 GHA Bougouhiya Gt M 142 0.00 0.00
## 143 SEN Niokolo Gpg M 143 0.00 0.00
## 144 BKF Sissili Gmsm M 144 0.00 0.00
## 145 BKF Lorepeni Gpg M 145 0.00 100.00
## 146 BKF KouriGUIon Gpg M 146 0.00 66.67
## 147 BKF Ouarkoye Gpg M 147 0.00 100.00
## 148 GHA Kumpole Gt M 148 0.00 100.00
## 149 GHA Psikp_ Gt M 149 0.00 100.00
## 150 SEN Diaguiri Gpg M 150 0.00 50.00
## 151 BKF Ouarkoye Gpg F 151 0.00 100.00
## 152 GHA Kandiaga Gt M 152 0.00 100.00
## 153 GHA Sissili Bidge Gt M 153 0.00 100.00
## 154 GHA Nabogo Gt F 154 0.00 0.00
## 155 GHA Volta Blanche Gt F 155 0.00 0.00
## 156 GUI Karifale Gpg F 156 0.00 0.00
## 157 SEN Mako Gpg M 157 0.00 100.00
## Prev_Tc Prev_Tv Prev_Tz Prev_Tsg Prev_TcTv Prev_TcTz Prev_TcTsg Prev_TvTz
## 1 6.10 0.65 1.09 10.46 0.00 0.00 0.65 0.00
## 2 1.10 13.24 0.37 0.00 0.74 1.10 0.00 0.00
## 3 3.64 11.36 0.00 0.00 0.00 0.00 0.00 0.00
## 4 1.51 2.01 0.50 3.02 0.00 0.00 0.00 0.00
## 5 21.54 15.38 0.51 26.15 6.67 1.54 15.90 1.03
## 6 7.18 0.51 1.03 7.69 1.03 0.51 0.51 0.00
## 7 0.00 0.00 0.00 0.00 0.00 0.00 0.00 0.00
## 8 1.37 0.68 0.68 34.93 0.00 0.00 7.53 0.00
## 9 6.16 2.74 0.68 8.22 0.00 0.00 0.68 0.00
## 10 5.34 3.05 0.00 0.00 0.00 0.76 0.00 0.00
## 11 1.89 12.26 1.89 0.00 1.89 0.94 0.00 0.00
## 12 0.94 2.83 1.89 0.00 0.00 0.00 0.00 1.89
## 13 0.00 5.66 5.66 0.00 0.00 1.89 0.00 2.83
## 14 2.11 27.37 1.05 0.00 0.00 1.05 0.00 1.05
## 15 0.00 0.00 0.00 0.00 0.00 0.00 0.00 0.00
## 16 3.19 2.13 1.06 3.19 0.00 0.00 1.06 0.00
## 17 1.06 0.00 1.06 0.00 0.00 0.00 0.00 0.00
## 18 0.00 0.00 0.00 1.14 0.00 0.00 0.00 0.00
## 19 15.91 9.09 0.00 28.41 0.00 0.00 15.91 0.00
## 20 29.89 4.60 0.00 25.29 0.00 0.00 10.34 0.00
## 21 2.35 0.00 0.00 0.00 0.00 0.00 0.00 0.00
## 22 18.52 1.23 1.23 18.52 0.00 0.00 6.17 0.00
## 23 10.00 2.50 2.50 0.00 1.25 0.00 0.00 0.00
## 24 3.85 12.82 8.97 0.00 0.00 10.26 0.00 14.10
## 25 0.00 20.51 12.82 0.00 0.00 0.00 0.00 11.54
## 26 11.54 0.00 2.56 69.23 0.00 0.00 7.69 0.00
## 27 2.60 0.00 0.00 0.00 0.00 0.00 0.00 0.00
## 28 5.33 2.67 1.33 0.00 0.00 1.33 0.00 0.00
## 29 5.33 34.67 0.00 0.00 0.00 0.00 0.00 0.00
## 30 1.41 1.41 0.00 0.00 0.00 0.00 0.00 0.00
## 31 33.80 0.00 0.00 14.08 0.00 1.41 21.13 1.41
## 32 0.00 0.00 0.00 0.00 0.00 0.00 0.00 0.00
## 33 0.00 0.00 0.00 0.00 0.00 0.00 0.00 0.00
## 34 0.00 1.54 1.54 4.62 0.00 0.00 0.00 0.00
## 35 0.00 6.25 15.63 0.00 0.00 0.00 0.00 0.00
## 37 6.56 13.11 4.92 0.00 0.00 0.00 0.00 0.00
## 38 15.00 8.33 0.00 58.33 1.67 0.00 10.00 0.00
## 39 5.08 11.86 1.69 0.00 0.00 1.69 0.00 0.00
## 40 0.00 1.72 0.00 0.00 0.00 0.00 0.00 0.00
## 41 10.53 0.00 10.53 15.79 0.00 0.00 1.75 0.00
## 42 1.82 0.00 0.00 5.45 0.00 0.00 0.00 0.00
## 43 1.89 9.43 24.53 0.00 0.00 16.98 0.00 7.55
## 44 0.00 0.00 1.89 0.00 0.00 0.00 0.00 0.00
## 45 1.89 62.26 1.89 11.32 0.00 0.00 0.00 0.00
## 46 9.43 1.89 3.77 24.53 1.89 0.00 1.89 0.00
## 47 1.92 0.00 0.00 0.00 0.00 0.00 0.00 0.00
## 48 1.92 1.92 3.85 0.00 0.00 0.00 0.00 0.00
## 49 1.96 9.80 0.00 19.61 3.92 1.96 0.00 0.00
## 50 12.00 2.00 14.00 4.00 0.00 0.00 0.00 0.00
## 51 2.00 0.00 0.00 2.00 0.00 0.00 0.00 0.00
## 52 0.00 2.00 0.00 2.00 0.00 0.00 0.00 0.00
## 53 6.00 0.00 0.00 4.00 0.00 0.00 0.00 0.00
## 54 0.00 0.00 0.00 0.00 0.00 0.00 0.00 0.00
## 55 0.00 0.00 0.00 0.00 0.00 0.00 0.00 0.00
## 56 0.00 26.83 7.32 0.00 0.00 0.00 0.00 7.32
## 57 0.00 53.85 2.56 0.00 0.00 0.00 0.00 15.38
## 58 0.00 5.13 0.00 0.00 0.00 0.00 0.00 0.00
## 59 2.56 0.00 0.00 2.56 0.00 0.00 0.00 0.00
## 60 7.89 0.00 0.00 73.68 0.00 0.00 5.26 0.00
## 61 2.63 2.63 0.00 0.00 0.00 0.00 0.00 0.00
## 62 0.00 0.00 0.00 0.00 0.00 0.00 0.00 0.00
## 63 0.00 2.86 0.00 0.00 0.00 0.00 0.00 0.00
## 64 0.00 0.00 0.00 0.00 0.00 0.00 0.00 0.00
## 65 0.00 20.59 0.00 0.00 0.00 0.00 0.00 0.00
## 66 0.00 26.47 0.00 23.53 0.00 0.00 0.00 0.00
## 67 0.00 0.00 6.06 0.00 0.00 0.00 0.00 0.00
## 68 30.30 18.18 0.00 0.00 0.00 0.00 0.00 0.00
## 69 0.00 15.63 15.63 0.00 0.00 18.75 0.00 0.00
## 70 12.50 12.50 0.00 0.00 0.00 0.00 0.00 0.00
## 71 0.00 0.00 0.00 0.00 0.00 0.00 0.00 0.00
## 72 0.00 13.33 26.67 0.00 0.00 3.33 0.00 3.33
## 73 0.00 0.00 33.33 0.00 6.67 3.33 0.00 0.00
## 74 0.00 3.33 3.33 36.67 0.00 0.00 0.00 0.00
## 75 0.00 0.00 0.00 0.00 0.00 0.00 0.00 0.00
## 76 0.00 6.67 3.33 0.00 0.00 0.00 0.00 0.00
## 77 0.00 3.45 0.00 0.00 0.00 0.00 0.00 0.00
## 78 0.00 0.00 0.00 0.00 0.00 0.00 0.00 0.00
## 79 0.00 0.00 0.00 0.00 0.00 0.00 0.00 0.00
## 80 0.00 65.38 0.00 7.69 0.00 0.00 0.00 0.00
## 81 3.85 0.00 7.69 3.85 0.00 0.00 3.85 0.00
## 82 0.00 0.00 0.00 0.00 0.00 0.00 0.00 0.00
## 83 0.00 0.00 0.00 0.00 0.00 0.00 0.00 0.00
## 84 0.00 4.00 0.00 0.00 0.00 0.00 0.00 0.00
## 85 8.00 0.00 4.00 4.00 0.00 0.00 0.00 0.00
## 86 0.00 0.00 0.00 0.00 0.00 0.00 0.00 0.00
## 87 0.00 0.00 0.00 0.00 0.00 0.00 0.00 0.00
## 88 4.35 30.43 13.04 4.35 0.00 0.00 4.35 0.00
## 89 0.00 5.00 0.00 0.00 0.00 0.00 0.00 0.00
## 90 55.00 5.00 0.00 10.00 10.00 0.00 0.00 0.00
## 91 0.00 26.32 5.26 21.05 0.00 0.00 0.00 0.00
## 92 0.00 5.56 0.00 0.00 0.00 0.00 0.00 0.00
## 93 0.00 5.56 0.00 0.00 0.00 0.00 0.00 0.00
## 94 0.00 0.00 0.00 0.00 0.00 0.00 0.00 0.00
## 95 0.00 6.25 0.00 0.00 0.00 0.00 0.00 12.50
## 96 0.00 6.25 0.00 0.00 0.00 0.00 0.00 0.00
## 97 0.00 0.00 0.00 0.00 0.00 0.00 0.00 0.00
## 98 0.00 43.75 0.00 0.00 0.00 0.00 0.00 0.00
## 99 6.67 6.67 0.00 0.00 0.00 0.00 0.00 0.00
## 100 0.00 0.00 0.00 0.00 0.00 0.00 0.00 0.00
## 101 13.33 0.00 0.00 0.00 0.00 0.00 0.00 0.00
## 102 0.00 0.00 0.00 0.00 0.00 0.00 0.00 0.00
## 103 0.00 7.69 0.00 0.00 0.00 0.00 0.00 0.00
## 104 0.00 53.85 0.00 23.08 0.00 0.00 0.00 0.00
## 105 7.69 0.00 0.00 0.00 0.00 0.00 0.00 0.00
## 106 0.00 8.33 0.00 0.00 0.00 0.00 0.00 0.00
## 107 0.00 8.33 0.00 0.00 0.00 0.00 0.00 0.00
## 108 0.00 0.00 0.00 33.33 0.00 0.00 0.00 0.00
## 109 0.00 0.00 0.00 0.00 0.00 0.00 0.00 0.00
## 110 0.00 0.00 0.00 0.00 0.00 0.00 0.00 0.00
## 111 0.00 0.00 0.00 0.00 0.00 0.00 0.00 0.00
## 112 0.00 0.00 33.33 0.00 0.00 22.22 0.00 11.11
## 113 0.00 11.11 0.00 0.00 0.00 0.00 0.00 0.00
## 114 0.00 0.00 0.00 11.11 0.00 0.00 0.00 0.00
## 115 0.00 0.00 0.00 0.00 0.00 0.00 0.00 0.00
## 116 0.00 0.00 37.50 0.00 0.00 0.00 0.00 62.50
## 117 0.00 0.00 0.00 0.00 0.00 0.00 0.00 0.00
## 118 0.00 0.00 0.00 12.50 0.00 0.00 0.00 0.00
## 119 0.00 0.00 0.00 0.00 0.00 0.00 0.00 0.00
## 120 0.00 28.57 0.00 0.00 0.00 0.00 0.00 0.00
## 121 0.00 42.86 0.00 0.00 0.00 0.00 0.00 0.00
## 122 0.00 42.86 0.00 0.00 0.00 0.00 0.00 0.00
## 123 0.00 0.00 0.00 0.00 0.00 0.00 0.00 0.00
## 124 0.00 14.29 0.00 28.57 0.00 0.00 0.00 0.00
## 125 0.00 66.67 33.33 0.00 0.00 0.00 0.00 0.00
## 126 0.00 0.00 16.67 0.00 0.00 0.00 0.00 83.33
## 127 0.00 0.00 0.00 0.00 0.00 0.00 0.00 0.00
## 128 0.00 16.67 0.00 0.00 0.00 0.00 0.00 0.00
## 129 0.00 0.00 0.00 0.00 0.00 0.00 0.00 0.00
## 130 0.00 16.67 0.00 0.00 0.00 0.00 0.00 0.00
## 131 16.67 0.00 0.00 16.67 0.00 0.00 0.00 0.00
## 132 0.00 0.00 0.00 0.00 0.00 0.00 0.00 0.00
## 133 0.00 80.00 20.00 0.00 0.00 0.00 0.00 0.00
## 134 0.00 40.00 60.00 0.00 0.00 0.00 0.00 0.00
## 135 0.00 20.00 80.00 0.00 0.00 0.00 0.00 0.00
## 136 0.00 0.00 0.00 0.00 0.00 0.00 0.00 0.00
## 137 0.00 40.00 0.00 20.00 0.00 0.00 0.00 0.00
## 138 0.00 25.00 0.00 0.00 0.00 0.00 0.00 0.00
## 139 0.00 0.00 0.00 0.00 0.00 0.00 0.00 0.00
## 140 0.00 0.00 0.00 0.00 0.00 0.00 0.00 0.00
## 141 0.00 0.00 25.00 0.00 0.00 0.00 0.00 0.00
## 142 0.00 0.00 0.00 0.00 0.00 0.00 0.00 0.00
## 143 0.00 66.67 0.00 0.00 0.00 0.00 0.00 0.00
## 144 0.00 0.00 0.00 0.00 0.00 0.00 0.00 0.00
## 145 0.00 0.00 0.00 0.00 0.00 0.00 0.00 0.00
## 146 0.00 33.33 0.00 0.00 0.00 0.00 0.00 0.00
## 147 0.00 33.33 0.00 0.00 0.00 0.00 0.00 0.00
## 148 0.00 50.00 50.00 0.00 0.00 0.00 0.00 0.00
## 149 0.00 0.00 50.00 0.00 0.00 0.00 0.00 50.00
## 150 0.00 0.00 50.00 0.00 0.00 0.00 0.00 0.00
## 151 50.00 50.00 0.00 0.00 0.00 0.00 0.00 0.00
## 152 0.00 0.00 100.00 0.00 0.00 0.00 0.00 0.00
## 153 0.00 0.00 100.00 0.00 0.00 0.00 0.00 0.00
## 154 0.00 0.00 0.00 0.00 0.00 0.00 0.00 0.00
## 155 0.00 0.00 0.00 0.00 0.00 0.00 0.00 0.00
## 156 0.00 0.00 0.00 0.00 0.00 0.00 0.00 0.00
## 157 0.00 100.00 0.00 0.00 0.00 0.00 0.00 0.00
## Prev_TvTsg Prev_TzTsg Prev_TcTvTz
## 1 0.44 0.65 0.00
## 2 0.00 0.00 0.00
## 3 0.00 0.00 0.00
## 4 0.00 0.00 0.00
## 5 0.00 0.00 0.00
## 6 0.00 1.03 0.00
## 7 0.00 0.00 0.00
## 8 0.00 0.00 0.00
## 9 0.68 0.68 0.00
## 10 0.00 0.00 0.00
## 11 0.00 0.00 0.00
## 12 0.00 0.00 0.00
## 13 0.00 0.00 0.00
## 14 0.00 0.00 0.00
## 15 0.00 0.00 0.00
## 16 0.00 0.00 0.00
## 17 0.00 0.00 0.00
## 18 0.00 0.00 0.00
## 19 0.00 2.27 0.00
## 20 0.00 0.00 0.00
## 21 0.00 0.00 0.00
## 22 6.17 1.23 0.00
## 23 1.25 0.00 0.00
## 24 0.00 0.00 1.28
## 25 0.00 0.00 0.00
## 26 0.00 0.00 0.00
## 27 0.00 0.00 0.00
## 28 0.00 0.00 0.00
## 29 0.00 0.00 0.00
## 30 0.00 0.00 0.00
## 31 0.00 1.41 0.00
## 32 0.00 0.00 0.00
## 33 0.00 0.00 0.00
## 34 0.00 0.00 0.00
## 35 0.00 0.00 0.00
## 37 0.00 0.00 0.00
## 38 0.00 0.00 0.00
## 39 0.00 0.00 0.00
## 40 0.00 0.00 0.00
## 41 0.00 0.00 0.00
## 42 0.00 0.00 0.00
## 43 0.00 0.00 5.66
## 44 0.00 0.00 0.00
## 45 9.43 3.77 0.00
## 46 0.00 0.00 0.00
## 47 0.00 0.00 0.00
## 48 0.00 0.00 0.00
## 49 0.00 0.00 0.00
## 50 0.00 0.00 0.00
## 51 0.00 0.00 0.00
## 52 2.00 0.00 0.00
## 53 0.00 0.00 0.00
## 54 0.00 0.00 0.00
## 55 0.00 0.00 0.00
## 56 0.00 0.00 0.00
## 57 0.00 0.00 0.00
## 58 0.00 0.00 0.00
## 59 0.00 0.00 0.00
## 60 0.00 0.00 0.00
## 61 0.00 0.00 0.00
## 62 0.00 0.00 0.00
## 63 0.00 0.00 0.00
## 64 0.00 0.00 0.00
## 65 0.00 0.00 0.00
## 66 2.94 0.00 0.00
## 67 0.00 0.00 0.00
## 68 0.00 0.00 0.00
## 69 0.00 0.00 0.00
## 70 0.00 0.00 0.00
## 71 0.00 0.00 0.00
## 72 0.00 0.00 3.33
## 73 0.00 0.00 3.33
## 74 0.00 0.00 0.00
## 75 0.00 0.00 0.00
## 76 0.00 0.00 0.00
## 77 0.00 0.00 0.00
## 78 0.00 0.00 0.00
## 79 0.00 0.00 0.00
## 80 7.69 0.00 0.00
## 81 0.00 0.00 0.00
## 82 0.00 0.00 0.00
## 83 0.00 0.00 0.00
## 84 0.00 0.00 0.00
## 85 0.00 0.00 0.00
## 86 0.00 0.00 0.00
## 87 0.00 0.00 0.00
## 88 4.35 8.70 0.00
## 89 0.00 0.00 0.00
## 90 0.00 0.00 0.00
## 91 31.58 0.00 0.00
## 92 0.00 0.00 0.00
## 93 0.00 0.00 0.00
## 94 0.00 0.00 0.00
## 95 0.00 0.00 0.00
## 96 0.00 0.00 0.00
## 97 0.00 0.00 0.00
## 98 0.00 0.00 0.00
## 99 0.00 0.00 0.00
## 100 0.00 0.00 0.00
## 101 0.00 0.00 0.00
## 102 0.00 0.00 0.00
## 103 0.00 0.00 0.00
## 104 15.38 0.00 0.00
## 105 0.00 0.00 0.00
## 106 0.00 0.00 0.00
## 107 0.00 0.00 0.00
## 108 16.67 0.00 0.00
## 109 0.00 0.00 0.00
## 110 0.00 0.00 0.00
## 111 0.00 0.00 0.00
## 112 0.00 0.00 0.00
## 113 0.00 0.00 0.00
## 114 11.11 0.00 0.00
## 115 0.00 0.00 0.00
## 116 0.00 0.00 0.00
## 117 0.00 0.00 0.00
## 118 0.00 0.00 0.00
## 119 0.00 0.00 0.00
## 120 0.00 0.00 0.00
## 121 0.00 0.00 0.00
## 122 0.00 0.00 0.00
## 123 0.00 0.00 0.00
## 124 28.57 0.00 0.00
## 125 0.00 0.00 0.00
## 126 0.00 0.00 0.00
## 127 0.00 0.00 0.00
## 128 0.00 0.00 0.00
## 129 0.00 0.00 0.00
## 130 0.00 0.00 0.00
## 131 0.00 0.00 0.00
## 132 0.00 0.00 0.00
## 133 0.00 0.00 0.00
## 134 0.00 0.00 0.00
## 135 0.00 0.00 0.00
## 136 0.00 0.00 0.00
## 137 20.00 0.00 0.00
## 138 0.00 0.00 0.00
## 139 0.00 0.00 0.00
## 140 0.00 0.00 0.00
## 141 0.00 0.00 0.00
## 142 0.00 0.00 0.00
## 143 0.00 0.00 0.00
## 144 0.00 0.00 0.00
## 145 66.67 33.33 0.00
## 146 33.33 0.00 0.00
## 147 33.33 33.33 0.00
## 148 0.00 0.00 0.00
## 149 0.00 0.00 0.00
## 150 0.00 0.00 0.00
## 151 0.00 0.00 0.00
## 152 0.00 0.00 0.00
## 153 0.00 0.00 0.00
## 154 0.00 0.00 0.00
## 155 0.00 0.00 0.00
## 156 0.00 0.00 0.00
## 157 0.00 0.00 0.00

data$Country=as.factor(data$Country)
data$Localisation=as.factor(data$Localisation)
data$Species=as.factor(data$Species)
data$Sex=as.factor(data$Sex)
str(data)

## 'data.frame': 156 obs. of 18 variables:
## $ Country : Factor w/ 15 levels "BKF","ETH","GHA",..: 2 1 1 9 5 5 8 14 12 1 ...
## $ Localisation: Factor w/ 94 levels "Arba minch, nech SAFr",..: 1 14 14 74 37 65 73 56 90 14 ...
## $ Species : Factor w/ 10 levels "Ga","Gb","Gff",..: 7 10 10 8 7 7 2 7 7 6 ...
## $ Sex : Factor w/ 3 levels "F","M","NI": 3 1 2 3 3 3 3 3 3 1 ...
## $ Sample : int 1 2 3 4 5 6 7 8 9 10 ...
## $ Prev_Sod : num 20.5 0 0 0 42.6 ...
## $ Prev_Tspp : num 20.04 16.54 15 7.04 88.72 ...
## $ Prev_Tc : num 6.1 1.1 3.64 1.51 21.54 ...
## $ Prev_Tv : num 0.65 13.24 11.36 2.01 15.38 ...
## $ Prev_Tz : num 1.09 0.37 0 0.5 0.51 1.03 0 0.68 0.68 0 ...
## $ Prev_Tsg : num 10.46 0 0 3.02 26.15 ...
## $ Prev_TcTv : num 0 0.74 0 0 6.67 1.03 0 0 0 0 ...
## $ Prev_TcTz : num 0 1.1 0 0 1.54 0.51 0 0 0 0.76 ...
## $ Prev_TcTsg : num 0.65 0 0 0 15.9 0.51 0 7.53 0.68 0 ...
## $ Prev_TvTz : num 0 0 0 0 1.03 0 0 0 0 0 ...
## $ Prev_TvTsg : num 0.44 0 0 0 0 0 0 0 0.68 0 ...
## $ Prev_TzTsg : num 0.65 0 0 0 0 1.03 0 0 0.68 0 ...
## $ Prev_TcTvTz : num 0 0 0 0 0 0 0 0 0 0 ...
## - attr(*, "na.action")= 'omit' Named int 36
## ..- attr(*, "names")= chr "36"

attach(data)

## The following objects are masked from data (pos = 3):
##
## Country, Localisation, Prev_Sod, Prev_Tc, Prev_TcTsg, Prev_TcTv,
## Prev_TcTvTz, Prev_TcTz, Prev_Tsg, Prev_Tspp, Prev_Tv, Prev_TvTsg,
## Prev_TvTz, Prev_Tz, Prev_TzTsg, Sample, Sex, Species

head(data)

## Country Localisation Species Sex Sample Prev_Sod Prev_Tspp Prev_Tc
## 1 ETH Arba minch, nech SAFr Gp NI 1 20.48 20.04 6.10
## 2 BKF Comoe Gt F 2 0.00 16.54 1.10
## 3 BKF Comoe Gt M 3 0.00 15.00 3.64
## 4 SEN Pout Gpg NI 4 0.00 7.04 1.51
## 5 KEN Katotoi Gp NI 5 42.56 88.72 21.54
## 6 KEN Mwea Gp NI 6 3.08 19.49 7.18
## Prev_Tv Prev_Tz Prev_Tsg Prev_TcTv Prev_TcTz Prev_TcTsg Prev_TvTz Prev_TvTsg
## 1 0.65 1.09 10.46 0.00 0.00 0.65 0.00 0.44
## 2 13.24 0.37 0.00 0.74 1.10 0.00 0.00 0.00
## 3 11.36 0.00 0.00 0.00 0.00 0.00 0.00 0.00
## 4 2.01 0.50 3.02 0.00 0.00 0.00 0.00 0.00
## 5 15.38 0.51 26.15 6.67 1.54 15.90 1.03 0.00
## 6 0.51 1.03 7.69 1.03 0.51 0.51 0.00 0.00
## Prev_TzTsg Prev_TcTvTz
## 1 0.65 0
## 2 0.00 0
## 3 0.00 0
## 4 0.00 0
## 5 0.00 0
## 6 1.03 0

data=na.omit(data)
data

## Country Localisation Species Sex Sample Prev_Sod Prev_Tspp
## 1 ETH Arba minch, nech SAFr Gp NI 1 20.48 20.04
## 2 BKF Comoe Gt F 2 0.00 16.54
## 3 BKF Comoe Gt M 3 0.00 15.00
## 4 SEN Pout Gpg NI 4 0.00 7.04
## 5 KEN Katotoi Gp NI 5 42.56 88.72
## 6 KEN Mwea Gp NI 6 3.08 19.49
## 7 SAF Phinda Gb NI 7 4.12 0.00
## 8 ZAM Mfuwe Gp NI 8 2.05 45.21
## 9 URT URTzania Gp NI 9 78.77 19.86
## 10 BKF Comoe Gmsm F 10 0.00 9.16
## 11 BKF Folonzo Gt F 11 0.00 18.87
## 12 BKF Folonzo Gpg F 12 0.00 7.55
## 13 BKF Folonzo Gpg M 13 1.89 16.04
## 14 BKF Folonzo Gt M 14 0.00 32.63
## 15 GUI Kangoliya Gpg F 15 95.74 0.00
## 16 UGA Buvuma island Gff NI 16 4.26 10.64
## 17 KEN Kari Gp NI 17 89.36 2.13
## 18 SEN Kayar Gpg NI 18 0.00 1.14
## 19 KEN Koibos Gp NI 19 0.00 71.59
## 20 KEN Meru nat. parc Gp NI 20 22.99 70.11
## 21 KEN Kari Gmm NI 21 63.53 2.35
## 22 URT MaSAFng-URTga Gmm NI 22 76.54 53.09
## 23 KEN Ruma nat. parc Gp NI 23 26.25 17.50
## 24 GHA Walewale Gt M 24 0.00 53.85
## 25 BKF Moussodougou Gpg F 25 0.00 44.87
## 26 ZIM Makuti Gmm NI 26 26.92 91.03
## 27 SAF False Bay Park Ga NI 27 0.00 2.60
## 28 BKF Comoe Gmsm M 28 0.00 10.67
## 29 BKF Folonzo Gmsm M 29 0.00 40.00
## 30 BKF Comoe Gpg F 30 0.00 2.82
## 31 URT MaSAFng-URTga Gp NI 31 66.20 73.24
## 32 BKF Kartasso Gpg F 32 0.00 0.00
## 33 BKF Kartasso Gpg M 33 0.00 0.00
## 34 SEN DiackSAFo Peulh Gpg NI 34 0.00 7.69
## 35 BKF Moussodougou Gpg M 35 0.00 21.88
## 37 BKF Comoe Gmed M 37 6.56 24.59
## 38 KEN Emsos Gp NI 38 0.00 93.33
## 39 BKF Folonzo Gmsm F 39 1.69 20.34
## 40 MLI Bani Gpg F 40 0.00 1.72
## 41 SAF SAFint Lucia Ga NI 41 0.00 38.60
## 42 UGA Budaka Gp NI 42 94.55 7.27
## 43 GHA Walewale Gt F 43 0.00 66.04
## 44 BKF Arly Gt F 44 0.00 1.89
## 45 BKF Kampty Gpg F 45 0.00 90.57
## 46 SAF Lower Mkhuze Ga NI 46 0.00 43.40
## 47 BKF Comoe Gpg M 47 0.00 1.92
## 48 MLI SEN Gpg M 48 0.00 7.69
## 49 KEN Ikapolok Gff NI 49 39.22 37.25
## 50 SAF Hluhluwe Gb NI 50 0.00 32.00
## 51 SAF North eastern KwaZulu-Natal Gb NI 51 0.00 4.00
## 52 MOZ Reserva Especial de Maputo Gb NI 52 14.00 6.00
## 53 MOZ Reserva Especial de Maputo Ga NI 53 0.00 10.00
## 54 BKF Bama Gpg F 54 0.00 0.00
## 55 BKF Arly Gt M 55 0.00 0.00
## 56 SEN Tambacounda Gpg F 56 0.00 41.46
## 57 SEN Tambacounda Gpg M 57 0.00 71.79
## 58 SEN SebikoURTe Gpg NI 58 0.00 5.13
## 59 SAF North eastern KwaZulu-Natal Ga NI 59 5.13 5.13
## 60 ZIM Makuti Gp NI 60 15.79 86.84
## 61 KEN Obekai Gff NI 61 0.00 5.26
## 62 MLI SEN Gpg F 62 0.00 0.00
## 63 ZAI ZAI Gpp NI 63 11.43 2.86
## 64 BKF Bama Gpg M 64 0.00 0.00
## 65 MLI Banco Gpg F 65 0.00 20.59
## 66 BKF Dedougou Gpg F 66 0.00 52.94
## 67 MLI Sikasso Gpg F 67 0.00 6.06
## 68 BKF Comoe Gmed F 68 12.12 51.52
## 69 GHA MorURTi Gt M 69 0.00 50.00
## 70 BKF Sissili Gt M 70 0.00 25.00
## 71 GUI Kangoliya Gpg M 71 0.00 0.00
## 72 BKF Folonzo Gmed F 72 0.00 50.00
## 73 BKF Folonzo Gmed M 73 0.00 46.67
## 74 SAF SAFint Lucia Gb NI 74 0.00 43.33
## 75 SWA Mlawula Nature Reserve Ga NI 75 0.00 0.00
## 76 URT Uguja island Ga NI 76 6.67 10.00
## 77 GUI Mini Gpg F 77 0.00 3.45
## 78 SEN Hann Gpg NI 78 0.00 0.00
## 79 UGA Omugo Gp NI 79 100.00 0.00
## 80 BKF Kampty Gpg M 80 0.00 80.77
## 81 ZIM Mushumb Gp NI 81 3.85 19.23
## 82 GUI Kifala Gpg M 82 0.00 0.00
## 83 MLI Sikasso Gpg M 83 0.00 0.00
## 84 MLI SS Gpg F 84 0.00 4.00
## 85 UGA Lira Gp NI 85 0.00 16.00
## 86 BKF Kenedougou Gpg F 86 0.00 0.00
## 87 MLI SS Gpg M 87 0.00 0.00
## 88 BKF Dedougou Gpg M 88 0.00 69.57
## 89 GUI Bafing Gpg F 89 0.00 5.00
## 90 KEN Kiria Gp NI 90 0.00 80.00
## 91 BKF Kampty Gpg NI 91 0.00 84.21
## 92 GUI Tinkisso Gpg M 92 0.00 5.56
## 93 ZIM Kemukura Gmm NI 93 22.22 5.56
## 94 BKF Kenedougou Gpg M 94 0.00 0.00
## 95 GHA Bougouhiya Gt F 95 0.00 18.75
## 96 GUI Dekonkore Gpg F 96 0.00 6.25
## 97 GUI Mini Gpg M 97 0.00 0.00
## 98 SEN Fleuve Gambie Gpg M 98 0.00 43.75
## 99 BKF Sissili Gt F 99 0.00 13.33
## 100 ZIM Rukomeshi Gmm NI 100 20.00 0.00
## 101 KEN Mwea nat. parc Gp NI 101 0.00 13.33
## 102 GUI Bafing Gpg M 102 0.00 0.00
## 103 GUI Tinkisso Gpg F 103 0.00 7.69
## 104 BKF Bouroum bouroum Gpg F 104 0.00 92.31
## 105 ZIM Mukondore Gmm NI 105 23.08 7.69
## 106 GUI Karifale Gpg M 106 0.00 8.33
## 107 GUI Lemonako Gpg F 107 0.00 0.00
## 108 BKF KouriGUIon Gpg F 108 0.00 50.00
## 109 MLI Bani Gpg M 109 0.00 0.00
## 110 MLI Sybi Gpg F 110 0.00 0.00
## 111 MLI Sybi Gpg M 111 0.00 0.00
## 112 GHA MorURTi Gt F 112 0.00 66.67
## 113 SEN Fleueve G Gpg F 113 0.00 11.11
## 114 BKF KouriGUIon Gpg NI 114 0.00 22.22
## 115 ZIM M. chiuyi Gmm NI 115 11.11 0.00
## 116 GHA Fumbissi Gt F 116 0.00 100.00
## 117 GUI Lemonako Gpg M 117 0.00 0.00
## 118 UGA Moyo Gp NI 118 87.50 12.50
## 119 SEN Diaguiri Gpg F 119 0.00 0.00
## 120 MLI Banco Gpg M 120 0.00 28.57
## 121 MLI Baoule Gpg F 121 0.00 42.86
## 122 MLI Baoule Gpg M 122 0.00 42.86
## 123 MLI Bougouni Gpg M 123 0.00 0.00
## 124 BKF Lorepeni Gpg F 124 0.00 71.43
## 125 GHA Fumbissi Gt M 125 0.00 100.00
## 126 GHA Grogro Gt M 126 0.00 100.00
## 127 SEN MousSAFlla Gpg M 127 0.00 0.00
## 128 MLI Baguineda Gpg F 128 0.00 16.67
## 129 MLI Bougouni Gpg F 129 0.00 0.00
## 130 MLI Kita Gpg M 130 0.00 16.67
## 131 ZIM Mushumb Gmm NI 131 0.00 33.33
## 132 URT Zanzibar Ga NI 132 16.67 0.00
## 133 GHA Grogro Gt F 133 0.00 100.00
## 134 GHA Kumpole Gt F 134 0.00 100.00
## 135 GHA Sissili Bidge Gt F 135 0.00 100.00
## 136 GUI Kifala Gpg F 136 0.00 0.00
## 137 BKF Bouroum bouroum Gpg M 137 0.00 80.00
## 138 SEN Fleuve Gambie Gpg F 138 0.00 25.00
## 139 ZIM Rukomeshi Gp NI 139 0.00 0.00
## 140 ZIM Gokwe Gp NI 140 0.00 0.00
## 141 URT Jozani Ga NI 141 0.00 25.00
## 142 GHA Bougouhiya Gt M 142 0.00 0.00
## 143 SEN Niokolo Gpg M 143 0.00 0.00
## 144 BKF Sissili Gmsm M 144 0.00 0.00
## 145 BKF Lorepeni Gpg M 145 0.00 100.00
## 146 BKF KouriGUIon Gpg M 146 0.00 66.67
## 147 BKF Ouarkoye Gpg M 147 0.00 100.00
## 148 GHA Kumpole Gt M 148 0.00 100.00
## 149 GHA Psikp_ Gt M 149 0.00 100.00
## 150 SEN Diaguiri Gpg M 150 0.00 50.00
## 151 BKF Ouarkoye Gpg F 151 0.00 100.00
## 152 GHA Kandiaga Gt M 152 0.00 100.00
## 153 GHA Sissili Bidge Gt M 153 0.00 100.00
## 154 GHA Nabogo Gt F 154 0.00 0.00
## 155 GHA Volta Blanche Gt F 155 0.00 0.00
## 156 GUI Karifale Gpg F 156 0.00 0.00
## 157 SEN Mako Gpg M 157 0.00 100.00
## Prev_Tc Prev_Tv Prev_Tz Prev_Tsg Prev_TcTv Prev_TcTz Prev_TcTsg Prev_TvTz
## 1 6.10 0.65 1.09 10.46 0.00 0.00 0.65 0.00
## 2 1.10 13.24 0.37 0.00 0.74 1.10 0.00 0.00
## 3 3.64 11.36 0.00 0.00 0.00 0.00 0.00 0.00
## 4 1.51 2.01 0.50 3.02 0.00 0.00 0.00 0.00
## 5 21.54 15.38 0.51 26.15 6.67 1.54 15.90 1.03
## 6 7.18 0.51 1.03 7.69 1.03 0.51 0.51 0.00
## 7 0.00 0.00 0.00 0.00 0.00 0.00 0.00 0.00
## 8 1.37 0.68 0.68 34.93 0.00 0.00 7.53 0.00
## 9 6.16 2.74 0.68 8.22 0.00 0.00 0.68 0.00
## 10 5.34 3.05 0.00 0.00 0.00 0.76 0.00 0.00
## 11 1.89 12.26 1.89 0.00 1.89 0.94 0.00 0.00
## 12 0.94 2.83 1.89 0.00 0.00 0.00 0.00 1.89
## 13 0.00 5.66 5.66 0.00 0.00 1.89 0.00 2.83
## 14 2.11 27.37 1.05 0.00 0.00 1.05 0.00 1.05
## 15 0.00 0.00 0.00 0.00 0.00 0.00 0.00 0.00
## 16 3.19 2.13 1.06 3.19 0.00 0.00 1.06 0.00
## 17 1.06 0.00 1.06 0.00 0.00 0.00 0.00 0.00
## 18 0.00 0.00 0.00 1.14 0.00 0.00 0.00 0.00
## 19 15.91 9.09 0.00 28.41 0.00 0.00 15.91 0.00
## 20 29.89 4.60 0.00 25.29 0.00 0.00 10.34 0.00
## 21 2.35 0.00 0.00 0.00 0.00 0.00 0.00 0.00
## 22 18.52 1.23 1.23 18.52 0.00 0.00 6.17 0.00
## 23 10.00 2.50 2.50 0.00 1.25 0.00 0.00 0.00
## 24 3.85 12.82 8.97 0.00 0.00 10.26 0.00 14.10
## 25 0.00 20.51 12.82 0.00 0.00 0.00 0.00 11.54
## 26 11.54 0.00 2.56 69.23 0.00 0.00 7.69 0.00
## 27 2.60 0.00 0.00 0.00 0.00 0.00 0.00 0.00
## 28 5.33 2.67 1.33 0.00 0.00 1.33 0.00 0.00
## 29 5.33 34.67 0.00 0.00 0.00 0.00 0.00 0.00
## 30 1.41 1.41 0.00 0.00 0.00 0.00 0.00 0.00
## 31 33.80 0.00 0.00 14.08 0.00 1.41 21.13 1.41
## 32 0.00 0.00 0.00 0.00 0.00 0.00 0.00 0.00
## 33 0.00 0.00 0.00 0.00 0.00 0.00 0.00 0.00
## 34 0.00 1.54 1.54 4.62 0.00 0.00 0.00 0.00
## 35 0.00 6.25 15.63 0.00 0.00 0.00 0.00 0.00
## 37 6.56 13.11 4.92 0.00 0.00 0.00 0.00 0.00
## 38 15.00 8.33 0.00 58.33 1.67 0.00 10.00 0.00
## 39 5.08 11.86 1.69 0.00 0.00 1.69 0.00 0.00
## 40 0.00 1.72 0.00 0.00 0.00 0.00 0.00 0.00
## 41 10.53 0.00 10.53 15.79 0.00 0.00 1.75 0.00
## 42 1.82 0.00 0.00 5.45 0.00 0.00 0.00 0.00
## 43 1.89 9.43 24.53 0.00 0.00 16.98 0.00 7.55
## 44 0.00 0.00 1.89 0.00 0.00 0.00 0.00 0.00
## 45 1.89 62.26 1.89 11.32 0.00 0.00 0.00 0.00
## 46 9.43 1.89 3.77 24.53 1.89 0.00 1.89 0.00
## 47 1.92 0.00 0.00 0.00 0.00 0.00 0.00 0.00
## 48 1.92 1.92 3.85 0.00 0.00 0.00 0.00 0.00
## 49 1.96 9.80 0.00 19.61 3.92 1.96 0.00 0.00
## 50 12.00 2.00 14.00 4.00 0.00 0.00 0.00 0.00
## 51 2.00 0.00 0.00 2.00 0.00 0.00 0.00 0.00
## 52 0.00 2.00 0.00 2.00 0.00 0.00 0.00 0.00
## 53 6.00 0.00 0.00 4.00 0.00 0.00 0.00 0.00
## 54 0.00 0.00 0.00 0.00 0.00 0.00 0.00 0.00
## 55 0.00 0.00 0.00 0.00 0.00 0.00 0.00 0.00
## 56 0.00 26.83 7.32 0.00 0.00 0.00 0.00 7.32
## 57 0.00 53.85 2.56 0.00 0.00 0.00 0.00 15.38
## 58 0.00 5.13 0.00 0.00 0.00 0.00 0.00 0.00
## 59 2.56 0.00 0.00 2.56 0.00 0.00 0.00 0.00
## 60 7.89 0.00 0.00 73.68 0.00 0.00 5.26 0.00
## 61 2.63 2.63 0.00 0.00 0.00 0.00 0.00 0.00
## 62 0.00 0.00 0.00 0.00 0.00 0.00 0.00 0.00
## 63 0.00 2.86 0.00 0.00 0.00 0.00 0.00 0.00
## 64 0.00 0.00 0.00 0.00 0.00 0.00 0.00 0.00
## 65 0.00 20.59 0.00 0.00 0.00 0.00 0.00 0.00
## 66 0.00 26.47 0.00 23.53 0.00 0.00 0.00 0.00
## 67 0.00 0.00 6.06 0.00 0.00 0.00 0.00 0.00
## 68 30.30 18.18 0.00 0.00 0.00 0.00 0.00 0.00
## 69 0.00 15.63 15.63 0.00 0.00 18.75 0.00 0.00
## 70 12.50 12.50 0.00 0.00 0.00 0.00 0.00 0.00
## 71 0.00 0.00 0.00 0.00 0.00 0.00 0.00 0.00
## 72 0.00 13.33 26.67 0.00 0.00 3.33 0.00 3.33
## 73 0.00 0.00 33.33 0.00 6.67 3.33 0.00 0.00
## 74 0.00 3.33 3.33 36.67 0.00 0.00 0.00 0.00
## 75 0.00 0.00 0.00 0.00 0.00 0.00 0.00 0.00
## 76 0.00 6.67 3.33 0.00 0.00 0.00 0.00 0.00
## 77 0.00 3.45 0.00 0.00 0.00 0.00 0.00 0.00
## 78 0.00 0.00 0.00 0.00 0.00 0.00 0.00 0.00
## 79 0.00 0.00 0.00 0.00 0.00 0.00 0.00 0.00
## 80 0.00 65.38 0.00 7.69 0.00 0.00 0.00 0.00
## 81 3.85 0.00 7.69 3.85 0.00 0.00 3.85 0.00
## 82 0.00 0.00 0.00 0.00 0.00 0.00 0.00 0.00
## 83 0.00 0.00 0.00 0.00 0.00 0.00 0.00 0.00
## 84 0.00 4.00 0.00 0.00 0.00 0.00 0.00 0.00
## 85 8.00 0.00 4.00 4.00 0.00 0.00 0.00 0.00
## 86 0.00 0.00 0.00 0.00 0.00 0.00 0.00 0.00
## 87 0.00 0.00 0.00 0.00 0.00 0.00 0.00 0.00
## 88 4.35 30.43 13.04 4.35 0.00 0.00 4.35 0.00
## 89 0.00 5.00 0.00 0.00 0.00 0.00 0.00 0.00
## 90 55.00 5.00 0.00 10.00 10.00 0.00 0.00 0.00
## 91 0.00 26.32 5.26 21.05 0.00 0.00 0.00 0.00
## 92 0.00 5.56 0.00 0.00 0.00 0.00 0.00 0.00
## 93 0.00 5.56 0.00 0.00 0.00 0.00 0.00 0.00
## 94 0.00 0.00 0.00 0.00 0.00 0.00 0.00 0.00
## 95 0.00 6.25 0.00 0.00 0.00 0.00 0.00 12.50
## 96 0.00 6.25 0.00 0.00 0.00 0.00 0.00 0.00
## 97 0.00 0.00 0.00 0.00 0.00 0.00 0.00 0.00
## 98 0.00 43.75 0.00 0.00 0.00 0.00 0.00 0.00
## 99 6.67 6.67 0.00 0.00 0.00 0.00 0.00 0.00
## 100 0.00 0.00 0.00 0.00 0.00 0.00 0.00 0.00
## 101 13.33 0.00 0.00 0.00 0.00 0.00 0.00 0.00
## 102 0.00 0.00 0.00 0.00 0.00 0.00 0.00 0.00
## 103 0.00 7.69 0.00 0.00 0.00 0.00 0.00 0.00
## 104 0.00 53.85 0.00 23.08 0.00 0.00 0.00 0.00
## 105 7.69 0.00 0.00 0.00 0.00 0.00 0.00 0.00
## 106 0.00 8.33 0.00 0.00 0.00 0.00 0.00 0.00
## 107 0.00 8.33 0.00 0.00 0.00 0.00 0.00 0.00
## 108 0.00 0.00 0.00 33.33 0.00 0.00 0.00 0.00
## 109 0.00 0.00 0.00 0.00 0.00 0.00 0.00 0.00
## 110 0.00 0.00 0.00 0.00 0.00 0.00 0.00 0.00
## 111 0.00 0.00 0.00 0.00 0.00 0.00 0.00 0.00
## 112 0.00 0.00 33.33 0.00 0.00 22.22 0.00 11.11
## 113 0.00 11.11 0.00 0.00 0.00 0.00 0.00 0.00
## 114 0.00 0.00 0.00 11.11 0.00 0.00 0.00 0.00
## 115 0.00 0.00 0.00 0.00 0.00 0.00 0.00 0.00
## 116 0.00 0.00 37.50 0.00 0.00 0.00 0.00 62.50
## 117 0.00 0.00 0.00 0.00 0.00 0.00 0.00 0.00
## 118 0.00 0.00 0.00 12.50 0.00 0.00 0.00 0.00
## 119 0.00 0.00 0.00 0.00 0.00 0.00 0.00 0.00
## 120 0.00 28.57 0.00 0.00 0.00 0.00 0.00 0.00
## 121 0.00 42.86 0.00 0.00 0.00 0.00 0.00 0.00
## 122 0.00 42.86 0.00 0.00 0.00 0.00 0.00 0.00
## 123 0.00 0.00 0.00 0.00 0.00 0.00 0.00 0.00
## 124 0.00 14.29 0.00 28.57 0.00 0.00 0.00 0.00
## 125 0.00 66.67 33.33 0.00 0.00 0.00 0.00 0.00
## 126 0.00 0.00 16.67 0.00 0.00 0.00 0.00 83.33
## 127 0.00 0.00 0.00 0.00 0.00 0.00 0.00 0.00
## 128 0.00 16.67 0.00 0.00 0.00 0.00 0.00 0.00
## 129 0.00 0.00 0.00 0.00 0.00 0.00 0.00 0.00
## 130 0.00 16.67 0.00 0.00 0.00 0.00 0.00 0.00
## 131 16.67 0.00 0.00 16.67 0.00 0.00 0.00 0.00
## 132 0.00 0.00 0.00 0.00 0.00 0.00 0.00 0.00
## 133 0.00 80.00 20.00 0.00 0.00 0.00 0.00 0.00
## 134 0.00 40.00 60.00 0.00 0.00 0.00 0.00 0.00
## 135 0.00 20.00 80.00 0.00 0.00 0.00 0.00 0.00
## 136 0.00 0.00 0.00 0.00 0.00 0.00 0.00 0.00
## 137 0.00 40.00 0.00 20.00 0.00 0.00 0.00 0.00
## 138 0.00 25.00 0.00 0.00 0.00 0.00 0.00 0.00
## 139 0.00 0.00 0.00 0.00 0.00 0.00 0.00 0.00
## 140 0.00 0.00 0.00 0.00 0.00 0.00 0.00 0.00
## 141 0.00 0.00 25.00 0.00 0.00 0.00 0.00 0.00
## 142 0.00 0.00 0.00 0.00 0.00 0.00 0.00 0.00
## 143 0.00 66.67 0.00 0.00 0.00 0.00 0.00 0.00
## 144 0.00 0.00 0.00 0.00 0.00 0.00 0.00 0.00
## 145 0.00 0.00 0.00 0.00 0.00 0.00 0.00 0.00
## 146 0.00 33.33 0.00 0.00 0.00 0.00 0.00 0.00
## 147 0.00 33.33 0.00 0.00 0.00 0.00 0.00 0.00
## 148 0.00 50.00 50.00 0.00 0.00 0.00 0.00 0.00
## 149 0.00 0.00 50.00 0.00 0.00 0.00 0.00 50.00
## 150 0.00 0.00 50.00 0.00 0.00 0.00 0.00 0.00
## 151 50.00 50.00 0.00 0.00 0.00 0.00 0.00 0.00
## 152 0.00 0.00 100.00 0.00 0.00 0.00 0.00 0.00
## 153 0.00 0.00 100.00 0.00 0.00 0.00 0.00 0.00
## 154 0.00 0.00 0.00 0.00 0.00 0.00 0.00 0.00
## 155 0.00 0.00 0.00 0.00 0.00 0.00 0.00 0.00
## 156 0.00 0.00 0.00 0.00 0.00 0.00 0.00 0.00
## 157 0.00 100.00 0.00 0.00 0.00 0.00 0.00 0.00
## Prev_TvTsg Prev_TzTsg Prev_TcTvTz
## 1 0.44 0.65 0.00
## 2 0.00 0.00 0.00
## 3 0.00 0.00 0.00
## 4 0.00 0.00 0.00
## 5 0.00 0.00 0.00
## 6 0.00 1.03 0.00
## 7 0.00 0.00 0.00
## 8 0.00 0.00 0.00
## 9 0.68 0.68 0.00
## 10 0.00 0.00 0.00
## 11 0.00 0.00 0.00
## 12 0.00 0.00 0.00
## 13 0.00 0.00 0.00
## 14 0.00 0.00 0.00
## 15 0.00 0.00 0.00
## 16 0.00 0.00 0.00
## 17 0.00 0.00 0.00
## 18 0.00 0.00 0.00
## 19 0.00 2.27 0.00
## 20 0.00 0.00 0.00
## 21 0.00 0.00 0.00
## 22 6.17 1.23 0.00
## 23 1.25 0.00 0.00
## 24 0.00 0.00 1.28
## 25 0.00 0.00 0.00
## 26 0.00 0.00 0.00
## 27 0.00 0.00 0.00
## 28 0.00 0.00 0.00
## 29 0.00 0.00 0.00
## 30 0.00 0.00 0.00
## 31 0.00 1.41 0.00
## 32 0.00 0.00 0.00
## 33 0.00 0.00 0.00
## 34 0.00 0.00 0.00
## 35 0.00 0.00 0.00
## 37 0.00 0.00 0.00
## 38 0.00 0.00 0.00
## 39 0.00 0.00 0.00
## 40 0.00 0.00 0.00
## 41 0.00 0.00 0.00
## 42 0.00 0.00 0.00
## 43 0.00 0.00 5.66
## 44 0.00 0.00 0.00
## 45 9.43 3.77 0.00
## 46 0.00 0.00 0.00
## 47 0.00 0.00 0.00
## 48 0.00 0.00 0.00
## 49 0.00 0.00 0.00
## 50 0.00 0.00 0.00
## 51 0.00 0.00 0.00
## 52 2.00 0.00 0.00
## 53 0.00 0.00 0.00
## 54 0.00 0.00 0.00
## 55 0.00 0.00 0.00
## 56 0.00 0.00 0.00
## 57 0.00 0.00 0.00
## 58 0.00 0.00 0.00
## 59 0.00 0.00 0.00
## 60 0.00 0.00 0.00
## 61 0.00 0.00 0.00
## 62 0.00 0.00 0.00
## 63 0.00 0.00 0.00
## 64 0.00 0.00 0.00
## 65 0.00 0.00 0.00
## 66 2.94 0.00 0.00
## 67 0.00 0.00 0.00
## 68 0.00 0.00 0.00
## 69 0.00 0.00 0.00
## 70 0.00 0.00 0.00
## 71 0.00 0.00 0.00
## 72 0.00 0.00 3.33
## 73 0.00 0.00 3.33
## 74 0.00 0.00 0.00
## 75 0.00 0.00 0.00
## 76 0.00 0.00 0.00
## 77 0.00 0.00 0.00
## 78 0.00 0.00 0.00
## 79 0.00 0.00 0.00
## 80 7.69 0.00 0.00
## 81 0.00 0.00 0.00
## 82 0.00 0.00 0.00
## 83 0.00 0.00 0.00
## 84 0.00 0.00 0.00
## 85 0.00 0.00 0.00
## 86 0.00 0.00 0.00
## 87 0.00 0.00 0.00
## 88 4.35 8.70 0.00
## 89 0.00 0.00 0.00
## 90 0.00 0.00 0.00
## 91 31.58 0.00 0.00
## 92 0.00 0.00 0.00
## 93 0.00 0.00 0.00
## 94 0.00 0.00 0.00
## 95 0.00 0.00 0.00
## 96 0.00 0.00 0.00
## 97 0.00 0.00 0.00
## 98 0.00 0.00 0.00
## 99 0.00 0.00 0.00
## 100 0.00 0.00 0.00
## 101 0.00 0.00 0.00
## 102 0.00 0.00 0.00
## 103 0.00 0.00 0.00
## 104 15.38 0.00 0.00
## 105 0.00 0.00 0.00
## 106 0.00 0.00 0.00
## 107 0.00 0.00 0.00
## 108 16.67 0.00 0.00
## 109 0.00 0.00 0.00
## 110 0.00 0.00 0.00
## 111 0.00 0.00 0.00
## 112 0.00 0.00 0.00
## 113 0.00 0.00 0.00
## 114 11.11 0.00 0.00
## 115 0.00 0.00 0.00
## 116 0.00 0.00 0.00
## 117 0.00 0.00 0.00
## 118 0.00 0.00 0.00
## 119 0.00 0.00 0.00
## 120 0.00 0.00 0.00
## 121 0.00 0.00 0.00
## 122 0.00 0.00 0.00
## 123 0.00 0.00 0.00
## 124 28.57 0.00 0.00
## 125 0.00 0.00 0.00
## 126 0.00 0.00 0.00
## 127 0.00 0.00 0.00
## 128 0.00 0.00 0.00
## 129 0.00 0.00 0.00
## 130 0.00 0.00 0.00
## 131 0.00 0.00 0.00
## 132 0.00 0.00 0.00
## 133 0.00 0.00 0.00
## 134 0.00 0.00 0.00
## 135 0.00 0.00 0.00
## 136 0.00 0.00 0.00
## 137 20.00 0.00 0.00
## 138 0.00 0.00 0.00
## 139 0.00 0.00 0.00
## 140 0.00 0.00 0.00
## 141 0.00 0.00 0.00
## 142 0.00 0.00 0.00
## 143 0.00 0.00 0.00
## 144 0.00 0.00 0.00
## 145 66.67 33.33 0.00
## 146 33.33 0.00 0.00
## 147 33.33 33.33 0.00
## 148 0.00 0.00 0.00
## 149 0.00 0.00 0.00
## 150 0.00 0.00 0.00
## 151 0.00 0.00 0.00
## 152 0.00 0.00 0.00
## 153 0.00 0.00 0.00
## 154 0.00 0.00 0.00
## 155 0.00 0.00 0.00
## 156 0.00 0.00 0.00
## 157 0.00 0.00 0.00

## Statistics showen in the mansucript consequently

model1<-glm((Prev_Tspp) ~ Country,data=data, family=gaussian())
model2<-glm((Prev_Tspp) ~ Species,data=data, family=gaussian())
model3<-glm((Prev_Tspp) ~ Country*Species,data=data, family=gaussian())
model4<-glm((Prev_Tspp) ~ Country+Species,data=data, family=gaussian())
model5<-glm((Prev_Tspp) ~ Country*Species+Localisation,data=data, family=gaussian())
model6<-glm((Prev_Tspp) ~ Country+Species+Localisation,data=data, family=gaussian())

#AICc(model1, model2, model3, model4)
model.set <- list(model1, model2, model3, model4, model5, model6)
model.names <- c("model1", "model2","model3", "model4", "model5", "model6")

aictab(model.set, modnames = model.names)

##
## Model selection based on AICc:
##
## K AICc Delta_AICc AICcWt Cum.Wt LL
## model1 16 1521.35 0.00 0.9 0.9 -742.72
## model4 23 1525.81 4.46 0.1 1.0 -735.72
## model3 27 1534.50 13.15 0.0 1.0 -734.34
## model2 11 1545.55 24.20 0.0 1.0 -760.86
## model6 100 1571.61 50.26 0.0 1.0 -502.17
## model5 103 1614.79 93.44 0.0 1.0 -498.39

summary(model1)

##
## Call:
## glm(formula = (Prev_Tspp) ~ Country, family = gaussian(), data = data)
##
## Deviance Residuals:
## Min 1Q Median 3Q Max
## -69.739 -19.625 -2.419 17.636 75.726
##
## Coefficients:
## Estimate Std. Error t value Pr(>|t|)
## (Intercept) 35.627 4.536 7.855 9.24e-13 ***
## CountryETH -15.587 30.088 -0.518 0.605225
## CountryGHA 34.112 8.350 4.085 7.35e-05 ***
## CountryGUI -33.209 8.919 -3.723 0.000284 ***
## CountryKEN 6.128 9.711 0.631 0.529059
## CountryMLI -25.200 8.350 -3.018 0.003021 **
## CountryMOZ -27.627 21.516 -1.284 0.201227
## CountrySAF -14.495 11.453 -1.266 0.207727
## CountrySEN -11.353 8.919 -1.273 0.205148
## CountrySWA -35.627 30.088 -1.184 0.238360
## CountryUGA -26.345 14.054 -1.875 0.062918 .
## CountryURT -5.429 12.962 -0.419 0.675975
## CountryZAI -32.767 30.088 -1.089 0.277985
## CountryZAM 9.583 30.088 0.318 0.750587
## CountryZIM -11.259 10.442 -1.078 0.282769
## ---
## Signif. codes: 0 '***' 0.001 '**' 0.01 '*' 0.05 '.' 0.1 ' ' 1
##
## (Dispersion parameter for gaussian family taken to be 884.7024)
##
## Null deviance: 180975 on 155 degrees of freedom
## Residual deviance: 124743 on 141 degrees of freedom
## AIC: 1517.4
##
## Number of Fisher Scoring iterations: 2

Anova(model1)

## Analysis of Deviance Table (Type II tests)
##
## Response: (Prev_Tspp)
## LR Chisq Df Pr(>Chisq)
## Country 63.56 14 2.76e-08 ***
## ---
## Signif. codes: 0 '***' 0.001 '**' 0.01 '*' 0.05 '.' 0.1 ' ' 1

Anova(model2)

## Analysis of Deviance Table (Type II tests)
##
## Response: (Prev_Tspp)
## LR Chisq Df Pr(>Chisq)
## Species 21.863 9 0.009324 **
## ---
## Signif. codes: 0 '***' 0.001 '**' 0.01 '*' 0.05 '.' 0.1 ' ' 1

#-----------------------------------------------------------------------
#impact of taxa and countries

# statistics in the manuscript
data_ga <- subset(data, Species=="Ga")
data_ga

## Country Localisation Species Sex Sample Prev_Sod Prev_Tspp
## 27 SAF False Bay Park Ga NI 27 0.00 2.60
## 41 SAF SAFint Lucia Ga NI 41 0.00 38.60
## 46 SAF Lower Mkhuze Ga NI 46 0.00 43.40
## 53 MOZ Reserva Especial de Maputo Ga NI 53 0.00 10.00
## 59 SAF North eastern KwaZulu-Natal Ga NI 59 5.13 5.13
## 75 SWA Mlawula Nature Reserve Ga NI 75 0.00 0.00
## 76 URT Uguja island Ga NI 76 6.67 10.00
## 132 URT Zanzibar Ga NI 132 16.67 0.00
## 141 URT Jozani Ga NI 141 0.00 25.00
## Prev_Tc Prev_Tv Prev_Tz Prev_Tsg Prev_TcTv Prev_TcTz Prev_TcTsg Prev_TvTz
## 27 2.60 0.00 0.00 0.00 0.00 0 0.00 0
## 41 10.53 0.00 10.53 15.79 0.00 0 1.75 0
## 46 9.43 1.89 3.77 24.53 1.89 0 1.89 0
## 53 6.00 0.00 0.00 4.00 0.00 0 0.00 0
## 59 2.56 0.00 0.00 2.56 0.00 0 0.00 0
## 75 0.00 0.00 0.00 0.00 0.00 0 0.00 0
## 76 0.00 6.67 3.33 0.00 0.00 0 0.00 0
## 132 0.00 0.00 0.00 0.00 0.00 0 0.00 0
## 141 0.00 0.00 25.00 0.00 0.00 0 0.00 0
## Prev_TvTsg Prev_TzTsg Prev_TcTvTz
## 27 0 0 0
## 41 0 0 0
## 46 0 0 0
## 53 0 0 0
## 59 0 0 0
## 75 0 0 0
## 76 0 0 0
## 132 0 0 0
## 141 0 0 0

model1<-glm((Prev_Tspp) ~ Country,data=data_ga, family=gaussian())
summary(model1)

##
## Call:
## glm(formula = (Prev_Tspp) ~ Country, family = gaussian(), data = data_ga)
##
## Deviance Residuals:
## 27 41 46 53 59 75 76 132
## -19.832 16.168 20.968 0.000 -17.302 0.000 -1.667 -11.667
## 141
## 13.333
##
## Coefficients:
## Estimate Std. Error t value Pr(>|t|)
## (Intercept) 10.000 18.495 0.541 0.612
## CountrySAF 12.433 20.678 0.601 0.574
## CountrySWA -10.000 26.156 -0.382 0.718
## CountryURT 1.667 21.357 0.078 0.941
##
## (Dispersion parameter for gaussian family taken to be 342.0791)
##
## Null deviance: 2214.7 on 8 degrees of freedom
## Residual deviance: 1710.4 on 5 degrees of freedom
## AIC: 82.766
##
## Number of Fisher Scoring iterations: 2

Anova(model1)

## Analysis of Deviance Table (Type II tests)
##
## Response: (Prev_Tspp)
## LR Chisq Df Pr(>Chisq)
## Country 1.4742 3 0.6882

#----------------------------------------------
data_gb <- subset(data, Species=="Gb")
data_gb

## Country Localisation Species Sex Sample Prev_Sod Prev_Tspp
## 7 SAF Phinda Gb NI 7 4.12 0.00
## 50 SAF Hluhluwe Gb NI 50 0.00 32.00
## 51 SAF North eastern KwaZulu-Natal Gb NI 51 0.00 4.00
## 52 MOZ Reserva Especial de Maputo Gb NI 52 14.00 6.00
## 74 SAF SAFint Lucia Gb NI 74 0.00 43.33
## Prev_Tc Prev_Tv Prev_Tz Prev_Tsg Prev_TcTv Prev_TcTz Prev_TcTsg Prev_TvTz
## 7 0 0.00 0.00 0.00 0 0 0 0
## 50 12 2.00 14.00 4.00 0 0 0 0
## 51 2 0.00 0.00 2.00 0 0 0 0
## 52 0 2.00 0.00 2.00 0 0 0 0
## 74 0 3.33 3.33 36.67 0 0 0 0
## Prev_TvTsg Prev_TzTsg Prev_TcTvTz
## 7 0 0 0
## 50 0 0 0
## 51 0 0 0
## 52 2 0 0
## 74 0 0 0

model1<-glm((Prev_Tspp) ~ Country,data=data_gb, family=gaussian())
summary(model1)

##
## Call:
## glm(formula = (Prev_Tspp) ~ Country, family = gaussian(), data = data_gb)
##
## Deviance Residuals:
## 7 50 51 52 74
## -19.83 12.17 -15.83 0.00 23.50
##
## Coefficients:
## Estimate Std. Error t value Pr(>|t|)
## (Intercept) 6.00 21.17 0.283 0.795
## CountrySAF 13.83 23.67 0.584 0.600
##
## (Dispersion parameter for gaussian family taken to be 448.0589)
##
## Null deviance: 1497.2 on 4 degrees of freedom
## Residual deviance: 1344.2 on 3 degrees of freedom
## AIC: 48.16
##
## Number of Fisher Scoring iterations: 2

Anova(model1)

## Analysis of Deviance Table (Type II tests)
##
## Response: (Prev_Tspp)
## LR Chisq Df Pr(>Chisq)
## Country 0.34163 1 0.5589

#-------------------------------------------
data_gff <- subset(data, Species=="Gff")
data_gff

## Country Localisation Species Sex Sample Prev_Sod Prev_Tspp Prev_Tc Prev_Tv
## 16 UGA Buvuma island Gff NI 16 4.26 10.64 3.19 2.13
## 49 KEN Ikapolok Gff NI 49 39.22 37.25 1.96 9.80
## 61 KEN Obekai Gff NI 61 0.00 5.26 2.63 2.63
## Prev_Tz Prev_Tsg Prev_TcTv Prev_TcTz Prev_TcTsg Prev_TvTz Prev_TvTsg
## 16 1.06 3.19 0.00 0.00 1.06 0 0
## 49 0.00 19.61 3.92 1.96 0.00 0 0
## 61 0.00 0.00 0.00 0.00 0.00 0 0
## Prev_TzTsg Prev_TcTvTz
## 16 0 0
## 49 0 0
## 61 0 0

model1<-glm((Prev_Tspp) ~ Country,data=data_gff, family=gaussian())
summary(model1)

##
## Call:
## glm(formula = (Prev_Tspp) ~ Country, family = gaussian(), data = data_gff)
##
## Deviance Residuals:
## 16 49 61
## 0.00 15.99 -15.99
##
## Coefficients:
## Estimate Std. Error t value Pr(>|t|)
## (Intercept) 21.26 16.00 1.329 0.411
## CountryUGA -10.61 27.70 -0.383 0.767
##
## (Dispersion parameter for gaussian family taken to be 511.68)
##
## Null deviance: 586.80 on 2 degrees of freedom
## Residual deviance: 511.68 on 1 degrees of freedom
## AIC: 29.931
##
## Number of Fisher Scoring iterations: 2

Anova(model1)

## Analysis of Deviance Table (Type II tests)
##
## Response: (Prev_Tspp)
## LR Chisq Df Pr(>Chisq)
## Country 0.14681 1 0.7016

#-------------------------------------------
data_gmm <- subset(data, Species=="Gmm")
data_gmm

## Country Localisation Species Sex Sample Prev_Sod Prev_Tspp Prev_Tc Prev_Tv
## 21 KEN Kari Gmm NI 21 63.53 2.35 2.35 0.00
## 22 URT MaSAFng-URTga Gmm NI 22 76.54 53.09 18.52 1.23
## 26 ZIM Makuti Gmm NI 26 26.92 91.03 11.54 0.00
## 93 ZIM Kemukura Gmm NI 93 22.22 5.56 0.00 5.56
## 100 ZIM Rukomeshi Gmm NI 100 20.00 0.00 0.00 0.00
## 105 ZIM Mukondore Gmm NI 105 23.08 7.69 7.69 0.00
## 115 ZIM M. chiuyi Gmm NI 115 11.11 0.00 0.00 0.00
## 131 ZIM Mushumb Gmm NI 131 0.00 33.33 16.67 0.00
## Prev_Tz Prev_Tsg Prev_TcTv Prev_TcTz Prev_TcTsg Prev_TvTz Prev_TvTsg
## 21 0.00 0.00 0 0 0.00 0 0.00
## 22 1.23 18.52 0 0 6.17 0 6.17
## 26 2.56 69.23 0 0 7.69 0 0.00
## 93 0.00 0.00 0 0 0.00 0 0.00
## 100 0.00 0.00 0 0 0.00 0 0.00
## 105 0.00 0.00 0 0 0.00 0 0.00
## 115 0.00 0.00 0 0 0.00 0 0.00
## 131 0.00 16.67 0 0 0.00 0 0.00
## Prev_TzTsg Prev_TcTvTz
## 21 0.00 0
## 22 1.23 0
## 26 0.00 0
## 93 0.00 0
## 100 0.00 0
## 105 0.00 0
## 115 0.00 0
## 131 0.00 0

model1<-glm((Prev_Tspp) ~ Country,data=data_gmm, family=gaussian())
summary(model1)

##
## Call:
## glm(formula = (Prev_Tspp) ~ Country, family = gaussian(), data = data_gmm)
##
## Deviance Residuals:
## 21 22 26 93 100 105 115 131
## 0.00 0.00 68.09 -17.38 -22.93 -15.24 -22.93 10.39
##
## Coefficients:
## Estimate Std. Error t value Pr(>|t|)
## (Intercept) 2.35 35.59 0.066 0.950
## CountryURT 50.74 50.32 1.008 0.360
## CountryZIM 20.59 38.44 0.536 0.615
##
## (Dispersion parameter for gaussian family taken to be 1266.263)
##
## Null deviance: 7652.9 on 7 degrees of freedom
## Residual deviance: 6331.3 on 5 degrees of freedom
## AIC: 84.094
##
## Number of Fisher Scoring iterations: 2

Anova(model1)

## Analysis of Deviance Table (Type II tests)
##
## Response: (Prev_Tspp)
## LR Chisq Df Pr(>Chisq)
## Country 1.0437 2 0.5934

#-----------------------------------------------
data_gp <- subset(data, Species=="Gp")
data_gp

## Country Localisation Species Sex Sample Prev_Sod Prev_Tspp Prev_Tc
## 1 ETH Arba minch, nech SAFr Gp NI 1 20.48 20.04 6.10
## 5 KEN Katotoi Gp NI 5 42.56 88.72 21.54
## 6 KEN Mwea Gp NI 6 3.08 19.49 7.18
## 8 ZAM Mfuwe Gp NI 8 2.05 45.21 1.37
## 9 URT URTzania Gp NI 9 78.77 19.86 6.16
## 17 KEN Kari Gp NI 17 89.36 2.13 1.06
## 19 KEN Koibos Gp NI 19 0.00 71.59 15.91
## 20 KEN Meru nat. parc Gp NI 20 22.99 70.11 29.89
## 23 KEN Ruma nat. parc Gp NI 23 26.25 17.50 10.00
## 31 URT MaSAFng-URTga Gp NI 31 66.20 73.24 33.80
## 38 KEN Emsos Gp NI 38 0.00 93.33 15.00
## 42 UGA Budaka Gp NI 42 94.55 7.27 1.82
## 60 ZIM Makuti Gp NI 60 15.79 86.84 7.89
## 79 UGA Omugo Gp NI 79 100.00 0.00 0.00
## 81 ZIM Mushumb Gp NI 81 3.85 19.23 3.85
## 85 UGA Lira Gp NI 85 0.00 16.00 8.00
## 90 KEN Kiria Gp NI 90 0.00 80.00 55.00
## 101 KEN Mwea nat. parc Gp NI 101 0.00 13.33 13.33
## 118 UGA Moyo Gp NI 118 87.50 12.50 0.00
## 139 ZIM Rukomeshi Gp NI 139 0.00 0.00 0.00
## 140 ZIM Gokwe Gp NI 140 0.00 0.00 0.00
## Prev_Tv Prev_Tz Prev_Tsg Prev_TcTv Prev_TcTz Prev_TcTsg Prev_TvTz
## 1 0.65 1.09 10.46 0.00 0.00 0.65 0.00
## 5 15.38 0.51 26.15 6.67 1.54 15.90 1.03
## 6 0.51 1.03 7.69 1.03 0.51 0.51 0.00
## 8 0.68 0.68 34.93 0.00 0.00 7.53 0.00
## 9 2.74 0.68 8.22 0.00 0.00 0.68 0.00
## 17 0.00 1.06 0.00 0.00 0.00 0.00 0.00
## 19 9.09 0.00 28.41 0.00 0.00 15.91 0.00
## 20 4.60 0.00 25.29 0.00 0.00 10.34 0.00
## 23 2.50 2.50 0.00 1.25 0.00 0.00 0.00
## 31 0.00 0.00 14.08 0.00 1.41 21.13 1.41
## 38 8.33 0.00 58.33 1.67 0.00 10.00 0.00
## 42 0.00 0.00 5.45 0.00 0.00 0.00 0.00
## 60 0.00 0.00 73.68 0.00 0.00 5.26 0.00
## 79 0.00 0.00 0.00 0.00 0.00 0.00 0.00
## 81 0.00 7.69 3.85 0.00 0.00 3.85 0.00
## 85 0.00 4.00 4.00 0.00 0.00 0.00 0.00
## 90 5.00 0.00 10.00 10.00 0.00 0.00 0.00
## 101 0.00 0.00 0.00 0.00 0.00 0.00 0.00
## 118 0.00 0.00 12.50 0.00 0.00 0.00 0.00
## 139 0.00 0.00 0.00 0.00 0.00 0.00 0.00
## 140 0.00 0.00 0.00 0.00 0.00 0.00 0.00
## Prev_TvTsg Prev_TzTsg Prev_TcTvTz
## 1 0.44 0.65 0
## 5 0.00 0.00 0
## 6 0.00 1.03 0
## 8 0.00 0.00 0
## 9 0.68 0.68 0
## 17 0.00 0.00 0
## 19 0.00 2.27 0
## 20 0.00 0.00 0
## 23 1.25 0.00 0
## 31 0.00 1.41 0
## 38 0.00 0.00 0
## 42 0.00 0.00 0
## 60 0.00 0.00 0
## 79 0.00 0.00 0
## 81 0.00 0.00 0
## 85 0.00 0.00 0
## 90 0.00 0.00 0
## 101 0.00 0.00 0
## 118 0.00 0.00 0
## 139 0.00 0.00 0
## 140 0.00 0.00 0

model1<-glm((Prev_Tspp) ~ Country,data=data_gp, family=gaussian())
summary(model1)

##
## Call:
## glm(formula = (Prev_Tspp) ~ Country, family = gaussian(), data = data_gp)
##
## Deviance Residuals:
## Min 1Q Median 3Q Max
## -48.56 -26.52 0.00 20.90 60.32
##
## Coefficients:
## Estimate Std. Error t value Pr(>|t|)
## (Intercept) 20.040 34.093 0.588 0.565
## CountryKEN 30.649 35.937 0.853 0.407
## CountryUGA -11.097 38.117 -0.291 0.775
## CountryURT 26.510 41.755 0.635 0.535
## CountryZAM 25.170 48.214 0.522 0.609
## CountryZIM 6.478 38.117 0.170 0.867
##
## (Dispersion parameter for gaussian family taken to be 1162.303)
##
## Null deviance: 23227 on 20 degrees of freedom
## Residual deviance: 17435 on 15 degrees of freedom
## AIC: 214.75
##
## Number of Fisher Scoring iterations: 2

Anova(model1)

## Analysis of Deviance Table (Type II tests)
##
## Response: (Prev_Tspp)
## LR Chisq Df Pr(>Chisq)
## Country 4.9833 5 0.4179

#-----------------------------------------------
data_gpg <- subset(data, Species=="Gpg")
data_gpg

## Country Localisation Species Sex Sample Prev_Sod Prev_Tspp Prev_Tc
## 4 SEN Pout Gpg NI 4 0.00 7.04 1.51
## 12 BKF Folonzo Gpg F 12 0.00 7.55 0.94
## 13 BKF Folonzo Gpg M 13 1.89 16.04 0.00
## 15 GUI Kangoliya Gpg F 15 95.74 0.00 0.00
## 18 SEN Kayar Gpg NI 18 0.00 1.14 0.00
## 25 BKF Moussodougou Gpg F 25 0.00 44.87 0.00
## 30 BKF Comoe Gpg F 30 0.00 2.82 1.41
## 32 BKF Kartasso Gpg F 32 0.00 0.00 0.00
## 33 BKF Kartasso Gpg M 33 0.00 0.00 0.00
## 34 SEN DiackSAFo Peulh Gpg NI 34 0.00 7.69 0.00
## 35 BKF Moussodougou Gpg M 35 0.00 21.88 0.00
## 40 MLI Bani Gpg F 40 0.00 1.72 0.00
## 45 BKF Kampty Gpg F 45 0.00 90.57 1.89
## 47 BKF Comoe Gpg M 47 0.00 1.92 1.92
## 48 MLI SEN Gpg M 48 0.00 7.69 1.92
## 54 BKF Bama Gpg F 54 0.00 0.00 0.00
## 56 SEN Tambacounda Gpg F 56 0.00 41.46 0.00
## 57 SEN Tambacounda Gpg M 57 0.00 71.79 0.00
## 58 SEN SebikoURTe Gpg NI 58 0.00 5.13 0.00
## 62 MLI SEN Gpg F 62 0.00 0.00 0.00
## 64 BKF Bama Gpg M 64 0.00 0.00 0.00
## 65 MLI Banco Gpg F 65 0.00 20.59 0.00
## 66 BKF Dedougou Gpg F 66 0.00 52.94 0.00
## 67 MLI Sikasso Gpg F 67 0.00 6.06 0.00
## 71 GUI Kangoliya Gpg M 71 0.00 0.00 0.00
## 77 GUI Mini Gpg F 77 0.00 3.45 0.00
## 78 SEN Hann Gpg NI 78 0.00 0.00 0.00
## 80 BKF Kampty Gpg M 80 0.00 80.77 0.00
## 82 GUI Kifala Gpg M 82 0.00 0.00 0.00
## 83 MLI Sikasso Gpg M 83 0.00 0.00 0.00
## 84 MLI SS Gpg F 84 0.00 4.00 0.00
## 86 BKF Kenedougou Gpg F 86 0.00 0.00 0.00
## 87 MLI SS Gpg M 87 0.00 0.00 0.00
## 88 BKF Dedougou Gpg M 88 0.00 69.57 4.35
## 89 GUI Bafing Gpg F 89 0.00 5.00 0.00
## 91 BKF Kampty Gpg NI 91 0.00 84.21 0.00
## 92 GUI Tinkisso Gpg M 92 0.00 5.56 0.00
## 94 BKF Kenedougou Gpg M 94 0.00 0.00 0.00
## 96 GUI Dekonkore Gpg F 96 0.00 6.25 0.00
## 97 GUI Mini Gpg M 97 0.00 0.00 0.00
## 98 SEN Fleuve Gambie Gpg M 98 0.00 43.75 0.00
## 102 GUI Bafing Gpg M 102 0.00 0.00 0.00
## 103 GUI Tinkisso Gpg F 103 0.00 7.69 0.00
## 104 BKF Bouroum bouroum Gpg F 104 0.00 92.31 0.00
## 106 GUI Karifale Gpg M 106 0.00 8.33 0.00
## 107 GUI Lemonako Gpg F 107 0.00 0.00 0.00
## 108 BKF KouriGUIon Gpg F 108 0.00 50.00 0.00
## 109 MLI Bani Gpg M 109 0.00 0.00 0.00
## 110 MLI Sybi Gpg F 110 0.00 0.00 0.00
## 111 MLI Sybi Gpg M 111 0.00 0.00 0.00
## 113 SEN Fleueve G Gpg F 113 0.00 11.11 0.00
## 114 BKF KouriGUIon Gpg NI 114 0.00 22.22 0.00
## 117 GUI Lemonako Gpg M 117 0.00 0.00 0.00
## 119 SEN Diaguiri Gpg F 119 0.00 0.00 0.00
## 120 MLI Banco Gpg M 120 0.00 28.57 0.00
## 121 MLI Baoule Gpg F 121 0.00 42.86 0.00
## 122 MLI Baoule Gpg M 122 0.00 42.86 0.00
## 123 MLI Bougouni Gpg M 123 0.00 0.00 0.00
## 124 BKF Lorepeni Gpg F 124 0.00 71.43 0.00
## 127 SEN MousSAFlla Gpg M 127 0.00 0.00 0.00
## 128 MLI Baguineda Gpg F 128 0.00 16.67 0.00
## 129 MLI Bougouni Gpg F 129 0.00 0.00 0.00
## 130 MLI Kita Gpg M 130 0.00 16.67 0.00
## 136 GUI Kifala Gpg F 136 0.00 0.00 0.00
## 137 BKF Bouroum bouroum Gpg M 137 0.00 80.00 0.00
## 138 SEN Fleuve Gambie Gpg F 138 0.00 25.00 0.00
## 143 SEN Niokolo Gpg M 143 0.00 0.00 0.00
## 145 BKF Lorepeni Gpg M 145 0.00 100.00 0.00
## 146 BKF KouriGUIon Gpg M 146 0.00 66.67 0.00
## 147 BKF Ouarkoye Gpg M 147 0.00 100.00 0.00
## 150 SEN Diaguiri Gpg M 150 0.00 50.00 0.00
## 151 BKF Ouarkoye Gpg F 151 0.00 100.00 50.00
## 156 GUI Karifale Gpg F 156 0.00 0.00 0.00
## 157 SEN Mako Gpg M 157 0.00 100.00 0.00
## Prev_Tv Prev_Tz Prev_Tsg Prev_TcTv Prev_TcTz Prev_TcTsg Prev_TvTz
## 4 2.01 0.50 3.02 0 0.00 0.00 0.00
## 12 2.83 1.89 0.00 0 0.00 0.00 1.89
## 13 5.66 5.66 0.00 0 1.89 0.00 2.83
## 15 0.00 0.00 0.00 0 0.00 0.00 0.00
## 18 0.00 0.00 1.14 0 0.00 0.00 0.00
## 25 20.51 12.82 0.00 0 0.00 0.00 11.54
## 30 1.41 0.00 0.00 0 0.00 0.00 0.00
## 32 0.00 0.00 0.00 0 0.00 0.00 0.00
## 33 0.00 0.00 0.00 0 0.00 0.00 0.00
## 34 1.54 1.54 4.62 0 0.00 0.00 0.00
## 35 6.25 15.63 0.00 0 0.00 0.00 0.00
## 40 1.72 0.00 0.00 0 0.00 0.00 0.00
## 45 62.26 1.89 11.32 0 0.00 0.00 0.00
## 47 0.00 0.00 0.00 0 0.00 0.00 0.00
## 48 1.92 3.85 0.00 0 0.00 0.00 0.00
## 54 0.00 0.00 0.00 0 0.00 0.00 0.00
## 56 26.83 7.32 0.00 0 0.00 0.00 7.32
## 57 53.85 2.56 0.00 0 0.00 0.00 15.38
## 58 5.13 0.00 0.00 0 0.00 0.00 0.00
## 62 0.00 0.00 0.00 0 0.00 0.00 0.00
## 64 0.00 0.00 0.00 0 0.00 0.00 0.00
## 65 20.59 0.00 0.00 0 0.00 0.00 0.00
## 66 26.47 0.00 23.53 0 0.00 0.00 0.00
## 67 0.00 6.06 0.00 0 0.00 0.00 0.00
## 71 0.00 0.00 0.00 0 0.00 0.00 0.00
## 77 3.45 0.00 0.00 0 0.00 0.00 0.00
## 78 0.00 0.00 0.00 0 0.00 0.00 0.00
## 80 65.38 0.00 7.69 0 0.00 0.00 0.00
## 82 0.00 0.00 0.00 0 0.00 0.00 0.00
## 83 0.00 0.00 0.00 0 0.00 0.00 0.00
## 84 4.00 0.00 0.00 0 0.00 0.00 0.00
## 86 0.00 0.00 0.00 0 0.00 0.00 0.00
## 87 0.00 0.00 0.00 0 0.00 0.00 0.00
## 88 30.43 13.04 4.35 0 0.00 4.35 0.00
## 89 5.00 0.00 0.00 0 0.00 0.00 0.00
## 91 26.32 5.26 21.05 0 0.00 0.00 0.00
## 92 5.56 0.00 0.00 0 0.00 0.00 0.00
## 94 0.00 0.00 0.00 0 0.00 0.00 0.00
## 96 6.25 0.00 0.00 0 0.00 0.00 0.00
## 97 0.00 0.00 0.00 0 0.00 0.00 0.00
## 98 43.75 0.00 0.00 0 0.00 0.00 0.00
## 102 0.00 0.00 0.00 0 0.00 0.00 0.00
## 103 7.69 0.00 0.00 0 0.00 0.00 0.00
## 104 53.85 0.00 23.08 0 0.00 0.00 0.00
## 106 8.33 0.00 0.00 0 0.00 0.00 0.00
## 107 8.33 0.00 0.00 0 0.00 0.00 0.00
## 108 0.00 0.00 33.33 0 0.00 0.00 0.00
## 109 0.00 0.00 0.00 0 0.00 0.00 0.00
## 110 0.00 0.00 0.00 0 0.00 0.00 0.00
## 111 0.00 0.00 0.00 0 0.00 0.00 0.00
## 113 11.11 0.00 0.00 0 0.00 0.00 0.00
## 114 0.00 0.00 11.11 0 0.00 0.00 0.00
## 117 0.00 0.00 0.00 0 0.00 0.00 0.00
## 119 0.00 0.00 0.00 0 0.00 0.00 0.00
## 120 28.57 0.00 0.00 0 0.00 0.00 0.00
## 121 42.86 0.00 0.00 0 0.00 0.00 0.00
## 122 42.86 0.00 0.00 0 0.00 0.00 0.00
## 123 0.00 0.00 0.00 0 0.00 0.00 0.00
## 124 14.29 0.00 28.57 0 0.00 0.00 0.00
## 127 0.00 0.00 0.00 0 0.00 0.00 0.00
## 128 16.67 0.00 0.00 0 0.00 0.00 0.00
## 129 0.00 0.00 0.00 0 0.00 0.00 0.00
## 130 16.67 0.00 0.00 0 0.00 0.00 0.00
## 136 0.00 0.00 0.00 0 0.00 0.00 0.00
## 137 40.00 0.00 20.00 0 0.00 0.00 0.00
## 138 25.00 0.00 0.00 0 0.00 0.00 0.00
## 143 66.67 0.00 0.00 0 0.00 0.00 0.00
## 145 0.00 0.00 0.00 0 0.00 0.00 0.00
## 146 33.33 0.00 0.00 0 0.00 0.00 0.00
## 147 33.33 0.00 0.00 0 0.00 0.00 0.00
## 150 0.00 50.00 0.00 0 0.00 0.00 0.00
## 151 50.00 0.00 0.00 0 0.00 0.00 0.00
## 156 0.00 0.00 0.00 0 0.00 0.00 0.00
## 157 100.00 0.00 0.00 0 0.00 0.00 0.00
## Prev_TvTsg Prev_TzTsg Prev_TcTvTz
## 4 0.00 0.00 0
## 12 0.00 0.00 0
## 13 0.00 0.00 0
## 15 0.00 0.00 0
## 18 0.00 0.00 0
## 25 0.00 0.00 0
## 30 0.00 0.00 0
## 32 0.00 0.00 0
## 33 0.00 0.00 0
## 34 0.00 0.00 0
## 35 0.00 0.00 0
## 40 0.00 0.00 0
## 45 9.43 3.77 0
## 47 0.00 0.00 0
## 48 0.00 0.00 0
## 54 0.00 0.00 0
## 56 0.00 0.00 0
## 57 0.00 0.00 0
## 58 0.00 0.00 0
## 62 0.00 0.00 0
## 64 0.00 0.00 0
## 65 0.00 0.00 0
## 66 2.94 0.00 0
## 67 0.00 0.00 0
## 71 0.00 0.00 0
## 77 0.00 0.00 0
## 78 0.00 0.00 0
## 80 7.69 0.00 0
## 82 0.00 0.00 0
## 83 0.00 0.00 0
## 84 0.00 0.00 0
## 86 0.00 0.00 0
## 87 0.00 0.00 0
## 88 4.35 8.70 0
## 89 0.00 0.00 0
## 91 31.58 0.00 0
## 92 0.00 0.00 0
## 94 0.00 0.00 0
## 96 0.00 0.00 0
## 97 0.00 0.00 0
## 98 0.00 0.00 0
## 102 0.00 0.00 0
## 103 0.00 0.00 0
## 104 15.38 0.00 0
## 106 0.00 0.00 0
## 107 0.00 0.00 0
## 108 16.67 0.00 0
## 109 0.00 0.00 0
## 110 0.00 0.00 0
## 111 0.00 0.00 0
## 113 0.00 0.00 0
## 114 11.11 0.00 0
## 117 0.00 0.00 0
## 119 0.00 0.00 0
## 120 0.00 0.00 0
## 121 0.00 0.00 0
## 122 0.00 0.00 0
## 123 0.00 0.00 0
## 124 28.57 0.00 0
## 127 0.00 0.00 0
## 128 0.00 0.00 0
## 129 0.00 0.00 0
## 130 0.00 0.00 0
## 136 0.00 0.00 0
## 137 20.00 0.00 0
## 138 0.00 0.00 0
## 143 0.00 0.00 0
## 145 66.67 33.33 0
## 146 33.33 0.00 0
## 147 33.33 33.33 0
## 150 0.00 0.00 0
## 151 0.00 0.00 0
## 156 0.00 0.00 0
## 157 0.00 0.00 0

model1<-glm((Prev_Tspp) ~ Country,data=data_gpg, family=gaussian())
summary(model1)

##
## Call:
## glm(formula = (Prev_Tspp) ~ Country, family = gaussian(), data = data_gpg)
##
## Deviance Residuals:
## Min 1Q Median 3Q Max
## -44.453 -17.072 -2.419 15.430 75.726
##
## Coefficients:
## Estimate Std. Error t value Pr(>|t|)
## (Intercept) 44.453 5.521 8.052 1.47e-11 ***
## CountryGUI -42.034 9.127 -4.605 1.79e-05 ***
## CountryMLI -34.025 8.632 -3.942 0.000189 ***
## CountrySEN -20.179 9.127 -2.211 0.030320 *
## ---
## Signif. codes: 0 '***' 0.001 '**' 0.01 '*' 0.05 '.' 0.1 ' ' 1
##
## (Dispersion parameter for gaussian family taken to be 792.4573)
##
## Null deviance: 76638 on 73 degrees of freedom
## Residual deviance: 55472 on 70 degrees of freedom
## AIC: 709.85
##
## Number of Fisher Scoring iterations: 2

Anova(model1)

## Analysis of Deviance Table (Type II tests)
##
## Response: (Prev_Tspp)
## LR Chisq Df Pr(>Chisq)
## Country 26.709 3 6.776e-06 ***
## ---
## Signif. codes: 0 '***' 0.001 '**' 0.01 '*' 0.05 '.' 0.1 ' ' 1

#-----------------------------------------------
data_gt <- subset(data, Species=="Gt")
data_gt

## Country Localisation Species Sex Sample Prev_Sod Prev_Tspp Prev_Tc Prev_Tv
## 2 BKF Comoe Gt F 2 0 16.54 1.10 13.24
## 3 BKF Comoe Gt M 3 0 15.00 3.64 11.36
## 11 BKF Folonzo Gt F 11 0 18.87 1.89 12.26
## 14 BKF Folonzo Gt M 14 0 32.63 2.11 27.37
## 24 GHA Walewale Gt M 24 0 53.85 3.85 12.82
## 43 GHA Walewale Gt F 43 0 66.04 1.89 9.43
## 44 BKF Arly Gt F 44 0 1.89 0.00 0.00
## 55 BKF Arly Gt M 55 0 0.00 0.00 0.00
## 69 GHA MorURTi Gt M 69 0 50.00 0.00 15.63
## 70 BKF Sissili Gt M 70 0 25.00 12.50 12.50
## 95 GHA Bougouhiya Gt F 95 0 18.75 0.00 6.25
## 99 BKF Sissili Gt F 99 0 13.33 6.67 6.67
## 112 GHA MorURTi Gt F 112 0 66.67 0.00 0.00
## 116 GHA Fumbissi Gt F 116 0 100.00 0.00 0.00
## 125 GHA Fumbissi Gt M 125 0 100.00 0.00 66.67
## 126 GHA Grogro Gt M 126 0 100.00 0.00 0.00
## 133 GHA Grogro Gt F 133 0 100.00 0.00 80.00
## 134 GHA Kumpole Gt F 134 0 100.00 0.00 40.00
## 135 GHA Sissili Bidge Gt F 135 0 100.00 0.00 20.00
## 142 GHA Bougouhiya Gt M 142 0 0.00 0.00 0.00
## 148 GHA Kumpole Gt M 148 0 100.00 0.00 50.00
## 149 GHA Psikp_ Gt M 149 0 100.00 0.00 0.00
## 152 GHA Kandiaga Gt M 152 0 100.00 0.00 0.00
## 153 GHA Sissili Bidge Gt M 153 0 100.00 0.00 0.00
## 154 GHA Nabogo Gt F 154 0 0.00 0.00 0.00
## 155 GHA Volta Blanche Gt F 155 0 0.00 0.00 0.00
## Prev_Tz Prev_Tsg Prev_TcTv Prev_TcTz Prev_TcTsg Prev_TvTz Prev_TvTsg
## 2 0.37 0 0.74 1.10 0 0.00 0
## 3 0.00 0 0.00 0.00 0 0.00 0
## 11 1.89 0 1.89 0.94 0 0.00 0
## 14 1.05 0 0.00 1.05 0 1.05 0
## 24 8.97 0 0.00 10.26 0 14.10 0
## 43 24.53 0 0.00 16.98 0 7.55 0
## 44 1.89 0 0.00 0.00 0 0.00 0
## 55 0.00 0 0.00 0.00 0 0.00 0
## 69 15.63 0 0.00 18.75 0 0.00 0
## 70 0.00 0 0.00 0.00 0 0.00 0
## 95 0.00 0 0.00 0.00 0 12.50 0
## 99 0.00 0 0.00 0.00 0 0.00 0
## 112 33.33 0 0.00 22.22 0 11.11 0
## 116 37.50 0 0.00 0.00 0 62.50 0
## 125 33.33 0 0.00 0.00 0 0.00 0
## 126 16.67 0 0.00 0.00 0 83.33 0
## 133 20.00 0 0.00 0.00 0 0.00 0
## 134 60.00 0 0.00 0.00 0 0.00 0
## 135 80.00 0 0.00 0.00 0 0.00 0
## 142 0.00 0 0.00 0.00 0 0.00 0
## 148 50.00 0 0.00 0.00 0 0.00 0
## 149 50.00 0 0.00 0.00 0 50.00 0
## 152 100.00 0 0.00 0.00 0 0.00 0
## 153 100.00 0 0.00 0.00 0 0.00 0
## 154 0.00 0 0.00 0.00 0 0.00 0
## 155 0.00 0 0.00 0.00 0 0.00 0
## Prev_TzTsg Prev_TcTvTz
## 2 0 0.00
## 3 0 0.00
## 11 0 0.00
## 14 0 0.00
## 24 0 1.28
## 43 0 5.66
## 44 0 0.00
## 55 0 0.00
## 69 0 0.00
## 70 0 0.00
## 95 0 0.00
## 99 0 0.00
## 112 0 0.00
## 116 0 0.00
## 125 0 0.00
## 126 0 0.00
## 133 0 0.00
## 134 0 0.00
## 135 0 0.00
## 142 0 0.00
## 148 0 0.00
## 149 0 0.00
## 152 0 0.00
## 153 0 0.00
## 154 0 0.00
## 155 0 0.00

model1<-glm((Prev_Tspp) ~ Country,data=data_gt, family=gaussian())
summary(model1)

##
## Call:
## glm(formula = (Prev_Tspp) ~ Country, family = gaussian(), data = data_gt)
##
## Deviance Residuals:
## Min 1Q Median 3Q Max
## -69.739 -14.935 2.298 30.261 30.261
##
## Coefficients:
## Estimate Std. Error t value Pr(>|t|)
## (Intercept) 15.41 12.04 1.280 0.212940
## CountryGHA 54.33 14.47 3.754 0.000978 ***
## ---
## Signif. codes: 0 '***' 0.001 '**' 0.01 '*' 0.05 '.' 0.1 ' ' 1
##
## (Dispersion parameter for gaussian family taken to be 1159.976)
##
## Null deviance: 44189 on 25 degrees of freedom
## Residual deviance: 27839 on 24 degrees of freedom
## AIC: 261.16
##
## Number of Fisher Scoring iterations: 2

Anova(model1)

## Analysis of Deviance Table (Type II tests)
##
## Response: (Prev_Tspp)
## LR Chisq Df Pr(>Chisq)
## Country 14.095 1 0.0001738 ***
## ---
## Signif. codes: 0 '***' 0.001 '**' 0.01 '*' 0.05 '.' 0.1 ' ' 1

#------------------------------------------------------
# all species

model1<-glm((Prev_Tspp) ~ Country,data=data, family=gaussian())
summary(model1)

##
## Call:
## glm(formula = (Prev_Tspp) ~ Country, family = gaussian(), data = data)
##
## Deviance Residuals:
## Min 1Q Median 3Q Max
## -69.739 -19.625 -2.419 17.636 75.726
##
## Coefficients:
## Estimate Std. Error t value Pr(>|t|)
## (Intercept) 35.627 4.536 7.855 9.24e-13 ***
## CountryETH -15.587 30.088 -0.518 0.605225
## CountryGHA 34.112 8.350 4.085 7.35e-05 ***
## CountryGUI -33.209 8.919 -3.723 0.000284 ***
## CountryKEN 6.128 9.711 0.631 0.529059
## CountryMLI -25.200 8.350 -3.018 0.003021 **
## CountryMOZ -27.627 21.516 -1.284 0.201227
## CountrySAF -14.495 11.453 -1.266 0.207727
## CountrySEN -11.353 8.919 -1.273 0.205148
## CountrySWA -35.627 30.088 -1.184 0.238360
## CountryUGA -26.345 14.054 -1.875 0.062918 .
## CountryURT -5.429 12.962 -0.419 0.675975
## CountryZAI -32.767 30.088 -1.089 0.277985
## CountryZAM 9.583 30.088 0.318 0.750587
## CountryZIM -11.259 10.442 -1.078 0.282769
## ---
## Signif. codes: 0 '***' 0.001 '**' 0.01 '*' 0.05 '.' 0.1 ' ' 1
##
## (Dispersion parameter for gaussian family taken to be 884.7024)
##
## Null deviance: 180975 on 155 degrees of freedom
## Residual deviance: 124743 on 141 degrees of freedom
## AIC: 1517.4
##
## Number of Fisher Scoring iterations: 2

Anova(model1)

## Analysis of Deviance Table (Type II tests)
##
## Response: (Prev_Tspp)
## LR Chisq Df Pr(>Chisq)
## Country 63.56 14 2.76e-08 ***
## ---
## Signif. codes: 0 '***' 0.001 '**' 0.01 '*' 0.05 '.' 0.1 ' ' 1

## Statistics for Table 2

#Glm Tspp per country
data$Country <- relevel(data$Country, ref= "BKF")
model1<-glm((Prev_Tspp) ~ Country,data=data, family=gaussian())
summary(model1)

##
## Call:
## glm(formula = (Prev_Tspp) ~ Country, family = gaussian(), data = data)
##
## Deviance Residuals:
## Min 1Q Median 3Q Max
## -69.739 -19.625 -2.419 17.636 75.726
##
## Coefficients:
## Estimate Std. Error t value Pr(>|t|)
## (Intercept) 35.627 4.536 7.855 9.24e-13 ***
## CountryETH -15.587 30.088 -0.518 0.605225
## CountryGHA 34.112 8.350 4.085 7.35e-05 ***
## CountryGUI -33.209 8.919 -3.723 0.000284 ***
## CountryKEN 6.128 9.711 0.631 0.529059
## CountryMLI -25.200 8.350 -3.018 0.003021 **
## CountryMOZ -27.627 21.516 -1.284 0.201227
## CountrySAF -14.495 11.453 -1.266 0.207727
## CountrySEN -11.353 8.919 -1.273 0.205148
## CountrySWA -35.627 30.088 -1.184 0.238360
## CountryUGA -26.345 14.054 -1.875 0.062918 .
## CountryURT -5.429 12.962 -0.419 0.675975
## CountryZAI -32.767 30.088 -1.089 0.277985
## CountryZAM 9.583 30.088 0.318 0.750587
## CountryZIM -11.259 10.442 -1.078 0.282769
## ---
## Signif. codes: 0 '***' 0.001 '**' 0.01 '*' 0.05 '.' 0.1 ' ' 1
##
## (Dispersion parameter for gaussian family taken to be 884.7024)
##
## Null deviance: 180975 on 155 degrees of freedom
## Residual deviance: 124743 on 141 degrees of freedom
## AIC: 1517.4
##
## Number of Fisher Scoring iterations: 2

data$Country <- relevel(data$Country, ref= "GHA")
model1<-glm((Prev_Tspp) ~ Country,data=data, family=gaussian())
summary(model1)

##
## Call:
## glm(formula = (Prev_Tspp) ~ Country, family = gaussian(), data = data)
##
## Deviance Residuals:
## Min 1Q Median 3Q Max
## -69.739 -19.625 -2.419 17.636 75.726
##
## Coefficients:
## Estimate Std. Error t value Pr(>|t|)
## (Intercept) 69.739 7.011 9.948 < 2e-16 ***
## CountryBKF -34.112 8.350 -4.085 7.35e-05 ***
## CountryETH -49.699 30.559 -1.626 0.106109
## CountryGUI -67.321 10.399 -6.474 1.47e-09 ***
## CountryKEN -27.984 11.085 -2.525 0.012692 *
## CountryMLI -59.312 9.915 -5.982 1.72e-08 ***
## CountryMOZ -61.739 22.170 -2.785 0.006092 **
## CountrySAF -48.607 12.639 -3.846 0.000181 ***
## CountrySEN -45.465 10.399 -4.372 2.37e-05 ***
## CountrySWA -69.739 30.559 -2.282 0.023979 *
## CountryUGA -60.457 15.036 -4.021 9.41e-05 ***
## CountryURT -39.541 14.021 -2.820 0.005494 **
## CountryZAI -66.879 30.559 -2.189 0.030277 *
## CountryZAM -24.529 30.559 -0.803 0.423504
## CountryZIM -45.371 11.731 -3.868 0.000167 ***
## ---
## Signif. codes: 0 '***' 0.001 '**' 0.01 '*' 0.05 '.' 0.1 ' ' 1
##
## (Dispersion parameter for gaussian family taken to be 884.7024)
##
## Null deviance: 180975 on 155 degrees of freedom
## Residual deviance: 124743 on 141 degrees of freedom
## AIC: 1517.4
##
## Number of Fisher Scoring iterations: 2

data$Country <- relevel(data$Country, ref= "GUI")
model1<-glm((Prev_Tspp) ~ Country,data=data, family=gaussian())
summary(model1)

##
## Call:
## glm(formula = (Prev_Tspp) ~ Country, family = gaussian(), data = data)
##
## Deviance Residuals:
## Min 1Q Median 3Q Max
## -69.739 -19.625 -2.419 17.636 75.726
##
## Coefficients:
## Estimate Std. Error t value Pr(>|t|)
## (Intercept) 2.4187 7.6799 0.315 0.753275
## CountryGHA 67.3208 10.3986 6.474 1.47e-09 ***
## CountryBKF 33.2088 8.9193 3.723 0.000284 ***
## CountryETH 17.6213 30.7194 0.574 0.567137
## CountryKEN 39.3363 11.5198 3.415 0.000834 ***
## CountryMLI 8.0086 10.3986 0.770 0.442494
## CountryMOZ 5.5813 22.3904 0.249 0.803512
## CountrySAF 18.7138 13.0218 1.437 0.152901
## CountrySEN 21.8553 10.8610 2.012 0.046095 *
## CountrySWA -2.4187 30.7194 -0.079 0.937356
## CountryUGA 6.8633 15.3597 0.447 0.655676
## CountryURT 27.7797 14.3677 1.933 0.055181 .
## CountryZAI 0.4413 30.7194 0.014 0.988558
## CountryZAM 42.7913 30.7194 1.393 0.165820
## CountryZIM 21.9493 12.1429 1.808 0.072803 .
## ---
## Signif. codes: 0 '***' 0.001 '**' 0.01 '*' 0.05 '.' 0.1 ' ' 1
##
## (Dispersion parameter for gaussian family taken to be 884.7024)
##
## Null deviance: 180975 on 155 degrees of freedom
## Residual deviance: 124743 on 141 degrees of freedom
## AIC: 1517.4
##
## Number of Fisher Scoring iterations: 2

data$Country <- relevel(data$Country, ref= "ETH")
model1<-glm((Prev_Tspp) ~ Country,data=data, family=gaussian())
summary(model1)

##
## Call:
## glm(formula = (Prev_Tspp) ~ Country, family = gaussian(), data = data)
##
## Deviance Residuals:
## Min 1Q Median 3Q Max
## -69.739 -19.625 -2.419 17.636 75.726
##
## Coefficients:
## Estimate Std. Error t value Pr(>|t|)
## (Intercept) 20.040 29.744 0.674 0.502
## CountryGUI -17.621 30.719 -0.574 0.567
## CountryGHA 49.699 30.559 1.626 0.106
## CountryBKF 15.587 30.088 0.518 0.605
## CountryKEN 21.715 30.958 0.701 0.484
## CountryMLI -9.613 30.559 -0.315 0.754
## CountryMOZ -12.040 36.429 -0.331 0.742
## CountrySAF 1.092 31.548 0.035 0.972
## CountrySEN 4.234 30.719 0.138 0.891
## CountrySWA -20.040 42.064 -0.476 0.635
## CountryUGA -10.758 32.583 -0.330 0.742
## CountryURT 10.158 32.127 0.316 0.752
## CountryZAI -17.180 42.064 -0.408 0.684
## CountryZAM 25.170 42.064 0.598 0.551
## CountryZIM 4.328 31.196 0.139 0.890
##
## (Dispersion parameter for gaussian family taken to be 884.7024)
##
## Null deviance: 180975 on 155 degrees of freedom
## Residual deviance: 124743 on 141 degrees of freedom
## AIC: 1517.4
##
## Number of Fisher Scoring iterations: 2

data$Country <- relevel(data$Country, ref= "KEN")
model1<-glm((Prev_Tspp) ~ Country,data=data, family=gaussian())
summary(model1)

##
## Call:
## glm(formula = (Prev_Tspp) ~ Country, family = gaussian(), data = data)
##
## Deviance Residuals:
## Min 1Q Median 3Q Max
## -69.739 -19.625 -2.419 17.636 75.726
##
## Coefficients:
## Estimate Std. Error t value Pr(>|t|)
## (Intercept) 41.755 8.586 4.863 3.05e-06 ***
## CountryETH -21.715 30.958 -0.701 0.484195
## CountryGUI -39.336 11.520 -3.415 0.000834 ***
## CountryGHA 27.984 11.085 2.525 0.012692 *
## CountryBKF -6.128 9.711 -0.631 0.529059
## CountryMLI -31.328 11.085 -2.826 0.005396 **
## CountryMOZ -33.755 22.717 -1.486 0.139546
## CountrySAF -20.622 13.576 -1.519 0.130997
## CountrySEN -17.481 11.520 -1.517 0.131385
## CountrySWA -41.755 30.958 -1.349 0.179582
## CountryUGA -32.473 15.832 -2.051 0.042114 *
## CountryURT -11.557 14.872 -0.777 0.438415
## CountryZAI -38.895 30.958 -1.256 0.211063
## CountryZAM 3.455 30.958 0.112 0.911298
## CountryZIM -17.387 12.736 -1.365 0.174356
## ---
## Signif. codes: 0 '***' 0.001 '**' 0.01 '*' 0.05 '.' 0.1 ' ' 1
##
## (Dispersion parameter for gaussian family taken to be 884.7024)
##
## Null deviance: 180975 on 155 degrees of freedom
## Residual deviance: 124743 on 141 degrees of freedom
## AIC: 1517.4
##
## Number of Fisher Scoring iterations: 2

data$Country <- relevel(data$Country, ref= "MLI")
model1<-glm((Prev_Tspp) ~ Country,data=data, family=gaussian())
summary(model1)

##
## Call:
## glm(formula = (Prev_Tspp) ~ Country, family = gaussian(), data = data)
##
## Deviance Residuals:
## Min 1Q Median 3Q Max
## -69.739 -19.625 -2.419 17.636 75.726
##
## Coefficients:
## Estimate Std. Error t value Pr(>|t|)
## (Intercept) 10.427 7.011 1.487 0.13916
## CountryKEN 31.328 11.085 2.826 0.00540 **
## CountryETH 9.613 30.559 0.315 0.75356
## CountryGUI -8.009 10.399 -0.770 0.44249
## CountryGHA 59.312 9.915 5.982 1.72e-08 ***
## CountryBKF 25.200 8.350 3.018 0.00302 **
## CountryMOZ -2.427 22.170 -0.109 0.91297
## CountrySAF 10.705 12.639 0.847 0.39842
## CountrySEN 13.847 10.399 1.332 0.18514
## CountrySWA -10.427 30.559 -0.341 0.73345
## CountryUGA -1.145 15.036 -0.076 0.93940
## CountryURT 19.771 14.021 1.410 0.16072
## CountryZAI -7.567 30.559 -0.248 0.80478
## CountryZAM 34.783 30.559 1.138 0.25696
## CountryZIM 13.941 11.731 1.188 0.23669
## ---
## Signif. codes: 0 '***' 0.001 '**' 0.01 '*' 0.05 '.' 0.1 ' ' 1
##
## (Dispersion parameter for gaussian family taken to be 884.7024)
##
## Null deviance: 180975 on 155 degrees of freedom
## Residual deviance: 124743 on 141 degrees of freedom
## AIC: 1517.4
##
## Number of Fisher Scoring iterations: 2

data$Country <- relevel(data$Country, ref= "MOZ")
model1<-glm((Prev_Tspp) ~ Country,data=data, family=gaussian())
summary(model1)

##
## Call:
## glm(formula = (Prev_Tspp) ~ Country, family = gaussian(), data = data)
##
## Deviance Residuals:
## Min 1Q Median 3Q Max
## -69.739 -19.625 -2.419 17.636 75.726
##
## Coefficients:
## Estimate Std. Error t value Pr(>|t|)
## (Intercept) 8.000 21.032 0.380 0.70424
## CountryMLI 2.427 22.170 0.109 0.91297
## CountryKEN 33.755 22.717 1.486 0.13955
## CountryETH 12.040 36.429 0.331 0.74151
## CountryGUI -5.581 22.390 -0.249 0.80351
## CountryGHA 61.739 22.170 2.785 0.00609 **
## CountryBKF 27.627 21.516 1.284 0.20123
## CountrySAF 13.132 23.515 0.558 0.57740
## CountrySEN 16.274 22.390 0.727 0.46854
## CountrySWA -8.000 36.429 -0.220 0.82650
## CountryUGA 1.282 24.886 0.052 0.95899
## CountryURT 22.198 24.286 0.914 0.36225
## CountryZAI -5.140 36.429 -0.141 0.88799
## CountryZAM 37.210 36.429 1.021 0.30879
## CountryZIM 16.368 23.040 0.710 0.47861
## ---
## Signif. codes: 0 '***' 0.001 '**' 0.01 '*' 0.05 '.' 0.1 ' ' 1
##
## (Dispersion parameter for gaussian family taken to be 884.7024)
##
## Null deviance: 180975 on 155 degrees of freedom
## Residual deviance: 124743 on 141 degrees of freedom
## AIC: 1517.4
##
## Number of Fisher Scoring iterations: 2

data$Country <- relevel(data$Country, ref= "ZAI")
model1<-glm((Prev_Tspp) ~ Country,data=data, family=gaussian())
summary(model1)

##
## Call:
## glm(formula = (Prev_Tspp) ~ Country, family = gaussian(), data = data)
##
## Deviance Residuals:
## Min 1Q Median 3Q Max
## -69.739 -19.625 -2.419 17.636 75.726
##
## Coefficients:
## Estimate Std. Error t value Pr(>|t|)
## (Intercept) 2.8600 29.7439 0.096 0.9235
## CountryMOZ 5.1400 36.4287 0.141 0.8880
## CountryMLI 7.5672 30.5590 0.248 0.8048
## CountryKEN 38.8950 30.9585 1.256 0.2111
## CountryETH 17.1800 42.0643 0.408 0.6836
## CountryGUI -0.4413 30.7194 -0.014 0.9886
## CountryGHA 66.8794 30.5590 2.189 0.0303 *
## CountryBKF 32.7674 30.0878 1.089 0.2780
## CountrySAF 18.2725 31.5482 0.579 0.5634
## CountrySEN 21.4140 30.7194 0.697 0.4869
## CountrySWA -2.8600 42.0643 -0.068 0.9459
## CountryUGA 6.4220 32.5829 0.197 0.8440
## CountryURT 27.3383 32.1271 0.851 0.3962
## CountryZAM 42.3500 42.0643 1.007 0.3158
## CountryZIM 21.5080 31.1957 0.689 0.4917
## ---
## Signif. codes: 0 '***' 0.001 '**' 0.01 '*' 0.05 '.' 0.1 ' ' 1
##
## (Dispersion parameter for gaussian family taken to be 884.7024)
##
## Null deviance: 180975 on 155 degrees of freedom
## Residual deviance: 124743 on 141 degrees of freedom
## AIC: 1517.4
##
## Number of Fisher Scoring iterations: 2

data$Country <- relevel(data$Country, ref= "SEN")
model1<-glm((Prev_Tspp) ~ Country,data=data, family=gaussian())
summary(model1)

##
## Call:
## glm(formula = (Prev_Tspp) ~ Country, family = gaussian(), data = data)
##
## Deviance Residuals:
## Min 1Q Median 3Q Max
## -69.739 -19.625 -2.419 17.636 75.726
##
## Coefficients:
## Estimate Std. Error t value Pr(>|t|)
## (Intercept) 24.274 7.680 3.161 0.00193 **
## CountryZAI -21.414 30.719 -0.697 0.48690
## CountryMOZ -16.274 22.390 -0.727 0.46854
## CountryMLI -13.847 10.399 -1.332 0.18514
## CountryKEN 17.481 11.520 1.517 0.13139
## CountryETH -4.234 30.719 -0.138 0.89057
## CountryGUI -21.855 10.861 -2.012 0.04609 *
## CountryGHA 45.465 10.399 4.372 2.37e-05 ***
## CountryBKF 11.353 8.919 1.273 0.20515
## CountrySAF -3.142 13.022 -0.241 0.80971
## CountrySWA -24.274 30.719 -0.790 0.43075
## CountryUGA -14.992 15.360 -0.976 0.33071
## CountryURT 5.924 14.368 0.412 0.68072
## CountryZAM 20.936 30.719 0.682 0.49666
## CountryZIM 0.094 12.143 0.008 0.99383
## ---
## Signif. codes: 0 '***' 0.001 '**' 0.01 '*' 0.05 '.' 0.1 ' ' 1
##
## (Dispersion parameter for gaussian family taken to be 884.7024)
##
## Null deviance: 180975 on 155 degrees of freedom
## Residual deviance: 124743 on 141 degrees of freedom
## AIC: 1517.4
##
## Number of Fisher Scoring iterations: 2

data$Country <- relevel(data$Country, ref= "SWA")
model1<-glm((Prev_Tspp) ~ Country,data=data, family=gaussian())
summary(model1)

##
## Call:
## glm(formula = (Prev_Tspp) ~ Country, family = gaussian(), data = data)
##
## Deviance Residuals:
## Min 1Q Median 3Q Max
## -69.739 -19.625 -2.419 17.636 75.726
##
## Coefficients:
## Estimate Std. Error t value Pr(>|t|)
## (Intercept) 4.935e-13 2.974e+01 0.000 1.000
## CountrySEN 2.427e+01 3.072e+01 0.790 0.431
## CountryZAI 2.860e+00 4.206e+01 0.068 0.946
## CountryMOZ 8.000e+00 3.643e+01 0.220 0.826
## CountryMLI 1.043e+01 3.056e+01 0.341 0.733
## CountryKEN 4.175e+01 3.096e+01 1.349 0.180
## CountryETH 2.004e+01 4.206e+01 0.476 0.635
## CountryGUI 2.419e+00 3.072e+01 0.079 0.937
## CountryGHA 6.974e+01 3.056e+01 2.282 0.024 *
## CountryBKF 3.563e+01 3.009e+01 1.184 0.238
## CountrySAF 2.113e+01 3.155e+01 0.670 0.504
## CountryUGA 9.282e+00 3.258e+01 0.285 0.776
## CountryURT 3.020e+01 3.213e+01 0.940 0.349
## CountryZAM 4.521e+01 4.206e+01 1.075 0.284
## CountryZIM 2.437e+01 3.120e+01 0.781 0.436
## ---
## Signif. codes: 0 '***' 0.001 '**' 0.01 '*' 0.05 '.' 0.1 ' ' 1
##
## (Dispersion parameter for gaussian family taken to be 884.7024)
##
## Null deviance: 180975 on 155 degrees of freedom
## Residual deviance: 124743 on 141 degrees of freedom
## AIC: 1517.4
##
## Number of Fisher Scoring iterations: 2

data$Country <- relevel(data$Country, ref= "URT")
model1<-glm((Prev_Tspp) ~ Country,data=data, family=gaussian())
summary(model1)

##
## Call:
## glm(formula = (Prev_Tspp) ~ Country, family = gaussian(), data = data)
##
## Deviance Residuals:
## Min 1Q Median 3Q Max
## -69.739 -19.625 -2.419 17.636 75.726
##
## Coefficients:
## Estimate Std. Error t value Pr(>|t|)
## (Intercept) 30.198 12.143 2.487 0.01405 *
## CountrySWA -30.198 32.127 -0.940 0.34884
## CountrySEN -5.924 14.368 -0.412 0.68072
## CountryZAI -27.338 32.127 -0.851 0.39624
## CountryMOZ -22.198 24.286 -0.914 0.36225
## CountryMLI -19.771 14.021 -1.410 0.16072
## CountryKEN 11.557 14.872 0.777 0.43842
## CountryETH -10.158 32.127 -0.316 0.75232
## CountryGUI -27.780 14.368 -1.933 0.05518 .
## CountryGHA 39.541 14.021 2.820 0.00549 **
## CountryBKF 5.429 12.962 0.419 0.67597
## CountrySAF -9.066 16.064 -0.564 0.57340
## CountryUGA -20.916 18.011 -1.161 0.24747
## CountryZAM 15.012 32.127 0.467 0.64104
## CountryZIM -5.830 15.360 -0.380 0.70482
## ---
## Signif. codes: 0 '***' 0.001 '**' 0.01 '*' 0.05 '.' 0.1 ' ' 1
##
## (Dispersion parameter for gaussian family taken to be 884.7024)
##
## Null deviance: 180975 on 155 degrees of freedom
## Residual deviance: 124743 on 141 degrees of freedom
## AIC: 1517.4
##
## Number of Fisher Scoring iterations: 2

data$Country <- relevel(data$Country, ref= "UGA")
model1<-glm((Prev_Tspp) ~ Country,data=data, family=gaussian())
summary(model1)

##
## Call:
## glm(formula = (Prev_Tspp) ~ Country, family = gaussian(), data = data)
##
## Deviance Residuals:
## Min 1Q Median 3Q Max
## -69.739 -19.625 -2.419 17.636 75.726
##
## Coefficients:
## Estimate Std. Error t value Pr(>|t|)
## (Intercept) 9.282 13.302 0.698 0.4865
## CountryURT 20.916 18.011 1.161 0.2475
## CountrySWA -9.282 32.583 -0.285 0.7762
## CountrySEN 14.992 15.360 0.976 0.3307
## CountryZAI -6.422 32.583 -0.197 0.8440
## CountryMOZ -1.282 24.886 -0.052 0.9590
## CountryMLI 1.145 15.036 0.076 0.9394
## CountryKEN 32.473 15.832 2.051 0.0421 *
## CountryETH 10.758 32.583 0.330 0.7418
## CountryGUI -6.863 15.360 -0.447 0.6557
## CountryGHA 60.457 15.036 4.021 9.41e-05 ***
## CountryBKF 26.345 14.054 1.875 0.0629 .
## CountrySAF 11.850 16.957 0.699 0.4858
## CountryZAM 35.928 32.583 1.103 0.2721
## CountryZIM 15.086 16.291 0.926 0.3560
## ---
## Signif. codes: 0 '***' 0.001 '**' 0.01 '*' 0.05 '.' 0.1 ' ' 1
##
## (Dispersion parameter for gaussian family taken to be 884.7024)
##
## Null deviance: 180975 on 155 degrees of freedom
## Residual deviance: 124743 on 141 degrees of freedom
## AIC: 1517.4
##
## Number of Fisher Scoring iterations: 2

data$Country <- relevel(data$Country, ref= "SAF")
model1<-glm((Prev_Tspp) ~ Country,data=data, family=gaussian())
summary(model1)

##
## Call:
## glm(formula = (Prev_Tspp) ~ Country, family = gaussian(), data = data)
##
## Deviance Residuals:
## Min 1Q Median 3Q Max
## -69.739 -19.625 -2.419 17.636 75.726
##
## Coefficients:
## Estimate Std. Error t value Pr(>|t|)
## (Intercept) 21.133 10.516 2.010 0.046388 *
## CountryUGA -11.850 16.957 -0.699 0.485784
## CountryURT 9.066 16.064 0.564 0.573397
## CountrySWA -21.133 31.548 -0.670 0.504050
## CountrySEN 3.141 13.022 0.241 0.809713
## CountryZAI -18.272 31.548 -0.579 0.563383
## CountryMOZ -13.132 23.515 -0.558 0.577401
## CountryMLI -10.705 12.639 -0.847 0.398419
## CountryKEN 20.623 13.576 1.519 0.130997
## CountryETH -1.092 31.548 -0.035 0.972424
## CountryGUI -18.714 13.022 -1.437 0.152901
## CountryGHA 48.607 12.639 3.846 0.000181 ***
## CountryBKF 14.495 11.453 1.266 0.207727
## CountryZAM 24.077 31.548 0.763 0.446621
## CountryZIM 3.235 14.109 0.229 0.818949
## ---
## Signif. codes: 0 '***' 0.001 '**' 0.01 '*' 0.05 '.' 0.1 ' ' 1
##
## (Dispersion parameter for gaussian family taken to be 884.7024)
##
## Null deviance: 180975 on 155 degrees of freedom
## Residual deviance: 124743 on 141 degrees of freedom
## AIC: 1517.4
##
## Number of Fisher Scoring iterations: 2

data$Country <- relevel(data$Country, ref= "ZAM")
model1<-glm((Prev_Tspp) ~ Country,data=data, family=gaussian())
summary(model1)

##
## Call:
## glm(formula = (Prev_Tspp) ~ Country, family = gaussian(), data = data)
##
## Deviance Residuals:
## Min 1Q Median 3Q Max
## -69.739 -19.625 -2.419 17.636 75.726
##
## Coefficients:
## Estimate Std. Error t value Pr(>|t|)
## (Intercept) 45.210 29.744 1.520 0.131
## CountrySAF -24.078 31.548 -0.763 0.447
## CountryUGA -35.928 32.583 -1.103 0.272
## CountryURT -15.012 32.127 -0.467 0.641
## CountrySWA -45.210 42.064 -1.075 0.284
## CountrySEN -20.936 30.719 -0.682 0.497
## CountryZAI -42.350 42.064 -1.007 0.316
## CountryMOZ -37.210 36.429 -1.021 0.309
## CountryMLI -34.783 30.559 -1.138 0.257
## CountryKEN -3.455 30.958 -0.112 0.911
## CountryETH -25.170 42.064 -0.598 0.551
## CountryGUI -42.791 30.719 -1.393 0.166
## CountryGHA 24.529 30.559 0.803 0.424
## CountryBKF -9.583 30.088 -0.318 0.751
## CountryZIM -20.842 31.196 -0.668 0.505
##
## (Dispersion parameter for gaussian family taken to be 884.7024)
##
## Null deviance: 180975 on 155 degrees of freedom
## Residual deviance: 124743 on 141 degrees of freedom
## AIC: 1517.4
##
## Number of Fisher Scoring iterations: 2

data$Country <- relevel(data$Country, ref= "ZIM")
model1<-glm((Prev_Tspp) ~ Country,data=data, family=gaussian())
summary(model1)

##
## Call:
## glm(formula = (Prev_Tspp) ~ Country, family = gaussian(), data = data)
##
## Deviance Residuals:
## Min 1Q Median 3Q Max
## -69.739 -19.625 -2.419 17.636 75.726
##
## Coefficients:
## Estimate Std. Error t value Pr(>|t|)
## (Intercept) 24.368 9.406 2.591 0.010584 *
## CountryZAM 20.842 31.196 0.668 0.505159
## CountrySAF -3.236 14.109 -0.229 0.818949
## CountryUGA -15.086 16.291 -0.926 0.356024
## CountryURT 5.830 15.360 0.380 0.704824
## CountrySWA -24.368 31.196 -0.781 0.436034
## CountrySEN -0.094 12.143 -0.008 0.993834
## CountryZAI -21.508 31.196 -0.689 0.491671
## CountryMOZ -16.368 23.040 -0.710 0.478611
## CountryMLI -13.941 11.731 -1.188 0.236692
## CountryKEN 17.387 12.736 1.365 0.174356
## CountryETH -4.328 31.196 -0.139 0.889856
## CountryGUI -21.949 12.143 -1.808 0.072803 .
## CountryGHA 45.371 11.731 3.868 0.000167 ***
## CountryBKF 11.259 10.442 1.078 0.282769
## ---
## Signif. codes: 0 '***' 0.001 '**' 0.01 '*' 0.05 '.' 0.1 ' ' 1
##
## (Dispersion parameter for gaussian family taken to be 884.7024)
##
## Null deviance: 180975 on 155 degrees of freedom
## Residual deviance: 124743 on 141 degrees of freedom
## AIC: 1517.4
##
## Number of Fisher Scoring iterations: 2

#--------------------------------------------------------------
# for Sodalis

# model selection
model1<-glm((Prev_Sod) ~ Country,data=data, family=gaussian())
model2<-glm((Prev_Sod) ~ Species,data=data, family=gaussian())
model3<-glm((Prev_Sod) ~ Country*Species,data=data, family=gaussian())
model4<-glm((Prev_Sod) ~ Country+Species,data=data, family=gaussian())
model5<-glm((Prev_Sod) ~ Country*Species+Localisation,data=data, family=gaussian())
model6<-glm((Prev_Sod) ~ Country+Species+Localisation,data=data, family=gaussian())

#AICc(model1, model2, model3, model4)
model.set <- list(model1, model2, model3, model4, model5, model6)
model.names <- c("model1", "model2","model3", "model4", "model5", "model6")

aictab(model.set, modnames = model.names)

##
## Model selection based on AICc:
##
## K AICc Delta_AICc AICcWt Cum.Wt LL
## model3 27 1296.12 0.00 0.97 0.97 -615.15
## model4 23 1302.75 6.64 0.03 1.00 -624.19
## model1 16 1330.18 34.06 0.00 1.00 -647.13
## model2 11 1346.01 49.90 0.00 1.00 -661.09
## model6 100 1563.38 267.27 0.00 1.00 -498.05
## model5 103 1596.16 300.05 0.00 1.00 -489.08

summary(model3)

##
## Call:
## glm(formula = (Prev_Sod) ~ Country * Species, family = gaussian(),
## data = data)
##
## Deviance Residuals:
## Min 1Q Median 3Q Max
## -70.512 -0.338 0.000 0.000 89.357
##
## Coefficients: (124 not defined because of singularities)
## Estimate Std. Error t value Pr(>|t|)
## (Intercept) -59.79500 14.23246 -4.201 4.89e-05 ***
## CountryZAM -2.86000 15.28811 -0.187 0.851895
## CountrySAF 61.07750 15.78949 3.868 0.000173 ***
## CountryUGA 65.60250 9.66905 6.785 3.71e-10 ***
## CountryURT 67.57500 11.84212 5.706 7.43e-08 ***
## CountrySWA 59.79500 19.73687 3.030 0.002955 **
## CountrySEN 59.72231 15.67139 3.811 0.000213 ***
## CountryZAI 71.22500 19.73687 3.609 0.000438 ***
## CountryMOZ 59.79500 19.73687 3.030 0.002955 **
## CountryMLI 59.72231 15.60496 3.827 0.000201 ***
## CountryKEN 15.56111 8.21711 1.894 0.060481 .
## CountryETH 15.57000 15.28811 1.018 0.310361
## CountryGUI 66.10497 15.67139 4.218 4.58e-05 ***
## CountryGHA 59.79500 14.59283 4.098 7.30e-05 ***
## CountryBKF 59.79500 15.03115 3.978 0.000115 ***
## SpeciesGb 14.00000 19.33810 0.724 0.470391
## SpeciesGff 63.84389 16.43423 3.885 0.000162 ***
## SpeciesGmed 4.67000 8.37364 0.558 0.578007
## SpeciesGmm 77.01667 15.28811 5.038 1.54e-06 ***
## SpeciesGmsm 0.33800 7.79544 0.043 0.965482
## SpeciesGp 64.70500 12.48269 5.184 8.11e-07 ***
## SpeciesGpg 0.07269 5.52849 0.013 0.989529
## SpeciesGpp NA NA NA NA
## SpeciesGt NA NA NA NA
## CountryZAM:SpeciesGb NA NA NA NA
## CountrySAF:SpeciesGb -14.25250 21.62065 -0.659 0.510928
## CountryUGA:SpeciesGb NA NA NA NA
## CountryURT:SpeciesGb NA NA NA NA
## CountrySWA:SpeciesGb NA NA NA NA
## CountrySEN:SpeciesGb NA NA NA NA
## CountryZAI:SpeciesGb NA NA NA NA
## CountryMOZ:SpeciesGb NA NA NA NA
## CountryMLI:SpeciesGb NA NA NA NA
## CountryKEN:SpeciesGb NA NA NA NA
## CountryETH:SpeciesGb NA NA NA NA
## CountryGUI:SpeciesGb NA NA NA NA
## CountryGHA:SpeciesGb NA NA NA NA
## CountryBKF:SpeciesGb NA NA NA NA
## CountryZAM:SpeciesGff NA NA NA NA
## CountrySAF:SpeciesGff NA NA NA NA
## CountryUGA:SpeciesGff -65.39139 18.65456 -3.505 0.000626 ***
## CountryURT:SpeciesGff NA NA NA NA
## CountrySWA:SpeciesGff NA NA NA NA
## CountrySEN:SpeciesGff NA NA NA NA
## CountryZAI:SpeciesGff NA NA NA NA
## CountryMOZ:SpeciesGff NA NA NA NA
## CountryMLI:SpeciesGff NA NA NA NA
## CountryKEN:SpeciesGff NA NA NA NA
## CountryETH:SpeciesGff NA NA NA NA
## CountryGUI:SpeciesGff NA NA NA NA
## CountryGHA:SpeciesGff NA NA NA NA
## CountryBKF:SpeciesGff NA NA NA NA
## CountryZAM:SpeciesGmed NA NA NA NA
## CountrySAF:SpeciesGmed NA NA NA NA
## CountryUGA:SpeciesGmed NA NA NA NA
## CountryURT:SpeciesGmed NA NA NA NA
## CountrySWA:SpeciesGmed NA NA NA NA
## CountrySEN:SpeciesGmed NA NA NA NA
## CountryZAI:SpeciesGmed NA NA NA NA
## CountryMOZ:SpeciesGmed NA NA NA NA
## CountryMLI:SpeciesGmed NA NA NA NA
## CountryKEN:SpeciesGmed NA NA NA NA
## CountryETH:SpeciesGmed NA NA NA NA
## CountryGUI:SpeciesGmed NA NA NA NA
## CountryGHA:SpeciesGmed NA NA NA NA
## CountryBKF:SpeciesGmed NA NA NA NA
## CountryZAM:SpeciesGmm NA NA NA NA
## CountrySAF:SpeciesGmm NA NA NA NA
## CountryUGA:SpeciesGmm NA NA NA NA
## CountryURT:SpeciesGmm -8.25667 18.93094 -0.436 0.663454
## CountrySWA:SpeciesGmm NA NA NA NA
## CountrySEN:SpeciesGmm NA NA NA NA
## CountryZAI:SpeciesGmm NA NA NA NA
## CountryMOZ:SpeciesGmm NA NA NA NA
## CountryMLI:SpeciesGmm NA NA NA NA
## CountryKEN:SpeciesGmm 30.74722 16.90164 1.819 0.071184 .
## CountryETH:SpeciesGmm NA NA NA NA
## CountryGUI:SpeciesGmm NA NA NA NA
## CountryGHA:SpeciesGmm NA NA NA NA
## CountryBKF:SpeciesGmm NA NA NA NA
## CountryZAM:SpeciesGmsm NA NA NA NA
## CountrySAF:SpeciesGmsm NA NA NA NA
## CountryUGA:SpeciesGmsm NA NA NA NA
## CountryURT:SpeciesGmsm NA NA NA NA
## CountrySWA:SpeciesGmsm NA NA NA NA
## CountrySEN:SpeciesGmsm NA NA NA NA
## CountryZAI:SpeciesGmsm NA NA NA NA
## CountryMOZ:SpeciesGmsm NA NA NA NA
## CountryMLI:SpeciesGmsm NA NA NA NA
## CountryKEN:SpeciesGmsm NA NA NA NA
## CountryETH:SpeciesGmsm NA NA NA NA
## CountryGUI:SpeciesGmsm NA NA NA NA
## CountryGHA:SpeciesGmsm NA NA NA NA
## CountryBKF:SpeciesGmsm NA NA NA NA
## CountryZAM:SpeciesGp NA NA NA NA
## CountrySAF:SpeciesGp NA NA NA NA
## CountryUGA:SpeciesGp NA NA NA NA
## CountryURT:SpeciesGp NA NA NA NA
## CountrySWA:SpeciesGp NA NA NA NA
## CountrySEN:SpeciesGp NA NA NA NA
## CountryZAI:SpeciesGp NA NA NA NA
## CountryMOZ:SpeciesGp NA NA NA NA
## CountryMLI:SpeciesGp NA NA NA NA
## CountryKEN:SpeciesGp NA NA NA NA
## CountryETH:SpeciesGp NA NA NA NA
## CountryGUI:SpeciesGp NA NA NA NA
## CountryGHA:SpeciesGp NA NA NA NA
## CountryBKF:SpeciesGp NA NA NA NA
## CountryZAM:SpeciesGpg NA NA NA NA
## CountrySAF:SpeciesGpg NA NA NA NA
## CountryUGA:SpeciesGpg NA NA NA NA
## CountryURT:SpeciesGpg NA NA NA NA
## CountrySWA:SpeciesGpg NA NA NA NA
## CountrySEN:SpeciesGpg NA NA NA NA
## CountryZAI:SpeciesGpg NA NA NA NA
## CountryMOZ:SpeciesGpg NA NA NA NA
## CountryMLI:SpeciesGpg NA NA NA NA
## CountryKEN:SpeciesGpg NA NA NA NA
## CountryETH:SpeciesGpg NA NA NA NA
## CountryGUI:SpeciesGpg NA NA NA NA
## CountryGHA:SpeciesGpg NA NA NA NA
## CountryBKF:SpeciesGpg NA NA NA NA
## CountryZAM:SpeciesGpp NA NA NA NA
## CountrySAF:SpeciesGpp NA NA NA NA
## CountryUGA:SpeciesGpp NA NA NA NA
## CountryURT:SpeciesGpp NA NA NA NA
## CountrySWA:SpeciesGpp NA NA NA NA
## CountrySEN:SpeciesGpp NA NA NA NA
## CountryZAI:SpeciesGpp NA NA NA NA
## CountryMOZ:SpeciesGpp NA NA NA NA
## CountryMLI:SpeciesGpp NA NA NA NA
## CountryKEN:SpeciesGpp NA NA NA NA
## CountryETH:SpeciesGpp NA NA NA NA
## CountryGUI:SpeciesGpp NA NA NA NA
## CountryGHA:SpeciesGpp NA NA NA NA
## CountryBKF:SpeciesGpp NA NA NA NA
## CountryZAM:SpeciesGt NA NA NA NA
## CountrySAF:SpeciesGt NA NA NA NA
## CountryUGA:SpeciesGt NA NA NA NA
## CountryURT:SpeciesGt NA NA NA NA
## CountrySWA:SpeciesGt NA NA NA NA
## CountrySEN:SpeciesGt NA NA NA NA
## CountryZAI:SpeciesGt NA NA NA NA
## CountryMOZ:SpeciesGt NA NA NA NA
## CountryMLI:SpeciesGt NA NA NA NA
## CountryKEN:SpeciesGt NA NA NA NA
## CountryETH:SpeciesGt NA NA NA NA
## CountryGUI:SpeciesGt NA NA NA NA
## CountryGHA:SpeciesGt NA NA NA NA
## CountryBKF:SpeciesGt NA NA NA NA
## ---
## Signif. codes: 0 '***' 0.001 '**' 0.01 '*' 0.05 '.' 0.1 ' ' 1
##
## (Dispersion parameter for gaussian family taken to be 186.9811)
##
## Null deviance: 64686 on 155 degrees of freedom
## Residual deviance: 24308 on 130 degrees of freedom
## AIC: 1284.3
##
## Number of Fisher Scoring iterations: 2

Anova(model3)

## Analysis of Deviance Table (Type II tests)
##
## Response: (Prev_Sod)
## LR Chisq Df Pr(>Chisq)
## Country 88.292 12 1.056e-13 ***
## Species 49.906 7 1.507e-08 ***
## Country:Species 15.979 4 0.003048 **
## ---
## Signif. codes: 0 '***' 0.001 '**' 0.01 '*' 0.05 '.' 0.1 ' ' 1

Anova(model1)

## Analysis of Deviance Table (Type II tests)
##
## Response: (Prev_Sod)
## LR Chisq Df Pr(>Chisq)
## Country 108.02 14 < 2.2e-16 ***
## ---
## Signif. codes: 0 '***' 0.001 '**' 0.01 '*' 0.05 '.' 0.1 ' ' 1

Anova(model2)

## Analysis of Deviance Table (Type II tests)
##
## Response: (Prev_Sod)
## LR Chisq Df Pr(>Chisq)
## Species 69.601 9 1.824e-11 ***
## ---
## Signif. codes: 0 '***' 0.001 '**' 0.01 '*' 0.05 '.' 0.1 ' ' 1

data$Country <- relevel(data$Country, ref= "BKF")
model1<-glm((Prev_Sod) ~ Country,data=data, family=gaussian())
summary(model1)

##
## Call:
## glm(formula = (Prev_Sod) ~ Country, family = gaussian(), data = data)
##
## Deviance Residuals:
## Min 1Q Median 3Q Max
## -57.262 -0.518 0.000 0.000 89.357
##
## Coefficients:
## Estimate Std. Error t value Pr(>|t|)
## (Intercept) 0.5177 2.4578 0.211 0.8335
## CountryZIM 11.7793 5.6584 2.082 0.0392 *
## CountryZAM 1.5323 16.3035 0.094 0.9253
## CountrySAF 0.6386 6.2058 0.103 0.9182
## CountryUGA 56.7443 7.6154 7.451 8.47e-12 ***
## CountryURT 40.2907 7.0239 5.736 5.68e-08 ***
## CountrySWA -0.5177 16.3035 -0.032 0.9747
## CountrySEN -0.5177 4.8331 -0.107 0.9149
## CountryZAI 10.9123 16.3035 0.669 0.5044
## CountryMOZ 6.4823 11.6586 0.556 0.5791
## CountryMLI -0.5177 4.5246 -0.114 0.9091
## CountryKEN 23.3982 5.2619 4.447 1.75e-05 ***
## CountryETH 19.9623 16.3035 1.224 0.2228
## CountryGUI 5.8650 4.8331 1.214 0.2270
## CountryGHA -0.5177 4.5246 -0.114 0.9091
## ---
## Signif. codes: 0 '***' 0.001 '**' 0.01 '*' 0.05 '.' 0.1 ' ' 1
##
## (Dispersion parameter for gaussian family taken to be 259.7641)
##
## Null deviance: 64686 on 155 degrees of freedom
## Residual deviance: 36627 on 141 degrees of freedom
## AIC: 1326.3
##
## Number of Fisher Scoring iterations: 2

data$Country <- relevel(data$Country, ref= "GHA")
model1<-glm((Prev_Sod) ~ Country,data=data, family=gaussian())
summary(model1)

##
## Call:
## glm(formula = (Prev_Sod) ~ Country, family = gaussian(), data = data)
##
## Deviance Residuals:
## Min 1Q Median 3Q Max
## -57.262 -0.518 0.000 0.000 89.357
##
## Coefficients:
## Estimate Std. Error t value Pr(>|t|)
## (Intercept) -2.073e-14 3.799e+00 0.000 1.000000
## CountryBKF 5.177e-01 4.525e+00 0.114 0.909074
## CountryZIM 1.230e+01 6.357e+00 1.934 0.055055 .
## CountryZAM 2.050e+00 1.656e+01 0.124 0.901649
## CountrySAF 1.156e+00 6.848e+00 0.169 0.866170
## CountryUGA 5.726e+01 8.148e+00 7.028 8.24e-11 ***
## CountryURT 4.081e+01 7.598e+00 5.371 3.15e-07 ***
## CountrySWA 4.437e-14 1.656e+01 0.000 1.000000
## CountrySEN 6.333e-14 5.635e+00 0.000 1.000000
## CountryZAI 1.143e+01 1.656e+01 0.690 0.491162
## CountryMOZ 7.000e+00 1.201e+01 0.583 0.561026
## CountryMLI 2.074e-14 5.372e+00 0.000 1.000000
## CountryKEN 2.392e+01 6.007e+00 3.982 0.000109 ***
## CountryETH 2.048e+01 1.656e+01 1.237 0.218217
## CountryGUI 6.383e+00 5.635e+00 1.133 0.259238
## ---
## Signif. codes: 0 '***' 0.001 '**' 0.01 '*' 0.05 '.' 0.1 ' ' 1
##
## (Dispersion parameter for gaussian family taken to be 259.7641)
##
## Null deviance: 64686 on 155 degrees of freedom
## Residual deviance: 36627 on 141 degrees of freedom
## AIC: 1326.3
##
## Number of Fisher Scoring iterations: 2

data$Country <- relevel(data$Country, ref= "GUI")
model1<-glm((Prev_Sod) ~ Country,data=data, family=gaussian())
summary(model1)

##
## Call:
## glm(formula = (Prev_Sod) ~ Country, family = gaussian(), data = data)
##
## Deviance Residuals:
## Min 1Q Median 3Q Max
## -57.262 -0.518 0.000 0.000 89.357
##
## Coefficients:
## Estimate Std. Error t value Pr(>|t|)
## (Intercept) 6.3827 4.1614 1.534 0.12733
## CountryGHA -6.3827 5.6346 -1.133 0.25924
## CountryBKF -5.8650 4.8331 -1.214 0.22696
## CountryZIM 5.9143 6.5798 0.899 0.37026
## CountryZAM -4.3327 16.6458 -0.260 0.79502
## CountrySAF -5.2264 7.0561 -0.741 0.46011
## CountryUGA 50.8793 8.3229 6.113 9.05e-09 ***
## CountryURT 34.4257 7.7853 4.422 1.94e-05 ***
## CountrySWA -6.3827 16.6458 -0.383 0.70197
## CountrySEN -6.3827 5.8852 -1.085 0.27998
## CountryZAI 5.0473 16.6458 0.303 0.76217
## CountryMOZ 0.6173 12.1326 0.051 0.95949
## CountryMLI -6.3827 5.6346 -1.133 0.25924
## CountryKEN 17.5332 6.2422 2.809 0.00568 **
## CountryETH 14.0973 16.6458 0.847 0.39848
## ---
## Signif. codes: 0 '***' 0.001 '**' 0.01 '*' 0.05 '.' 0.1 ' ' 1
##
## (Dispersion parameter for gaussian family taken to be 259.7641)
##
## Null deviance: 64686 on 155 degrees of freedom
## Residual deviance: 36627 on 141 degrees of freedom
## AIC: 1326.3
##
## Number of Fisher Scoring iterations: 2

data$Country <- relevel(data$Country, ref= "ETH")
model1<-glm((Prev_Sod) ~ Country,data=data, family=gaussian())
summary(model1)

##
## Call:
## glm(formula = (Prev_Sod) ~ Country, family = gaussian(), data = data)
##
## Deviance Residuals:
## Min 1Q Median 3Q Max
## -57.262 -0.518 0.000 0.000 89.357
##
## Coefficients:
## Estimate Std. Error t value Pr(>|t|)
## (Intercept) 20.480 16.117 1.271 0.206
## CountryGUI -14.097 16.646 -0.847 0.398
## CountryGHA -20.480 16.559 -1.237 0.218
## CountryBKF -19.962 16.304 -1.224 0.223
## CountryZIM -8.183 16.904 -0.484 0.629
## CountryZAM -18.430 22.793 -0.809 0.420
## CountrySAF -19.324 17.095 -1.130 0.260
## CountryUGA 36.782 17.656 2.083 0.039 *
## CountryURT 20.328 17.409 1.168 0.245
## CountrySWA -20.480 22.793 -0.899 0.370
## CountrySEN -20.480 16.646 -1.230 0.221
## CountryZAI -9.050 22.793 -0.397 0.692
## CountryMOZ -13.480 19.739 -0.683 0.496
## CountryMLI -20.480 16.559 -1.237 0.218
## CountryKEN 3.436 16.775 0.205 0.838
## ---
## Signif. codes: 0 '***' 0.001 '**' 0.01 '*' 0.05 '.' 0.1 ' ' 1
##
## (Dispersion parameter for gaussian family taken to be 259.7641)
##
## Null deviance: 64686 on 155 degrees of freedom
## Residual deviance: 36627 on 141 degrees of freedom
## AIC: 1326.3
##
## Number of Fisher Scoring iterations: 2

data$Country <- relevel(data$Country, ref= "KEN")
model1<-glm((Prev_Sod) ~ Country,data=data, family=gaussian())
summary(model1)

##
## Call:
## glm(formula = (Prev_Sod) ~ Country, family = gaussian(), data = data)
##
## Deviance Residuals:
## Min 1Q Median 3Q Max
## -57.262 -0.518 0.000 0.000 89.357
##
## Coefficients:
## Estimate Std. Error t value Pr(>|t|)
## (Intercept) 23.916 4.653 5.140 8.99e-07 ***
## CountryETH -3.436 16.775 -0.205 0.838012
## CountryGUI -17.533 6.242 -2.809 0.005678 **
## CountryGHA -23.916 6.007 -3.982 0.000109 ***
## CountryBKF -23.398 5.262 -4.447 1.75e-05 ***
## CountryZIM -11.619 6.901 -1.684 0.094462 .
## CountryZAM -21.866 16.775 -1.303 0.194544
## CountrySAF -22.760 7.356 -3.094 0.002383 **
## CountryUGA 33.346 8.579 3.887 0.000156 ***
## CountryURT 16.893 8.059 2.096 0.037850 *
## CountrySWA -23.916 16.775 -1.426 0.156178
## CountrySEN -23.916 6.242 -3.831 0.000191 ***
## CountryZAI -12.486 16.775 -0.744 0.457935
## CountryMOZ -16.916 12.310 -1.374 0.171564
## CountryMLI -23.916 6.007 -3.982 0.000109 ***
## ---
## Signif. codes: 0 '***' 0.001 '**' 0.01 '*' 0.05 '.' 0.1 ' ' 1
##
## (Dispersion parameter for gaussian family taken to be 259.7641)
##
## Null deviance: 64686 on 155 degrees of freedom
## Residual deviance: 36627 on 141 degrees of freedom
## AIC: 1326.3
##
## Number of Fisher Scoring iterations: 2

data$Country <- relevel(data$Country, ref= "MLI")
model1<-glm((Prev_Sod) ~ Country,data=data, family=gaussian())
summary(model1)

##
## Call:
## glm(formula = (Prev_Sod) ~ Country, family = gaussian(), data = data)
##
## Deviance Residuals:
## Min 1Q Median 3Q Max
## -57.262 -0.518 0.000 0.000 89.357
##
## Coefficients:
## Estimate Std. Error t value Pr(>|t|)
## (Intercept) -1.593e-14 3.799e+00 0.000 1.000000
## CountryKEN 2.392e+01 6.007e+00 3.982 0.000109 ***
## CountryETH 2.048e+01 1.656e+01 1.237 0.218217
## CountryGUI 6.383e+00 5.635e+00 1.133 0.259238
## CountryGHA 1.606e-14 5.372e+00 0.000 1.000000
## CountryBKF 5.177e-01 4.525e+00 0.114 0.909074
## CountryZIM 1.230e+01 6.357e+00 1.934 0.055055 .
## CountryZAM 2.050e+00 1.656e+01 0.124 0.901649
## CountrySAF 1.156e+00 6.848e+00 0.169 0.866170
## CountryUGA 5.726e+01 8.148e+00 7.028 8.24e-11 ***
## CountryURT 4.081e+01 7.598e+00 5.371 3.15e-07 ***
## CountrySWA -1.030e-14 1.656e+01 0.000 1.000000
## CountrySEN -5.405e-15 5.635e+00 0.000 1.000000
## CountryZAI 1.143e+01 1.656e+01 0.690 0.491162
## CountryMOZ 7.000e+00 1.201e+01 0.583 0.561026
## ---
## Signif. codes: 0 '***' 0.001 '**' 0.01 '*' 0.05 '.' 0.1 ' ' 1
##
## (Dispersion parameter for gaussian family taken to be 259.7641)
##
## Null deviance: 64686 on 155 degrees of freedom
## Residual deviance: 36627 on 141 degrees of freedom
## AIC: 1326.3
##
## Number of Fisher Scoring iterations: 2

data$Country <- relevel(data$Country, ref= "MOZ")
model1<-glm((Prev_Sod) ~ Country,data=data, family=gaussian())
summary(model1)

##
## Call:
## glm(formula = (Prev_Sod) ~ Country, family = gaussian(), data = data)
##
## Deviance Residuals:
## Min 1Q Median 3Q Max
## -57.262 -0.518 0.000 0.000 89.357
##
## Coefficients:
## Estimate Std. Error t value Pr(>|t|)
## (Intercept) 7.0000 11.3966 0.614 0.540060
## CountryMLI -7.0000 12.0130 -0.583 0.561026
## CountryKEN 16.9158 12.3097 1.374 0.171564
## CountryETH 13.4800 19.7395 0.683 0.495793
## CountryGUI -0.6173 12.1326 -0.051 0.959491
## CountryGHA -7.0000 12.0130 -0.583 0.561026
## CountryBKF -6.4823 11.6586 -0.556 0.579084
## CountryZIM 5.2970 12.4843 0.424 0.672000
## CountryZAM -4.9500 19.7395 -0.251 0.802359
## CountrySAF -5.8438 12.7418 -0.459 0.647206
## CountryUGA 50.2620 13.4846 3.727 0.000279 ***
## CountryURT 33.8083 13.1596 2.569 0.011236 *
## CountrySWA -7.0000 19.7395 -0.355 0.723404
## CountrySEN -7.0000 12.1326 -0.577 0.564887
## CountryZAI 4.4300 19.7395 0.224 0.822753
## ---
## Signif. codes: 0 '***' 0.001 '**' 0.01 '*' 0.05 '.' 0.1 ' ' 1
##
## (Dispersion parameter for gaussian family taken to be 259.7641)
##
## Null deviance: 64686 on 155 degrees of freedom
## Residual deviance: 36627 on 141 degrees of freedom
## AIC: 1326.3
##
## Number of Fisher Scoring iterations: 2

data$Country <- relevel(data$Country, ref= "ZAI")
model1<-glm((Prev_Sod) ~ Country,data=data, family=gaussian())
summary(model1)

##
## Call:
## glm(formula = (Prev_Sod) ~ Country, family = gaussian(), data = data)
##
## Deviance Residuals:
## Min 1Q Median 3Q Max
## -57.262 -0.518 0.000 0.000 89.357
##
## Coefficients:
## Estimate Std. Error t value Pr(>|t|)
## (Intercept) 11.430 16.117 0.709 0.4794
## CountryMOZ -4.430 19.739 -0.224 0.8228
## CountryMLI -11.430 16.559 -0.690 0.4912
## CountryKEN 12.486 16.775 0.744 0.4579
## CountryETH 9.050 22.793 0.397 0.6919
## CountryGUI -5.047 16.646 -0.303 0.7622
## CountryGHA -11.430 16.559 -0.690 0.4912
## CountryBKF -10.912 16.303 -0.669 0.5044
## CountryZIM 0.867 16.904 0.051 0.9592
## CountryZAM -9.380 22.793 -0.412 0.6813
## CountrySAF -10.274 17.095 -0.601 0.5488
## CountryUGA 45.832 17.655 2.596 0.0104 *
## CountryURT 29.378 17.409 1.688 0.0937 .
## CountrySWA -11.430 22.793 -0.501 0.6168
## CountrySEN -11.430 16.646 -0.687 0.4934
## ---
## Signif. codes: 0 '***' 0.001 '**' 0.01 '*' 0.05 '.' 0.1 ' ' 1
##
## (Dispersion parameter for gaussian family taken to be 259.7641)
##
## Null deviance: 64686 on 155 degrees of freedom
## Residual deviance: 36627 on 141 degrees of freedom
## AIC: 1326.3
##
## Number of Fisher Scoring iterations: 2

data$Country <- relevel(data$Country, ref= "SEN")
model1<-glm((Prev_Sod) ~ Country,data=data, family=gaussian())
summary(model1)

##
## Call:
## glm(formula = (Prev_Sod) ~ Country, family = gaussian(), data = data)
##
## Deviance Residuals:
## Min 1Q Median 3Q Max
## -57.262 -0.518 0.000 0.000 89.357
##
## Coefficients:
## Estimate Std. Error t value Pr(>|t|)
## (Intercept) -5.003e-14 4.161e+00 0.000 1.000000
## CountryZAI 1.143e+01 1.665e+01 0.687 0.493424
## CountryMOZ 7.000e+00 1.213e+01 0.577 0.564887
## CountryMLI 5.839e-14 5.635e+00 0.000 1.000000
## CountryKEN 2.392e+01 6.242e+00 3.831 0.000191 ***
## CountryETH 2.048e+01 1.665e+01 1.230 0.220618
## CountryGUI 6.383e+00 5.885e+00 1.085 0.279979
## CountryGHA 3.874e-14 5.635e+00 0.000 1.000000
## CountryBKF 5.177e-01 4.833e+00 0.107 0.914853
## CountryZIM 1.230e+01 6.580e+00 1.869 0.063712 .
## CountryZAM 2.050e+00 1.665e+01 0.123 0.902160
## CountrySAF 1.156e+00 7.056e+00 0.164 0.870071
## CountryUGA 5.726e+01 8.323e+00 6.880 1.80e-10 ***
## CountryURT 4.081e+01 7.785e+00 5.242 5.69e-07 ***
## CountrySWA 3.082e-13 1.665e+01 0.000 1.000000
## ---
## Signif. codes: 0 '***' 0.001 '**' 0.01 '*' 0.05 '.' 0.1 ' ' 1
##
## (Dispersion parameter for gaussian family taken to be 259.7641)
##
## Null deviance: 64686 on 155 degrees of freedom
## Residual deviance: 36627 on 141 degrees of freedom
## AIC: 1326.3
##
## Number of Fisher Scoring iterations: 2

data$Country <- relevel(data$Country, ref= "SWA")
model1<-glm((Prev_Sod) ~ Country,data=data, family=gaussian())
summary(model1)

##
## Call:
## glm(formula = (Prev_Sod) ~ Country, family = gaussian(), data = data)
##
## Deviance Residuals:
## Min 1Q Median 3Q Max
## -57.262 -0.518 0.000 0.000 89.357
##
## Coefficients:
## Estimate Std. Error t value Pr(>|t|)
## (Intercept) 2.094e-13 1.612e+01 0.000 1.00000
## CountrySEN -2.094e-13 1.665e+01 0.000 1.00000
## CountryZAI 1.143e+01 2.279e+01 0.501 0.61683
## CountryMOZ 7.000e+00 1.974e+01 0.355 0.72340
## CountryMLI -2.721e-13 1.656e+01 0.000 1.00000
## CountryKEN 2.392e+01 1.678e+01 1.426 0.15618
## CountryETH 2.048e+01 2.279e+01 0.899 0.37044
## CountryGUI 6.383e+00 1.665e+01 0.383 0.70197
## CountryGHA -1.970e-13 1.656e+01 0.000 1.00000
## CountryBKF 5.177e-01 1.630e+01 0.032 0.97471
## CountryZIM 1.230e+01 1.690e+01 0.727 0.46815
## CountryZAM 2.050e+00 2.279e+01 0.090 0.92846
## CountrySAF 1.156e+00 1.709e+01 0.068 0.94617
## CountryUGA 5.726e+01 1.766e+01 3.243 0.00148 **
## CountryURT 4.081e+01 1.741e+01 2.344 0.02047 *
## ---
## Signif. codes: 0 '***' 0.001 '**' 0.01 '*' 0.05 '.' 0.1 ' ' 1
##
## (Dispersion parameter for gaussian family taken to be 259.7641)
##
## Null deviance: 64686 on 155 degrees of freedom
## Residual deviance: 36627 on 141 degrees of freedom
## AIC: 1326.3
##
## Number of Fisher Scoring iterations: 2

data$Country <- relevel(data$Country, ref= "URT")
model1<-glm((Prev_Sod) ~ Country,data=data, family=gaussian())
summary(model1)

##
## Call:
## glm(formula = (Prev_Sod) ~ Country, family = gaussian(), data = data)
##
## Deviance Residuals:
## Min 1Q Median 3Q Max
## -57.262 -0.518 0.000 0.000 89.357
##
## Coefficients:
## Estimate Std. Error t value Pr(>|t|)
## (Intercept) 40.808 6.580 6.202 5.82e-09 ***
## CountrySWA -40.808 17.409 -2.344 0.020467 *
## CountrySEN -40.808 7.785 -5.242 5.69e-07 ***
## CountryZAI -29.378 17.409 -1.688 0.093703 .
## CountryMOZ -33.808 13.160 -2.569 0.011236 *
## CountryMLI -40.808 7.598 -5.371 3.15e-07 ***
## CountryKEN -16.892 8.059 -2.096 0.037850 *
## CountryETH -20.328 17.409 -1.168 0.244890
## CountryGUI -34.426 7.785 -4.422 1.94e-05 ***
## CountryGHA -40.808 7.598 -5.371 3.15e-07 ***
## CountryBKF -40.291 7.024 -5.736 5.68e-08 ***
## CountryZIM -28.511 8.323 -3.426 0.000804 ***
## CountryZAM -38.758 17.409 -2.226 0.027574 *
## CountrySAF -39.652 8.704 -4.555 1.12e-05 ***
## CountryUGA 16.454 9.759 1.686 0.094022 .
## ---
## Signif. codes: 0 '***' 0.001 '**' 0.01 '*' 0.05 '.' 0.1 ' ' 1
##
## (Dispersion parameter for gaussian family taken to be 259.7641)
##
## Null deviance: 64686 on 155 degrees of freedom
## Residual deviance: 36627 on 141 degrees of freedom
## AIC: 1326.3
##
## Number of Fisher Scoring iterations: 2

data$Country <- relevel(data$Country, ref= "UGA")
model1<-glm((Prev_Sod) ~ Country,data=data, family=gaussian())
summary(model1)

##
## Call:
## glm(formula = (Prev_Sod) ~ Country, family = gaussian(), data = data)
##
## Deviance Residuals:
## Min 1Q Median 3Q Max
## -57.262 -0.518 0.000 0.000 89.357
##
## Coefficients:
## Estimate Std. Error t value Pr(>|t|)
## (Intercept) 57.262 7.208 7.944 5.61e-13 ***
## CountryURT -16.454 9.759 -1.686 0.094022 .
## CountrySWA -57.262 17.656 -3.243 0.001475 **
## CountrySEN -57.262 8.323 -6.880 1.80e-10 ***
## CountryZAI -45.832 17.656 -2.596 0.010433 *
## CountryMOZ -50.262 13.485 -3.727 0.000279 ***
## CountryMLI -57.262 8.148 -7.028 8.24e-11 ***
## CountryKEN -33.346 8.579 -3.887 0.000156 ***
## CountryETH -36.782 17.656 -2.083 0.039028 *
## CountryGUI -50.879 8.323 -6.113 9.05e-09 ***
## CountryGHA -57.262 8.148 -7.028 8.24e-11 ***
## CountryBKF -56.744 7.615 -7.451 8.47e-12 ***
## CountryZIM -44.965 8.828 -5.094 1.11e-06 ***
## CountryZAM -55.212 17.656 -3.127 0.002144 **
## CountrySAF -56.106 9.188 -6.106 9.37e-09 ***
## ---
## Signif. codes: 0 '***' 0.001 '**' 0.01 '*' 0.05 '.' 0.1 ' ' 1
##
## (Dispersion parameter for gaussian family taken to be 259.7641)
##
## Null deviance: 64686 on 155 degrees of freedom
## Residual deviance: 36627 on 141 degrees of freedom
## AIC: 1326.3
##
## Number of Fisher Scoring iterations: 2

data$Country <- relevel(data$Country, ref= "SAF")
model1<-glm((Prev_Sod) ~ Country,data=data, family=gaussian())
summary(model1)

##
## Call:
## glm(formula = (Prev_Sod) ~ Country, family = gaussian(), data = data)
##
## Deviance Residuals:
## Min 1Q Median 3Q Max
## -57.262 -0.518 0.000 0.000 89.357
##
## Coefficients:
## Estimate Std. Error t value Pr(>|t|)
## (Intercept) 1.1562 5.6983 0.203 0.83950
## CountryUGA 56.1057 9.1882 6.106 9.37e-09 ***
## CountryURT 39.6521 8.7043 4.555 1.12e-05 ***
## CountrySWA -1.1562 17.0949 -0.068 0.94617
## CountrySEN -1.1563 7.0561 -0.164 0.87007
## CountryZAI 10.2737 17.0949 0.601 0.54882
## CountryMOZ 5.8438 12.7418 0.459 0.64721
## CountryMLI -1.1562 6.8485 -0.169 0.86617
## CountryKEN 22.7596 7.3565 3.094 0.00238 **
## CountryETH 19.3238 17.0949 1.130 0.26023
## CountryGUI 5.2264 7.0561 0.741 0.46011
## CountryGHA -1.1562 6.8485 -0.169 0.86617
## CountryBKF -0.6386 6.2058 -0.103 0.91819
## CountryZIM 11.1408 7.6451 1.457 0.14727
## CountryZAM 0.8938 17.0949 0.052 0.95838
## ---
## Signif. codes: 0 '***' 0.001 '**' 0.01 '*' 0.05 '.' 0.1 ' ' 1
##
## (Dispersion parameter for gaussian family taken to be 259.7641)
##
## Null deviance: 64686 on 155 degrees of freedom
## Residual deviance: 36627 on 141 degrees of freedom
## AIC: 1326.3
##
## Number of Fisher Scoring iterations: 2

data$Country <- relevel(data$Country, ref= "ZAM")
model1<-glm((Prev_Sod) ~ Country,data=data, family=gaussian())
summary(model1)

##
## Call:
## glm(formula = (Prev_Sod) ~ Country, family = gaussian(), data = data)
##
## Deviance Residuals:
## Min 1Q Median 3Q Max
## -57.262 -0.518 0.000 0.000 89.357
##
## Coefficients:
## Estimate Std. Error t value Pr(>|t|)
## (Intercept) 2.0500 16.1172 0.127 0.89897
## CountrySAF -0.8937 17.0949 -0.052 0.95838
## CountryUGA 55.2120 17.6555 3.127 0.00214 **
## CountryURT 38.7583 17.4086 2.226 0.02757 *
## CountrySWA -2.0500 22.7932 -0.090 0.92846
## CountrySEN -2.0500 16.6458 -0.123 0.90216
## CountryZAI 9.3800 22.7932 0.412 0.68131
## CountryMOZ 4.9500 19.7395 0.251 0.80236
## CountryMLI -2.0500 16.5588 -0.124 0.90165
## CountryKEN 21.8658 16.7753 1.303 0.19454
## CountryETH 18.4300 22.7932 0.809 0.42012
## CountryGUI 4.3327 16.6458 0.260 0.79502
## CountryGHA -2.0500 16.5588 -0.124 0.90165
## CountryBKF -1.5323 16.3035 -0.094 0.92525
## CountryZIM 10.2470 16.9039 0.606 0.54536
## ---
## Signif. codes: 0 '***' 0.001 '**' 0.01 '*' 0.05 '.' 0.1 ' ' 1
##
## (Dispersion parameter for gaussian family taken to be 259.7641)
##
## Null deviance: 64686 on 155 degrees of freedom
## Residual deviance: 36627 on 141 degrees of freedom
## AIC: 1326.3
##
## Number of Fisher Scoring iterations: 2

data$Country <- relevel(data$Country, ref= "ZIM")
model1<-glm((Prev_Sod) ~ Country,data=data, family=gaussian())
summary(model1)

##
## Call:
## glm(formula = (Prev_Sod) ~ Country, family = gaussian(), data = data)
##
## Deviance Residuals:
## Min 1Q Median 3Q Max
## -57.262 -0.518 0.000 0.000 89.357
##
## Coefficients:
## Estimate Std. Error t value Pr(>|t|)
## (Intercept) 12.297 5.097 2.413 0.017118 *
## CountryZAM -10.247 16.904 -0.606 0.545361
## CountrySAF -11.141 7.645 -1.457 0.147271
## CountryUGA 44.965 8.828 5.094 1.11e-06 ***
## CountryURT 28.511 8.323 3.426 0.000804 ***
## CountrySWA -12.297 16.904 -0.727 0.468146
## CountrySEN -12.297 6.580 -1.869 0.063712 .
## CountryZAI -0.867 16.904 -0.051 0.959167
## CountryMOZ -5.297 12.484 -0.424 0.672000
## CountryMLI -12.297 6.357 -1.934 0.055055 .
## CountryKEN 11.619 6.901 1.684 0.094462 .
## CountryETH 8.183 16.904 0.484 0.629073
## CountryGUI -5.914 6.580 -0.899 0.370260
## CountryGHA -12.297 6.357 -1.934 0.055055 .
## CountryBKF -11.779 5.658 -2.082 0.039174 *
## ---
## Signif. codes: 0 '***' 0.001 '**' 0.01 '*' 0.05 '.' 0.1 ' ' 1
##
## (Dispersion parameter for gaussian family taken to be 259.7641)
##
## Null deviance: 64686 on 155 degrees of freedom
## Residual deviance: 36627 on 141 degrees of freedom
## AIC: 1326.3
##
## Number of Fisher Scoring iterations: 2

## Statistics for table 3

# for trypanosome Tspp

data$Species <- relevel(data$Species, ref= "Ga")
model1<-glm((Prev_Tspp) ~ Species,data=data, family=gaussian())
summary(model1)

##
## Call:
## glm(formula = (Prev_Tspp) ~ Species, family = gaussian(), data = data)
##
## Deviance Residuals:
## Min 1Q Median 3Q Max
## -53.02 -23.57 -14.97 21.89 76.43
##
## Coefficients:
## Estimate Std. Error t value Pr(>|t|)
## (Intercept) 14.970 10.945 1.368 0.17349
## SpeciesGb 2.096 18.314 0.114 0.90904
## SpeciesGff 2.747 21.890 0.125 0.90032
## SpeciesGmed 28.225 19.731 1.430 0.15472
## SpeciesGmm 9.161 15.955 0.574 0.56672
## SpeciesGmsm 1.064 18.314 0.058 0.95375
## SpeciesGp 21.049 13.082 1.609 0.10977
## SpeciesGpg 8.596 11.591 0.742 0.45955
## SpeciesGpp -12.110 34.611 -0.350 0.72692
## SpeciesGt 38.052 12.699 2.997 0.00321 **
## ---
## Signif. codes: 0 '***' 0.001 '**' 0.01 '*' 0.05 '.' 0.1 ' ' 1
##
## (Dispersion parameter for gaussian family taken to be 1078.113)
##
## Null deviance: 180975 on 155 degrees of freedom
## Residual deviance: 157405 on 146 degrees of freedom
## AIC: 1543.7
##
## Number of Fisher Scoring iterations: 2

data$Species <- relevel(data$Species, ref= "Gb")
model1<-glm((Prev_Tspp) ~ Species,data=data, family=gaussian())
summary(model1)

##
## Call:
## glm(formula = (Prev_Tspp) ~ Species, family = gaussian(), data = data)
##
## Deviance Residuals:
## Min 1Q Median 3Q Max
## -53.02 -23.57 -14.97 21.89 76.43
##
## Coefficients:
## Estimate Std. Error t value Pr(>|t|)
## (Intercept) 17.0660 14.6841 1.162 0.2470
## SpeciesGa -2.0960 18.3143 -0.114 0.9090
## SpeciesGff 0.6507 23.9790 0.027 0.9784
## SpeciesGmed 26.1290 22.0261 1.186 0.2374
## SpeciesGmm 7.0652 18.7186 0.377 0.7064
## SpeciesGmsm -1.0320 20.7664 -0.050 0.9604
## SpeciesGp 18.9526 16.3390 1.160 0.2480
## SpeciesGpg 6.4995 15.1721 0.428 0.6690
## SpeciesGpp -14.2060 35.9685 -0.395 0.6935
## SpeciesGt 35.9559 16.0340 2.242 0.0264 *
## ---
## Signif. codes: 0 '***' 0.001 '**' 0.01 '*' 0.05 '.' 0.1 ' ' 1
##
## (Dispersion parameter for gaussian family taken to be 1078.113)
##
## Null deviance: 180975 on 155 degrees of freedom
## Residual deviance: 157405 on 146 degrees of freedom
## AIC: 1543.7
##
## Number of Fisher Scoring iterations: 2

data$Species <- relevel(data$Species, ref= "Gff")
model1<-glm((Prev_Tspp) ~ Species,data=data, family=gaussian())
summary(model1)

##
## Call:
## glm(formula = (Prev_Tspp) ~ Species, family = gaussian(), data = data)
##
## Deviance Residuals:
## Min 1Q Median 3Q Max
## -53.02 -23.57 -14.97 21.89 76.43
##
## Coefficients:
## Estimate Std. Error t value Pr(>|t|)
## (Intercept) 17.7167 18.9571 0.935 0.3516
## SpeciesGb -0.6507 23.9790 -0.027 0.9784
## SpeciesGa -2.7467 21.8898 -0.125 0.9003
## SpeciesGmed 25.4783 25.0779 1.016 0.3113
## SpeciesGmm 6.4146 22.2292 0.289 0.7733
## SpeciesGmsm -1.6827 23.9790 -0.070 0.9442
## SpeciesGp 18.3019 20.2660 0.903 0.3680
## SpeciesGpg 5.8489 19.3375 0.302 0.7627
## SpeciesGpp -14.8567 37.9142 -0.392 0.6957
## SpeciesGt 35.3053 20.0209 1.763 0.0799 .
## ---
## Signif. codes: 0 '***' 0.001 '**' 0.01 '*' 0.05 '.' 0.1 ' ' 1
##
## (Dispersion parameter for gaussian family taken to be 1078.113)
##
## Null deviance: 180975 on 155 degrees of freedom
## Residual deviance: 157405 on 146 degrees of freedom
## AIC: 1543.7
##
## Number of Fisher Scoring iterations: 2

data$Species <- relevel(data$Species, ref= "Gmed")
model1<-glm((Prev_Tspp) ~ Species,data=data, family=gaussian())
summary(model1)

##
## Call:
## glm(formula = (Prev_Tspp) ~ Species, family = gaussian(), data = data)
##
## Deviance Residuals:
## Min 1Q Median 3Q Max
## -53.02 -23.57 -14.97 21.89 76.43
##
## Coefficients:
## Estimate Std. Error t value Pr(>|t|)
## (Intercept) 43.195 16.417 2.631 0.00943 **
## SpeciesGff -25.478 25.078 -1.016 0.31132
## SpeciesGb -26.129 22.026 -1.186 0.23744
## SpeciesGa -28.225 19.731 -1.430 0.15472
## SpeciesGmm -19.064 20.107 -0.948 0.34464
## SpeciesGmsm -27.161 22.026 -1.233 0.21951
## SpeciesGp -7.176 17.913 -0.401 0.68928
## SpeciesGpg -19.629 16.855 -1.165 0.24608
## SpeciesGpp -40.335 36.710 -1.099 0.27369
## SpeciesGt 9.827 17.635 0.557 0.57822
## ---
## Signif. codes: 0 '***' 0.001 '**' 0.01 '*' 0.05 '.' 0.1 ' ' 1
##
## (Dispersion parameter for gaussian family taken to be 1078.113)
##
## Null deviance: 180975 on 155 degrees of freedom
## Residual deviance: 157405 on 146 degrees of freedom
## AIC: 1543.7
##
## Number of Fisher Scoring iterations: 2

data$Species <- relevel(data$Species, ref= "Gmm")
model1<-glm((Prev_Tspp) ~ Species,data=data, family=gaussian())
summary(model1)

##
## Call:
## glm(formula = (Prev_Tspp) ~ Species, family = gaussian(), data = data)
##
## Deviance Residuals:
## Min 1Q Median 3Q Max
## -53.02 -23.57 -14.97 21.89 76.43
##
## Coefficients:
## Estimate Std. Error t value Pr(>|t|)
## (Intercept) 24.1312 11.6088 2.079 0.0394 *
## SpeciesGmed 19.0637 20.1070 0.948 0.3446
## SpeciesGff -6.4146 22.2292 -0.289 0.7733
## SpeciesGb -7.0652 18.7186 -0.377 0.7064
## SpeciesGa -9.1612 15.9548 -0.574 0.5667
## SpeciesGmsm -8.0972 18.7186 -0.433 0.6660
## SpeciesGp 11.8873 13.6420 0.871 0.3850
## SpeciesGpg -0.5657 12.2202 -0.046 0.9631
## SpeciesGpp -21.2713 34.8264 -0.611 0.5423
## SpeciesGt 28.8907 13.2752 2.176 0.0311 *
## ---
## Signif. codes: 0 '***' 0.001 '**' 0.01 '*' 0.05 '.' 0.1 ' ' 1
##
## (Dispersion parameter for gaussian family taken to be 1078.113)
##
## Null deviance: 180975 on 155 degrees of freedom
## Residual deviance: 157405 on 146 degrees of freedom
## AIC: 1543.7
##
## Number of Fisher Scoring iterations: 2

data$Species <- relevel(data$Species, ref= "Gmsm")
model1<-glm((Prev_Tspp) ~ Species,data=data, family=gaussian())
summary(model1)

##
## Call:
## glm(formula = (Prev_Tspp) ~ Species, family = gaussian(), data = data)
##
## Deviance Residuals:
## Min 1Q Median 3Q Max
## -53.02 -23.57 -14.97 21.89 76.43
##
## Coefficients:
## Estimate Std. Error t value Pr(>|t|)
## (Intercept) 16.034 14.684 1.092 0.2767
## SpeciesGmm 8.097 18.719 0.433 0.6660
## SpeciesGmed 27.161 22.026 1.233 0.2195
## SpeciesGff 1.683 23.979 0.070 0.9442
## SpeciesGb 1.032 20.766 0.050 0.9604
## SpeciesGa -1.064 18.314 -0.058 0.9538
## SpeciesGp 19.985 16.339 1.223 0.2233
## SpeciesGpg 7.532 15.172 0.496 0.6204
## SpeciesGpp -13.174 35.969 -0.366 0.7147
## SpeciesGt 36.988 16.034 2.307 0.0225 *
## ---
## Signif. codes: 0 '***' 0.001 '**' 0.01 '*' 0.05 '.' 0.1 ' ' 1
##
## (Dispersion parameter for gaussian family taken to be 1078.113)
##
## Null deviance: 180975 on 155 degrees of freedom
## Residual deviance: 157405 on 146 degrees of freedom
## AIC: 1543.7
##
## Number of Fisher Scoring iterations: 2

data$Species <- relevel(data$Species, ref= "Gp")
model1<-glm((Prev_Tspp) ~ Species,data=data, family=gaussian())
summary(model1)

##
## Call:
## glm(formula = (Prev_Tspp) ~ Species, family = gaussian(), data = data)
##
## Deviance Residuals:
## Min 1Q Median 3Q Max
## -53.02 -23.57 -14.97 21.89 76.43
##
## Coefficients:
## Estimate Std. Error t value Pr(>|t|)
## (Intercept) 36.019 7.165 5.027 1.44e-06 ***
## SpeciesGmsm -19.985 16.339 -1.223 0.2233
## SpeciesGmm -11.887 13.642 -0.871 0.3850
## SpeciesGmed 7.176 17.913 0.401 0.6893
## SpeciesGff -18.302 20.266 -0.903 0.3680
## SpeciesGb -18.953 16.339 -1.160 0.2480
## SpeciesGa -21.049 13.082 -1.609 0.1098
## SpeciesGpg -12.453 8.118 -1.534 0.1272
## SpeciesGpp -33.159 33.607 -0.987 0.3254
## SpeciesGt 17.003 9.634 1.765 0.0797 .
## ---
## Signif. codes: 0 '***' 0.001 '**' 0.01 '*' 0.05 '.' 0.1 ' ' 1
##
## (Dispersion parameter for gaussian family taken to be 1078.113)
##
## Null deviance: 180975 on 155 degrees of freedom
## Residual deviance: 157405 on 146 degrees of freedom
## AIC: 1543.7
##
## Number of Fisher Scoring iterations: 2

data$Species <- relevel(data$Species, ref= "Gpg")
model1<-glm((Prev_Tspp) ~ Species,data=data, family=gaussian())
summary(model1)

##
## Call:
## glm(formula = (Prev_Tspp) ~ Species, family = gaussian(), data = data)
##
## Deviance Residuals:
## Min 1Q Median 3Q Max
## -53.02 -23.57 -14.97 21.89 76.43
##
## Coefficients:
## Estimate Std. Error t value Pr(>|t|)
## (Intercept) 23.5655 3.8169 6.174 6.26e-09 ***
## SpeciesGp 12.4530 8.1184 1.534 0.127210
## SpeciesGmsm -7.5315 15.1721 -0.496 0.620353
## SpeciesGmm 0.5657 12.2202 0.046 0.963140
## SpeciesGmed 19.6295 16.8552 1.165 0.246083
## SpeciesGff -5.8489 19.3375 -0.302 0.762730
## SpeciesGb -6.4995 15.1721 -0.428 0.669000
## SpeciesGa -8.5955 11.5914 -0.742 0.459553
## SpeciesGpp -20.7055 33.0557 -0.626 0.532042
## SpeciesGt 29.4564 7.4857 3.935 0.000128 ***
## ---
## Signif. codes: 0 '***' 0.001 '**' 0.01 '*' 0.05 '.' 0.1 ' ' 1
##
## (Dispersion parameter for gaussian family taken to be 1078.113)
##
## Null deviance: 180975 on 155 degrees of freedom
## Residual deviance: 157405 on 146 degrees of freedom
## AIC: 1543.7
##
## Number of Fisher Scoring iterations: 2

data$Species <- relevel(data$Species, ref= "Gpp")
model1<-glm((Prev_Tspp) ~ Species,data=data, family=gaussian())
summary(model1)

##
## Call:
## glm(formula = (Prev_Tspp) ~ Species, family = gaussian(), data = data)
##
## Deviance Residuals:
## Min 1Q Median 3Q Max
## -53.02 -23.57 -14.97 21.89 76.43
##
## Coefficients:
## Estimate Std. Error t value Pr(>|t|)
## (Intercept) 2.86 32.84 0.087 0.931
## SpeciesGpg 20.71 33.06 0.626 0.532
## SpeciesGp 33.16 33.61 0.987 0.325
## SpeciesGmsm 13.17 35.97 0.366 0.715
## SpeciesGmm 21.27 34.83 0.611 0.542
## SpeciesGmed 40.34 36.71 1.099 0.274
## SpeciesGff 14.86 37.91 0.392 0.696
## SpeciesGb 14.21 35.97 0.395 0.693
## SpeciesGa 12.11 34.61 0.350 0.727
## SpeciesGt 50.16 33.46 1.499 0.136
##
## (Dispersion parameter for gaussian family taken to be 1078.113)
##
## Null deviance: 180975 on 155 degrees of freedom
## Residual deviance: 157405 on 146 degrees of freedom
## AIC: 1543.7
##
## Number of Fisher Scoring iterations: 2

data$Species <- relevel(data$Species, ref= "Gt")
model1<-glm((Prev_Tspp) ~ Species,data=data, family=gaussian())
summary(model1)

##
## Call:
## glm(formula = (Prev_Tspp) ~ Species, family = gaussian(), data = data)
##
## Deviance Residuals:
## Min 1Q Median 3Q Max
## -53.02 -23.57 -14.97 21.89 76.43
##
## Coefficients:
## Estimate Std. Error t value Pr(>|t|)
## (Intercept) 53.022 6.439 8.234 9.33e-14 ***
## SpeciesGpp -50.162 33.460 -1.499 0.135992
## SpeciesGpg -29.456 7.486 -3.935 0.000128 ***
## SpeciesGp -17.003 9.634 -1.765 0.079651 .
## SpeciesGmsm -36.988 16.034 -2.307 0.022470 *
## SpeciesGmm -28.891 13.275 -2.176 0.031141 *
## SpeciesGmed -9.827 17.635 -0.557 0.578217
## SpeciesGff -35.305 20.021 -1.763 0.079922 .
## SpeciesGb -35.956 16.034 -2.242 0.026437 *
## SpeciesGa -38.052 12.699 -2.997 0.003210 **
## ---
## Signif. codes: 0 '***' 0.001 '**' 0.01 '*' 0.05 '.' 0.1 ' ' 1
##
## (Dispersion parameter for gaussian family taken to be 1078.113)
##
## Null deviance: 180975 on 155 degrees of freedom
## Residual deviance: 157405 on 146 degrees of freedom
## AIC: 1543.7
##
## Number of Fisher Scoring iterations: 2

#--------------------------------------------------------------
# for Sodalis

data$Species <- relevel(data$Species, ref= "Ga")
model1<-glm((Prev_Sod) ~ Species,data=data, family=gaussian())
summary(model1)

##
## Call:
## glm(formula = (Prev_Sod) ~ Species, family = gaussian(), data = data)
##
## Deviance Residuals:
## Min 1Q Median 3Q Max
## -31.116 -1.319 -1.319 0.000 94.421
##
## Coefficients:
## Estimate Std. Error t value Pr(>|t|)
## (Intercept) 3.1633 5.7738 0.548 0.58461
## SpeciesGt -3.1633 6.6990 -0.472 0.63748
## SpeciesGpp 8.2667 18.2583 0.453 0.65139
## SpeciesGpg -1.8440 6.1148 -0.302 0.76341
## SpeciesGp 27.9524 6.9010 4.050 8.27e-05 ***
## SpeciesGmsm -2.8253 9.6614 -0.292 0.77037
## SpeciesGmm 27.2617 8.4167 3.239 0.00149 **
## SpeciesGmed 1.5067 10.4088 0.145 0.88511
## SpeciesGff 11.3300 11.5476 0.981 0.32814
## SpeciesGb 0.4607 9.6614 0.048 0.96204
## ---
## Signif. codes: 0 '***' 0.001 '**' 0.01 '*' 0.05 '.' 0.1 ' ' 1
##
## (Dispersion parameter for gaussian family taken to be 300.0283)
##
## Null deviance: 64686 on 155 degrees of freedom
## Residual deviance: 43804 on 146 degrees of freedom
## AIC: 1344.2
##
## Number of Fisher Scoring iterations: 2

Anova(model1)

## Analysis of Deviance Table (Type II tests)
##
## Response: (Prev_Sod)
## LR Chisq Df Pr(>Chisq)
## Species 69.601 9 1.824e-11 ***
## ---
## Signif. codes: 0 '***' 0.001 '**' 0.01 '*' 0.05 '.' 0.1 ' ' 1

data$Species <- relevel(data$Species, ref= "Gb")
model1<-glm((Prev_Sod) ~ Species,data=data, family=gaussian())
summary(model1)

##
## Call:
## glm(formula = (Prev_Sod) ~ Species, family = gaussian(), data = data)
##
## Deviance Residuals:
## Min 1Q Median 3Q Max
## -31.116 -1.319 -1.319 0.000 94.421
##
## Coefficients:
## Estimate Std. Error t value Pr(>|t|)
## (Intercept) 3.6240 7.7463 0.468 0.64060
## SpeciesGa -0.4607 9.6614 -0.048 0.96204
## SpeciesGt -3.6240 8.4584 -0.428 0.66896
## SpeciesGpp 7.8060 18.9746 0.411 0.68139
## SpeciesGpg -2.3047 8.0038 -0.288 0.77379
## SpeciesGp 27.4917 8.6193 3.190 0.00174 **
## SpeciesGmsm -3.2860 10.9550 -0.300 0.76464
## SpeciesGmm 26.8010 9.8747 2.714 0.00745 **
## SpeciesGmed 1.0460 11.6195 0.090 0.92839
## SpeciesGff 10.8693 12.6497 0.859 0.39161
## ---
## Signif. codes: 0 '***' 0.001 '**' 0.01 '*' 0.05 '.' 0.1 ' ' 1
##
## (Dispersion parameter for gaussian family taken to be 300.0283)
##
## Null deviance: 64686 on 155 degrees of freedom
## Residual deviance: 43804 on 146 degrees of freedom
## AIC: 1344.2
##
## Number of Fisher Scoring iterations: 2

data$Species <- relevel(data$Species, ref= "Gff")
model1<-glm((Prev_Sod) ~ Species,data=data, family=gaussian())
summary(model1)

##
## Call:
## glm(formula = (Prev_Sod) ~ Species, family = gaussian(), data = data)
##
## Deviance Residuals:
## Min 1Q Median 3Q Max
## -31.116 -1.319 -1.319 0.000 94.421
##
## Coefficients:
## Estimate Std. Error t value Pr(>|t|)
## (Intercept) 14.493 10.000 1.449 0.149
## SpeciesGb -10.869 12.650 -0.859 0.392
## SpeciesGa -11.330 11.548 -0.981 0.328
## SpeciesGt -14.493 10.562 -1.372 0.172
## SpeciesGpp -3.063 20.001 -0.153 0.878
## SpeciesGpg -13.174 10.201 -1.291 0.199
## SpeciesGp 16.622 10.691 1.555 0.122
## SpeciesGmsm -14.155 12.650 -1.119 0.265
## SpeciesGmm 15.932 11.727 1.359 0.176
## SpeciesGmed -9.823 13.229 -0.743 0.459
##
## (Dispersion parameter for gaussian family taken to be 300.0283)
##
## Null deviance: 64686 on 155 degrees of freedom
## Residual deviance: 43804 on 146 degrees of freedom
## AIC: 1344.2
##
## Number of Fisher Scoring iterations: 2

data$Species <- relevel(data$Species, ref= "Gmed")
model1<-glm((Prev_Sod) ~ Species,data=data, family=gaussian())
summary(model1)

##
## Call:
## glm(formula = (Prev_Sod) ~ Species, family = gaussian(), data = data)
##
## Deviance Residuals:
## Min 1Q Median 3Q Max
## -31.116 -1.319 -1.319 0.000 94.421
##
## Coefficients:
## Estimate Std. Error t value Pr(>|t|)
## (Intercept) 4.670 8.661 0.539 0.59056
## SpeciesGff 9.823 13.229 0.743 0.45895
## SpeciesGb -1.046 11.619 -0.090 0.92839
## SpeciesGa -1.507 10.409 -0.145 0.88511
## SpeciesGt -4.670 9.303 -0.502 0.61643
## SpeciesGpp 6.760 19.366 0.349 0.72754
## SpeciesGpg -3.351 8.892 -0.377 0.70684
## SpeciesGp 26.446 9.450 2.799 0.00583 **
## SpeciesGmsm -4.332 11.619 -0.373 0.70982
## SpeciesGmm 25.755 10.607 2.428 0.01640 *
## ---
## Signif. codes: 0 '***' 0.001 '**' 0.01 '*' 0.05 '.' 0.1 ' ' 1
##
## (Dispersion parameter for gaussian family taken to be 300.0283)
##
## Null deviance: 64686 on 155 degrees of freedom
## Residual deviance: 43804 on 146 degrees of freedom
## AIC: 1344.2
##
## Number of Fisher Scoring iterations: 2

data$Species <- relevel(data$Species, ref= "Gmm")
model1<-glm((Prev_Sod) ~ Species,data=data, family=gaussian())
summary(model1)

##
## Call:
## glm(formula = (Prev_Sod) ~ Species, family = gaussian(), data = data)
##
## Deviance Residuals:
## Min 1Q Median 3Q Max
## -31.116 -1.319 -1.319 0.000 94.421
##
## Coefficients:
## Estimate Std. Error t value Pr(>|t|)
## (Intercept) 30.4250 6.1240 4.968 1.87e-06 ***
## SpeciesGmed -25.7550 10.6071 -2.428 0.01640 *
## SpeciesGff -15.9317 11.7266 -1.359 0.17637
## SpeciesGb -26.8010 9.8747 -2.714 0.00745 **
## SpeciesGa -27.2617 8.4167 -3.239 0.00149 **
## SpeciesGt -30.4250 7.0031 -4.345 2.60e-05 ***
## SpeciesGpp -18.9950 18.3720 -1.034 0.30289
## SpeciesGpg -29.1057 6.4465 -4.515 1.30e-05 ***
## SpeciesGp 0.6907 7.1966 0.096 0.92367
## SpeciesGmsm -30.0870 9.8747 -3.047 0.00274 **
## ---
## Signif. codes: 0 '***' 0.001 '**' 0.01 '*' 0.05 '.' 0.1 ' ' 1
##
## (Dispersion parameter for gaussian family taken to be 300.0283)
##
## Null deviance: 64686 on 155 degrees of freedom
## Residual deviance: 43804 on 146 degrees of freedom
## AIC: 1344.2
##
## Number of Fisher Scoring iterations: 2

data$Species <- relevel(data$Species, ref= "Gmsm")
model1<-glm((Prev_Sod) ~ Species,data=data, family=gaussian())
summary(model1)

##
## Call:
## glm(formula = (Prev_Sod) ~ Species, family = gaussian(), data = data)
##
## Deviance Residuals:
## Min 1Q Median 3Q Max
## -31.116 -1.319 -1.319 0.000 94.421
##
## Coefficients:
## Estimate Std. Error t value Pr(>|t|)
## (Intercept) 0.3380 7.7463 0.044 0.965256
## SpeciesGmm 30.0870 9.8747 3.047 0.002745 **
## SpeciesGmed 4.3320 11.6195 0.373 0.709822
## SpeciesGff 14.1553 12.6497 1.119 0.264967
## SpeciesGb 3.2860 10.9550 0.300 0.764638
## SpeciesGa 2.8253 9.6614 0.292 0.770368
## SpeciesGt -0.3380 8.4584 -0.040 0.968180
## SpeciesGpp 11.0920 18.9746 0.585 0.559738
## SpeciesGpg 0.9813 8.0038 0.123 0.902586
## SpeciesGp 30.7777 8.6193 3.571 0.000482 ***
## ---
## Signif. codes: 0 '***' 0.001 '**' 0.01 '*' 0.05 '.' 0.1 ' ' 1
##
## (Dispersion parameter for gaussian family taken to be 300.0283)
##
## Null deviance: 64686 on 155 degrees of freedom
## Residual deviance: 43804 on 146 degrees of freedom
## AIC: 1344.2
##
## Number of Fisher Scoring iterations: 2

data$Species <- relevel(data$Species, ref= "Gp")
model1<-glm((Prev_Sod) ~ Species,data=data, family=gaussian())
summary(model1)

##
## Call:
## glm(formula = (Prev_Sod) ~ Species, family = gaussian(), data = data)
##
## Deviance Residuals:
## Min 1Q Median 3Q Max
## -31.116 -1.319 -1.319 0.000 94.421
##
## Coefficients:
## Estimate Std. Error t value Pr(>|t|)
## (Intercept) 31.1157 3.7798 8.232 9.43e-14 ***
## SpeciesGmsm -30.7777 8.6193 -3.571 0.000482 ***
## SpeciesGmm -0.6907 7.1966 -0.096 0.923669
## SpeciesGmed -26.4457 9.4496 -2.799 0.005826 **
## SpeciesGff -16.6224 10.6910 -1.555 0.122157
## SpeciesGb -27.4917 8.6193 -3.190 0.001744 **
## SpeciesGa -27.9524 6.9010 -4.050 8.27e-05 ***
## SpeciesGt -31.1157 5.0820 -6.123 8.08e-09 ***
## SpeciesGpp -19.6857 17.7289 -1.110 0.268664
## SpeciesGpg -29.7964 4.2827 -6.957 1.08e-10 ***
## ---
## Signif. codes: 0 '***' 0.001 '**' 0.01 '*' 0.05 '.' 0.1 ' ' 1
##
## (Dispersion parameter for gaussian family taken to be 300.0283)
##
## Null deviance: 64686 on 155 degrees of freedom
## Residual deviance: 43804 on 146 degrees of freedom
## AIC: 1344.2
##
## Number of Fisher Scoring iterations: 2

data$Species <- relevel(data$Species, ref= "Gpg")
model1<-glm((Prev_Sod) ~ Species,data=data, family=gaussian())
summary(model1)

##
## Call:
## glm(formula = (Prev_Sod) ~ Species, family = gaussian(), data = data)
##
## Deviance Residuals:
## Min 1Q Median 3Q Max
## -31.116 -1.319 -1.319 0.000 94.421
##
## Coefficients:
## Estimate Std. Error t value Pr(>|t|)
## (Intercept) 1.3193 2.0136 0.655 0.513
## SpeciesGp 29.7964 4.2827 6.957 1.08e-10 ***
## SpeciesGmsm -0.9813 8.0038 -0.123 0.903
## SpeciesGmm 29.1057 6.4465 4.515 1.30e-05 ***
## SpeciesGmed 3.3507 8.8917 0.377 0.707
## SpeciesGff 13.1740 10.2012 1.291 0.199
## SpeciesGb 2.3047 8.0038 0.288 0.774
## SpeciesGa 1.8440 6.1148 0.302 0.763
## SpeciesGt -1.3193 3.9489 -0.334 0.739
## SpeciesGpp 10.1107 17.4380 0.580 0.563
## ---
## Signif. codes: 0 '***' 0.001 '**' 0.01 '*' 0.05 '.' 0.1 ' ' 1
##
## (Dispersion parameter for gaussian family taken to be 300.0283)
##
## Null deviance: 64686 on 155 degrees of freedom
## Residual deviance: 43804 on 146 degrees of freedom
## AIC: 1344.2
##
## Number of Fisher Scoring iterations: 2

data$Species <- relevel(data$Species, ref= "Gpp")
model1<-glm((Prev_Sod) ~ Species,data=data, family=gaussian())
summary(model1)

##
## Call:
## glm(formula = (Prev_Sod) ~ Species, family = gaussian(), data = data)
##
## Deviance Residuals:
## Min 1Q Median 3Q Max
## -31.116 -1.319 -1.319 0.000 94.421
##
## Coefficients:
## Estimate Std. Error t value Pr(>|t|)
## (Intercept) 11.430 17.321 0.660 0.510
## SpeciesGpg -10.111 17.438 -0.580 0.563
## SpeciesGp 19.686 17.729 1.110 0.269
## SpeciesGmsm -11.092 18.975 -0.585 0.560
## SpeciesGmm 18.995 18.372 1.034 0.303
## SpeciesGmed -6.760 19.366 -0.349 0.728
## SpeciesGff 3.063 20.001 0.153 0.878
## SpeciesGb -7.806 18.975 -0.411 0.681
## SpeciesGa -8.267 18.258 -0.453 0.651
## SpeciesGt -11.430 17.651 -0.648 0.518
##
## (Dispersion parameter for gaussian family taken to be 300.0283)
##
## Null deviance: 64686 on 155 degrees of freedom
## Residual deviance: 43804 on 146 degrees of freedom
## AIC: 1344.2
##
## Number of Fisher Scoring iterations: 2

data$Species <- relevel(data$Species, ref= "Gt")
model1<-glm((Prev_Sod) ~ Species,data=data, family=gaussian())
summary(model1)

##
## Call:
## glm(formula = (Prev_Sod) ~ Species, family = gaussian(), data = data)
##
## Deviance Residuals:
## Min 1Q Median 3Q Max
## -31.116 -1.319 -1.319 0.000 94.421
##
## Coefficients:
## Estimate Std. Error t value Pr(>|t|)
## (Intercept) 3.508e-14 3.397e+00 0.000 1.000
## SpeciesGpp 1.143e+01 1.765e+01 0.648 0.518
## SpeciesGpg 1.319e+00 3.949e+00 0.334 0.739
## SpeciesGp 3.112e+01 5.082e+00 6.123 8.08e-09 ***
## SpeciesGmsm 3.380e-01 8.458e+00 0.040 0.968
## SpeciesGmm 3.042e+01 7.003e+00 4.345 2.60e-05 ***
## SpeciesGmed 4.670e+00 9.303e+00 0.502 0.616
## SpeciesGff 1.449e+01 1.056e+01 1.372 0.172
## SpeciesGb 3.624e+00 8.458e+00 0.428 0.669
## SpeciesGa 3.163e+00 6.699e+00 0.472 0.637
## ---
## Signif. codes: 0 '***' 0.001 '**' 0.01 '*' 0.05 '.' 0.1 ' ' 1
##
## (Dispersion parameter for gaussian family taken to be 300.0283)
##
## Null deviance: 64686 on 155 degrees of freedom
## Residual deviance: 43804 on 146 degrees of freedom
## AIC: 1344.2
##
## Number of Fisher Scoring iterations: 2

## Statistics for table 4

# species with significant differences between countries

data_ga <- subset(data, Species=="Ga")
data_ga

## Country Localisation Species Sex Sample Prev_Sod Prev_Tspp
## 27 SAF False Bay Park Ga NI 27 0.00 2.60
## 41 SAF SAFint Lucia Ga NI 41 0.00 38.60
## 46 SAF Lower Mkhuze Ga NI 46 0.00 43.40
## 53 MOZ Reserva Especial de Maputo Ga NI 53 0.00 10.00
## 59 SAF North eastern KwaZulu-Natal Ga NI 59 5.13 5.13
## 75 SWA Mlawula Nature Reserve Ga NI 75 0.00 0.00
## 76 URT Uguja island Ga NI 76 6.67 10.00
## 132 URT Zanzibar Ga NI 132 16.67 0.00
## 141 URT Jozani Ga NI 141 0.00 25.00
## Prev_Tc Prev_Tv Prev_Tz Prev_Tsg Prev_TcTv Prev_TcTz Prev_TcTsg Prev_TvTz
## 27 2.60 0.00 0.00 0.00 0.00 0 0.00 0
## 41 10.53 0.00 10.53 15.79 0.00 0 1.75 0
## 46 9.43 1.89 3.77 24.53 1.89 0 1.89 0
## 53 6.00 0.00 0.00 4.00 0.00 0 0.00 0
## 59 2.56 0.00 0.00 2.56 0.00 0 0.00 0
## 75 0.00 0.00 0.00 0.00 0.00 0 0.00 0
## 76 0.00 6.67 3.33 0.00 0.00 0 0.00 0
## 132 0.00 0.00 0.00 0.00 0.00 0 0.00 0
## 141 0.00 0.00 25.00 0.00 0.00 0 0.00 0
## Prev_TvTsg Prev_TzTsg Prev_TcTvTz
## 27 0 0 0
## 41 0 0 0
## 46 0 0 0
## 53 0 0 0
## 59 0 0 0
## 75 0 0 0
## 76 0 0 0
## 132 0 0 0
## 141 0 0 0

model1<-glm((Prev_Tspp) ~ Country,data=data_ga, family=gaussian())
summary(model1)

##
## Call:
## glm(formula = (Prev_Tspp) ~ Country, family = gaussian(), data = data_ga)
##
## Deviance Residuals:
## 27 41 46 53 59 75 76 132
## -19.832 16.168 20.968 0.000 -17.302 0.000 -1.667 -11.667
## 141
## 13.333
##
## Coefficients:
## Estimate Std. Error t value Pr(>|t|)
## (Intercept) 22.433 9.248 2.426 0.0597 .
## CountryURT -10.766 14.126 -0.762 0.4804
## CountrySWA -22.433 20.678 -1.085 0.3275
## CountryMOZ -12.432 20.678 -0.601 0.5739
## ---
## Signif. codes: 0 '***' 0.001 '**' 0.01 '*' 0.05 '.' 0.1 ' ' 1
##
## (Dispersion parameter for gaussian family taken to be 342.0791)
##
## Null deviance: 2214.7 on 8 degrees of freedom
## Residual deviance: 1710.4 on 5 degrees of freedom
## AIC: 82.766
##
## Number of Fisher Scoring iterations: 2

Anova(model1)

## Analysis of Deviance Table (Type II tests)
##
## Response: (Prev_Tspp)
## LR Chisq Df Pr(>Chisq)
## Country 1.4742 3 0.6882

model2<-glm((Prev_Sod) ~ Country,data=data_ga, family=gaussian())
summary(model2)

##
## Call:
## glm(formula = (Prev_Sod) ~ Country, family = gaussian(), data = data_ga)
##
## Deviance Residuals:
## 27 41 46 53 59 75 76 132 141
## -1.282 -1.282 -1.282 0.000 3.848 0.000 -1.110 8.890 -7.780
##
## Coefficients:
## Estimate Std. Error t value Pr(>|t|)
## (Intercept) 1.282 2.833 0.453 0.670
## CountryURT 6.498 4.328 1.501 0.194
## CountrySWA -1.282 6.335 -0.202 0.848
## CountryMOZ -1.282 6.335 -0.202 0.848
##
## (Dispersion parameter for gaussian family taken to be 32.10606)
##
## Null deviance: 258.63 on 8 degrees of freedom
## Residual deviance: 160.53 on 5 degrees of freedom
## AIC: 61.472
##
## Number of Fisher Scoring iterations: 2

Anova(model2)

## Analysis of Deviance Table (Type II tests)
##
## Response: (Prev_Sod)
## LR Chisq Df Pr(>Chisq)
## Country 3.0556 3 0.3831

#-----------------------------------------------

data_gb <- subset(data, Species=="Gb")
data_gb

## Country Localisation Species Sex Sample Prev_Sod Prev_Tspp
## 7 SAF Phinda Gb NI 7 4.12 0.00
## 50 SAF Hluhluwe Gb NI 50 0.00 32.00
## 51 SAF North eastern KwaZulu-Natal Gb NI 51 0.00 4.00
## 52 MOZ Reserva Especial de Maputo Gb NI 52 14.00 6.00
## 74 SAF SAFint Lucia Gb NI 74 0.00 43.33
## Prev_Tc Prev_Tv Prev_Tz Prev_Tsg Prev_TcTv Prev_TcTz Prev_TcTsg Prev_TvTz
## 7 0 0.00 0.00 0.00 0 0 0 0
## 50 12 2.00 14.00 4.00 0 0 0 0
## 51 2 0.00 0.00 2.00 0 0 0 0
## 52 0 2.00 0.00 2.00 0 0 0 0
## 74 0 3.33 3.33 36.67 0 0 0 0
## Prev_TvTsg Prev_TzTsg Prev_TcTvTz
## 7 0 0 0
## 50 0 0 0
## 51 0 0 0
## 52 2 0 0
## 74 0 0 0

model1<-glm((Prev_Tspp) ~ Country,data=data_gb, family=gaussian())
summary(model1)

##
## Call:
## glm(formula = (Prev_Tspp) ~ Country, family = gaussian(), data = data_gb)
##
## Deviance Residuals:
## 7 50 51 52 74
## -19.83 12.17 -15.83 0.00 23.50
##
## Coefficients:
## Estimate Std. Error t value Pr(>|t|)
## (Intercept) 19.83 10.58 1.874 0.158
## CountryMOZ -13.83 23.67 -0.584 0.600
##
## (Dispersion parameter for gaussian family taken to be 448.0589)
##
## Null deviance: 1497.2 on 4 degrees of freedom
## Residual deviance: 1344.2 on 3 degrees of freedom
## AIC: 48.16
##
## Number of Fisher Scoring iterations: 2

Anova(model1)

## Analysis of Deviance Table (Type II tests)
##
## Response: (Prev_Tspp)
## LR Chisq Df Pr(>Chisq)
## Country 0.34163 1 0.5589

model2<-glm((Prev_Sod) ~ Country,data=data_gb, family=gaussian())
summary(model2)

##
## Call:
## glm(formula = (Prev_Sod) ~ Country, family = gaussian(), data = data_gb)
##
## Deviance Residuals:
## 7 50 51 52 74
## 3.09 -1.03 -1.03 0.00 -1.03
##
## Coefficients:
## Estimate Std. Error t value Pr(>|t|)
## (Intercept) 1.030 1.030 1.000 0.3910
## CountryMOZ 12.970 2.303 5.631 0.0111 *
## ---
## Signif. codes: 0 '***' 0.001 '**' 0.01 '*' 0.05 '.' 0.1 ' ' 1
##
## (Dispersion parameter for gaussian family taken to be 4.2436)
##
## Null deviance: 147.308 on 4 degrees of freedom
## Residual deviance: 12.731 on 3 degrees of freedom
## AIC: 24.862
##
## Number of Fisher Scoring iterations: 2

Anova(model2)

## Analysis of Deviance Table (Type II tests)
##
## Response: (Prev_Sod)
## LR Chisq Df Pr(>Chisq)
## Country 31.713 1 1.787e-08 ***
## ---
## Signif. codes: 0 '***' 0.001 '**' 0.01 '*' 0.05 '.' 0.1 ' ' 1

#-------------------------------------------------------------------------
data_gff <- subset(data, Species=="Gff")
data_gff

## Country Localisation Species Sex Sample Prev_Sod Prev_Tspp Prev_Tc Prev_Tv
## 16 UGA Buvuma island Gff NI 16 4.26 10.64 3.19 2.13
## 49 KEN Ikapolok Gff NI 49 39.22 37.25 1.96 9.80
## 61 KEN Obekai Gff NI 61 0.00 5.26 2.63 2.63
## Prev_Tz Prev_Tsg Prev_TcTv Prev_TcTz Prev_TcTsg Prev_TvTz Prev_TvTsg
## 16 1.06 3.19 0.00 0.00 1.06 0 0
## 49 0.00 19.61 3.92 1.96 0.00 0 0
## 61 0.00 0.00 0.00 0.00 0.00 0 0
## Prev_TzTsg Prev_TcTvTz
## 16 0 0
## 49 0 0
## 61 0 0

model1<-glm((Prev_Tspp) ~ Country,data=data_gff, family=gaussian())
summary(model1)

##
## Call:
## glm(formula = (Prev_Tspp) ~ Country, family = gaussian(), data = data_gff)
##
## Deviance Residuals:
## 16 49 61
## 0.00 15.99 -15.99
##
## Coefficients:
## Estimate Std. Error t value Pr(>|t|)
## (Intercept) 10.64 22.62 0.470 0.720
## CountryKEN 10.61 27.70 0.383 0.767
##
## (Dispersion parameter for gaussian family taken to be 511.68)
##
## Null deviance: 586.80 on 2 degrees of freedom
## Residual deviance: 511.68 on 1 degrees of freedom
## AIC: 29.931
##
## Number of Fisher Scoring iterations: 2

Anova(model1)

## Analysis of Deviance Table (Type II tests)
##
## Response: (Prev_Tspp)
## LR Chisq Df Pr(>Chisq)
## Country 0.14681 1 0.7016

model2<-glm((Prev_Sod) ~ Country,data=data_gff, family=gaussian())
summary(model2)

##
## Call:
## glm(formula = (Prev_Sod) ~ Country, family = gaussian(), data = data_gff)
##
## Deviance Residuals:
## 16 49 61
## 0.00 19.61 -19.61
##
## Coefficients:
## Estimate Std. Error t value Pr(>|t|)
## (Intercept) 4.26 27.73 0.154 0.903
## CountryKEN 15.35 33.97 0.452 0.730
##
## (Dispersion parameter for gaussian family taken to be 769.1042)
##
## Null deviance: 926.19 on 2 degrees of freedom
## Residual deviance: 769.10 on 1 degrees of freedom
## AIC: 31.153
##
## Number of Fisher Scoring iterations: 2

Anova(model2)

## Analysis of Deviance Table (Type II tests)
##
## Response: (Prev_Sod)
## LR Chisq Df Pr(>Chisq)
## Country 0.20424 1 0.6513

#-------------------------------------------------------------------------
data_gmm <- subset(data, Species=="Gmm")
data_gmm

## Country Localisation Species Sex Sample Prev_Sod Prev_Tspp Prev_Tc Prev_Tv
## 21 KEN Kari Gmm NI 21 63.53 2.35 2.35 0.00
## 22 URT MaSAFng-URTga Gmm NI 22 76.54 53.09 18.52 1.23
## 26 ZIM Makuti Gmm NI 26 26.92 91.03 11.54 0.00
## 93 ZIM Kemukura Gmm NI 93 22.22 5.56 0.00 5.56
## 100 ZIM Rukomeshi Gmm NI 100 20.00 0.00 0.00 0.00
## 105 ZIM Mukondore Gmm NI 105 23.08 7.69 7.69 0.00
## 115 ZIM M. chiuyi Gmm NI 115 11.11 0.00 0.00 0.00
## 131 ZIM Mushumb Gmm NI 131 0.00 33.33 16.67 0.00
## Prev_Tz Prev_Tsg Prev_TcTv Prev_TcTz Prev_TcTsg Prev_TvTz Prev_TvTsg
## 21 0.00 0.00 0 0 0.00 0 0.00
## 22 1.23 18.52 0 0 6.17 0 6.17
## 26 2.56 69.23 0 0 7.69 0 0.00
## 93 0.00 0.00 0 0 0.00 0 0.00
## 100 0.00 0.00 0 0 0.00 0 0.00
## 105 0.00 0.00 0 0 0.00 0 0.00
## 115 0.00 0.00 0 0 0.00 0 0.00
## 131 0.00 16.67 0 0 0.00 0 0.00
## Prev_TzTsg Prev_TcTvTz
## 21 0.00 0
## 22 1.23 0
## 26 0.00 0
## 93 0.00 0
## 100 0.00 0
## 105 0.00 0
## 115 0.00 0
## 131 0.00 0

model1<-glm((Prev_Tspp) ~ Country,data=data_gmm, family=gaussian())
summary(model1)

##
## Call:
## glm(formula = (Prev_Tspp) ~ Country, family = gaussian(), data = data_gmm)
##
## Deviance Residuals:
## 21 22 26 93 100 105 115 131
## 0.00 0.00 68.09 -17.38 -22.93 -15.24 -22.93 10.39
##
## Coefficients:
## Estimate Std. Error t value Pr(>|t|)
## (Intercept) 22.93 14.53 1.579 0.175
## CountryURT 30.15 38.44 0.785 0.468
## CountryKEN -20.59 38.44 -0.536 0.615
##
## (Dispersion parameter for gaussian family taken to be 1266.263)
##
## Null deviance: 7652.9 on 7 degrees of freedom
## Residual deviance: 6331.3 on 5 degrees of freedom
## AIC: 84.094
##
## Number of Fisher Scoring iterations: 2

Anova(model1)

## Analysis of Deviance Table (Type II tests)
##
## Response: (Prev_Tspp)
## LR Chisq Df Pr(>Chisq)
## Country 1.0437 2 0.5934

model2<-glm((Prev_Sod) ~ Country,data=data_gmm, family=gaussian())
summary(model2)

##
## Call:
## glm(formula = (Prev_Sod) ~ Country, family = gaussian(), data = data_gmm)
##
## Deviance Residuals:
## 21 22 26 93 100 105 115 131
## 0.000 0.000 9.698 4.998 2.778 5.858 -6.112 -17.222
##
## Coefficients:
## Estimate Std. Error t value Pr(>|t|)
## (Intercept) 17.222 4.062 4.240 0.00817 **
## CountryURT 59.318 10.747 5.519 0.00267 **
## CountryKEN 46.308 10.747 4.309 0.00765 **
## ---
## Signif. codes: 0 '***' 0.001 '**' 0.01 '*' 0.05 '.' 0.1 ' ' 1
##
## (Dispersion parameter for gaussian family taken to be 99.0037)
##
## Null deviance: 4763.52 on 7 degrees of freedom
## Residual deviance: 495.02 on 5 degrees of freedom
## AIC: 63.704
##
## Number of Fisher Scoring iterations: 2

Anova(model2)

## Analysis of Deviance Table (Type II tests)
##
## Response: (Prev_Sod)
## LR Chisq Df Pr(>Chisq)
## Country 43.115 2 4.343e-10 ***
## ---
## Signif. codes: 0 '***' 0.001 '**' 0.01 '*' 0.05 '.' 0.1 ' ' 1

data_gmm$Country <- relevel(data_gmm$Country, ref= "URT")
model2<-glm((Prev_Sod) ~ Country,data=data_gmm, family=gaussian())
summary(model2)

##
## Call:
## glm(formula = (Prev_Sod) ~ Country, family = gaussian(), data = data_gmm)
##
## Deviance Residuals:
## 21 22 26 93 100 105 115 131
## 0.000 0.000 9.698 4.998 2.778 5.858 -6.112 -17.222
##
## Coefficients:
## Estimate Std. Error t value Pr(>|t|)
## (Intercept) 76.54 9.95 7.692 0.000592 ***
## CountryZIM -59.32 10.75 -5.519 0.002673 **
## CountryKEN -13.01 14.07 -0.925 0.397621
## ---
## Signif. codes: 0 '***' 0.001 '**' 0.01 '*' 0.05 '.' 0.1 ' ' 1
##
## (Dispersion parameter for gaussian family taken to be 99.0037)
##
## Null deviance: 4763.52 on 7 degrees of freedom
## Residual deviance: 495.02 on 5 degrees of freedom
## AIC: 63.704
##
## Number of Fisher Scoring iterations: 2

Anova(model2)

## Analysis of Deviance Table (Type II tests)
##
## Response: (Prev_Sod)
## LR Chisq Df Pr(>Chisq)
## Country 43.115 2 4.343e-10 ***
## ---
## Signif. codes: 0 '***' 0.001 '**' 0.01 '*' 0.05 '.' 0.1 ' ' 1

data_gmm$Country <- relevel(data_gmm$Country, ref= "ZAM")
model2<-glm((Prev_Sod) ~ Country,data=data_gmm, family=gaussian())
summary(model2)

##
## Call:
## glm(formula = (Prev_Sod) ~ Country, family = gaussian(), data = data_gmm)
##
## Deviance Residuals:
## 21 22 26 93 100 105 115 131
## 0.000 0.000 9.698 4.998 2.778 5.858 -6.112 -17.222
##
## Coefficients:
## Estimate Std. Error t value Pr(>|t|)
## (Intercept) 76.54 9.95 7.692 0.000592 ***
## CountryZIM -59.32 10.75 -5.519 0.002673 **
## CountryKEN -13.01 14.07 -0.925 0.397621
## ---
## Signif. codes: 0 '***' 0.001 '**' 0.01 '*' 0.05 '.' 0.1 ' ' 1
##
## (Dispersion parameter for gaussian family taken to be 99.0037)
##
## Null deviance: 4763.52 on 7 degrees of freedom
## Residual deviance: 495.02 on 5 degrees of freedom
## AIC: 63.704
##
## Number of Fisher Scoring iterations: 2

Anova(model2)

## Analysis of Deviance Table (Type II tests)
##
## Response: (Prev_Sod)
## LR Chisq Df Pr(>Chisq)
## Country 43.115 2 4.343e-10 ***
## ---
## Signif. codes: 0 '***' 0.001 '**' 0.01 '*' 0.05 '.' 0.1 ' ' 1

data_gmm$Country <- relevel(data_gmm$Country, ref= "ZIM")
model2<-glm((Prev_Sod) ~ Country,data=data_gmm, family=gaussian())
summary(model2)

##
## Call:
## glm(formula = (Prev_Sod) ~ Country, family = gaussian(), data = data_gmm)
##
## Deviance Residuals:
## 21 22 26 93 100 105 115 131
## 0.000 0.000 9.698 4.998 2.778 5.858 -6.112 -17.222
##
## Coefficients:
## Estimate Std. Error t value Pr(>|t|)
## (Intercept) 17.222 4.062 4.240 0.00817 **
## CountryURT 59.318 10.747 5.519 0.00267 **
## CountryKEN 46.308 10.747 4.309 0.00765 **
## ---
## Signif. codes: 0 '***' 0.001 '**' 0.01 '*' 0.05 '.' 0.1 ' ' 1
##
## (Dispersion parameter for gaussian family taken to be 99.0037)
##
## Null deviance: 4763.52 on 7 degrees of freedom
## Residual deviance: 495.02 on 5 degrees of freedom
## AIC: 63.704
##
## Number of Fisher Scoring iterations: 2

Anova(model2)

## Analysis of Deviance Table (Type II tests)
##
## Response: (Prev_Sod)
## LR Chisq Df Pr(>Chisq)
## Country 43.115 2 4.343e-10 ***
## ---
## Signif. codes: 0 '***' 0.001 '**' 0.01 '*' 0.05 '.' 0.1 ' ' 1

#----------------------------------------------------------------
#Gp

data_gp <- subset(data, Species=="Gp")
data_gp

## Country Localisation Species Sex Sample Prev_Sod Prev_Tspp Prev_Tc
## 1 ETH Arba minch, nech SAFr Gp NI 1 20.48 20.04 6.10
## 5 KEN Katotoi Gp NI 5 42.56 88.72 21.54
## 6 KEN Mwea Gp NI 6 3.08 19.49 7.18
## 8 ZAM Mfuwe Gp NI 8 2.05 45.21 1.37
## 9 URT URTzania Gp NI 9 78.77 19.86 6.16
## 17 KEN Kari Gp NI 17 89.36 2.13 1.06
## 19 KEN Koibos Gp NI 19 0.00 71.59 15.91
## 20 KEN Meru nat. parc Gp NI 20 22.99 70.11 29.89
## 23 KEN Ruma nat. parc Gp NI 23 26.25 17.50 10.00
## 31 URT MaSAFng-URTga Gp NI 31 66.20 73.24 33.80
## 38 KEN Emsos Gp NI 38 0.00 93.33 15.00
## 42 UGA Budaka Gp NI 42 94.55 7.27 1.82
## 60 ZIM Makuti Gp NI 60 15.79 86.84 7.89
## 79 UGA Omugo Gp NI 79 100.00 0.00 0.00
## 81 ZIM Mushumb Gp NI 81 3.85 19.23 3.85
## 85 UGA Lira Gp NI 85 0.00 16.00 8.00
## 90 KEN Kiria Gp NI 90 0.00 80.00 55.00
## 101 KEN Mwea nat. parc Gp NI 101 0.00 13.33 13.33
## 118 UGA Moyo Gp NI 118 87.50 12.50 0.00
## 139 ZIM Rukomeshi Gp NI 139 0.00 0.00 0.00
## 140 ZIM Gokwe Gp NI 140 0.00 0.00 0.00
## Prev_Tv Prev_Tz Prev_Tsg Prev_TcTv Prev_TcTz Prev_TcTsg Prev_TvTz
## 1 0.65 1.09 10.46 0.00 0.00 0.65 0.00
## 5 15.38 0.51 26.15 6.67 1.54 15.90 1.03
## 6 0.51 1.03 7.69 1.03 0.51 0.51 0.00
## 8 0.68 0.68 34.93 0.00 0.00 7.53 0.00
## 9 2.74 0.68 8.22 0.00 0.00 0.68 0.00
## 17 0.00 1.06 0.00 0.00 0.00 0.00 0.00
## 19 9.09 0.00 28.41 0.00 0.00 15.91 0.00
## 20 4.60 0.00 25.29 0.00 0.00 10.34 0.00
## 23 2.50 2.50 0.00 1.25 0.00 0.00 0.00
## 31 0.00 0.00 14.08 0.00 1.41 21.13 1.41
## 38 8.33 0.00 58.33 1.67 0.00 10.00 0.00
## 42 0.00 0.00 5.45 0.00 0.00 0.00 0.00
## 60 0.00 0.00 73.68 0.00 0.00 5.26 0.00
## 79 0.00 0.00 0.00 0.00 0.00 0.00 0.00
## 81 0.00 7.69 3.85 0.00 0.00 3.85 0.00
## 85 0.00 4.00 4.00 0.00 0.00 0.00 0.00
## 90 5.00 0.00 10.00 10.00 0.00 0.00 0.00
## 101 0.00 0.00 0.00 0.00 0.00 0.00 0.00
## 118 0.00 0.00 12.50 0.00 0.00 0.00 0.00
## 139 0.00 0.00 0.00 0.00 0.00 0.00 0.00
## 140 0.00 0.00 0.00 0.00 0.00 0.00 0.00
## Prev_TvTsg Prev_TzTsg Prev_TcTvTz
## 1 0.44 0.65 0
## 5 0.00 0.00 0
## 6 0.00 1.03 0
## 8 0.00 0.00 0
## 9 0.68 0.68 0
## 17 0.00 0.00 0
## 19 0.00 2.27 0
## 20 0.00 0.00 0
## 23 1.25 0.00 0
## 31 0.00 1.41 0
## 38 0.00 0.00 0
## 42 0.00 0.00 0
## 60 0.00 0.00 0
## 79 0.00 0.00 0
## 81 0.00 0.00 0
## 85 0.00 0.00 0
## 90 0.00 0.00 0
## 101 0.00 0.00 0
## 118 0.00 0.00 0
## 139 0.00 0.00 0
## 140 0.00 0.00 0

model1<-glm((Prev_Tspp) ~ Country,data=data_gp, family=gaussian())
summary(model1)

##
## Call:
## glm(formula = (Prev_Tspp) ~ Country, family = gaussian(), data = data_gp)
##
## Deviance Residuals:
## Min 1Q Median 3Q Max
## -48.56 -26.52 0.00 20.90 60.32
##
## Coefficients:
## Estimate Std. Error t value Pr(>|t|)
## (Intercept) 26.518 17.046 1.556 0.141
## CountryZAM 18.692 38.117 0.490 0.631
## CountryUGA -17.575 24.107 -0.729 0.477
## CountryURT 20.032 29.525 0.678 0.508
## CountryKEN 24.171 20.487 1.180 0.256
## CountryETH -6.478 38.117 -0.170 0.867
##
## (Dispersion parameter for gaussian family taken to be 1162.303)
##
## Null deviance: 23227 on 20 degrees of freedom
## Residual deviance: 17435 on 15 degrees of freedom
## AIC: 214.75
##
## Number of Fisher Scoring iterations: 2

Anova(model1)

## Analysis of Deviance Table (Type II tests)
##
## Response: (Prev_Tspp)
## LR Chisq Df Pr(>Chisq)
## Country 4.9833 5 0.4179

model2<-glm((Prev_Sod) ~ Country,data=data_gp, family=gaussian())
summary(model2)

##
## Call:
## glm(formula = (Prev_Sod) ~ Country, family = gaussian(), data = data_gp)
##
## Deviance Residuals:
## Min 1Q Median 3Q Max
## -70.51 -17.39 0.00 10.88 68.89
##
## Coefficients:
## Estimate Std. Error t value Pr(>|t|)
## (Intercept) 4.91 15.39 0.319 0.75406
## CountryZAM -2.86 34.41 -0.083 0.93485
## CountryUGA 65.60 21.76 3.015 0.00871 **
## CountryURT 67.58 26.65 2.535 0.02285 *
## CountryKEN 15.56 18.49 0.841 0.41332
## CountryETH 15.57 34.41 0.453 0.65737
## ---
## Signif. codes: 0 '***' 0.001 '**' 0.01 '*' 0.05 '.' 0.1 ' ' 1
##
## (Dispersion parameter for gaussian family taken to be 947.1115)
##
## Null deviance: 28563 on 20 degrees of freedom
## Residual deviance: 14207 on 15 degrees of freedom
## AIC: 210.45
##
## Number of Fisher Scoring iterations: 2

Anova(model2)

## Analysis of Deviance Table (Type II tests)
##
## Response: (Prev_Sod)
## LR Chisq Df Pr(>Chisq)
## Country 15.158 5 0.00971 **
## ---
## Signif. codes: 0 '***' 0.001 '**' 0.01 '*' 0.05 '.' 0.1 ' ' 1

data_gp$Country <- relevel(data_gp$Country, ref= "KEN")
model2<-glm((Prev_Sod) ~ Country,data=data_gp, family=gaussian())
summary(model2)

##
## Call:
## glm(formula = (Prev_Sod) ~ Country, family = gaussian(), data = data_gp)
##
## Deviance Residuals:
## Min 1Q Median 3Q Max
## -70.51 -17.39 0.00 10.88 68.89
##
## Coefficients:
## Estimate Std. Error t value Pr(>|t|)
## (Intercept) 20.471111 10.258392 1.996 0.0645 .
## CountryZIM -15.561111 18.493580 -0.841 0.4133
## CountryZAM -18.421111 32.439885 -0.568 0.5785
## CountryUGA 50.041389 18.493580 2.706 0.0163 *
## CountryURT 52.013889 24.058063 2.162 0.0472 *
## CountryETH 0.008889 32.439885 0.000 0.9998
## ---
## Signif. codes: 0 '***' 0.001 '**' 0.01 '*' 0.05 '.' 0.1 ' ' 1
##
## (Dispersion parameter for gaussian family taken to be 947.1115)
##
## Null deviance: 28563 on 20 degrees of freedom
## Residual deviance: 14207 on 15 degrees of freedom
## AIC: 210.45
##
## Number of Fisher Scoring iterations: 2

Anova(model2)

## Analysis of Deviance Table (Type II tests)
##
## Response: (Prev_Sod)
## LR Chisq Df Pr(>Chisq)
## Country 15.158 5 0.00971 **
## ---
## Signif. codes: 0 '***' 0.001 '**' 0.01 '*' 0.05 '.' 0.1 ' ' 1

data_gp$Country <- relevel(data_gp$Country, ref= "URT")
model2<-glm((Prev_Sod) ~ Country,data=data_gp, family=gaussian())
summary(model2)

##
## Call:
## glm(formula = (Prev_Sod) ~ Country, family = gaussian(), data = data_gp)
##
## Deviance Residuals:
## Min 1Q Median 3Q Max
## -70.51 -17.39 0.00 10.88 68.89
##
## Coefficients:
## Estimate Std. Error t value Pr(>|t|)
## (Intercept) 72.485 21.761 3.331 0.00456 **
## CountryKEN -52.014 24.058 -2.162 0.04719 *
## CountryZIM -67.575 26.652 -2.535 0.02285 *
## CountryZAM -70.435 37.692 -1.869 0.08132 .
## CountryUGA -1.972 26.652 -0.074 0.94198
## CountryETH -52.005 37.692 -1.380 0.18789
## ---
## Signif. codes: 0 '***' 0.001 '**' 0.01 '*' 0.05 '.' 0.1 ' ' 1
##
## (Dispersion parameter for gaussian family taken to be 947.1115)
##
## Null deviance: 28563 on 20 degrees of freedom
## Residual deviance: 14207 on 15 degrees of freedom
## AIC: 210.45
##
## Number of Fisher Scoring iterations: 2

Anova(model2)

## Analysis of Deviance Table (Type II tests)
##
## Response: (Prev_Sod)
## LR Chisq Df Pr(>Chisq)
## Country 15.158 5 0.00971 **
## ---
## Signif. codes: 0 '***' 0.001 '**' 0.01 '*' 0.05 '.' 0.1 ' ' 1

data_gp$Country <- relevel(data_gp$Country, ref= "UGA")
model2<-glm((Prev_Sod) ~ Country,data=data_gp, family=gaussian())
summary(model2)

##
## Call:
## glm(formula = (Prev_Sod) ~ Country, family = gaussian(), data = data_gp)
##
## Deviance Residuals:
## Min 1Q Median 3Q Max
## -70.51 -17.39 0.00 10.88 68.89
##
## Coefficients:
## Estimate Std. Error t value Pr(>|t|)
## (Intercept) 70.513 15.388 4.582 0.000359 ***
## CountryURT 1.972 26.652 0.074 0.941981
## CountryKEN -50.041 18.494 -2.706 0.016266 *
## CountryZIM -65.602 21.761 -3.015 0.008709 **
## CountryZAM -68.463 34.408 -1.990 0.065170 .
## CountryETH -50.032 34.408 -1.454 0.166520
## ---
## Signif. codes: 0 '***' 0.001 '**' 0.01 '*' 0.05 '.' 0.1 ' ' 1
##
## (Dispersion parameter for gaussian family taken to be 947.1115)
##
## Null deviance: 28563 on 20 degrees of freedom
## Residual deviance: 14207 on 15 degrees of freedom
## AIC: 210.45
##
## Number of Fisher Scoring iterations: 2

Anova(model2)

## Analysis of Deviance Table (Type II tests)
##
## Response: (Prev_Sod)
## LR Chisq Df Pr(>Chisq)
## Country 15.158 5 0.00971 **
## ---
## Signif. codes: 0 '***' 0.001 '**' 0.01 '*' 0.05 '.' 0.1 ' ' 1

data_gp$Country <- relevel(data_gp$Country, ref= "ZAM")
model2<-glm((Prev_Sod) ~ Country,data=data_gp, family=gaussian())
summary(model2)

##
## Call:
## glm(formula = (Prev_Sod) ~ Country, family = gaussian(), data = data_gp)
##
## Deviance Residuals:
## Min 1Q Median 3Q Max
## -70.51 -17.39 0.00 10.88 68.89
##
## Coefficients:
## Estimate Std. Error t value Pr(>|t|)
## (Intercept) 2.05 30.77 0.067 0.9478
## CountryUGA 68.46 34.41 1.990 0.0652 .
## CountryURT 70.44 37.69 1.869 0.0813 .
## CountryKEN 18.42 32.44 0.568 0.5785
## CountryZIM 2.86 34.41 0.083 0.9349
## CountryETH 18.43 43.52 0.423 0.6780
## ---
## Signif. codes: 0 '***' 0.001 '**' 0.01 '*' 0.05 '.' 0.1 ' ' 1
##
## (Dispersion parameter for gaussian family taken to be 947.1115)
##
## Null deviance: 28563 on 20 degrees of freedom
## Residual deviance: 14207 on 15 degrees of freedom
## AIC: 210.45
##
## Number of Fisher Scoring iterations: 2

Anova(model2)

## Analysis of Deviance Table (Type II tests)
##
## Response: (Prev_Sod)
## LR Chisq Df Pr(>Chisq)
## Country 15.158 5 0.00971 **
## ---
## Signif. codes: 0 '***' 0.001 '**' 0.01 '*' 0.05 '.' 0.1 ' ' 1

data_gp$Country <- relevel(data_gp$Country, ref= "ZIM")
model2<-glm((Prev_Sod) ~ Country,data=data_gp, family=gaussian())
summary(model2)

##
## Call:
## glm(formula = (Prev_Sod) ~ Country, family = gaussian(), data = data_gp)
##
## Deviance Residuals:
## Min 1Q Median 3Q Max
## -70.51 -17.39 0.00 10.88 68.89
##
## Coefficients:
## Estimate Std. Error t value Pr(>|t|)
## (Intercept) 4.91 15.39 0.319 0.75406
## CountryZAM -2.86 34.41 -0.083 0.93485
## CountryUGA 65.60 21.76 3.015 0.00871 **
## CountryURT 67.58 26.65 2.535 0.02285 *
## CountryKEN 15.56 18.49 0.841 0.41332
## CountryETH 15.57 34.41 0.453 0.65737
## ---
## Signif. codes: 0 '***' 0.001 '**' 0.01 '*' 0.05 '.' 0.1 ' ' 1
##
## (Dispersion parameter for gaussian family taken to be 947.1115)
##
## Null deviance: 28563 on 20 degrees of freedom
## Residual deviance: 14207 on 15 degrees of freedom
## AIC: 210.45
##
## Number of Fisher Scoring iterations: 2

Anova(model2)

## Analysis of Deviance Table (Type II tests)
##
## Response: (Prev_Sod)
## LR Chisq Df Pr(>Chisq)
## Country 15.158 5 0.00971 **
## ---
## Signif. codes: 0 '***' 0.001 '**' 0.01 '*' 0.05 '.' 0.1 ' ' 1

#------------------------------------------------------------------
#Gpg

data_gpg <- subset(data, Species=="Gpg")
data_gpg

## Country Localisation Species Sex Sample Prev_Sod Prev_Tspp Prev_Tc
## 4 SEN Pout Gpg NI 4 0.00 7.04 1.51
## 12 BKF Folonzo Gpg F 12 0.00 7.55 0.94
## 13 BKF Folonzo Gpg M 13 1.89 16.04 0.00
## 15 GUI Kangoliya Gpg F 15 95.74 0.00 0.00
## 18 SEN Kayar Gpg NI 18 0.00 1.14 0.00
## 25 BKF Moussodougou Gpg F 25 0.00 44.87 0.00
## 30 BKF Comoe Gpg F 30 0.00 2.82 1.41
## 32 BKF Kartasso Gpg F 32 0.00 0.00 0.00
## 33 BKF Kartasso Gpg M 33 0.00 0.00 0.00
## 34 SEN DiackSAFo Peulh Gpg NI 34 0.00 7.69 0.00
## 35 BKF Moussodougou Gpg M 35 0.00 21.88 0.00
## 40 MLI Bani Gpg F 40 0.00 1.72 0.00
## 45 BKF Kampty Gpg F 45 0.00 90.57 1.89
## 47 BKF Comoe Gpg M 47 0.00 1.92 1.92
## 48 MLI SEN Gpg M 48 0.00 7.69 1.92
## 54 BKF Bama Gpg F 54 0.00 0.00 0.00
## 56 SEN Tambacounda Gpg F 56 0.00 41.46 0.00
## 57 SEN Tambacounda Gpg M 57 0.00 71.79 0.00
## 58 SEN SebikoURTe Gpg NI 58 0.00 5.13 0.00
## 62 MLI SEN Gpg F 62 0.00 0.00 0.00
## 64 BKF Bama Gpg M 64 0.00 0.00 0.00
## 65 MLI Banco Gpg F 65 0.00 20.59 0.00
## 66 BKF Dedougou Gpg F 66 0.00 52.94 0.00
## 67 MLI Sikasso Gpg F 67 0.00 6.06 0.00
## 71 GUI Kangoliya Gpg M 71 0.00 0.00 0.00
## 77 GUI Mini Gpg F 77 0.00 3.45 0.00
## 78 SEN Hann Gpg NI 78 0.00 0.00 0.00
## 80 BKF Kampty Gpg M 80 0.00 80.77 0.00
## 82 GUI Kifala Gpg M 82 0.00 0.00 0.00
## 83 MLI Sikasso Gpg M 83 0.00 0.00 0.00
## 84 MLI SS Gpg F 84 0.00 4.00 0.00
## 86 BKF Kenedougou Gpg F 86 0.00 0.00 0.00
## 87 MLI SS Gpg M 87 0.00 0.00 0.00
## 88 BKF Dedougou Gpg M 88 0.00 69.57 4.35
## 89 GUI Bafing Gpg F 89 0.00 5.00 0.00
## 91 BKF Kampty Gpg NI 91 0.00 84.21 0.00
## 92 GUI Tinkisso Gpg M 92 0.00 5.56 0.00
## 94 BKF Kenedougou Gpg M 94 0.00 0.00 0.00
## 96 GUI Dekonkore Gpg F 96 0.00 6.25 0.00
## 97 GUI Mini Gpg M 97 0.00 0.00 0.00
## 98 SEN Fleuve Gambie Gpg M 98 0.00 43.75 0.00
## 102 GUI Bafing Gpg M 102 0.00 0.00 0.00
## 103 GUI Tinkisso Gpg F 103 0.00 7.69 0.00
## 104 BKF Bouroum bouroum Gpg F 104 0.00 92.31 0.00
## 106 GUI Karifale Gpg M 106 0.00 8.33 0.00
## 107 GUI Lemonako Gpg F 107 0.00 0.00 0.00
## 108 BKF KouriGUIon Gpg F 108 0.00 50.00 0.00
## 109 MLI Bani Gpg M 109 0.00 0.00 0.00
## 110 MLI Sybi Gpg F 110 0.00 0.00 0.00
## 111 MLI Sybi Gpg M 111 0.00 0.00 0.00
## 113 SEN Fleueve G Gpg F 113 0.00 11.11 0.00
## 114 BKF KouriGUIon Gpg NI 114 0.00 22.22 0.00
## 117 GUI Lemonako Gpg M 117 0.00 0.00 0.00
## 119 SEN Diaguiri Gpg F 119 0.00 0.00 0.00
## 120 MLI Banco Gpg M 120 0.00 28.57 0.00
## 121 MLI Baoule Gpg F 121 0.00 42.86 0.00
## 122 MLI Baoule Gpg M 122 0.00 42.86 0.00
## 123 MLI Bougouni Gpg M 123 0.00 0.00 0.00
## 124 BKF Lorepeni Gpg F 124 0.00 71.43 0.00
## 127 SEN MousSAFlla Gpg M 127 0.00 0.00 0.00
## 128 MLI Baguineda Gpg F 128 0.00 16.67 0.00
## 129 MLI Bougouni Gpg F 129 0.00 0.00 0.00
## 130 MLI Kita Gpg M 130 0.00 16.67 0.00
## 136 GUI Kifala Gpg F 136 0.00 0.00 0.00
## 137 BKF Bouroum bouroum Gpg M 137 0.00 80.00 0.00
## 138 SEN Fleuve Gambie Gpg F 138 0.00 25.00 0.00
## 143 SEN Niokolo Gpg M 143 0.00 0.00 0.00
## 145 BKF Lorepeni Gpg M 145 0.00 100.00 0.00
## 146 BKF KouriGUIon Gpg M 146 0.00 66.67 0.00
## 147 BKF Ouarkoye Gpg M 147 0.00 100.00 0.00
## 150 SEN Diaguiri Gpg M 150 0.00 50.00 0.00
## 151 BKF Ouarkoye Gpg F 151 0.00 100.00 50.00
## 156 GUI Karifale Gpg F 156 0.00 0.00 0.00
## 157 SEN Mako Gpg M 157 0.00 100.00 0.00
## Prev_Tv Prev_Tz Prev_Tsg Prev_TcTv Prev_TcTz Prev_TcTsg Prev_TvTz
## 4 2.01 0.50 3.02 0 0.00 0.00 0.00
## 12 2.83 1.89 0.00 0 0.00 0.00 1.89
## 13 5.66 5.66 0.00 0 1.89 0.00 2.83
## 15 0.00 0.00 0.00 0 0.00 0.00 0.00
## 18 0.00 0.00 1.14 0 0.00 0.00 0.00
## 25 20.51 12.82 0.00 0 0.00 0.00 11.54
## 30 1.41 0.00 0.00 0 0.00 0.00 0.00
## 32 0.00 0.00 0.00 0 0.00 0.00 0.00
## 33 0.00 0.00 0.00 0 0.00 0.00 0.00
## 34 1.54 1.54 4.62 0 0.00 0.00 0.00
## 35 6.25 15.63 0.00 0 0.00 0.00 0.00
## 40 1.72 0.00 0.00 0 0.00 0.00 0.00
## 45 62.26 1.89 11.32 0 0.00 0.00 0.00
## 47 0.00 0.00 0.00 0 0.00 0.00 0.00
## 48 1.92 3.85 0.00 0 0.00 0.00 0.00
## 54 0.00 0.00 0.00 0 0.00 0.00 0.00
## 56 26.83 7.32 0.00 0 0.00 0.00 7.32
## 57 53.85 2.56 0.00 0 0.00 0.00 15.38
## 58 5.13 0.00 0.00 0 0.00 0.00 0.00
## 62 0.00 0.00 0.00 0 0.00 0.00 0.00
## 64 0.00 0.00 0.00 0 0.00 0.00 0.00
## 65 20.59 0.00 0.00 0 0.00 0.00 0.00
## 66 26.47 0.00 23.53 0 0.00 0.00 0.00
## 67 0.00 6.06 0.00 0 0.00 0.00 0.00
## 71 0.00 0.00 0.00 0 0.00 0.00 0.00
## 77 3.45 0.00 0.00 0 0.00 0.00 0.00
## 78 0.00 0.00 0.00 0 0.00 0.00 0.00
## 80 65.38 0.00 7.69 0 0.00 0.00 0.00
## 82 0.00 0.00 0.00 0 0.00 0.00 0.00
## 83 0.00 0.00 0.00 0 0.00 0.00 0.00
## 84 4.00 0.00 0.00 0 0.00 0.00 0.00
## 86 0.00 0.00 0.00 0 0.00 0.00 0.00
## 87 0.00 0.00 0.00 0 0.00 0.00 0.00
## 88 30.43 13.04 4.35 0 0.00 4.35 0.00
## 89 5.00 0.00 0.00 0 0.00 0.00 0.00
## 91 26.32 5.26 21.05 0 0.00 0.00 0.00
## 92 5.56 0.00 0.00 0 0.00 0.00 0.00
## 94 0.00 0.00 0.00 0 0.00 0.00 0.00
## 96 6.25 0.00 0.00 0 0.00 0.00 0.00
## 97 0.00 0.00 0.00 0 0.00 0.00 0.00
## 98 43.75 0.00 0.00 0 0.00 0.00 0.00
## 102 0.00 0.00 0.00 0 0.00 0.00 0.00
## 103 7.69 0.00 0.00 0 0.00 0.00 0.00
## 104 53.85 0.00 23.08 0 0.00 0.00 0.00
## 106 8.33 0.00 0.00 0 0.00 0.00 0.00
## 107 8.33 0.00 0.00 0 0.00 0.00 0.00
## 108 0.00 0.00 33.33 0 0.00 0.00 0.00
## 109 0.00 0.00 0.00 0 0.00 0.00 0.00
## 110 0.00 0.00 0.00 0 0.00 0.00 0.00
## 111 0.00 0.00 0.00 0 0.00 0.00 0.00
## 113 11.11 0.00 0.00 0 0.00 0.00 0.00
## 114 0.00 0.00 11.11 0 0.00 0.00 0.00
## 117 0.00 0.00 0.00 0 0.00 0.00 0.00
## 119 0.00 0.00 0.00 0 0.00 0.00 0.00
## 120 28.57 0.00 0.00 0 0.00 0.00 0.00
## 121 42.86 0.00 0.00 0 0.00 0.00 0.00
## 122 42.86 0.00 0.00 0 0.00 0.00 0.00
## 123 0.00 0.00 0.00 0 0.00 0.00 0.00
## 124 14.29 0.00 28.57 0 0.00 0.00 0.00
## 127 0.00 0.00 0.00 0 0.00 0.00 0.00
## 128 16.67 0.00 0.00 0 0.00 0.00 0.00
## 129 0.00 0.00 0.00 0 0.00 0.00 0.00
## 130 16.67 0.00 0.00 0 0.00 0.00 0.00
## 136 0.00 0.00 0.00 0 0.00 0.00 0.00
## 137 40.00 0.00 20.00 0 0.00 0.00 0.00
## 138 25.00 0.00 0.00 0 0.00 0.00 0.00
## 143 66.67 0.00 0.00 0 0.00 0.00 0.00
## 145 0.00 0.00 0.00 0 0.00 0.00 0.00
## 146 33.33 0.00 0.00 0 0.00 0.00 0.00
## 147 33.33 0.00 0.00 0 0.00 0.00 0.00
## 150 0.00 50.00 0.00 0 0.00 0.00 0.00
## 151 50.00 0.00 0.00 0 0.00 0.00 0.00
## 156 0.00 0.00 0.00 0 0.00 0.00 0.00
## 157 100.00 0.00 0.00 0 0.00 0.00 0.00
## Prev_TvTsg Prev_TzTsg Prev_TcTvTz
## 4 0.00 0.00 0
## 12 0.00 0.00 0
## 13 0.00 0.00 0
## 15 0.00 0.00 0
## 18 0.00 0.00 0
## 25 0.00 0.00 0
## 30 0.00 0.00 0
## 32 0.00 0.00 0
## 33 0.00 0.00 0
## 34 0.00 0.00 0
## 35 0.00 0.00 0
## 40 0.00 0.00 0
## 45 9.43 3.77 0
## 47 0.00 0.00 0
## 48 0.00 0.00 0
## 54 0.00 0.00 0
## 56 0.00 0.00 0
## 57 0.00 0.00 0
## 58 0.00 0.00 0
## 62 0.00 0.00 0
## 64 0.00 0.00 0
## 65 0.00 0.00 0
## 66 2.94 0.00 0
## 67 0.00 0.00 0
## 71 0.00 0.00 0
## 77 0.00 0.00 0
## 78 0.00 0.00 0
## 80 7.69 0.00 0
## 82 0.00 0.00 0
## 83 0.00 0.00 0
## 84 0.00 0.00 0
## 86 0.00 0.00 0
## 87 0.00 0.00 0
## 88 4.35 8.70 0
## 89 0.00 0.00 0
## 91 31.58 0.00 0
## 92 0.00 0.00 0
## 94 0.00 0.00 0
## 96 0.00 0.00 0
## 97 0.00 0.00 0
## 98 0.00 0.00 0
## 102 0.00 0.00 0
## 103 0.00 0.00 0
## 104 15.38 0.00 0
## 106 0.00 0.00 0
## 107 0.00 0.00 0
## 108 16.67 0.00 0
## 109 0.00 0.00 0
## 110 0.00 0.00 0
## 111 0.00 0.00 0
## 113 0.00 0.00 0
## 114 11.11 0.00 0
## 117 0.00 0.00 0
## 119 0.00 0.00 0
## 120 0.00 0.00 0
## 121 0.00 0.00 0
## 122 0.00 0.00 0
## 123 0.00 0.00 0
## 124 28.57 0.00 0
## 127 0.00 0.00 0
## 128 0.00 0.00 0
## 129 0.00 0.00 0
## 130 0.00 0.00 0
## 136 0.00 0.00 0
## 137 20.00 0.00 0
## 138 0.00 0.00 0
## 143 0.00 0.00 0
## 145 66.67 33.33 0
## 146 33.33 0.00 0
## 147 33.33 33.33 0
## 150 0.00 0.00 0
## 151 0.00 0.00 0
## 156 0.00 0.00 0
## 157 0.00 0.00 0

model1<-glm((Prev_Tspp) ~ Country,data=data_gpg, family=gaussian())
summary(model1)

##
## Call:
## glm(formula = (Prev_Tspp) ~ Country, family = gaussian(), data = data_gpg)
##
## Deviance Residuals:
## Min 1Q Median 3Q Max
## -44.453 -17.072 -2.419 15.430 75.726
##
## Coefficients:
## Estimate Std. Error t value Pr(>|t|)
## (Intercept) 24.274 7.268 3.340 0.00135 **
## CountryMLI -13.847 9.842 -1.407 0.16386
## CountryGUI -21.855 10.279 -2.126 0.03702 *
## CountryBKF 20.179 9.127 2.211 0.03032 *
## ---
## Signif. codes: 0 '***' 0.001 '**' 0.01 '*' 0.05 '.' 0.1 ' ' 1
##
## (Dispersion parameter for gaussian family taken to be 792.4573)
##
## Null deviance: 76638 on 73 degrees of freedom
## Residual deviance: 55472 on 70 degrees of freedom
## AIC: 709.85
##
## Number of Fisher Scoring iterations: 2

Anova(model1)

## Analysis of Deviance Table (Type II tests)
##
## Response: (Prev_Tspp)
## LR Chisq Df Pr(>Chisq)
## Country 26.709 3 6.776e-06 ***
## ---
## Signif. codes: 0 '***' 0.001 '**' 0.01 '*' 0.05 '.' 0.1 ' ' 1

model2<-glm((Prev_Sod) ~ Country,data=data_gpg, family=gaussian())
summary(model2)

##
## Call:
## glm(formula = (Prev_Sod) ~ Country, family = gaussian(), data = data_gpg)
##
## Deviance Residuals:
## Min 1Q Median 3Q Max
## -6.383 -0.073 -0.073 0.000 89.357
##
## Coefficients:
## Estimate Std. Error t value Pr(>|t|)
## (Intercept) 1.424e-14 2.855e+00 0.000 1.000
## CountryMLI -1.781e-14 3.866e+00 0.000 1.000
## CountryGUI 6.383e+00 4.038e+00 1.581 0.118
## CountryBKF 7.269e-02 3.585e+00 0.020 0.984
##
## (Dispersion parameter for gaussian family taken to be 122.2644)
##
## Null deviance: 9040.9 on 73 degrees of freedom
## Residual deviance: 8558.5 on 70 degrees of freedom
## AIC: 571.55
##
## Number of Fisher Scoring iterations: 2

Anova(model2)

## Analysis of Deviance Table (Type II tests)
##
## Response: (Prev_Sod)
## LR Chisq Df Pr(>Chisq)
## Country 3.9456 3 0.2674

data_gpg$Country <- relevel(data_gpg$Country, ref= "GUI")
model1<-glm((Prev_Tspp) ~ Country,data=data_gpg, family=gaussian())
summary(model1)

##
## Call:
## glm(formula = (Prev_Tspp) ~ Country, family = gaussian(), data = data_gpg)
##
## Deviance Residuals:
## Min 1Q Median 3Q Max
## -44.453 -17.072 -2.419 15.430 75.726
##
## Coefficients:
## Estimate Std. Error t value Pr(>|t|)
## (Intercept) 2.419 7.268 0.333 0.740
## CountrySEN 21.855 10.279 2.126 0.037 *
## CountryMLI 8.009 9.842 0.814 0.419
## CountryBKF 42.034 9.127 4.605 1.79e-05 ***
## ---
## Signif. codes: 0 '***' 0.001 '**' 0.01 '*' 0.05 '.' 0.1 ' ' 1
##
## (Dispersion parameter for gaussian family taken to be 792.4573)
##
## Null deviance: 76638 on 73 degrees of freedom
## Residual deviance: 55472 on 70 degrees of freedom
## AIC: 709.85
##
## Number of Fisher Scoring iterations: 2

Anova(model1)

## Analysis of Deviance Table (Type II tests)
##
## Response: (Prev_Tspp)
## LR Chisq Df Pr(>Chisq)
## Country 26.709 3 6.776e-06 ***
## ---
## Signif. codes: 0 '***' 0.001 '**' 0.01 '*' 0.05 '.' 0.1 ' ' 1

data_gpg$Country <- relevel(data_gpg$Country, ref= "MLI")
model1<-glm((Prev_Tspp) ~ Country,data=data_gpg, family=gaussian())
summary(model1)

##
## Call:
## glm(formula = (Prev_Tspp) ~ Country, family = gaussian(), data = data_gpg)
##
## Deviance Residuals:
## Min 1Q Median 3Q Max
## -44.453 -17.072 -2.419 15.430 75.726
##
## Coefficients:
## Estimate Std. Error t value Pr(>|t|)
## (Intercept) 10.427 6.635 1.572 0.120574
## CountryGUI -8.009 9.842 -0.814 0.418547
## CountrySEN 13.847 9.842 1.407 0.163860
## CountryBKF 34.025 8.632 3.942 0.000189 ***
## ---
## Signif. codes: 0 '***' 0.001 '**' 0.01 '*' 0.05 '.' 0.1 ' ' 1
##
## (Dispersion parameter for gaussian family taken to be 792.4573)
##
## Null deviance: 76638 on 73 degrees of freedom
## Residual deviance: 55472 on 70 degrees of freedom
## AIC: 709.85
##
## Number of Fisher Scoring iterations: 2

Anova(model1)

## Analysis of Deviance Table (Type II tests)
##
## Response: (Prev_Tspp)
## LR Chisq Df Pr(>Chisq)
## Country 26.709 3 6.776e-06 ***
## ---
## Signif. codes: 0 '***' 0.001 '**' 0.01 '*' 0.05 '.' 0.1 ' ' 1

data_gpg$Country <- relevel(data_gpg$Country, ref= "SEN")
model1<-glm((Prev_Tspp) ~ Country,data=data_gpg, family=gaussian())
summary(model1)

##
## Call:
## glm(formula = (Prev_Tspp) ~ Country, family = gaussian(), data = data_gpg)
##
## Deviance Residuals:
## Min 1Q Median 3Q Max
## -44.453 -17.072 -2.419 15.430 75.726
##
## Coefficients:
## Estimate Std. Error t value Pr(>|t|)
## (Intercept) 24.274 7.268 3.340 0.00135 **
## CountryMLI -13.847 9.842 -1.407 0.16386
## CountryGUI -21.855 10.279 -2.126 0.03702 *
## CountryBKF 20.179 9.127 2.211 0.03032 *
## ---
## Signif. codes: 0 '***' 0.001 '**' 0.01 '*' 0.05 '.' 0.1 ' ' 1
##
## (Dispersion parameter for gaussian family taken to be 792.4573)
##
## Null deviance: 76638 on 73 degrees of freedom
## Residual deviance: 55472 on 70 degrees of freedom
## AIC: 709.85
##
## Number of Fisher Scoring iterations: 2

Anova(model1)

## Analysis of Deviance Table (Type II tests)
##
## Response: (Prev_Tspp)
## LR Chisq Df Pr(>Chisq)
## Country 26.709 3 6.776e-06 ***
## ---
## Signif. codes: 0 '***' 0.001 '**' 0.01 '*' 0.05 '.' 0.1 ' ' 1

#-------------------------------------------------------------
#Gt

data_gt <- subset(data, Species=="Gt")
data_gt

## Country Localisation Species Sex Sample Prev_Sod Prev_Tspp Prev_Tc Prev_Tv
## 2 BKF Comoe Gt F 2 0 16.54 1.10 13.24
## 3 BKF Comoe Gt M 3 0 15.00 3.64 11.36
## 11 BKF Folonzo Gt F 11 0 18.87 1.89 12.26
## 14 BKF Folonzo Gt M 14 0 32.63 2.11 27.37
## 24 GHA Walewale Gt M 24 0 53.85 3.85 12.82
## 43 GHA Walewale Gt F 43 0 66.04 1.89 9.43
## 44 BKF Arly Gt F 44 0 1.89 0.00 0.00
## 55 BKF Arly Gt M 55 0 0.00 0.00 0.00
## 69 GHA MorURTi Gt M 69 0 50.00 0.00 15.63
## 70 BKF Sissili Gt M 70 0 25.00 12.50 12.50
## 95 GHA Bougouhiya Gt F 95 0 18.75 0.00 6.25
## 99 BKF Sissili Gt F 99 0 13.33 6.67 6.67
## 112 GHA MorURTi Gt F 112 0 66.67 0.00 0.00
## 116 GHA Fumbissi Gt F 116 0 100.00 0.00 0.00
## 125 GHA Fumbissi Gt M 125 0 100.00 0.00 66.67
## 126 GHA Grogro Gt M 126 0 100.00 0.00 0.00
## 133 GHA Grogro Gt F 133 0 100.00 0.00 80.00
## 134 GHA Kumpole Gt F 134 0 100.00 0.00 40.00
## 135 GHA Sissili Bidge Gt F 135 0 100.00 0.00 20.00
## 142 GHA Bougouhiya Gt M 142 0 0.00 0.00 0.00
## 148 GHA Kumpole Gt M 148 0 100.00 0.00 50.00
## 149 GHA Psikp_ Gt M 149 0 100.00 0.00 0.00
## 152 GHA Kandiaga Gt M 152 0 100.00 0.00 0.00
## 153 GHA Sissili Bidge Gt M 153 0 100.00 0.00 0.00
## 154 GHA Nabogo Gt F 154 0 0.00 0.00 0.00
## 155 GHA Volta Blanche Gt F 155 0 0.00 0.00 0.00
## Prev_Tz Prev_Tsg Prev_TcTv Prev_TcTz Prev_TcTsg Prev_TvTz Prev_TvTsg
## 2 0.37 0 0.74 1.10 0 0.00 0
## 3 0.00 0 0.00 0.00 0 0.00 0
## 11 1.89 0 1.89 0.94 0 0.00 0
## 14 1.05 0 0.00 1.05 0 1.05 0
## 24 8.97 0 0.00 10.26 0 14.10 0
## 43 24.53 0 0.00 16.98 0 7.55 0
## 44 1.89 0 0.00 0.00 0 0.00 0
## 55 0.00 0 0.00 0.00 0 0.00 0
## 69 15.63 0 0.00 18.75 0 0.00 0
## 70 0.00 0 0.00 0.00 0 0.00 0
## 95 0.00 0 0.00 0.00 0 12.50 0
## 99 0.00 0 0.00 0.00 0 0.00 0
## 112 33.33 0 0.00 22.22 0 11.11 0
## 116 37.50 0 0.00 0.00 0 62.50 0
## 125 33.33 0 0.00 0.00 0 0.00 0
## 126 16.67 0 0.00 0.00 0 83.33 0
## 133 20.00 0 0.00 0.00 0 0.00 0
## 134 60.00 0 0.00 0.00 0 0.00 0
## 135 80.00 0 0.00 0.00 0 0.00 0
## 142 0.00 0 0.00 0.00 0 0.00 0
## 148 50.00 0 0.00 0.00 0 0.00 0
## 149 50.00 0 0.00 0.00 0 50.00 0
## 152 100.00 0 0.00 0.00 0 0.00 0
## 153 100.00 0 0.00 0.00 0 0.00 0
## 154 0.00 0 0.00 0.00 0 0.00 0
## 155 0.00 0 0.00 0.00 0 0.00 0
## Prev_TzTsg Prev_TcTvTz
## 2 0 0.00
## 3 0 0.00
## 11 0 0.00
## 14 0 0.00
## 24 0 1.28
## 43 0 5.66
## 44 0 0.00
## 55 0 0.00
## 69 0 0.00
## 70 0 0.00
## 95 0 0.00
## 99 0 0.00
## 112 0 0.00
## 116 0 0.00
## 125 0 0.00
## 126 0 0.00
## 133 0 0.00
## 134 0 0.00
## 135 0 0.00
## 142 0 0.00
## 148 0 0.00
## 149 0 0.00
## 152 0 0.00
## 153 0 0.00
## 154 0 0.00
## 155 0 0.00

model1<-glm((Prev_Tspp) ~ Country,data=data_gt, family=gaussian())
summary(model1)

##
## Call:
## glm(formula = (Prev_Tspp) ~ Country, family = gaussian(), data = data_gt)
##
## Deviance Residuals:
## Min 1Q Median 3Q Max
## -69.739 -14.935 2.297 30.261 30.261
##
## Coefficients:
## Estimate Std. Error t value Pr(>|t|)
## (Intercept) 69.739 8.028 8.687 7.12e-09 ***
## CountryBKF -54.332 14.472 -3.754 0.000978 ***
## ---
## Signif. codes: 0 '***' 0.001 '**' 0.01 '*' 0.05 '.' 0.1 ' ' 1
##
## (Dispersion parameter for gaussian family taken to be 1159.976)
##
## Null deviance: 44189 on 25 degrees of freedom
## Residual deviance: 27839 on 24 degrees of freedom
## AIC: 261.16
##
## Number of Fisher Scoring iterations: 2

Anova(model1)

## Analysis of Deviance Table (Type II tests)
##
## Response: (Prev_Tspp)
## LR Chisq Df Pr(>Chisq)
## Country 14.095 1 0.0001738 ***
## ---
## Signif. codes: 0 '***' 0.001 '**' 0.01 '*' 0.05 '.' 0.1 ' ' 1

model2<-glm((Prev_Sod) ~ Country,data=data_gt, family=gaussian())
summary(model2)

##
## Call:
## glm(formula = (Prev_Sod) ~ Country, family = gaussian(), data = data_gt)
##
## Deviance Residuals:
## Min 1Q Median 3Q Max
## 0 0 0 0 0
##
## Coefficients:
## Estimate Std. Error t value Pr(>|t|)
## (Intercept) 0 0 NA NA
## CountryBKF 0 0 NA NA
##
## (Dispersion parameter for gaussian family taken to be 0)
##
## Null deviance: 0 on 25 degrees of freedom
## Residual deviance: 0 on 24 degrees of freedom
## AIC: -Inf
##
## Number of Fisher Scoring iterations: 1

Anova(model2)

## Analysis of Deviance Table (Type II tests)
##
## Response: (Prev_Sod)
## LR Chisq Df Pr(>Chisq)
## Country 1

## Selecting the GLM model for trypanosome species and mixed infection

#TC selection model
model1<-glm((Prev_Tc) ~ Country,data=data, family=gaussian())
model2<-glm((Prev_Tc) ~ Species,data=data, family=gaussian())
model3<-glm((Prev_Tc) ~ Country*Species,data=data, family=gaussian())
model4<-glm((Prev_Tc) ~ Country+Species,data=data, family=gaussian())
model5<-glm((Prev_Tc) ~ Country*Species+Localisation,data=data, family=gaussian())
model6<-glm((Prev_Tc) ~ Country+Species+Localisation,data=data, family=gaussian())

#AICc(model1, model2, model3, model4)
model.set <- list(model1, model2, model3, model4, model5, model6)
model.names <- c("model1", "model2","model3", "model4", "model5", "model6")

aictab(model.set, modnames = model.names)

##
## Model selection based on AICc:
##
## K AICc Delta_AICc AICcWt Cum.Wt LL
## model2 11 1077.78 0.00 0.43 0.43 -526.97
## model4 23 1078.08 0.30 0.37 0.81 -511.86
## model3 27 1079.90 2.12 0.15 0.96 -507.04
## model1 16 1082.45 4.67 0.04 1.00 -523.27
## model6 100 1416.98 339.20 0.00 1.00 -424.85
## model5 103 1455.28 377.50 0.00 1.00 -418.64

summary(model1)

##
## Call:
## glm(formula = (Prev_Tc) ~ Country, family = gaussian(), data = data)
##
## Deviance Residuals:
## Min 1Q Median 3Q Max
## -13.594 -3.404 -0.107 0.000 46.596
##
## Coefficients:
## Estimate Std. Error t value Pr(>|t|)
## (Intercept) 4.764 2.304 2.068 0.04048 *
## CountryZAM -3.394 7.641 -0.444 0.65759
## CountrySAF 0.126 3.456 0.036 0.97097
## CountryUGA -2.162 3.990 -0.542 0.58881
## CountryURT 4.983 3.762 1.324 0.18751
## CountrySWA -4.764 7.641 -0.623 0.53398
## CountrySEN -4.663 2.974 -1.568 0.11915
## CountryZAI -4.764 7.641 -0.623 0.53398
## CountryMOZ -1.764 5.643 -0.313 0.75506
## CountryMLI -4.657 2.873 -1.621 0.10729
## CountryKEN 9.890 3.119 3.170 0.00187 **
## CountryETH 1.336 7.641 0.175 0.86145
## CountryGUI -4.764 2.974 -1.602 0.11145
## CountryGHA -4.445 2.873 -1.547 0.12411
## CountryBKF -1.360 2.558 -0.532 0.59568
## ---
## Signif. codes: 0 '***' 0.001 '**' 0.01 '*' 0.05 '.' 0.1 ' ' 1
##
## (Dispersion parameter for gaussian family taken to be 53.07752)
##
## Null deviance: 10017.9 on 155 degrees of freedom
## Residual deviance: 7483.9 on 141 degrees of freedom
## AIC: 1078.5
##
## Number of Fisher Scoring iterations: 2

Anova(model1)

## Analysis of Deviance Table (Type II tests)
##
## Response: (Prev_Tc)
## LR Chisq Df Pr(>Chisq)
## Country 47.741 14 1.451e-05 ***
## ---
## Signif. codes: 0 '***' 0.001 '**' 0.01 '*' 0.05 '.' 0.1 ' ' 1

#model2 is the best AICc=1084.04
Anova(model2)

## Analysis of Deviance Table (Type II tests)
##
## Response: (Prev_Tc)
## LR Chisq Df Pr(>Chisq)
## Species 40.364 9 6.527e-06 ***
## ---
## Signif. codes: 0 '***' 0.001 '**' 0.01 '*' 0.05 '.' 0.1 ' ' 1

#-------------------------------------------------------------------------
#Tv selection model
model1<-glm((Prev_Tv) ~ Country,data=data, family=gaussian())
model2<-glm((Prev_Tv) ~ Species,data=data, family=gaussian())
model3<-glm((Prev_Tv) ~ Country*Species,data=data, family=gaussian())
model4<-glm((Prev_Tv) ~ Country+Species,data=data, family=gaussian())
model5<-glm((Prev_Tv) ~ Country*Species+Localisation,data=data, family=gaussian())
model6<-glm((Prev_Tv) ~ Country+Species+Localisation,data=data, family=gaussian())

#AICc(model1, model2, model3, model4)
model.set <- list(model1, model2, model3, model4, model5, model6)
model.names <- c("model1", "model2","model3", "model4", "model5", "model6")

aictab(model.set, modnames = model.names)

##
## Model selection based on AICc:
##
## K AICc Delta_AICc AICcWt Cum.Wt LL
## model2 11 1352.64 0.00 0.51 0.51 -664.40
## model1 16 1352.73 0.09 0.49 1.00 -658.41
## model4 23 1368.94 16.30 0.00 1.00 -657.29
## model3 27 1380.29 27.66 0.00 1.00 -657.24
## model6 100 1652.45 299.82 0.00 1.00 -542.59
## model5 103 1703.17 350.54 0.00 1.00 -542.59

summary(model1)

##
## Call:
## glm(formula = (Prev_Tv) ~ Country, family = gaussian(), data = data)
##
## Deviance Residuals:
## Min 1Q Median 3Q Max
## -22.393 -9.770 -1.427 3.072 77.607
##
## Coefficients:
## Estimate Std. Error t value Pr(>|t|)
## (Intercept) 0.5560 5.4787 0.101 0.91931
## CountryZAM 0.1240 18.1708 0.007 0.99456
## CountrySAF 0.3465 8.2180 0.042 0.96643
## CountryUGA -0.1300 9.4894 -0.014 0.98909
## CountryURT 1.2173 8.9467 0.136 0.89196
## CountrySWA -0.5560 18.1708 -0.031 0.97563
## CountrySEN 21.8367 7.0730 3.087 0.00243 **
## CountryZAI 2.3040 18.1708 0.127 0.89928
## CountryMOZ 0.4440 13.4200 0.033 0.97365
## CountryMLI 9.2140 6.8331 1.348 0.17968
## CountryKEN 4.2640 7.4182 0.575 0.56634
## CountryETH 0.0940 18.1708 0.005 0.99588
## CountryGUI 2.4180 7.0730 0.342 0.73296
## CountryGHA 16.1551 6.8331 2.364 0.01943 *
## CountryBKF 14.6205 6.0825 2.404 0.01753 *
## ---
## Signif. codes: 0 '***' 0.001 '**' 0.01 '*' 0.05 '.' 0.1 ' ' 1
##
## (Dispersion parameter for gaussian family taken to be 300.1614)
##
## Null deviance: 50549 on 155 degrees of freedom
## Residual deviance: 42323 on 141 degrees of freedom
## AIC: 1348.8
##
## Number of Fisher Scoring iterations: 2

Anova(model1)

## Analysis of Deviance Table (Type II tests)
##
## Response: (Prev_Tv)
## LR Chisq Df Pr(>Chisq)
## Country 27.404 14 0.01705 *
## ---
## Signif. codes: 0 '***' 0.001 '**' 0.01 '*' 0.05 '.' 0.1 ' ' 1

#model1 is the best AICc= 1360.14
Anova(model2)

## Analysis of Deviance Table (Type II tests)
##
## Response: (Prev_Tv)
## LR Chisq Df Pr(>Chisq)
## Species 15.475 9 0.07868 .
## ---
## Signif. codes: 0 '***' 0.001 '**' 0.01 '*' 0.05 '.' 0.1 ' ' 1

#-------------------------------------------------------------------------
#Tz selection model
model1<-glm((Prev_Tz) ~ Country,data=data, family=gaussian())
model2<-glm((Prev_Tz) ~ Species,data=data, family=gaussian())
model3<-glm((Prev_Tz) ~ Country*Species,data=data, family=gaussian())
model4<-glm((Prev_Tz) ~ Country+Species,data=data, family=gaussian())
model5<-glm((Prev_Tz) ~ Country*Species+Localisation,data=data, family=gaussian())
model6<-glm((Prev_Tz) ~ Country+Species+Localisation,data=data, family=gaussian())

#AICc(model1, model2, model3, model4)
model.set <- list(model1, model2, model3, model4, model5, model6)
model.names <- c("model1", "model2","model3", "model4", "model5", "model6")

aictab(model.set, modnames = model.names)

##
## Model selection based on AICc:
##
## K AICc Delta_AICc AICcWt Cum.Wt LL
## model1 16 1260.42 0.00 1 1 -612.25
## model4 23 1272.68 12.25 0 1 -609.16
## model2 11 1283.49 23.07 0 1 -629.83
## model3 27 1284.10 23.68 0 1 -609.14
## model6 100 1444.79 184.37 0 1 -438.76
## model5 103 1495.05 234.63 0 1 -438.53

summary(model1)

##
## Call:
## glm(formula = (Prev_Tz) ~ Country, family = gaussian(), data = data)
##
## Deviance Residuals:
## Min 1Q Median 3Q Max
## -34.998 -3.008 -1.012 0.000 65.002
##
## Coefficients:
## Estimate Std. Error t value Pr(>|t|)
## (Intercept) 1.0250 4.0756 0.251 0.802
## CountryZAM -0.3450 13.5173 -0.026 0.980
## CountrySAF 2.9287 6.1134 0.479 0.633
## CountryUGA -0.0130 7.0591 -0.002 0.999
## CountryURT 4.0150 6.6554 0.603 0.547
## CountrySWA -1.0250 13.5173 -0.076 0.940
## CountrySEN 3.1030 5.2616 0.590 0.556
## CountryZAI -1.0250 13.5173 -0.076 0.940
## CountryMOZ -1.0250 9.9832 -0.103 0.918
## CountryMLI -0.4744 5.0832 -0.093 0.926
## CountryKEN -0.6000 5.5184 -0.109 0.914
## CountryETH 0.0650 13.5173 0.005 0.996
## CountryGUI -1.0250 5.2616 -0.195 0.846
## CountryGHA 33.9728 5.0832 6.683 5.02e-10 ***
## CountryBKF 1.9827 4.5248 0.438 0.662
## ---
## Signif. codes: 0 '***' 0.001 '**' 0.01 '*' 0.05 '.' 0.1 ' ' 1
##
## (Dispersion parameter for gaussian family taken to be 166.1055)
##
## Null deviance: 41046 on 155 degrees of freedom
## Residual deviance: 23421 on 141 degrees of freedom
## AIC: 1256.5
##
## Number of Fisher Scoring iterations: 2

Anova(model1)

## Analysis of Deviance Table (Type II tests)
##
## Response: (Prev_Tz)
## LR Chisq Df Pr(>Chisq)
## Country 106.11 14 3.167e-16 ***
## ---
## Signif. codes: 0 '***' 0.001 '**' 0.01 '*' 0.05 '.' 0.1 ' ' 1

#model1 is the best AICc= 1267.24
Anova(model2)

## Analysis of Deviance Table (Type II tests)
##
## Response: (Prev_Tz)
## LR Chisq Df Pr(>Chisq)
## Species 58.253 9 2.906e-09 ***
## ---
## Signif. codes: 0 '***' 0.001 '**' 0.01 '*' 0.05 '.' 0.1 ' ' 1

#-------------------------------------------------------------------------
#Tsg selection model
model1<-glm((Prev_Tsg) ~ Country,data=data, family=gaussian())
model2<-glm((Prev_Tsg) ~ Species,data=data, family=gaussian())
model3<-glm((Prev_Tsg) ~ Country*Species,data=data, family=gaussian())
model4<-glm((Prev_Tsg) ~ Country+Species,data=data, family=gaussian())
model5<-glm((Prev_Tsg) ~ Country*Species+Localisation,data=data, family=gaussian())
model6<-glm((Prev_Tsg) ~ Country+Species+Localisation,data=data, family=gaussian())

#AICc(model1, model2, model3, model4)
model.set <- list(model1, model2, model3, model4, model5, model6)
model.names <- c("model1", "model2","model3", "model4", "model5", "model6")

aictab(model.set, modnames = model.names)

##
## Model selection based on AICc:
##
## K AICc Delta_AICc AICcWt Cum.Wt LL
## model2 11 1203.33 0.00 0.78 0.78 -589.75
## model1 16 1205.83 2.50 0.22 1.00 -584.96
## model4 23 1214.77 11.45 0.00 1.00 -580.21
## model3 27 1223.72 20.40 0.00 1.00 -578.96
## model6 100 1364.93 161.60 0.00 1.00 -398.83
## model5 103 1409.96 206.63 0.00 1.00 -395.98

summary(model1)

##
## Call:
## glm(formula = (Prev_Tsg) ~ Country, family = gaussian(), data = data)
##
## Deviance Residuals:
## Min 1Q Median 3Q Max
## -16.34 -4.28 0.00 0.00 57.34
##
## Coefficients:
## Estimate Std. Error t value Pr(>|t|)
## (Intercept) 16.343 3.421 4.777 4.42e-06 ***
## CountryZAM 18.587 11.347 1.638 0.103654
## CountrySAF -5.649 5.132 -1.101 0.272872
## CountryUGA -11.315 5.926 -1.909 0.058245 .
## CountryURT -9.540 5.587 -1.707 0.089940 .
## CountrySWA -16.343 11.347 -1.440 0.152018
## CountrySEN -15.758 4.417 -3.568 0.000493 ***
## CountryZAI -16.343 11.347 -1.440 0.152018
## CountryMOZ -13.343 8.381 -1.592 0.113597
## CountryMLI -16.343 4.267 -3.830 0.000192 ***
## CountryKEN -1.720 4.633 -0.371 0.711037
## CountryETH -5.883 11.347 -0.518 0.604963
## CountryGUI -16.343 4.417 -3.700 0.000308 ***
## CountryGHA -16.343 4.267 -3.830 0.000192 ***
## CountryBKF -12.063 3.798 -3.176 0.001835 **
## ---
## Signif. codes: 0 '***' 0.001 '**' 0.01 '*' 0.05 '.' 0.1 ' ' 1
##
## (Dispersion parameter for gaussian family taken to be 117.0589)
##
## Null deviance: 21742 on 155 degrees of freedom
## Residual deviance: 16505 on 141 degrees of freedom
## AIC: 1201.9
##
## Number of Fisher Scoring iterations: 2

Anova(model1)

## Analysis of Deviance Table (Type II tests)
##
## Response: (Prev_Tsg)
## LR Chisq Df Pr(>Chisq)
## Country 44.737 14 4.5e-05 ***
## ---
## Signif. codes: 0 '***' 0.001 '**' 0.01 '*' 0.05 '.' 0.1 ' ' 1

#model2 is the best AICc=1210.49
Anova(model2)

## Analysis of Deviance Table (Type II tests)
##
## Response: (Prev_Tsg)
## LR Chisq Df Pr(>Chisq)
## Species 34.871 9 6.28e-05 ***
## ---
## Signif. codes: 0 '***' 0.001 '**' 0.01 '*' 0.05 '.' 0.1 ' ' 1

#-------------------------------------------------------------------------
#TcTv selection model
model1<-glm((Prev_TcTv) ~ Country,data=data, family=gaussian())
model2<-glm((Prev_TcTv) ~ Species,data=data, family=gaussian())
model3<-glm((Prev_TcTv) ~ Country*Species,data=data, family=gaussian())
model4<-glm((Prev_TcTv) ~ Country+Species,data=data, family=gaussian())
model5<-glm((Prev_TcTv) ~ Country*Species+Localisation,data=data, family=gaussian())
model6<-glm((Prev_TcTv) ~ Country+Species+Localisation,data=data, family=gaussian())

#AICc(model1, model2, model3, model4)
model.set <- list(model1, model2, model3, model4, model5, model6)
model.names <- c("model1", "model2","model3", "model4", "model5", "model6")

aictab(model.set, modnames = model.names)

##
## Model selection based on AICc:
##
## K AICc Delta_AICc AICcWt Cum.Wt LL
## model1 16 487.19 0.00 0.71 0.71 -225.64
## model2 11 489.12 1.93 0.27 0.98 -232.64
## model4 23 494.45 7.26 0.02 1.00 -220.04
## model3 27 502.03 14.84 0.00 1.00 -218.11
## model6 100 763.59 276.41 0.00 1.00 -98.16
## model5 103 814.32 327.13 0.00 1.00 -98.16

summary(model1)

##
## Call:
## glm(formula = (Prev_TcTv) ~ Country, family = gaussian(), data = data)
##
## Deviance Residuals:
## Min 1Q Median 3Q Max
## -2.0450 -0.2163 0.0000 0.0000 7.9550
##
## Coefficients:
## Estimate Std. Error t value Pr(>|t|)
## (Intercept) 5.955e-15 3.419e-01 0.000 1.000
## CountryZAM -1.108e-14 1.134e+00 0.000 1.000
## CountrySAF 2.362e-01 5.128e-01 0.461 0.646
## CountryUGA -5.150e-15 5.922e-01 0.000 1.000
## CountryURT -2.163e-14 5.583e-01 0.000 1.000
## CountrySWA -1.589e-14 1.134e+00 0.000 1.000
## CountrySEN -5.951e-15 4.414e-01 0.000 1.000
## CountryZAI -1.082e-14 1.134e+00 0.000 1.000
## CountryMOZ -1.477e-14 8.374e-01 0.000 1.000
## CountryMLI -5.405e-15 4.264e-01 0.000 1.000
## CountryKEN 2.045e+00 4.629e-01 4.418 1.97e-05 ***
## CountryETH -6.762e-15 1.134e+00 0.000 1.000
## CountryGUI -5.751e-15 4.414e-01 0.000 1.000
## CountryGHA -5.573e-15 4.264e-01 0.000 1.000
## CountryBKF 2.163e-01 3.796e-01 0.570 0.570
## ---
## Signif. codes: 0 '***' 0.001 '**' 0.01 '*' 0.05 '.' 0.1 ' ' 1
##
## (Dispersion parameter for gaussian family taken to be 1.168838)
##
## Null deviance: 209.26 on 155 degrees of freedom
## Residual deviance: 164.81 on 141 degrees of freedom
## AIC: 483.28
##
## Number of Fisher Scoring iterations: 2

Anova(model1)

## Analysis of Deviance Table (Type II tests)
##
## Response: (Prev_TcTv)
## LR Chisq Df Pr(>Chisq)
## Country 38.037 14 0.000513 ***
## ---
## Signif. codes: 0 '***' 0.001 '**' 0.01 '*' 0.05 '.' 0.1 ' ' 1

#model1 is the best AICc= 489.05
Anova(model2)

## Analysis of Deviance Table (Type II tests)
##
## Response: (Prev_TcTv)
## LR Chisq Df Pr(>Chisq)
## Species 23.46 9 0.005242 **
## ---
## Signif. codes: 0 '***' 0.001 '**' 0.01 '*' 0.05 '.' 0.1 ' ' 1

#-------------------------------------------------------------------------
#TcTz selection model
model1<-glm((Prev_TcTz) ~ Country,data=data, family=gaussian())
model2<-glm((Prev_TcTz) ~ Species,data=data, family=gaussian())
model3<-glm((Prev_TcTz) ~ Country*Species,data=data, family=gaussian())
model4<-glm((Prev_TcTz) ~ Country+Species,data=data, family=gaussian())
model5<-glm((Prev_TcTz) ~ Country*Species+Localisation,data=data, family=gaussian())
model6<-glm((Prev_TcTz) ~ Country+Species+Localisation,data=data, family=gaussian())

#AICc(model1, model2, model3, model4)
model.set <- list(model1, model2, model3, model4, model5, model6)
model.names <- c("model1", "model2","model3", "model4", "model5", "model6")

aictab(model.set, modnames = model.names)

##
## Model selection based on AICc:
##
## K AICc Delta_AICc AICcWt Cum.Wt LL
## model2 11 766.95 0.00 0.88 0.88 -371.56
## model1 16 770.90 3.96 0.12 1.00 -367.49
## model4 23 787.67 20.72 0.00 1.00 -366.65
## model3 27 799.03 32.08 0.00 1.00 -366.61
## model6 100 799.64 32.70 0.00 1.00 -116.19
## model5 103 847.28 80.33 0.00 1.00 -114.64

summary(model1)

##
## Call:
## glm(formula = (Prev_TcTz) ~ Country, family = gaussian(), data = data)
##
## Deviance Residuals:
## Min 1Q Median 3Q Max
## -3.7894 -0.3586 0.0000 0.0000 18.4306
##
## Coefficients:
## Estimate Std. Error t value Pr(>|t|)
## (Intercept) 2.194e-15 8.488e-01 0.000 1.000000
## CountryZAM -5.394e-15 2.815e+00 0.000 1.000000
## CountrySAF -3.226e-16 1.273e+00 0.000 1.000000
## CountryUGA -2.595e-15 1.470e+00 0.000 1.000000
## CountryURT 2.350e-01 1.386e+00 0.170 0.865610
## CountrySWA -5.910e-15 2.815e+00 0.000 1.000000
## CountrySEN -3.719e-15 1.096e+00 0.000 1.000000
## CountryZAI 1.144e-16 2.815e+00 0.000 1.000000
## CountryMOZ 4.144e-15 2.079e+00 0.000 1.000000
## CountryMLI -5.334e-16 1.059e+00 0.000 1.000000
## CountryKEN 3.342e-01 1.149e+00 0.291 0.771656
## CountryETH -2.672e-15 2.815e+00 0.000 1.000000
## CountryGUI -3.155e-15 1.096e+00 0.000 1.000000
## CountryGHA 3.789e+00 1.059e+00 3.580 0.000472 ***
## CountryBKF 3.586e-01 9.423e-01 0.381 0.704107
## ---
## Signif. codes: 0 '***' 0.001 '**' 0.01 '*' 0.05 '.' 0.1 ' ' 1
##
## (Dispersion parameter for gaussian family taken to be 7.204325)
##
## Null deviance: 1230.7 on 155 degrees of freedom
## Residual deviance: 1015.8 on 141 degrees of freedom
## AIC: 766.99
##
## Number of Fisher Scoring iterations: 2

Anova(model1)

## Analysis of Deviance Table (Type II tests)
##
## Response: (Prev_TcTz)
## LR Chisq Df Pr(>Chisq)
## Country 29.822 14 0.008075 **
## ---
## Signif. codes: 0 '***' 0.001 '**' 0.01 '*' 0.05 '.' 0.1 ' ' 1

#model2 is the best AICc= 770.69
Anova(model2)

## Analysis of Deviance Table (Type II tests)
##
## Response: (Prev_TcTz)
## LR Chisq Df Pr(>Chisq)
## Species 21.903 9 0.009192 **
## ---
## Signif. codes: 0 '***' 0.001 '**' 0.01 '*' 0.05 '.' 0.1 ' ' 1

#-------------------------------------------------------------------------
#TcTsg selection model
model1<-glm((Prev_TcTsg) ~ Country,data=data, family=gaussian())
model2<-glm((Prev_TcTsg) ~ Species,data=data, family=gaussian())
model3<-glm((Prev_TcTsg) ~ Country*Species,data=data, family=gaussian())
model4<-glm((Prev_TcTsg) ~ Country+Species,data=data, family=gaussian())
model5<-glm((Prev_TcTsg) ~ Country*Species+Localisation,data=data, family=gaussian())
model6<-glm((Prev_TcTsg) ~ Country+Species+Localisation,data=data, family=gaussian())

#AICc(model1, model2, model3, model4)
model.set <- list(model1, model2, model3, model4, model5, model6)
model.names <- c("model1", "model2","model3", "model4", "model5", "model6")

aictab(model.set, modnames = model.names)

##
## Model selection based on AICc:
##
## K AICc Delta_AICc AICcWt Cum.Wt LL
## model4 23 741.85 0.00 0.46 0.46 -343.74
## model5 103 742.24 0.39 0.38 0.85 -62.12
## model3 27 744.18 2.34 0.14 0.99 -339.19
## model2 11 749.43 7.59 0.01 1.00 -362.80
## model1 16 757.86 16.01 0.00 1.00 -360.97
## model6 100 949.58 207.73 0.00 1.00 -191.15

summary(model1)

##
## Call:
## glm(formula = (Prev_TcTsg) ~ Country, family = gaussian(), data = data)
##
## Deviance Residuals:
## Min 1Q Median 3Q Max
## -4.6633 -0.1012 0.0000 0.0000 16.4667
##
## Coefficients:
## Estimate Std. Error t value Pr(>|t|)
## (Intercept) 1.6800 0.8140 2.064 0.0409 *
## CountryZAM 5.8500 2.6998 2.167 0.0319 *
## CountrySAF -1.2250 1.2210 -1.003 0.3175
## CountryUGA -1.4680 1.4099 -1.041 0.2996
## CountryURT 2.9833 1.3293 2.244 0.0264 *
## CountrySWA -1.6800 2.6998 -0.622 0.5348
## CountrySEN -1.6800 1.0509 -1.599 0.1121
## CountryZAI -1.6800 2.6998 -0.622 0.5348
## CountryMOZ -1.6800 1.9939 -0.843 0.4009
## CountryMLI -1.6800 1.0153 -1.655 0.1002
## CountryKEN 2.7083 1.1022 2.457 0.0152 *
## CountryETH -1.0300 2.6998 -0.382 0.7034
## CountryGUI -1.6800 1.0509 -1.599 0.1121
## CountryGHA -1.6800 1.0153 -1.655 0.1002
## CountryBKF -1.5788 0.9037 -1.747 0.0828 .
## ---
## Signif. codes: 0 '***' 0.001 '**' 0.01 '*' 0.05 '.' 0.1 ' ' 1
##
## (Dispersion parameter for gaussian family taken to be 6.626342)
##
## Null deviance: 1299.26 on 155 degrees of freedom
## Residual deviance: 934.31 on 141 degrees of freedom
## AIC: 753.94
##
## Number of Fisher Scoring iterations: 2

Anova(model1)

## Analysis of Deviance Table (Type II tests)
##
## Response: (Prev_TcTsg)
## LR Chisq Df Pr(>Chisq)
## Country 55.075 14 8.404e-07 ***
## ---
## Signif. codes: 0 '***' 0.001 '**' 0.01 '*' 0.05 '.' 0.1 ' ' 1

#Model3 is the best AICc = 750.40
Anova(model2)

## Analysis of Deviance Table (Type II tests)
##
## Response: (Prev_TcTsg)
## LR Chisq Df Pr(>Chisq)
## Species 52.321 9 3.93e-08 ***
## ---
## Signif. codes: 0 '***' 0.001 '**' 0.01 '*' 0.05 '.' 0.1 ' ' 1

#-------------------------------------------------------------------------
#TvTz selection model
model1<-glm((Prev_TvTz) ~ Country,data=data, family=gaussian())
model2<-glm((Prev_TvTz) ~ Species,data=data, family=gaussian())
model3<-glm((Prev_TvTz) ~ Country*Species,data=data, family=gaussian())
model4<-glm((Prev_TvTz) ~ Country+Species,data=data, family=gaussian())
model5<-glm((Prev_TvTz) ~ Country*Species+Localisation,data=data, family=gaussian())
model6<-glm((Prev_TvTz) ~ Country+Species+Localisation,data=data, family=gaussian())

#AICc(model1, model2, model3, model4)
model.set <- list(model1, model2, model3, model4, model5, model6)
model.names <- c("model1", "model2","model3", "model4", "model5", "model6")

aictab(model.set, modnames = model.names)

##
## Model selection based on AICc:
##
## K AICc Delta_AICc AICcWt Cum.Wt LL
## model1 16 1143.40 0.00 0.66 0.66 -553.74
## model2 11 1144.76 1.36 0.34 1.00 -560.46
## model4 23 1161.80 18.40 0.00 1.00 -553.72
## model3 27 1173.25 29.84 0.00 1.00 -553.72
## model6 100 1571.22 427.82 0.00 1.00 -501.98
## model5 103 1621.93 478.53 0.00 1.00 -501.96

summary(model1)

##
## Call:
## glm(formula = (Prev_TvTz) ~ Country, family = gaussian(), data = data)
##
## Deviance Residuals:
## Min 1Q Median 3Q Max
## -13.394 -0.480 -0.086 0.000 69.936
##
## Coefficients:
## Estimate Std. Error t value Pr(>|t|)
## (Intercept) -3.951e-16 2.801e+00 0.000 1.000000
## CountryZAM -6.294e-15 9.290e+00 0.000 1.000000
## CountrySAF -5.800e-16 4.201e+00 0.000 1.000000
## CountryUGA 1.167e-15 4.851e+00 0.000 1.000000
## CountryURT 2.350e-01 4.574e+00 0.051 0.959097
## CountrySWA 1.059e-15 9.290e+00 0.000 1.000000
## CountrySEN 1.513e+00 3.616e+00 0.419 0.676212
## CountryZAI -5.622e-15 9.290e+00 0.000 1.000000
## CountryMOZ 4.382e-15 6.861e+00 0.000 1.000000
## CountryMLI -1.316e-16 3.493e+00 0.000 1.000000
## CountryKEN 8.583e-02 3.793e+00 0.023 0.981976
## CountryETH -8.040e-15 9.290e+00 0.000 1.000000
## CountryGUI 0.000e+00 3.616e+00 0.000 1.000000
## CountryGHA 1.339e+01 3.493e+00 3.834 0.000189 ***
## CountryBKF 4.800e-01 3.110e+00 0.154 0.877548
## ---
## Signif. codes: 0 '***' 0.001 '**' 0.01 '*' 0.05 '.' 0.1 ' ' 1
##
## (Dispersion parameter for gaussian family taken to be 78.45348)
##
## Null deviance: 13808 on 155 degrees of freedom
## Residual deviance: 11062 on 141 degrees of freedom
## AIC: 1139.5
##
## Number of Fisher Scoring iterations: 2

Anova(model1)

## Analysis of Deviance Table (Type II tests)
##
## Response: (Prev_TvTz)
## LR Chisq Df Pr(>Chisq)
## Country 35.005 14 0.001467 **
## ---
## Signif. codes: 0 '***' 0.001 '**' 0.01 '*' 0.05 '.' 0.1 ' ' 1

#model1 is the best AICc = 1149.47
Anova(model2)

## Analysis of Deviance Table (Type II tests)
##
## Response: (Prev_TvTz)
## LR Chisq Df Pr(>Chisq)
## Species 21.204 9 0.01177 *
## ---
## Signif. codes: 0 '***' 0.001 '**' 0.01 '*' 0.05 '.' 0.1 ' ' 1

#---------------------------------------------------------------------------
#TvTsg selection model
model1<-glm((Prev_TvTsg) ~ Country,data=data, family=gaussian())
model2<-glm((Prev_TvTsg) ~ Species,data=data, family=gaussian())
model3<-glm((Prev_TvTsg) ~ Country*Species,data=data, family=gaussian())
model4<-glm((Prev_TvTsg) ~ Country+Species,data=data, family=gaussian())
model5<-glm((Prev_TvTsg) ~ Country*Species+Localisation,data=data, family=gaussian())
model6<-glm((Prev_TvTsg) ~ Country+Species+Localisation,data=data, family=gaussian())

#AICc(model1, model2, model3, model4)
model.set <- list(model1, model2, model3, model4, model5, model6)
model.names <- c("model1", "model2","model3", "model4", "model5", "model6")

aictab(model.set, modnames = model.names)

##
## Model selection based on AICc:
##
## K AICc Delta_AICc AICcWt Cum.Wt LL
## model4 23 1083.12 0.00 0.97 0.97 -514.38
## model1 16 1090.71 7.59 0.02 0.99 -527.40
## model2 11 1093.22 10.10 0.01 1.00 -534.69
## model3 27 1094.08 10.96 0.00 1.00 -514.14
## model6 100 1402.52 319.40 0.00 1.00 -417.62
## model5 103 1451.91 368.79 0.00 1.00 -416.95

summary(model1)

##
## Call:
## glm(formula = (Prev_TvTsg) ~ Country, family = gaussian(), data = data)
##
## Deviance Residuals:
## Min 1Q Median 3Q Max
## -6.536 -0.104 0.000 0.000 60.134
##
## Coefficients:
## Estimate Std. Error t value Pr(>|t|)
## (Intercept) -2.773e-15 2.366e+00 0.000 1.000
## CountryZAM -6.818e-14 7.846e+00 0.000 1.000
## CountrySAF -1.283e-14 3.548e+00 0.000 1.000
## CountryUGA -7.956e-16 4.097e+00 0.000 1.000
## CountryURT 1.142e+00 3.863e+00 0.296 0.768
## CountrySWA 7.575e-15 7.846e+00 0.000 1.000
## CountrySEN 2.589e-15 3.054e+00 0.000 1.000
## CountryZAI -2.430e-14 7.846e+00 0.000 1.000
## CountryMOZ 1.000e+00 5.795e+00 0.173 0.863
## CountryMLI 1.225e-14 2.951e+00 0.000 1.000
## CountryKEN 1.042e-01 3.203e+00 0.033 0.974
## CountryETH 4.400e-01 7.846e+00 0.056 0.955
## CountryGUI -3.846e-15 3.054e+00 0.000 1.000
## CountryGHA 8.723e-15 2.951e+00 0.000 1.000
## CountryBKF 6.536e+00 2.626e+00 2.489 0.014 *
## ---
## Signif. codes: 0 '***' 0.001 '**' 0.01 '*' 0.05 '.' 0.1 ' ' 1
##
## (Dispersion parameter for gaussian family taken to be 55.96374)
##
## Null deviance: 9193.0 on 155 degrees of freedom
## Residual deviance: 7890.9 on 141 degrees of freedom
## AIC: 1086.8
##
## Number of Fisher Scoring iterations: 2

Anova(model1)

## Analysis of Deviance Table (Type II tests)
##
## Response: (Prev_TvTsg)
## LR Chisq Df Pr(>Chisq)
## Country 23.266 14 0.05607 .
## ---
## Signif. codes: 0 '***' 0.001 '**' 0.01 '*' 0.05 '.' 0.1 ' ' 1

#model4 is the best AICc = 1088.66
Anova(model2)

## Analysis of Deviance Table (Type II tests)
##
## Response: (Prev_TvTsg)
## LR Chisq Df Pr(>Chisq)
## Species 8.9004 9 0.4465

#----------------------------------------------------------------------------

#TzTsg selection model
model1<-glm((Prev_TzTsg) ~ Country,data=data, family=gaussian())
model2<-glm((Prev_TzTsg) ~ Species,data=data, family=gaussian())
model3<-glm((Prev_TzTsg) ~ Country*Species,data=data, family=gaussian())
model4<-glm((Prev_TzTsg) ~ Country+Species,data=data, family=gaussian())
model5<-glm((Prev_TzTsg) ~ Country*Species+Localisation,data=data, family=gaussian())
model6<-glm((Prev_TzTsg) ~ Country+Species+Localisation,data=data, family=gaussian())

#AICc(model1, model2, model3, model4)
model.set <- list(model1, model2, model3, model4, model5, model6)
model.names <- c("model1", "model2","model3", "model4", "model5", "model6")

aictab(model.set, modnames = model.names)

##
## Model selection based on AICc:
##
## K AICc Delta_AICc AICcWt Cum.Wt LL
## model2 11 881.89 0.00 0.98 0.98 -429.03
## model1 16 889.56 7.68 0.02 1.00 -426.82
## model4 23 900.88 18.99 0.00 1.00 -423.26
## model3 27 912.31 30.42 0.00 1.00 -423.25
## model6 100 1322.73 440.84 0.00 1.00 -377.73
## model5 103 1373.45 491.57 0.00 1.00 -377.73

summary(model1)

##
## Call:
## glm(formula = (Prev_TzTsg) ~ Country, family = gaussian(), data = data)
##
## Deviance Residuals:
## Min 1Q Median 3Q Max
## -1.8402 -0.8751 0.0000 0.0000 31.4898
##
## Coefficients:
## Estimate Std. Error t value Pr(>|t|)
## (Intercept) -2.222e-15 1.242e+00 0.000 1.000
## CountryZAM -1.814e-14 4.118e+00 0.000 1.000
## CountrySAF -1.850e-15 1.862e+00 0.000 1.000
## CountryUGA 1.740e-15 2.150e+00 0.000 1.000
## CountryURT 5.533e-01 2.027e+00 0.273 0.785
## CountrySWA 1.445e-15 4.118e+00 0.000 1.000
## CountrySEN 1.765e-15 1.603e+00 0.000 1.000
## CountryZAI -6.094e-15 4.118e+00 0.000 1.000
## CountryMOZ 1.689e-15 3.041e+00 0.000 1.000
## CountryMLI 5.128e-15 1.549e+00 0.000 1.000
## CountryKEN 2.750e-01 1.681e+00 0.164 0.870
## CountryETH 6.500e-01 4.118e+00 0.158 0.875
## CountryGUI 7.305e-16 1.603e+00 0.000 1.000
## CountryGHA 3.877e-15 1.549e+00 0.000 1.000
## CountryBKF 1.840e+00 1.378e+00 1.335 0.184
##
## (Dispersion parameter for gaussian family taken to be 15.41486)
##
## Null deviance: 2274.4 on 155 degrees of freedom
## Residual deviance: 2173.5 on 141 degrees of freedom
## AIC: 885.65
##
## Number of Fisher Scoring iterations: 2

Anova(model1)

## Analysis of Deviance Table (Type II tests)
##
## Response: (Prev_TzTsg)
## LR Chisq Df Pr(>Chisq)
## Country 6.5477 14 0.9507

#model2 is the best AICc = 886.37
Anova(model2)

## Analysis of Deviance Table (Type II tests)
##
## Response: (Prev_TzTsg)
## LR Chisq Df Pr(>Chisq)
## Species 2.5265 9 0.9802

#----------------------------------------------------------------------------

#TcTvTz selection model
model1<-glm((Prev_TcTvTz) ~ Country,data=data, family=gaussian())
model2<-glm((Prev_TcTvTz) ~ Species,data=data, family=gaussian())
model3<-glm((Prev_TcTvTz) ~ Country*Species,data=data, family=gaussian())
model4<-glm((Prev_TcTvTz) ~ Country+Species,data=data, family=gaussian())
model5<-glm((Prev_TcTvTz) ~ Country*Species+Localisation,data=data, family=gaussian())
model6<-glm((Prev_TcTvTz) ~ Country+Species+Localisation,data=data, family=gaussian())

#AICc(model1, model2, model3, model4)
model.set <- list(model1, model2, model3, model4, model5, model6)
model.names <- c("model1", "model2","model3", "model4", "model5", "model6")

aictab(model.set, modnames = model.names)

##
## Model selection based on AICc:
##
## K AICc Delta_AICc AICcWt Cum.Wt LL
## model2 11 265.19 0.00 1 1 -120.68
## model4 23 292.69 27.51 0 1 -119.17
## model3 27 304.14 38.96 0 1 -119.17
## model1 16 307.67 42.49 0 1 -135.88
## model6 100 672.31 407.12 0 1 -52.52
## model5 103 723.04 457.85 0 1 -52.52

summary(model1)

##
## Call:
## glm(formula = (Prev_TcTvTz) ~ Country, family = gaussian(), data = data)
##
## Deviance Residuals:
## Min 1Q Median 3Q Max
## -0.3856 -0.1549 0.0000 0.0000 5.2744
##
## Coefficients:
## Estimate Std. Error t value Pr(>|t|)
## (Intercept) 1.150e-17 1.923e-01 0.000 1.000
## CountryZAM -1.619e-15 6.378e-01 0.000 1.000
## CountrySAF -6.904e-16 2.885e-01 0.000 1.000
## CountryUGA -3.656e-16 3.331e-01 0.000 1.000
## CountryURT 2.697e-16 3.140e-01 0.000 1.000
## CountrySWA -1.530e-16 6.378e-01 0.000 1.000
## CountrySEN 1.372e-16 2.483e-01 0.000 1.000
## CountryZAI -7.134e-16 6.378e-01 0.000 1.000
## CountryMOZ -3.098e-16 4.711e-01 0.000 1.000
## CountryMLI 3.191e-16 2.398e-01 0.000 1.000
## CountryKEN 2.307e-16 2.604e-01 0.000 1.000
## CountryETH -9.883e-16 6.378e-01 0.000 1.000
## CountryGUI 1.577e-17 2.483e-01 0.000 1.000
## CountryGHA 3.856e-01 2.398e-01 1.607 0.110
## CountryBKF 1.549e-01 2.135e-01 0.725 0.469
##
## (Dispersion parameter for gaussian family taken to be 0.3698193)
##
## Null deviance: 54.666 on 155 degrees of freedom
## Residual deviance: 52.145 on 141 degrees of freedom
## AIC: 303.76
##
## Number of Fisher Scoring iterations: 2

Anova(model1)

## Analysis of Deviance Table (Type II tests)
##
## Response: (Prev_TcTvTz)
## LR Chisq Df Pr(>Chisq)
## Country 6.8186 14 0.9415

#model2 is the best AICc = 265.72
Anova(model2)

## Analysis of Deviance Table (Type II tests)
##
## Response: (Prev_TcTvTz)
## LR Chisq Df Pr(>Chisq)
## Species 39.998 9 7.605e-06 ***
## ---
## Signif. codes: 0 '***' 0.001 '**' 0.01 '*' 0.05 '.' 0.1 ' ' 1

## Statistics for Supplementary table 3

#======== Glm Tc per country
data$Country <- relevel(data$Country, ref= "BKF")
model1<-glm((Prev_Tc) ~ Country,data=data, family=gaussian())
summary(model1)

##
## Call:
## glm(formula = (Prev_Tc) ~ Country, family = gaussian(), data = data)
##
## Deviance Residuals:
## Min 1Q Median 3Q Max
## -13.594 -3.404 -0.107 0.000 46.596
##
## Coefficients:
## Estimate Std. Error t value Pr(>|t|)
## (Intercept) 3.4037 1.1110 3.064 0.00262 **
## CountryZIM 1.3603 2.5578 0.532 0.59568
## CountryZAM -2.0337 7.3697 -0.276 0.78298
## CountrySAF 1.4863 2.8052 0.530 0.59706
## CountryUGA -0.8017 3.4424 -0.233 0.81618
## CountryURT 6.3429 3.1750 1.998 0.04766 *
## CountrySWA -3.4037 7.3697 -0.462 0.64490
## CountrySEN -3.3031 2.1847 -1.512 0.13279
## CountryZAI -3.4037 7.3697 -0.462 0.64490
## CountryMOZ -0.4037 5.2700 -0.077 0.93904
## CountryMLI -3.2971 2.0453 -1.612 0.10919
## CountryKEN 11.2504 2.3785 4.730 5.39e-06 ***
## CountryETH 2.6963 7.3697 0.366 0.71502
## CountryGUI -3.4037 2.1847 -1.558 0.12148
## CountryGHA -3.0848 2.0453 -1.508 0.13372
## ---
## Signif. codes: 0 '***' 0.001 '**' 0.01 '*' 0.05 '.' 0.1 ' ' 1
##
## (Dispersion parameter for gaussian family taken to be 53.07752)
##
## Null deviance: 10017.9 on 155 degrees of freedom
## Residual deviance: 7483.9 on 141 degrees of freedom
## AIC: 1078.5
##
## Number of Fisher Scoring iterations: 2

data$Country <- relevel(data$Country, ref= "GHA")
model1<-glm((Prev_Tc) ~ Country,data=data, family=gaussian())
summary(model1)

##
## Call:
## glm(formula = (Prev_Tc) ~ Country, family = gaussian(), data = data)
##
## Deviance Residuals:
## Min 1Q Median 3Q Max
## -13.594 -3.404 -0.107 0.000 46.596
##
## Coefficients:
## Estimate Std. Error t value Pr(>|t|)
## (Intercept) 0.3189 1.7172 0.186 0.85294
## CountryBKF 3.0848 2.0453 1.508 0.13372
## CountryZIM 4.4451 2.8734 1.547 0.12411
## CountryZAM 1.0511 7.4851 0.140 0.88852
## CountrySAF 4.5711 3.0957 1.477 0.14201
## CountryUGA 2.2831 3.6830 0.620 0.53632
## CountryURT 9.4278 3.4344 2.745 0.00684 **
## CountrySWA -0.3189 7.4851 -0.043 0.96608
## CountrySEN -0.2182 2.5470 -0.086 0.93184
## CountryZAI -0.3189 7.4851 -0.043 0.96608
## CountryMOZ 2.6811 5.4302 0.494 0.62226
## CountryMLI -0.2122 2.4285 -0.087 0.93049
## CountryKEN 14.3353 2.7151 5.280 4.79e-07 ***
## CountryETH 5.7811 7.4851 0.772 0.44120
## CountryGUI -0.3189 2.5470 -0.125 0.90054
## ---
## Signif. codes: 0 '***' 0.001 '**' 0.01 '*' 0.05 '.' 0.1 ' ' 1
##
## (Dispersion parameter for gaussian family taken to be 53.07752)
##
## Null deviance: 10017.9 on 155 degrees of freedom
## Residual deviance: 7483.9 on 141 degrees of freedom
## AIC: 1078.5
##
## Number of Fisher Scoring iterations: 2

data$Country <- relevel(data$Country, ref= "GUI")
model1<-glm((Prev_Tc) ~ Country,data=data, family=gaussian())
summary(model1)

##
## Call:
## glm(formula = (Prev_Tc) ~ Country, family = gaussian(), data = data)
##
## Deviance Residuals:
## Min 1Q Median 3Q Max
## -13.594 -3.404 -0.107 0.000 46.596
##
## Coefficients:
## Estimate Std. Error t value Pr(>|t|)
## (Intercept) 1.204e-14 1.881e+00 0.000 1.00000
## CountryGHA 3.189e-01 2.547e+00 0.125 0.90054
## CountryBKF 3.404e+00 2.185e+00 1.558 0.12148
## CountryZIM 4.764e+00 2.974e+00 1.602 0.11145
## CountryZAM 1.370e+00 7.524e+00 0.182 0.85579
## CountrySAF 4.890e+00 3.190e+00 1.533 0.12748
## CountryUGA 2.602e+00 3.762e+00 0.692 0.49031
## CountryURT 9.747e+00 3.519e+00 2.770 0.00637 **
## CountrySWA 5.479e-15 7.524e+00 0.000 1.00000
## CountrySEN 1.007e-01 2.660e+00 0.038 0.96987
## CountryZAI -3.096e-14 7.524e+00 0.000 1.00000
## CountryMOZ 3.000e+00 5.484e+00 0.547 0.58523
## CountryMLI 1.067e-01 2.547e+00 0.042 0.96665
## CountryKEN 1.465e+01 2.822e+00 5.194 7.08e-07 ***
## CountryETH 6.100e+00 7.524e+00 0.811 0.41890
## ---
## Signif. codes: 0 '***' 0.001 '**' 0.01 '*' 0.05 '.' 0.1 ' ' 1
##
## (Dispersion parameter for gaussian family taken to be 53.07752)
##
## Null deviance: 10017.9 on 155 degrees of freedom
## Residual deviance: 7483.9 on 141 degrees of freedom
## AIC: 1078.5
##
## Number of Fisher Scoring iterations: 2

data$Country <- relevel(data$Country, ref= "ETH")
model1<-glm((Prev_Tc) ~ Country,data=data, family=gaussian())
summary(model1)

##
## Call:
## glm(formula = (Prev_Tc) ~ Country, family = gaussian(), data = data)
##
## Deviance Residuals:
## Min 1Q Median 3Q Max
## -13.594 -3.404 -0.107 0.000 46.596
##
## Coefficients:
## Estimate Std. Error t value Pr(>|t|)
## (Intercept) 6.100 7.285 0.837 0.404
## CountryGUI -6.100 7.524 -0.811 0.419
## CountryGHA -5.781 7.485 -0.772 0.441
## CountryBKF -2.696 7.370 -0.366 0.715
## CountryZIM -1.336 7.641 -0.175 0.861
## CountryZAM -4.730 10.303 -0.459 0.647
## CountrySAF -1.210 7.727 -0.157 0.876
## CountryUGA -3.498 7.981 -0.438 0.662
## CountryURT 3.647 7.869 0.463 0.644
## CountrySWA -6.100 10.303 -0.592 0.555
## CountrySEN -5.999 7.524 -0.797 0.427
## CountryZAI -6.100 10.303 -0.592 0.555
## CountryMOZ -3.100 8.923 -0.347 0.729
## CountryMLI -5.993 7.485 -0.801 0.425
## CountryKEN 8.554 7.583 1.128 0.261
##
## (Dispersion parameter for gaussian family taken to be 53.07752)
##
## Null deviance: 10017.9 on 155 degrees of freedom
## Residual deviance: 7483.9 on 141 degrees of freedom
## AIC: 1078.5
##
## Number of Fisher Scoring iterations: 2

data$Country <- relevel(data$Country, ref= "KEN")
model1<-glm((Prev_Tc) ~ Country,data=data, family=gaussian())
summary(model1)

##
## Call:
## glm(formula = (Prev_Tc) ~ Country, family = gaussian(), data = data)
##
## Deviance Residuals:
## Min 1Q Median 3Q Max
## -13.594 -3.404 -0.107 0.000 46.596
##
## Coefficients:
## Estimate Std. Error t value Pr(>|t|)
## (Intercept) 14.654 2.103 6.968 1.13e-10 ***
## CountryETH -8.554 7.583 -1.128 0.26120
## CountryGUI -14.654 2.822 -5.194 7.08e-07 ***
## CountryGHA -14.335 2.715 -5.280 4.79e-07 ***
## CountryBKF -11.250 2.379 -4.730 5.39e-06 ***
## CountryZIM -9.890 3.119 -3.170 0.00187 **
## CountryZAM -13.284 7.583 -1.752 0.08197 .
## CountrySAF -9.764 3.325 -2.936 0.00388 **
## CountryUGA -12.052 3.878 -3.108 0.00228 **
## CountryURT -4.908 3.643 -1.347 0.18007
## CountrySWA -14.654 7.583 -1.933 0.05530 .
## CountrySEN -14.554 2.822 -5.158 8.31e-07 ***
## CountryZAI -14.654 7.583 -1.933 0.05530 .
## CountryMOZ -11.654 5.564 -2.094 0.03801 *
## CountryMLI -14.547 2.715 -5.358 3.35e-07 ***
## ---
## Signif. codes: 0 '***' 0.001 '**' 0.01 '*' 0.05 '.' 0.1 ' ' 1
##
## (Dispersion parameter for gaussian family taken to be 53.07752)
##
## Null deviance: 10017.9 on 155 degrees of freedom
## Residual deviance: 7483.9 on 141 degrees of freedom
## AIC: 1078.5
##
## Number of Fisher Scoring iterations: 2

data$Country <- relevel(data$Country, ref= "MLI")
model1<-glm((Prev_Tc) ~ Country,data=data, family=gaussian())
summary(model1)

##
## Call:
## glm(formula = (Prev_Tc) ~ Country, family = gaussian(), data = data)
##
## Deviance Residuals:
## Min 1Q Median 3Q Max
## -13.594 -3.404 -0.107 0.000 46.596
##
## Coefficients:
## Estimate Std. Error t value Pr(>|t|)
## (Intercept) 0.1067 1.7172 0.062 0.95056
## CountryKEN 14.5475 2.7151 5.358 3.35e-07 ***
## CountryETH 5.9933 7.4851 0.801 0.42465
## CountryGUI -0.1067 2.5470 -0.042 0.96665
## CountryGHA 0.2122 2.4285 0.087 0.93049
## CountryBKF 3.2971 2.0453 1.612 0.10919
## CountryZIM 4.6573 2.8734 1.621 0.10729
## CountryZAM 1.2633 7.4851 0.169 0.86621
## CountrySAF 4.7833 3.0957 1.545 0.12455
## CountryUGA 2.4953 3.6830 0.678 0.49918
## CountryURT 9.6400 3.4344 2.807 0.00571 **
## CountrySWA -0.1067 7.4851 -0.014 0.98865
## CountrySEN -0.0060 2.5470 -0.002 0.99812
## CountryZAI -0.1067 7.4851 -0.014 0.98865
## CountryMOZ 2.8933 5.4302 0.533 0.59500
## ---
## Signif. codes: 0 '***' 0.001 '**' 0.01 '*' 0.05 '.' 0.1 ' ' 1
##
## (Dispersion parameter for gaussian family taken to be 53.07752)
##
## Null deviance: 10017.9 on 155 degrees of freedom
## Residual deviance: 7483.9 on 141 degrees of freedom
## AIC: 1078.5
##
## Number of Fisher Scoring iterations: 2

data$Country <- relevel(data$Country, ref= "MOZ")
model1<-glm((Prev_Tc) ~ Country,data=data, family=gaussian())
summary(model1)

##
## Call:
## glm(formula = (Prev_Tc) ~ Country, family = gaussian(), data = data)
##
## Deviance Residuals:
## Min 1Q Median 3Q Max
## -13.594 -3.404 -0.107 0.000 46.596
##
## Coefficients:
## Estimate Std. Error t value Pr(>|t|)
## (Intercept) 3.0000 5.1516 0.582 0.561
## CountryMLI -2.8933 5.4302 -0.533 0.595
## CountryKEN 11.6542 5.5643 2.094 0.038 *
## CountryETH 3.1000 8.9228 0.347 0.729
## CountryGUI -3.0000 5.4843 -0.547 0.585
## CountryGHA -2.6811 5.4302 -0.494 0.622
## CountryBKF 0.4037 5.2700 0.077 0.939
## CountryZIM 1.7640 5.6433 0.313 0.755
## CountryZAM -1.6300 8.9228 -0.183 0.855
## CountrySAF 1.8900 5.7596 0.328 0.743
## CountryUGA -0.3980 6.0954 -0.065 0.948
## CountryURT 6.7467 5.9485 1.134 0.259
## CountrySWA -3.0000 8.9228 -0.336 0.737
## CountrySEN -2.8993 5.4843 -0.529 0.598
## CountryZAI -3.0000 8.9228 -0.336 0.737
## ---
## Signif. codes: 0 '***' 0.001 '**' 0.01 '*' 0.05 '.' 0.1 ' ' 1
##
## (Dispersion parameter for gaussian family taken to be 53.07752)
##
## Null deviance: 10017.9 on 155 degrees of freedom
## Residual deviance: 7483.9 on 141 degrees of freedom
## AIC: 1078.5
##
## Number of Fisher Scoring iterations: 2

data$Country <- relevel(data$Country, ref= "ZAI")
model1<-glm((Prev_Tc) ~ Country,data=data, family=gaussian())
summary(model1)

##
## Call:
## glm(formula = (Prev_Tc) ~ Country, family = gaussian(), data = data)
##
## Deviance Residuals:
## Min 1Q Median 3Q Max
## -13.594 -3.404 -0.107 0.000 46.596
##
## Coefficients:
## Estimate Std. Error t value Pr(>|t|)
## (Intercept) -3.752e-14 7.285e+00 0.000 1.0000
## CountryMOZ 3.000e+00 8.923e+00 0.336 0.7372
## CountryMLI 1.067e-01 7.485e+00 0.014 0.9887
## CountryKEN 1.465e+01 7.583e+00 1.933 0.0553 .
## CountryETH 6.100e+00 1.030e+01 0.592 0.5548
## CountryGUI 3.013e-14 7.524e+00 0.000 1.0000
## CountryGHA 3.189e-01 7.485e+00 0.043 0.9661
## CountryBKF 3.404e+00 7.370e+00 0.462 0.6449
## CountryZIM 4.764e+00 7.641e+00 0.623 0.5340
## CountryZAM 1.370e+00 1.030e+01 0.133 0.8944
## CountrySAF 4.890e+00 7.727e+00 0.633 0.5279
## CountryUGA 2.602e+00 7.981e+00 0.326 0.7449
## CountryURT 9.747e+00 7.869e+00 1.239 0.2176
## CountrySWA 3.592e-14 1.030e+01 0.000 1.0000
## CountrySEN 1.007e-01 7.524e+00 0.013 0.9893
## ---
## Signif. codes: 0 '***' 0.001 '**' 0.01 '*' 0.05 '.' 0.1 ' ' 1
##
## (Dispersion parameter for gaussian family taken to be 53.07752)
##
## Null deviance: 10017.9 on 155 degrees of freedom
## Residual deviance: 7483.9 on 141 degrees of freedom
## AIC: 1078.5
##
## Number of Fisher Scoring iterations: 2

data$Country <- relevel(data$Country, ref= "SEN")
model1<-glm((Prev_Tc) ~ Country,data=data, family=gaussian())
summary(model1)

##
## Call:
## glm(formula = (Prev_Tc) ~ Country, family = gaussian(), data = data)
##
## Deviance Residuals:
## Min 1Q Median 3Q Max
## -13.594 -3.404 -0.107 0.000 46.596
##
## Coefficients:
## Estimate Std. Error t value Pr(>|t|)
## (Intercept) 0.1007 1.8811 0.054 0.95740
## CountryZAI -0.1007 7.5244 -0.013 0.98934
## CountryMOZ 2.8993 5.4843 0.529 0.59787
## CountryMLI 0.0060 2.5470 0.002 0.99812
## CountryKEN 14.5535 2.8216 5.158 8.31e-07 ***
## CountryETH 5.9993 7.5244 0.797 0.42661
## CountryGUI -0.1007 2.6603 -0.038 0.96987
## CountryGHA 0.2182 2.5470 0.086 0.93184
## CountryBKF 3.3031 2.1847 1.512 0.13279
## CountryZIM 4.6633 2.9743 1.568 0.11915
## CountryZAM 1.2693 7.5244 0.169 0.86628
## CountrySAF 4.7893 3.1895 1.502 0.13544
## CountryUGA 2.5013 3.7622 0.665 0.50722
## CountryURT 9.6460 3.5192 2.741 0.00692 **
## CountrySWA -0.1007 7.5244 -0.013 0.98934
## ---
## Signif. codes: 0 '***' 0.001 '**' 0.01 '*' 0.05 '.' 0.1 ' ' 1
##
## (Dispersion parameter for gaussian family taken to be 53.07752)
##
## Null deviance: 10017.9 on 155 degrees of freedom
## Residual deviance: 7483.9 on 141 degrees of freedom
## AIC: 1078.5
##
## Number of Fisher Scoring iterations: 2

data$Country <- relevel(data$Country, ref= "SWA")
model1<-glm((Prev_Tc) ~ Country,data=data, family=gaussian())
summary(model1)

##
## Call:
## glm(formula = (Prev_Tc) ~ Country, family = gaussian(), data = data)
##
## Deviance Residuals:
## Min 1Q Median 3Q Max
## -13.594 -3.404 -0.107 0.000 46.596
##
## Coefficients:
## Estimate Std. Error t value Pr(>|t|)
## (Intercept) 2.653e-14 7.285e+00 0.000 1.0000
## CountrySEN 1.007e-01 7.524e+00 0.013 0.9893
## CountryZAI -2.192e-14 1.030e+01 0.000 1.0000
## CountryMOZ 3.000e+00 8.923e+00 0.336 0.7372
## CountryMLI 1.067e-01 7.485e+00 0.014 0.9887
## CountryKEN 1.465e+01 7.583e+00 1.933 0.0553 .
## CountryETH 6.100e+00 1.030e+01 0.592 0.5548
## CountryGUI -2.485e-14 7.524e+00 0.000 1.0000
## CountryGHA 3.189e-01 7.485e+00 0.043 0.9661
## CountryBKF 3.404e+00 7.370e+00 0.462 0.6449
## CountryZIM 4.764e+00 7.641e+00 0.623 0.5340
## CountryZAM 1.370e+00 1.030e+01 0.133 0.8944
## CountrySAF 4.890e+00 7.727e+00 0.633 0.5279
## CountryUGA 2.602e+00 7.981e+00 0.326 0.7449
## CountryURT 9.747e+00 7.869e+00 1.239 0.2176
## ---
## Signif. codes: 0 '***' 0.001 '**' 0.01 '*' 0.05 '.' 0.1 ' ' 1
##
## (Dispersion parameter for gaussian family taken to be 53.07752)
##
## Null deviance: 10017.9 on 155 degrees of freedom
## Residual deviance: 7483.9 on 141 degrees of freedom
## AIC: 1078.5
##
## Number of Fisher Scoring iterations: 2

data$Country <- relevel(data$Country, ref= "URT")
model1<-glm((Prev_Tc) ~ Country,data=data, family=gaussian())
summary(model1)

##
## Call:
## glm(formula = (Prev_Tc) ~ Country, family = gaussian(), data = data)
##
## Deviance Residuals:
## Min 1Q Median 3Q Max
## -13.594 -3.404 -0.107 0.000 46.596
##
## Coefficients:
## Estimate Std. Error t value Pr(>|t|)
## (Intercept) 9.747 2.974 3.277 0.00132 **
## CountrySWA -9.747 7.869 -1.239 0.21756
## CountrySEN -9.646 3.519 -2.741 0.00692 **
## CountryZAI -9.747 7.869 -1.239 0.21756
## CountryMOZ -6.747 5.949 -1.134 0.25865
## CountryMLI -9.640 3.434 -2.807 0.00571 **
## CountryKEN 4.908 3.643 1.347 0.18007
## CountryETH -3.647 7.869 -0.463 0.64378
## CountryGUI -9.747 3.519 -2.770 0.00637 **
## CountryGHA -9.428 3.434 -2.745 0.00684 **
## CountryBKF -6.343 3.175 -1.998 0.04766 *
## CountryZIM -4.983 3.762 -1.324 0.18751
## CountryZAM -8.377 7.869 -1.064 0.28893
## CountrySAF -4.857 3.935 -1.234 0.21912
## CountryUGA -7.145 4.412 -1.620 0.10757
## ---
## Signif. codes: 0 '***' 0.001 '**' 0.01 '*' 0.05 '.' 0.1 ' ' 1
##
## (Dispersion parameter for gaussian family taken to be 53.07752)
##
## Null deviance: 10017.9 on 155 degrees of freedom
## Residual deviance: 7483.9 on 141 degrees of freedom
## AIC: 1078.5
##
## Number of Fisher Scoring iterations: 2

data$Country <- relevel(data$Country, ref= "UGA")
model1<-glm((Prev_Tc) ~ Country,data=data, family=gaussian())
summary(model1)

##
## Call:
## glm(formula = (Prev_Tc) ~ Country, family = gaussian(), data = data)
##
## Deviance Residuals:
## Min 1Q Median 3Q Max
## -13.594 -3.404 -0.107 0.000 46.596
##
## Coefficients:
## Estimate Std. Error t value Pr(>|t|)
## (Intercept) 2.6020 3.2581 0.799 0.42586
## CountryURT 7.1447 4.4115 1.620 0.10757
## CountrySWA -2.6020 7.9808 -0.326 0.74488
## CountrySEN -2.5013 3.7622 -0.665 0.50722
## CountryZAI -2.6020 7.9808 -0.326 0.74488
## CountryMOZ 0.3980 6.0954 0.065 0.94803
## CountryMLI -2.4953 3.6830 -0.678 0.49918
## CountryKEN 12.0522 3.8780 3.108 0.00228 **
## CountryETH 3.4980 7.9808 0.438 0.66184
## CountryGUI -2.6020 3.7622 -0.692 0.49031
## CountryGHA -2.2831 3.6830 -0.620 0.53632
## CountryBKF 0.8017 3.4424 0.233 0.81618
## CountryZIM 2.1620 3.9904 0.542 0.58881
## CountryZAM -1.2320 7.9808 -0.154 0.87754
## CountrySAF 2.2880 4.1533 0.551 0.58259
## ---
## Signif. codes: 0 '***' 0.001 '**' 0.01 '*' 0.05 '.' 0.1 ' ' 1
##
## (Dispersion parameter for gaussian family taken to be 53.07752)
##
## Null deviance: 10017.9 on 155 degrees of freedom
## Residual deviance: 7483.9 on 141 degrees of freedom
## AIC: 1078.5
##
## Number of Fisher Scoring iterations: 2

data$Country <- relevel(data$Country, ref= "SAF")
model1<-glm((Prev_Tc) ~ Country,data=data, family=gaussian())
summary(model1)

##
## Call:
## glm(formula = (Prev_Tc) ~ Country, family = gaussian(), data = data)
##
## Deviance Residuals:
## Min 1Q Median 3Q Max
## -13.594 -3.404 -0.107 0.000 46.596
##
## Coefficients:
## Estimate Std. Error t value Pr(>|t|)
## (Intercept) 4.890 2.576 1.898 0.05968 .
## CountryUGA -2.288 4.153 -0.551 0.58259
## CountryURT 4.857 3.935 1.234 0.21912
## CountrySWA -4.890 7.727 -0.633 0.52788
## CountrySEN -4.789 3.189 -1.502 0.13544
## CountryZAI -4.890 7.727 -0.633 0.52788
## CountryMOZ -1.890 5.760 -0.328 0.74329
## CountryMLI -4.783 3.096 -1.545 0.12455
## CountryKEN 9.764 3.325 2.936 0.00388 **
## CountryETH 1.210 7.727 0.157 0.87579
## CountryGUI -4.890 3.189 -1.533 0.12748
## CountryGHA -4.571 3.096 -1.477 0.14201
## CountryBKF -1.486 2.805 -0.530 0.59706
## CountryZIM -0.126 3.456 -0.036 0.97097
## CountryZAM -3.520 7.727 -0.456 0.64943
## ---
## Signif. codes: 0 '***' 0.001 '**' 0.01 '*' 0.05 '.' 0.1 ' ' 1
##
## (Dispersion parameter for gaussian family taken to be 53.07752)
##
## Null deviance: 10017.9 on 155 degrees of freedom
## Residual deviance: 7483.9 on 141 degrees of freedom
## AIC: 1078.5
##
## Number of Fisher Scoring iterations: 2

data$Country <- relevel(data$Country, ref= "ZAM")
model1<-glm((Prev_Tc) ~ Country,data=data, family=gaussian())
summary(model1)

##
## Call:
## glm(formula = (Prev_Tc) ~ Country, family = gaussian(), data = data)
##
## Deviance Residuals:
## Min 1Q Median 3Q Max
## -13.594 -3.404 -0.107 0.000 46.596
##
## Coefficients:
## Estimate Std. Error t value Pr(>|t|)
## (Intercept) 1.370 7.285 0.188 0.851
## CountrySAF 3.520 7.727 0.456 0.649
## CountryUGA 1.232 7.981 0.154 0.878
## CountryURT 8.377 7.869 1.064 0.289
## CountrySWA -1.370 10.303 -0.133 0.894
## CountrySEN -1.269 7.524 -0.169 0.866
## CountryZAI -1.370 10.303 -0.133 0.894
## CountryMOZ 1.630 8.923 0.183 0.855
## CountryMLI -1.263 7.485 -0.169 0.866
## CountryKEN 13.284 7.583 1.752 0.082 .
## CountryETH 4.730 10.303 0.459 0.647
## CountryGUI -1.370 7.524 -0.182 0.856
## CountryGHA -1.051 7.485 -0.140 0.889
## CountryBKF 2.034 7.370 0.276 0.783
## CountryZIM 3.394 7.641 0.444 0.658
## ---
## Signif. codes: 0 '***' 0.001 '**' 0.01 '*' 0.05 '.' 0.1 ' ' 1
##
## (Dispersion parameter for gaussian family taken to be 53.07752)
##
## Null deviance: 10017.9 on 155 degrees of freedom
## Residual deviance: 7483.9 on 141 degrees of freedom
## AIC: 1078.5
##
## Number of Fisher Scoring iterations: 2

data$Country <- relevel(data$Country, ref= "ZIM")
model1<-glm((Prev_Tc) ~ Country,data=data, family=gaussian())
summary(model1)

##
## Call:
## glm(formula = (Prev_Tc) ~ Country, family = gaussian(), data = data)
##
## Deviance Residuals:
## Min 1Q Median 3Q Max
## -13.594 -3.404 -0.107 0.000 46.596
##
## Coefficients:
## Estimate Std. Error t value Pr(>|t|)
## (Intercept) 4.764 2.304 2.068 0.04048 *
## CountryZAM -3.394 7.641 -0.444 0.65759
## CountrySAF 0.126 3.456 0.036 0.97097
## CountryUGA -2.162 3.990 -0.542 0.58881
## CountryURT 4.983 3.762 1.324 0.18751
## CountrySWA -4.764 7.641 -0.623 0.53398
## CountrySEN -4.663 2.974 -1.568 0.11915
## CountryZAI -4.764 7.641 -0.623 0.53398
## CountryMOZ -1.764 5.643 -0.313 0.75506
## CountryMLI -4.657 2.873 -1.621 0.10729
## CountryKEN 9.890 3.119 3.170 0.00187 **
## CountryETH 1.336 7.641 0.175 0.86145
## CountryGUI -4.764 2.974 -1.602 0.11145
## CountryGHA -4.445 2.873 -1.547 0.12411
## CountryBKF -1.360 2.558 -0.532 0.59568
## ---
## Signif. codes: 0 '***' 0.001 '**' 0.01 '*' 0.05 '.' 0.1 ' ' 1
##
## (Dispersion parameter for gaussian family taken to be 53.07752)
##
## Null deviance: 10017.9 on 155 degrees of freedom
## Residual deviance: 7483.9 on 141 degrees of freedom
## AIC: 1078.5
##
## Number of Fisher Scoring iterations: 2

#======== Glm Tv per country
data$Country <- relevel(data$Country, ref= "BKF")
model1<-glm((Prev_Tv) ~ Country,data=data, family=gaussian())
summary(model1)

##
## Call:
## glm(formula = (Prev_Tv) ~ Country, family = gaussian(), data = data)
##
## Deviance Residuals:
## Min 1Q Median 3Q Max
## -22.393 -9.770 -1.427 3.072 77.607
##
## Coefficients:
## Estimate Std. Error t value Pr(>|t|)
## (Intercept) 15.177 2.642 5.744 5.46e-08 ***
## CountryZIM -14.621 6.082 -2.404 0.0175 *
## CountryZAM -14.497 17.525 -0.827 0.4095
## CountrySAF -14.274 6.671 -2.140 0.0341 *
## CountryUGA -14.751 8.186 -1.802 0.0737 .
## CountryURT -13.403 7.550 -1.775 0.0780 .
## CountrySWA -15.177 17.525 -0.866 0.3880
## CountrySEN 7.216 5.195 1.389 0.1670
## CountryZAI -12.317 17.525 -0.703 0.4834
## CountryMOZ -14.177 12.532 -1.131 0.2599
## CountryMLI -5.407 4.864 -1.112 0.2682
## CountryKEN -10.357 5.656 -1.831 0.0692 .
## CountryETH -14.527 17.525 -0.829 0.4086
## CountryGUI -12.203 5.195 -2.349 0.0202 *
## CountryGHA 1.535 4.864 0.316 0.7528
## ---
## Signif. codes: 0 '***' 0.001 '**' 0.01 '*' 0.05 '.' 0.1 ' ' 1
##
## (Dispersion parameter for gaussian family taken to be 300.1614)
##
## Null deviance: 50549 on 155 degrees of freedom
## Residual deviance: 42323 on 141 degrees of freedom
## AIC: 1348.8
##
## Number of Fisher Scoring iterations: 2

data$Country <- relevel(data$Country, ref= "GHA")
model1<-glm((Prev_Tv) ~ Country,data=data, family=gaussian())
summary(model1)

##
## Call:
## glm(formula = (Prev_Tv) ~ Country, family = gaussian(), data = data)
##
## Deviance Residuals:
## Min 1Q Median 3Q Max
## -22.393 -9.770 -1.427 3.072 77.607
##
## Coefficients:
## Estimate Std. Error t value Pr(>|t|)
## (Intercept) 16.711 4.084 4.092 7.16e-05 ***
## CountryBKF -1.535 4.864 -0.316 0.7528
## CountryZIM -16.155 6.833 -2.364 0.0194 *
## CountryZAM -16.031 17.800 -0.901 0.3693
## CountrySAF -15.809 7.362 -2.147 0.0335 *
## CountryUGA -16.285 8.758 -1.859 0.0651 .
## CountryURT -14.938 8.167 -1.829 0.0695 .
## CountrySWA -16.711 17.800 -0.939 0.3494
## CountrySEN 5.682 6.057 0.938 0.3498
## CountryZAI -13.851 17.800 -0.778 0.4378
## CountryMOZ -15.711 12.913 -1.217 0.2258
## CountryMLI -6.941 5.775 -1.202 0.2314
## CountryKEN -11.891 6.457 -1.842 0.0676 .
## CountryETH -16.061 17.800 -0.902 0.3684
## CountryGUI -13.737 6.057 -2.268 0.0248 *
## ---
## Signif. codes: 0 '***' 0.001 '**' 0.01 '*' 0.05 '.' 0.1 ' ' 1
##
## (Dispersion parameter for gaussian family taken to be 300.1614)
##
## Null deviance: 50549 on 155 degrees of freedom
## Residual deviance: 42323 on 141 degrees of freedom
## AIC: 1348.8
##
## Number of Fisher Scoring iterations: 2

data$Country <- relevel(data$Country, ref= "GUI")
model1<-glm((Prev_Tv) ~ Country,data=data, family=gaussian())
summary(model1)

##
## Call:
## glm(formula = (Prev_Tv) ~ Country, family = gaussian(), data = data)
##
## Deviance Residuals:
## Min 1Q Median 3Q Max
## -22.393 -9.770 -1.427 3.072 77.607
##
## Coefficients:
## Estimate Std. Error t value Pr(>|t|)
## (Intercept) 2.974 4.473 0.665 0.50725
## CountryGHA 13.737 6.057 2.268 0.02485 *
## CountryBKF 12.203 5.195 2.349 0.02022 *
## CountryZIM -2.418 7.073 -0.342 0.73296
## CountryZAM -2.294 17.893 -0.128 0.89817
## CountrySAF -2.071 7.585 -0.273 0.78517
## CountryUGA -2.548 8.947 -0.285 0.77622
## CountryURT -1.201 8.369 -0.143 0.88613
## CountrySWA -2.974 17.893 -0.166 0.86823
## CountrySEN 19.419 6.326 3.070 0.00257 **
## CountryZAI -0.114 17.893 -0.006 0.99493
## CountryMOZ -1.974 13.042 -0.151 0.87991
## CountryMLI 6.796 6.057 1.122 0.26376
## CountryKEN 1.846 6.710 0.275 0.78363
## CountryETH -2.324 17.893 -0.130 0.89685
## ---
## Signif. codes: 0 '***' 0.001 '**' 0.01 '*' 0.05 '.' 0.1 ' ' 1
##
## (Dispersion parameter for gaussian family taken to be 300.1614)
##
## Null deviance: 50549 on 155 degrees of freedom
## Residual deviance: 42323 on 141 degrees of freedom
## AIC: 1348.8
##
## Number of Fisher Scoring iterations: 2

data$Country <- relevel(data$Country, ref= "ETH")
model1<-glm((Prev_Tv) ~ Country,data=data, family=gaussian())
summary(model1)

##
## Call:
## glm(formula = (Prev_Tv) ~ Country, family = gaussian(), data = data)
##
## Deviance Residuals:
## Min 1Q Median 3Q Max
## -22.393 -9.770 -1.427 3.072 77.607
##
## Coefficients:
## Estimate Std. Error t value Pr(>|t|)
## (Intercept) 0.6500 17.3252 0.038 0.970
## CountryGUI 2.3240 17.8934 0.130 0.897
## CountryGHA 16.0611 17.7999 0.902 0.368
## CountryBKF 14.5265 17.5255 0.829 0.409
## CountryZIM -0.0940 18.1708 -0.005 0.996
## CountryZAM 0.0300 24.5015 0.001 0.999
## CountrySAF 0.2525 18.3761 0.014 0.989
## CountryUGA -0.2240 18.9788 -0.012 0.991
## CountryURT 1.1233 18.7133 0.060 0.952
## CountrySWA -0.6500 24.5015 -0.027 0.979
## CountrySEN 21.7427 17.8934 1.215 0.226
## CountryZAI 2.2100 24.5015 0.090 0.928
## CountryMOZ 0.3500 21.2189 0.016 0.987
## CountryMLI 9.1200 17.7999 0.512 0.609
## CountryKEN 4.1700 18.0326 0.231 0.817
##
## (Dispersion parameter for gaussian family taken to be 300.1614)
##
## Null deviance: 50549 on 155 degrees of freedom
## Residual deviance: 42323 on 141 degrees of freedom
## AIC: 1348.8
##
## Number of Fisher Scoring iterations: 2

data$Country <- relevel(data$Country, ref= "KEN")
model1<-glm((Prev_Tv) ~ Country,data=data, family=gaussian())
summary(model1)

##
## Call:
## glm(formula = (Prev_Tv) ~ Country, family = gaussian(), data = data)
##
## Deviance Residuals:
## Min 1Q Median 3Q Max
## -22.393 -9.770 -1.427 3.072 77.607
##
## Coefficients:
## Estimate Std. Error t value Pr(>|t|)
## (Intercept) 4.820 5.001 0.964 0.33683
## CountryETH -4.170 18.033 -0.231 0.81746
## CountryGUI -1.846 6.710 -0.275 0.78363
## CountryGHA 11.891 6.457 1.842 0.06762 .
## CountryBKF 10.357 5.656 1.831 0.06922 .
## CountryZIM -4.264 7.418 -0.575 0.56634
## CountryZAM -4.140 18.033 -0.230 0.81875
## CountrySAF -3.918 7.908 -0.495 0.62109
## CountryUGA -4.394 9.222 -0.476 0.63448
## CountryURT -3.047 8.663 -0.352 0.72559
## CountrySWA -4.820 18.033 -0.267 0.78963
## CountrySEN 17.573 6.710 2.619 0.00979 **
## CountryZAI -1.960 18.033 -0.109 0.91360
## CountryMOZ -3.820 13.232 -0.289 0.77324
## CountryMLI 4.950 6.457 0.767 0.44457
## ---
## Signif. codes: 0 '***' 0.001 '**' 0.01 '*' 0.05 '.' 0.1 ' ' 1
##
## (Dispersion parameter for gaussian family taken to be 300.1614)
##
## Null deviance: 50549 on 155 degrees of freedom
## Residual deviance: 42323 on 141 degrees of freedom
## AIC: 1348.8
##
## Number of Fisher Scoring iterations: 2

data$Country <- relevel(data$Country, ref= "MLI")
model1<-glm((Prev_Tv) ~ Country,data=data, family=gaussian())
summary(model1)

##
## Call:
## glm(formula = (Prev_Tv) ~ Country, family = gaussian(), data = data)
##
## Deviance Residuals:
## Min 1Q Median 3Q Max
## -22.393 -9.770 -1.427 3.072 77.607
##
## Coefficients:
## Estimate Std. Error t value Pr(>|t|)
## (Intercept) 9.770 4.084 2.393 0.0181 *
## CountryKEN -4.950 6.457 -0.767 0.4446
## CountryETH -9.120 17.800 -0.512 0.6092
## CountryGUI -6.796 6.057 -1.122 0.2638
## CountryGHA 6.941 5.775 1.202 0.2314
## CountryBKF 5.407 4.864 1.112 0.2682
## CountryZIM -9.214 6.833 -1.348 0.1797
## CountryZAM -9.090 17.800 -0.511 0.6104
## CountrySAF -8.868 7.362 -1.205 0.2304
## CountryUGA -9.344 8.758 -1.067 0.2879
## CountryURT -7.997 8.167 -0.979 0.3292
## CountrySWA -9.770 17.800 -0.549 0.5840
## CountrySEN 12.623 6.057 2.084 0.0390 *
## CountryZAI -6.910 17.800 -0.388 0.6985
## CountryMOZ -8.770 12.913 -0.679 0.4982
## ---
## Signif. codes: 0 '***' 0.001 '**' 0.01 '*' 0.05 '.' 0.1 ' ' 1
##
## (Dispersion parameter for gaussian family taken to be 300.1614)
##
## Null deviance: 50549 on 155 degrees of freedom
## Residual deviance: 42323 on 141 degrees of freedom
## AIC: 1348.8
##
## Number of Fisher Scoring iterations: 2

data$Country <- relevel(data$Country, ref= "MOZ")
model1<-glm((Prev_Tv) ~ Country,data=data, family=gaussian())
summary(model1)

##
## Call:
## glm(formula = (Prev_Tv) ~ Country, family = gaussian(), data = data)
##
## Deviance Residuals:
## Min 1Q Median 3Q Max
## -22.393 -9.770 -1.427 3.072 77.607
##
## Coefficients:
## Estimate Std. Error t value Pr(>|t|)
## (Intercept) 1.0000 12.2507 0.082 0.935
## CountryMLI 8.7700 12.9134 0.679 0.498
## CountryKEN 3.8200 13.2323 0.289 0.773
## CountryETH -0.3500 21.2189 -0.016 0.987
## CountryGUI 1.9740 13.0419 0.151 0.880
## CountryGHA 15.7111 12.9134 1.217 0.226
## CountryBKF 14.1765 12.5324 1.131 0.260
## CountryZIM -0.4440 13.4200 -0.033 0.974
## CountryZAM -0.3200 21.2189 -0.015 0.988
## CountrySAF -0.0975 13.6967 -0.007 0.994
## CountryUGA -0.5740 14.4953 -0.040 0.968
## CountryURT 0.7733 14.1459 0.055 0.956
## CountrySWA -1.0000 21.2189 -0.047 0.962
## CountrySEN 21.3927 13.0419 1.640 0.103
## CountryZAI 1.8600 21.2189 0.088 0.930
##
## (Dispersion parameter for gaussian family taken to be 300.1614)
##
## Null deviance: 50549 on 155 degrees of freedom
## Residual deviance: 42323 on 141 degrees of freedom
## AIC: 1348.8
##
## Number of Fisher Scoring iterations: 2

data$Country <- relevel(data$Country, ref= "ZAI")
model1<-glm((Prev_Tv) ~ Country,data=data, family=gaussian())
summary(model1)

##
## Call:
## glm(formula = (Prev_Tv) ~ Country, family = gaussian(), data = data)
##
## Deviance Residuals:
## Min 1Q Median 3Q Max
## -22.393 -9.770 -1.427 3.072 77.607
##
## Coefficients:
## Estimate Std. Error t value Pr(>|t|)
## (Intercept) 2.860 17.325 0.165 0.869
## CountryMOZ -1.860 21.219 -0.088 0.930
## CountryMLI 6.910 17.800 0.388 0.698
## CountryKEN 1.960 18.033 0.109 0.914
## CountryETH -2.210 24.502 -0.090 0.928
## CountryGUI 0.114 17.893 0.006 0.995
## CountryGHA 13.851 17.800 0.778 0.438
## CountryBKF 12.316 17.526 0.703 0.483
## CountryZIM -2.304 18.171 -0.127 0.899
## CountryZAM -2.180 24.502 -0.089 0.929
## CountrySAF -1.958 18.376 -0.107 0.915
## CountryUGA -2.434 18.979 -0.128 0.898
## CountryURT -1.087 18.713 -0.058 0.954
## CountrySWA -2.860 24.502 -0.117 0.907
## CountrySEN 19.533 17.893 1.092 0.277
##
## (Dispersion parameter for gaussian family taken to be 300.1614)
##
## Null deviance: 50549 on 155 degrees of freedom
## Residual deviance: 42323 on 141 degrees of freedom
## AIC: 1348.8
##
## Number of Fisher Scoring iterations: 2

data$Country <- relevel(data$Country, ref= "SEN")
model1<-glm((Prev_Tv) ~ Country,data=data, family=gaussian())
summary(model1)

##
## Call:
## glm(formula = (Prev_Tv) ~ Country, family = gaussian(), data = data)
##
## Deviance Residuals:
## Min 1Q Median 3Q Max
## -22.393 -9.770 -1.427 3.072 77.607
##
## Coefficients:
## Estimate Std. Error t value Pr(>|t|)
## (Intercept) 22.393 4.473 5.006 1.63e-06 ***
## CountryZAI -19.533 17.893 -1.092 0.27686
## CountryMOZ -21.393 13.042 -1.640 0.10317
## CountryMLI -12.623 6.057 -2.084 0.03896 *
## CountryKEN -17.573 6.710 -2.619 0.00979 **
## CountryETH -21.743 17.893 -1.215 0.22635
## CountryGUI -19.419 6.326 -3.070 0.00257 **
## CountryGHA -5.682 6.057 -0.938 0.34984
## CountryBKF -7.216 5.195 -1.389 0.16703
## CountryZIM -21.837 7.073 -3.087 0.00243 **
## CountryZAM -21.713 17.893 -1.213 0.22699
## CountrySAF -21.490 7.585 -2.833 0.00528 **
## CountryUGA -21.967 8.947 -2.455 0.01529 *
## CountryURT -20.619 8.369 -2.464 0.01495 *
## CountrySWA -22.393 17.893 -1.251 0.21284
## ---
## Signif. codes: 0 '***' 0.001 '**' 0.01 '*' 0.05 '.' 0.1 ' ' 1
##
## (Dispersion parameter for gaussian family taken to be 300.1614)
##
## Null deviance: 50549 on 155 degrees of freedom
## Residual deviance: 42323 on 141 degrees of freedom
## AIC: 1348.8
##
## Number of Fisher Scoring iterations: 2

data$Country <- relevel(data$Country, ref= "SWA")
model1<-glm((Prev_Tv) ~ Country,data=data, family=gaussian())
summary(model1)

##
## Call:
## glm(formula = (Prev_Tv) ~ Country, family = gaussian(), data = data)
##
## Deviance Residuals:
## Min 1Q Median 3Q Max
## -22.393 -9.770 -1.427 3.072 77.607
##
## Coefficients:
## Estimate Std. Error t value Pr(>|t|)
## (Intercept) 1.314e-13 1.733e+01 0.000 1.000
## CountrySEN 2.239e+01 1.789e+01 1.251 0.213
## CountryZAI 2.860e+00 2.450e+01 0.117 0.907
## CountryMOZ 1.000e+00 2.122e+01 0.047 0.962
## CountryMLI 9.770e+00 1.780e+01 0.549 0.584
## CountryKEN 4.820e+00 1.803e+01 0.267 0.790
## CountryETH 6.500e-01 2.450e+01 0.027 0.979
## CountryGUI 2.974e+00 1.789e+01 0.166 0.868
## CountryGHA 1.671e+01 1.780e+01 0.939 0.349
## CountryBKF 1.518e+01 1.753e+01 0.866 0.388
## CountryZIM 5.560e-01 1.817e+01 0.031 0.976
## CountryZAM 6.800e-01 2.450e+01 0.028 0.978
## CountrySAF 9.025e-01 1.838e+01 0.049 0.961
## CountryUGA 4.260e-01 1.898e+01 0.022 0.982
## CountryURT 1.773e+00 1.871e+01 0.095 0.925
##
## (Dispersion parameter for gaussian family taken to be 300.1614)
##
## Null deviance: 50549 on 155 degrees of freedom
## Residual deviance: 42323 on 141 degrees of freedom
## AIC: 1348.8
##
## Number of Fisher Scoring iterations: 2

data$Country <- relevel(data$Country, ref= "URT")
model1<-glm((Prev_Tv) ~ Country,data=data, family=gaussian())
summary(model1)

##
## Call:
## glm(formula = (Prev_Tv) ~ Country, family = gaussian(), data = data)
##
## Deviance Residuals:
## Min 1Q Median 3Q Max
## -22.393 -9.770 -1.427 3.072 77.607
##
## Coefficients:
## Estimate Std. Error t value Pr(>|t|)
## (Intercept) 1.7733 7.0730 0.251 0.8024
## CountrySWA -1.7733 18.7133 -0.095 0.9246
## CountrySEN 20.6193 8.3689 2.464 0.0149 *
## CountryZAI 1.0867 18.7133 0.058 0.9538
## CountryMOZ -0.7733 14.1459 -0.055 0.9565
## CountryMLI 7.9967 8.1672 0.979 0.3292
## CountryKEN 3.0467 8.6626 0.352 0.7256
## CountryETH -1.1233 18.7133 -0.060 0.9522
## CountryGUI 1.2007 8.3689 0.143 0.8861
## CountryGHA 14.9378 8.1672 1.829 0.0695 .
## CountryBKF 13.4032 7.5503 1.775 0.0780 .
## CountryZIM -1.2173 8.9467 -0.136 0.8920
## CountryZAM -1.0933 18.7133 -0.058 0.9535
## CountrySAF -0.8708 9.3567 -0.093 0.9260
## CountryUGA -1.3473 10.4909 -0.128 0.8980
## ---
## Signif. codes: 0 '***' 0.001 '**' 0.01 '*' 0.05 '.' 0.1 ' ' 1
##
## (Dispersion parameter for gaussian family taken to be 300.1614)
##
## Null deviance: 50549 on 155 degrees of freedom
## Residual deviance: 42323 on 141 degrees of freedom
## AIC: 1348.8
##
## Number of Fisher Scoring iterations: 2

data$Country <- relevel(data$Country, ref= "UGA")
model1<-glm((Prev_Tv) ~ Country,data=data, family=gaussian())
summary(model1)

##
## Call:
## glm(formula = (Prev_Tv) ~ Country, family = gaussian(), data = data)
##
## Deviance Residuals:
## Min 1Q Median 3Q Max
## -22.393 -9.770 -1.427 3.072 77.607
##
## Coefficients:
## Estimate Std. Error t value Pr(>|t|)
## (Intercept) 0.4260 7.7481 0.055 0.9562
## CountryURT 1.3473 10.4909 0.128 0.8980
## CountrySWA -0.4260 18.9788 -0.022 0.9821
## CountrySEN 21.9667 8.9467 2.455 0.0153 *
## CountryZAI 2.4340 18.9788 0.128 0.8981
## CountryMOZ 0.5740 14.4953 0.040 0.9685
## CountryMLI 9.3440 8.7583 1.067 0.2879
## CountryKEN 4.3940 9.2220 0.476 0.6345
## CountryETH 0.2240 18.9788 0.012 0.9906
## CountryGUI 2.5480 8.9467 0.285 0.7762
## CountryGHA 16.2851 8.7583 1.859 0.0651 .
## CountryBKF 14.7505 8.1861 1.802 0.0737 .
## CountryZIM 0.1300 9.4894 0.014 0.9891
## CountryZAM 0.2540 18.9788 0.013 0.9893
## CountrySAF 0.4765 9.8769 0.048 0.9616
## ---
## Signif. codes: 0 '***' 0.001 '**' 0.01 '*' 0.05 '.' 0.1 ' ' 1
##
## (Dispersion parameter for gaussian family taken to be 300.1614)
##
## Null deviance: 50549 on 155 degrees of freedom
## Residual deviance: 42323 on 141 degrees of freedom
## AIC: 1348.8
##
## Number of Fisher Scoring iterations: 2

data$Country <- relevel(data$Country, ref= "SAF")
model1<-glm((Prev_Tv) ~ Country,data=data, family=gaussian())
summary(model1)

##
## Call:
## glm(formula = (Prev_Tv) ~ Country, family = gaussian(), data = data)
##
## Deviance Residuals:
## Min 1Q Median 3Q Max
## -22.393 -9.770 -1.427 3.072 77.607
##
## Coefficients:
## Estimate Std. Error t value Pr(>|t|)
## (Intercept) 0.9025 6.1254 0.147 0.88308
## CountryUGA -0.4765 9.8769 -0.048 0.96159
## CountryURT 0.8708 9.3567 0.093 0.92598
## CountrySWA -0.9025 18.3761 -0.049 0.96090
## CountrySEN 21.4902 7.5849 2.833 0.00528 **
## CountryZAI 1.9575 18.3761 0.107 0.91532
## CountryMOZ 0.0975 13.6967 0.007 0.99433
## CountryMLI 8.8675 7.3618 1.205 0.23040
## CountryKEN 3.9175 7.9078 0.495 0.62109
## CountryETH -0.2525 18.3761 -0.014 0.98906
## CountryGUI 2.0715 7.5849 0.273 0.78517
## CountryGHA 15.8086 7.3618 2.147 0.03347 *
## CountryBKF 14.2740 6.6709 2.140 0.03410 *
## CountryZIM -0.3465 8.2180 -0.042 0.96643
## CountryZAM -0.2225 18.3761 -0.012 0.99036
## ---
## Signif. codes: 0 '***' 0.001 '**' 0.01 '*' 0.05 '.' 0.1 ' ' 1
##
## (Dispersion parameter for gaussian family taken to be 300.1614)
##
## Null deviance: 50549 on 155 degrees of freedom
## Residual deviance: 42323 on 141 degrees of freedom
## AIC: 1348.8
##
## Number of Fisher Scoring iterations: 2

data$Country <- relevel(data$Country, ref= "ZAM")
model1<-glm((Prev_Tv) ~ Country,data=data, family=gaussian())
summary(model1)

##
## Call:
## glm(formula = (Prev_Tv) ~ Country, family = gaussian(), data = data)
##
## Deviance Residuals:
## Min 1Q Median 3Q Max
## -22.393 -9.770 -1.427 3.072 77.607
##
## Coefficients:
## Estimate Std. Error t value Pr(>|t|)
## (Intercept) 0.6800 17.3252 0.039 0.969
## CountrySAF 0.2225 18.3761 0.012 0.990
## CountryUGA -0.2540 18.9788 -0.013 0.989
## CountryURT 1.0933 18.7133 0.058 0.953
## CountrySWA -0.6800 24.5015 -0.028 0.978
## CountrySEN 21.7127 17.8934 1.213 0.227
## CountryZAI 2.1800 24.5015 0.089 0.929
## CountryMOZ 0.3200 21.2189 0.015 0.988
## CountryMLI 9.0900 17.7999 0.511 0.610
## CountryKEN 4.1400 18.0326 0.230 0.819
## CountryETH -0.0300 24.5015 -0.001 0.999
## CountryGUI 2.2940 17.8934 0.128 0.898
## CountryGHA 16.0311 17.7999 0.901 0.369
## CountryBKF 14.4965 17.5255 0.827 0.410
## CountryZIM -0.1240 18.1708 -0.007 0.995
##
## (Dispersion parameter for gaussian family taken to be 300.1614)
##
## Null deviance: 50549 on 155 degrees of freedom
## Residual deviance: 42323 on 141 degrees of freedom
## AIC: 1348.8
##
## Number of Fisher Scoring iterations: 2

data$Country <- relevel(data$Country, ref= "ZIM")
model1<-glm((Prev_Tv) ~ Country,data=data, family=gaussian())
summary(model1)

##
## Call:
## glm(formula = (Prev_Tv) ~ Country, family = gaussian(), data = data)
##
## Deviance Residuals:
## Min 1Q Median 3Q Max
## -22.393 -9.770 -1.427 3.072 77.607
##
## Coefficients:
## Estimate Std. Error t value Pr(>|t|)
## (Intercept) 0.5560 5.4787 0.101 0.91931
## CountryZAM 0.1240 18.1708 0.007 0.99456
## CountrySAF 0.3465 8.2180 0.042 0.96643
## CountryUGA -0.1300 9.4894 -0.014 0.98909
## CountryURT 1.2173 8.9467 0.136 0.89196
## CountrySWA -0.5560 18.1708 -0.031 0.97563
## CountrySEN 21.8367 7.0730 3.087 0.00243 **
## CountryZAI 2.3040 18.1708 0.127 0.89928
## CountryMOZ 0.4440 13.4200 0.033 0.97365
## CountryMLI 9.2140 6.8331 1.348 0.17968
## CountryKEN 4.2640 7.4182 0.575 0.56634
## CountryETH 0.0940 18.1708 0.005 0.99588
## CountryGUI 2.4180 7.0730 0.342 0.73296
## CountryGHA 16.1551 6.8331 2.364 0.01943 *
## CountryBKF 14.6205 6.0825 2.404 0.01753 *
## ---
## Signif. codes: 0 '***' 0.001 '**' 0.01 '*' 0.05 '.' 0.1 ' ' 1
##
## (Dispersion parameter for gaussian family taken to be 300.1614)
##
## Null deviance: 50549 on 155 degrees of freedom
## Residual deviance: 42323 on 141 degrees of freedom
## AIC: 1348.8
##
## Number of Fisher Scoring iterations: 2

#======== Glm Tz per country
data$Country <- relevel(data$Country, ref= "BKF")
model1<-glm((Prev_Tz) ~ Country,data=data, family=gaussian())
summary(model1)

##
## Call:
## glm(formula = (Prev_Tz) ~ Country, family = gaussian(), data = data)
##
## Deviance Residuals:
## Min 1Q Median 3Q Max
## -34.998 -3.008 -1.012 0.000 65.002
##
## Coefficients:
## Estimate Std. Error t value Pr(>|t|)
## (Intercept) 3.0077 1.9654 1.530 0.128
## CountryZIM -1.9827 4.5248 -0.438 0.662
## CountryZAM -2.3277 13.0372 -0.179 0.859
## CountrySAF 0.9461 4.9625 0.191 0.849
## CountryUGA -1.9957 6.0897 -0.328 0.744
## CountryURT 2.0323 5.6167 0.362 0.718
## CountrySWA -3.0077 13.0372 -0.231 0.818
## CountrySEN 1.1203 3.8648 0.290 0.772
## CountryZAI -3.0077 13.0372 -0.231 0.818
## CountryMOZ -3.0077 9.3229 -0.323 0.747
## CountryMLI -2.4571 3.6181 -0.679 0.498
## CountryKEN -2.5827 4.2077 -0.614 0.540
## CountryETH -1.9177 13.0372 -0.147 0.883
## CountryGUI -3.0077 3.8648 -0.778 0.438
## CountryGHA 31.9901 3.6181 8.842 3.46e-15 ***
## ---
## Signif. codes: 0 '***' 0.001 '**' 0.01 '*' 0.05 '.' 0.1 ' ' 1
##
## (Dispersion parameter for gaussian family taken to be 166.1055)
##
## Null deviance: 41046 on 155 degrees of freedom
## Residual deviance: 23421 on 141 degrees of freedom
## AIC: 1256.5
##
## Number of Fisher Scoring iterations: 2

data$Country <- relevel(data$Country, ref= "GHA")
model1<-glm((Prev_Tz) ~ Country,data=data, family=gaussian())
summary(model1)

##
## Call:
## glm(formula = (Prev_Tz) ~ Country, family = gaussian(), data = data)
##
## Deviance Residuals:
## Min 1Q Median 3Q Max
## -34.998 -3.008 -1.012 0.000 65.002
##
## Coefficients:
## Estimate Std. Error t value Pr(>|t|)
## (Intercept) 34.998 3.038 11.521 < 2e-16 ***
## CountryBKF -31.990 3.618 -8.842 3.46e-15 ***
## CountryZIM -33.973 5.083 -6.683 5.02e-10 ***
## CountryZAM -34.318 13.241 -2.592 0.010555 *
## CountrySAF -31.044 5.476 -5.669 7.83e-08 ***
## CountryUGA -33.986 6.515 -5.216 6.38e-07 ***
## CountryURT -29.958 6.076 -4.931 2.27e-06 ***
## CountrySWA -34.998 13.241 -2.643 0.009145 **
## CountrySEN -30.870 4.506 -6.851 2.09e-10 ***
## CountryZAI -34.998 13.241 -2.643 0.009145 **
## CountryMOZ -34.998 9.606 -3.643 0.000378 ***
## CountryMLI -34.447 4.296 -8.018 3.71e-13 ***
## CountryKEN -34.573 4.803 -7.198 3.33e-11 ***
## CountryETH -33.908 13.241 -2.561 0.011496 *
## CountryGUI -34.998 4.506 -7.767 1.50e-12 ***
## ---
## Signif. codes: 0 '***' 0.001 '**' 0.01 '*' 0.05 '.' 0.1 ' ' 1
##
## (Dispersion parameter for gaussian family taken to be 166.1055)
##
## Null deviance: 41046 on 155 degrees of freedom
## Residual deviance: 23421 on 141 degrees of freedom
## AIC: 1256.5
##
## Number of Fisher Scoring iterations: 2

data$Country <- relevel(data$Country, ref= "GUI")
model1<-glm((Prev_Tz) ~ Country,data=data, family=gaussian())
summary(model1)

##
## Call:
## glm(formula = (Prev_Tz) ~ Country, family = gaussian(), data = data)
##
## Deviance Residuals:
## Min 1Q Median 3Q Max
## -34.998 -3.008 -1.012 0.000 65.002
##
## Coefficients:
## Estimate Std. Error t value Pr(>|t|)
## (Intercept) 0.000e+00 3.328e+00 0.000 1.000
## CountryGHA 3.500e+01 4.506e+00 7.767 1.5e-12 ***
## CountryBKF 3.008e+00 3.865e+00 0.778 0.438
## CountryZIM 1.025e+00 5.262e+00 0.195 0.846
## CountryZAM 6.800e-01 1.331e+01 0.051 0.959
## CountrySAF 3.954e+00 5.642e+00 0.701 0.485
## CountryUGA 1.012e+00 6.655e+00 0.152 0.879
## CountryURT 5.040e+00 6.226e+00 0.810 0.420
## CountrySWA -8.168e-15 1.331e+01 0.000 1.000
## CountrySEN 4.128e+00 4.706e+00 0.877 0.382
## CountryZAI 3.757e-15 1.331e+01 0.000 1.000
## CountryMOZ 6.415e-16 9.702e+00 0.000 1.000
## CountryMLI 5.506e-01 4.506e+00 0.122 0.903
## CountryKEN 4.250e-01 4.992e+00 0.085 0.932
## CountryETH 1.090e+00 1.331e+01 0.082 0.935
## ---
## Signif. codes: 0 '***' 0.001 '**' 0.01 '*' 0.05 '.' 0.1 ' ' 1
##
## (Dispersion parameter for gaussian family taken to be 166.1055)
##
## Null deviance: 41046 on 155 degrees of freedom
## Residual deviance: 23421 on 141 degrees of freedom
## AIC: 1256.5
##
## Number of Fisher Scoring iterations: 2

data$Country <- relevel(data$Country, ref= "ETH")
model1<-glm((Prev_Tz) ~ Country,data=data, family=gaussian())
summary(model1)

##
## Call:
## glm(formula = (Prev_Tz) ~ Country, family = gaussian(), data = data)
##
## Deviance Residuals:
## Min 1Q Median 3Q Max
## -34.998 -3.008 -1.012 0.000 65.002
##
## Coefficients:
## Estimate Std. Error t value Pr(>|t|)
## (Intercept) 1.0900 12.8882 0.085 0.9327
## CountryGUI -1.0900 13.3109 -0.082 0.9349
## CountryGHA 33.9078 13.2414 2.561 0.0115 *
## CountryBKF 1.9177 13.0372 0.147 0.8833
## CountryZIM -0.0650 13.5173 -0.005 0.9962
## CountryZAM -0.4100 18.2267 -0.022 0.9821
## CountrySAF 2.8638 13.6700 0.209 0.8344
## CountryUGA -0.0780 14.1183 -0.006 0.9956
## CountryURT 3.9500 13.9208 0.284 0.7770
## CountrySWA -1.0900 18.2267 -0.060 0.9524
## CountrySEN 3.0380 13.3109 0.228 0.8198
## CountryZAI -1.0900 18.2267 -0.060 0.9524
## CountryMOZ -1.0900 15.7848 -0.069 0.9450
## CountryMLI -0.5394 13.2414 -0.041 0.9676
## CountryKEN -0.6650 13.4145 -0.050 0.9605
## ---
## Signif. codes: 0 '***' 0.001 '**' 0.01 '*' 0.05 '.' 0.1 ' ' 1
##
## (Dispersion parameter for gaussian family taken to be 166.1055)
##
## Null deviance: 41046 on 155 degrees of freedom
## Residual deviance: 23421 on 141 degrees of freedom
## AIC: 1256.5
##
## Number of Fisher Scoring iterations: 2

data$Country <- relevel(data$Country, ref= "KEN")
model1<-glm((Prev_Tz) ~ Country,data=data, family=gaussian())
summary(model1)

##
## Call:
## glm(formula = (Prev_Tz) ~ Country, family = gaussian(), data = data)
##
## Deviance Residuals:
## Min 1Q Median 3Q Max
## -34.998 -3.008 -1.012 0.000 65.002
##
## Coefficients:
## Estimate Std. Error t value Pr(>|t|)
## (Intercept) 0.4250 3.7205 0.114 0.909
## CountryETH 0.6650 13.4145 0.050 0.961
## CountryGUI -0.4250 4.9916 -0.085 0.932
## CountryGHA 34.5728 4.8031 7.198 3.33e-11 ***
## CountryBKF 2.5827 4.2077 0.614 0.540
## CountryZIM 0.6000 5.5184 0.109 0.914
## CountryZAM 0.2550 13.4145 0.019 0.985
## CountrySAF 3.5287 5.8826 0.600 0.550
## CountryUGA 0.5870 6.8603 0.086 0.932
## CountryURT 4.6150 6.4441 0.716 0.475
## CountrySWA -0.4250 13.4145 -0.032 0.975
## CountrySEN 3.7030 4.9916 0.742 0.459
## CountryZAI -0.4250 13.4145 -0.032 0.975
## CountryMOZ -0.4250 9.8435 -0.043 0.966
## CountryMLI 0.1256 4.8031 0.026 0.979
## ---
## Signif. codes: 0 '***' 0.001 '**' 0.01 '*' 0.05 '.' 0.1 ' ' 1
##
## (Dispersion parameter for gaussian family taken to be 166.1055)
##
## Null deviance: 41046 on 155 degrees of freedom
## Residual deviance: 23421 on 141 degrees of freedom
## AIC: 1256.5
##
## Number of Fisher Scoring iterations: 2

data$Country <- relevel(data$Country, ref= "MLI")
model1<-glm((Prev_Tz) ~ Country,data=data, family=gaussian())
summary(model1)

##
## Call:
## glm(formula = (Prev_Tz) ~ Country, family = gaussian(), data = data)
##
## Deviance Residuals:
## Min 1Q Median 3Q Max
## -34.998 -3.008 -1.012 0.000 65.002
##
## Coefficients:
## Estimate Std. Error t value Pr(>|t|)
## (Intercept) 0.5506 3.0378 0.181 0.856
## CountryKEN -0.1256 4.8031 -0.026 0.979
## CountryETH 0.5394 13.2414 0.041 0.968
## CountryGUI -0.5506 4.5058 -0.122 0.903
## CountryGHA 34.4472 4.2961 8.018 3.71e-13 ***
## CountryBKF 2.4571 3.6181 0.679 0.498
## CountryZIM 0.4744 5.0832 0.093 0.926
## CountryZAM 0.1294 13.2414 0.010 0.992
## CountrySAF 3.4032 5.4764 0.621 0.535
## CountryUGA 0.4614 6.5153 0.071 0.944
## CountryURT 4.4894 6.0756 0.739 0.461
## CountrySWA -0.5506 13.2414 -0.042 0.967
## CountrySEN 3.5774 4.5058 0.794 0.429
## CountryZAI -0.5506 13.2414 -0.042 0.967
## CountryMOZ -0.5506 9.6063 -0.057 0.954
## ---
## Signif. codes: 0 '***' 0.001 '**' 0.01 '*' 0.05 '.' 0.1 ' ' 1
##
## (Dispersion parameter for gaussian family taken to be 166.1055)
##
## Null deviance: 41046 on 155 degrees of freedom
## Residual deviance: 23421 on 141 degrees of freedom
## AIC: 1256.5
##
## Number of Fisher Scoring iterations: 2

data$Country <- relevel(data$Country, ref= "MOZ")
model1<-glm((Prev_Tz) ~ Country,data=data, family=gaussian())
summary(model1)

##
## Call:
## glm(formula = (Prev_Tz) ~ Country, family = gaussian(), data = data)
##
## Deviance Residuals:
## Min 1Q Median 3Q Max
## -34.998 -3.008 -1.012 0.000 65.002
##
## Coefficients:
## Estimate Std. Error t value Pr(>|t|)
## (Intercept) 3.176e-14 9.113e+00 0.000 1.000000
## CountryMLI 5.506e-01 9.606e+00 0.057 0.954378
## CountryKEN 4.250e-01 9.844e+00 0.043 0.965623
## CountryETH 1.090e+00 1.578e+01 0.069 0.945044
## CountryGUI -3.618e-14 9.702e+00 0.000 1.000000
## CountryGHA 3.500e+01 9.606e+00 3.643 0.000378 ***
## CountryBKF 3.008e+00 9.323e+00 0.323 0.747466
## CountryZIM 1.025e+00 9.983e+00 0.103 0.918368
## CountryZAM 6.800e-01 1.578e+01 0.043 0.965699
## CountrySAF 3.954e+00 1.019e+01 0.388 0.698571
## CountryUGA 1.012e+00 1.078e+01 0.094 0.925361
## CountryURT 5.040e+00 1.052e+01 0.479 0.632721
## CountrySWA -2.555e-14 1.578e+01 0.000 1.000000
## CountrySEN 4.128e+00 9.702e+00 0.425 0.671132
## CountryZAI -4.045e-14 1.578e+01 0.000 1.000000
## ---
## Signif. codes: 0 '***' 0.001 '**' 0.01 '*' 0.05 '.' 0.1 ' ' 1
##
## (Dispersion parameter for gaussian family taken to be 166.1055)
##
## Null deviance: 41046 on 155 degrees of freedom
## Residual deviance: 23421 on 141 degrees of freedom
## AIC: 1256.5
##
## Number of Fisher Scoring iterations: 2

data$Country <- relevel(data$Country, ref= "ZAI")
model1<-glm((Prev_Tz) ~ Country,data=data, family=gaussian())
summary(model1)

##
## Call:
## glm(formula = (Prev_Tz) ~ Country, family = gaussian(), data = data)
##
## Deviance Residuals:
## Min 1Q Median 3Q Max
## -34.998 -3.008 -1.012 0.000 65.002
##
## Coefficients:
## Estimate Std. Error t value Pr(>|t|)
## (Intercept) 9.204e-14 1.289e+01 0.000 1.00000
## CountryMOZ -9.421e-14 1.578e+01 0.000 1.00000
## CountryMLI 5.506e-01 1.324e+01 0.042 0.96689
## CountryKEN 4.250e-01 1.341e+01 0.032 0.97477
## CountryETH 1.090e+00 1.823e+01 0.060 0.95240
## CountryGUI -8.908e-14 1.331e+01 0.000 1.00000
## CountryGHA 3.500e+01 1.324e+01 2.643 0.00915 **
## CountryBKF 3.008e+00 1.304e+01 0.231 0.81788
## CountryZIM 1.025e+00 1.352e+01 0.076 0.93966
## CountryZAM 6.800e-01 1.823e+01 0.037 0.97029
## CountrySAF 3.954e+00 1.367e+01 0.289 0.77283
## CountryUGA 1.012e+00 1.412e+01 0.072 0.94296
## CountryURT 5.040e+00 1.392e+01 0.362 0.71786
## CountrySWA -9.613e-14 1.823e+01 0.000 1.00000
## CountrySEN 4.128e+00 1.331e+01 0.310 0.75693
## ---
## Signif. codes: 0 '***' 0.001 '**' 0.01 '*' 0.05 '.' 0.1 ' ' 1
##
## (Dispersion parameter for gaussian family taken to be 166.1055)
##
## Null deviance: 41046 on 155 degrees of freedom
## Residual deviance: 23421 on 141 degrees of freedom
## AIC: 1256.5
##
## Number of Fisher Scoring iterations: 2

data$Country <- relevel(data$Country, ref= "SEN")
model1<-glm((Prev_Tz) ~ Country,data=data, family=gaussian())
summary(model1)

##
## Call:
## glm(formula = (Prev_Tz) ~ Country, family = gaussian(), data = data)
##
## Deviance Residuals:
## Min 1Q Median 3Q Max
## -34.998 -3.008 -1.012 0.000 65.002
##
## Coefficients:
## Estimate Std. Error t value Pr(>|t|)
## (Intercept) 4.1280 3.3277 1.240 0.217
## CountryZAI -4.1280 13.3109 -0.310 0.757
## CountryMOZ -4.1280 9.7019 -0.425 0.671
## CountryMLI -3.5774 4.5058 -0.794 0.429
## CountryKEN -3.7030 4.9916 -0.742 0.459
## CountryETH -3.0380 13.3109 -0.228 0.820
## CountryGUI -4.1280 4.7061 -0.877 0.382
## CountryGHA 30.8698 4.5058 6.851 2.09e-10 ***
## CountryBKF -1.1203 3.8648 -0.290 0.772
## CountryZIM -3.1030 5.2616 -0.590 0.556
## CountryZAM -3.4480 13.3109 -0.259 0.796
## CountrySAF -0.1742 5.6424 -0.031 0.975
## CountryUGA -3.1160 6.6554 -0.468 0.640
## CountryURT 0.9120 6.2256 0.146 0.884
## CountrySWA -4.1280 13.3109 -0.310 0.757
## ---
## Signif. codes: 0 '***' 0.001 '**' 0.01 '*' 0.05 '.' 0.1 ' ' 1
##
## (Dispersion parameter for gaussian family taken to be 166.1055)
##
## Null deviance: 41046 on 155 degrees of freedom
## Residual deviance: 23421 on 141 degrees of freedom
## AIC: 1256.5
##
## Number of Fisher Scoring iterations: 2

data$Country <- relevel(data$Country, ref= "SWA")
model1<-glm((Prev_Tz) ~ Country,data=data, family=gaussian())
summary(model1)

##
## Call:
## glm(formula = (Prev_Tz) ~ Country, family = gaussian(), data = data)
##
## Deviance Residuals:
## Min 1Q Median 3Q Max
## -34.998 -3.008 -1.012 0.000 65.002
##
## Coefficients:
## Estimate Std. Error t value Pr(>|t|)
## (Intercept) 6.756e-15 1.289e+01 0.000 1.00000
## CountrySEN 4.128e+00 1.331e+01 0.310 0.75693
## CountryZAI -6.944e-15 1.823e+01 0.000 1.00000
## CountryMOZ -3.519e-15 1.578e+01 0.000 1.00000
## CountryMLI 5.506e-01 1.324e+01 0.042 0.96689
## CountryKEN 4.250e-01 1.341e+01 0.032 0.97477
## CountryETH 1.090e+00 1.823e+01 0.060 0.95240
## CountryGUI -1.781e-14 1.331e+01 0.000 1.00000
## CountryGHA 3.500e+01 1.324e+01 2.643 0.00915 **
## CountryBKF 3.008e+00 1.304e+01 0.231 0.81788
## CountryZIM 1.025e+00 1.352e+01 0.076 0.93966
## CountryZAM 6.800e-01 1.823e+01 0.037 0.97029
## CountrySAF 3.954e+00 1.367e+01 0.289 0.77283
## CountryUGA 1.012e+00 1.412e+01 0.072 0.94296
## CountryURT 5.040e+00 1.392e+01 0.362 0.71786
## ---
## Signif. codes: 0 '***' 0.001 '**' 0.01 '*' 0.05 '.' 0.1 ' ' 1
##
## (Dispersion parameter for gaussian family taken to be 166.1055)
##
## Null deviance: 41046 on 155 degrees of freedom
## Residual deviance: 23421 on 141 degrees of freedom
## AIC: 1256.5
##
## Number of Fisher Scoring iterations: 2

data$Country <- relevel(data$Country, ref= "URT")
model1<-glm((Prev_Tz) ~ Country,data=data, family=gaussian())
summary(model1)

##
## Call:
## glm(formula = (Prev_Tz) ~ Country, family = gaussian(), data = data)
##
## Deviance Residuals:
## Min 1Q Median 3Q Max
## -34.998 -3.008 -1.012 0.000 65.002
##
## Coefficients:
## Estimate Std. Error t value Pr(>|t|)
## (Intercept) 5.040 5.262 0.958 0.340
## CountrySWA -5.040 13.921 -0.362 0.718
## CountrySEN -0.912 6.226 -0.146 0.884
## CountryZAI -5.040 13.921 -0.362 0.718
## CountryMOZ -5.040 10.523 -0.479 0.633
## CountryMLI -4.489 6.076 -0.739 0.461
## CountryKEN -4.615 6.444 -0.716 0.475
## CountryETH -3.950 13.921 -0.284 0.777
## CountryGUI -5.040 6.226 -0.810 0.420
## CountryGHA 29.958 6.076 4.931 2.27e-06 ***
## CountryBKF -2.032 5.617 -0.362 0.718
## CountryZIM -4.015 6.655 -0.603 0.547
## CountryZAM -4.360 13.921 -0.313 0.755
## CountrySAF -1.086 6.960 -0.156 0.876
## CountryUGA -4.028 7.804 -0.516 0.607
## ---
## Signif. codes: 0 '***' 0.001 '**' 0.01 '*' 0.05 '.' 0.1 ' ' 1
##
## (Dispersion parameter for gaussian family taken to be 166.1055)
##
## Null deviance: 41046 on 155 degrees of freedom
## Residual deviance: 23421 on 141 degrees of freedom
## AIC: 1256.5
##
## Number of Fisher Scoring iterations: 2

data$Country <- relevel(data$Country, ref= "UGA")
model1<-glm((Prev_Tz) ~ Country,data=data, family=gaussian())
summary(model1)

##
## Call:
## glm(formula = (Prev_Tz) ~ Country, family = gaussian(), data = data)
##
## Deviance Residuals:
## Min 1Q Median 3Q Max
## -34.998 -3.008 -1.012 0.000 65.002
##
## Coefficients:
## Estimate Std. Error t value Pr(>|t|)
## (Intercept) 1.0120 5.7638 0.176 0.861
## CountryURT 4.0280 7.8042 0.516 0.607
## CountrySWA -1.0120 14.1183 -0.072 0.943
## CountrySEN 3.1160 6.6554 0.468 0.640
## CountryZAI -1.0120 14.1183 -0.072 0.943
## CountryMOZ -1.0120 10.7830 -0.094 0.925
## CountryMLI -0.4614 6.5153 -0.071 0.944
## CountryKEN -0.5870 6.8603 -0.086 0.932
## CountryETH 0.0780 14.1183 0.006 0.996
## CountryGUI -1.0120 6.6554 -0.152 0.879
## CountryGHA 33.9858 6.5153 5.216 6.38e-07 ***
## CountryBKF 1.9957 6.0897 0.328 0.744
## CountryZIM 0.0130 7.0591 0.002 0.999
## CountryZAM -0.3320 14.1183 -0.024 0.981
## CountrySAF 2.9417 7.3474 0.400 0.689
## ---
## Signif. codes: 0 '***' 0.001 '**' 0.01 '*' 0.05 '.' 0.1 ' ' 1
##
## (Dispersion parameter for gaussian family taken to be 166.1055)
##
## Null deviance: 41046 on 155 degrees of freedom
## Residual deviance: 23421 on 141 degrees of freedom
## AIC: 1256.5
##
## Number of Fisher Scoring iterations: 2

data$Country <- relevel(data$Country, ref= "SAF")
model1<-glm((Prev_Tz) ~ Country,data=data, family=gaussian())
summary(model1)

##
## Call:
## glm(formula = (Prev_Tz) ~ Country, family = gaussian(), data = data)
##
## Deviance Residuals:
## Min 1Q Median 3Q Max
## -34.998 -3.008 -1.012 0.000 65.002
##
## Coefficients:
## Estimate Std. Error t value Pr(>|t|)
## (Intercept) 3.9538 4.5567 0.868 0.387
## CountryUGA -2.9417 7.3474 -0.400 0.689
## CountryURT 1.0862 6.9604 0.156 0.876
## CountrySWA -3.9538 13.6700 -0.289 0.773
## CountrySEN 0.1743 5.6424 0.031 0.975
## CountryZAI -3.9537 13.6700 -0.289 0.773
## CountryMOZ -3.9537 10.1890 -0.388 0.699
## CountryMLI -3.4032 5.4764 -0.621 0.535
## CountryKEN -3.5288 5.8826 -0.600 0.550
## CountryETH -2.8637 13.6700 -0.209 0.834
## CountryGUI -3.9537 5.6424 -0.701 0.485
## CountryGHA 31.0440 5.4764 5.669 7.83e-08 ***
## CountryBKF -0.9461 4.9625 -0.191 0.849
## CountryZIM -2.9288 6.1134 -0.479 0.633
## CountryZAM -3.2737 13.6700 -0.239 0.811
## ---
## Signif. codes: 0 '***' 0.001 '**' 0.01 '*' 0.05 '.' 0.1 ' ' 1
##
## (Dispersion parameter for gaussian family taken to be 166.1055)
##
## Null deviance: 41046 on 155 degrees of freedom
## Residual deviance: 23421 on 141 degrees of freedom
## AIC: 1256.5
##
## Number of Fisher Scoring iterations: 2

data$Country <- relevel(data$Country, ref= "ZAM")
model1<-glm((Prev_Tz) ~ Country,data=data, family=gaussian())
summary(model1)

##
## Call:
## glm(formula = (Prev_Tz) ~ Country, family = gaussian(), data = data)
##
## Deviance Residuals:
## Min 1Q Median 3Q Max
## -34.998 -3.008 -1.012 0.000 65.002
##
## Coefficients:
## Estimate Std. Error t value Pr(>|t|)
## (Intercept) 0.6800 12.8882 0.053 0.9580
## CountrySAF 3.2737 13.6700 0.239 0.8111
## CountryUGA 0.3320 14.1183 0.024 0.9813
## CountryURT 4.3600 13.9208 0.313 0.7546
## CountrySWA -0.6800 18.2267 -0.037 0.9703
## CountrySEN 3.4480 13.3109 0.259 0.7960
## CountryZAI -0.6800 18.2267 -0.037 0.9703
## CountryMOZ -0.6800 15.7847 -0.043 0.9657
## CountryMLI -0.1294 13.2414 -0.010 0.9922
## CountryKEN -0.2550 13.4145 -0.019 0.9849
## CountryETH 0.4100 18.2267 0.022 0.9821
## CountryGUI -0.6800 13.3109 -0.051 0.9593
## CountryGHA 34.3178 13.2414 2.592 0.0106 *
## CountryBKF 2.3277 13.0372 0.179 0.8586
## CountryZIM 0.3450 13.5173 0.026 0.9797
## ---
## Signif. codes: 0 '***' 0.001 '**' 0.01 '*' 0.05 '.' 0.1 ' ' 1
##
## (Dispersion parameter for gaussian family taken to be 166.1055)
##
## Null deviance: 41046 on 155 degrees of freedom
## Residual deviance: 23421 on 141 degrees of freedom
## AIC: 1256.5
##
## Number of Fisher Scoring iterations: 2

data$Country <- relevel(data$Country, ref= "ZIM")
model1<-glm((Prev_Tz) ~ Country,data=data, family=gaussian())
summary(model1)

##
## Call:
## glm(formula = (Prev_Tz) ~ Country, family = gaussian(), data = data)
##
## Deviance Residuals:
## Min 1Q Median 3Q Max
## -34.998 -3.008 -1.012 0.000 65.002
##
## Coefficients:
## Estimate Std. Error t value Pr(>|t|)
## (Intercept) 1.0250 4.0756 0.251 0.802
## CountryZAM -0.3450 13.5173 -0.026 0.980
## CountrySAF 2.9287 6.1134 0.479 0.633
## CountryUGA -0.0130 7.0591 -0.002 0.999
## CountryURT 4.0150 6.6554 0.603 0.547
## CountrySWA -1.0250 13.5173 -0.076 0.940
## CountrySEN 3.1030 5.2616 0.590 0.556
## CountryZAI -1.0250 13.5173 -0.076 0.940
## CountryMOZ -1.0250 9.9832 -0.103 0.918
## CountryMLI -0.4744 5.0832 -0.093 0.926
## CountryKEN -0.6000 5.5184 -0.109 0.914
## CountryETH 0.0650 13.5173 0.005 0.996
## CountryGUI -1.0250 5.2616 -0.195 0.846
## CountryGHA 33.9728 5.0832 6.683 5.02e-10 ***
## CountryBKF 1.9827 4.5248 0.438 0.662
## ---
## Signif. codes: 0 '***' 0.001 '**' 0.01 '*' 0.05 '.' 0.1 ' ' 1
##
## (Dispersion parameter for gaussian family taken to be 166.1055)
##
## Null deviance: 41046 on 155 degrees of freedom
## Residual deviance: 23421 on 141 degrees of freedom
## AIC: 1256.5
##
## Number of Fisher Scoring iterations: 2

#======== Glm Tsg per country
data$Country <- relevel(data$Country, ref= "BKF")
model1<-glm((Prev_Tsg) ~ Country,data=data, family=gaussian())
summary(model1)

##
## Call:
## glm(formula = (Prev_Tsg) ~ Country, family = gaussian(), data = data)
##
## Deviance Residuals:
## Min 1Q Median 3Q Max
## -16.34 -4.28 0.00 0.00 57.34
##
## Coefficients:
## Estimate Std. Error t value Pr(>|t|)
## (Intercept) 4.2798 1.6499 2.594 0.01049 *
## CountryZIM 12.0632 3.7984 3.176 0.00184 **
## CountryZAM 30.6502 10.9445 2.801 0.00582 **
## CountrySAF 6.4140 4.1659 1.540 0.12589
## CountryUGA 0.7482 5.1122 0.146 0.88384
## CountryURT 2.5236 4.7151 0.535 0.59335
## CountrySWA -4.2798 10.9445 -0.391 0.69635
## CountrySEN -3.6944 3.2444 -1.139 0.25676
## CountryZAI -4.2798 10.9445 -0.391 0.69635
## CountryMOZ -1.2798 7.8264 -0.164 0.87034
## CountryMLI -4.2798 3.0374 -1.409 0.16102
## CountryKEN 10.3436 3.5323 2.928 0.00398 **
## CountryETH 6.1802 10.9445 0.565 0.57318
## CountryGUI -4.2798 3.2444 -1.319 0.18927
## CountryGHA -4.2798 3.0374 -1.409 0.16102
## ---
## Signif. codes: 0 '***' 0.001 '**' 0.01 '*' 0.05 '.' 0.1 ' ' 1
##
## (Dispersion parameter for gaussian family taken to be 117.0589)
##
## Null deviance: 21742 on 155 degrees of freedom
## Residual deviance: 16505 on 141 degrees of freedom
## AIC: 1201.9
##
## Number of Fisher Scoring iterations: 2

data$Country <- relevel(data$Country, ref= "GHA")
model1<-glm((Prev_Tsg) ~ Country,data=data, family=gaussian())
summary(model1)

##
## Call:
## glm(formula = (Prev_Tsg) ~ Country, family = gaussian(), data = data)
##
## Deviance Residuals:
## Min 1Q Median 3Q Max
## -16.34 -4.28 0.00 0.00 57.34
##
## Coefficients:
## Estimate Std. Error t value Pr(>|t|)
## (Intercept) -7.822e-15 2.550e+00 0.000 1.000000
## CountryBKF 4.280e+00 3.037e+00 1.409 0.161025
## CountryZIM 1.634e+01 4.267e+00 3.830 0.000192 ***
## CountryZAM 3.493e+01 1.112e+01 3.142 0.002043 **
## CountrySAF 1.069e+01 4.597e+00 2.326 0.021441 *
## CountryUGA 5.028e+00 5.469e+00 0.919 0.359516
## CountryURT 6.803e+00 5.100e+00 1.334 0.184385
## CountrySWA 1.775e-14 1.112e+01 0.000 1.000000
## CountrySEN 5.853e-01 3.782e+00 0.155 0.877241
## CountryZAI -2.223e-15 1.112e+01 0.000 1.000000
## CountryMOZ 3.000e+00 8.064e+00 0.372 0.710444
## CountryMLI 9.383e-15 3.606e+00 0.000 1.000000
## CountryKEN 1.462e+01 4.032e+00 3.627 0.000400 ***
## CountryETH 1.046e+01 1.112e+01 0.941 0.348315
## CountryGUI -1.164e-16 3.782e+00 0.000 1.000000
## ---
## Signif. codes: 0 '***' 0.001 '**' 0.01 '*' 0.05 '.' 0.1 ' ' 1
##
## (Dispersion parameter for gaussian family taken to be 117.0589)
##
## Null deviance: 21742 on 155 degrees of freedom
## Residual deviance: 16505 on 141 degrees of freedom
## AIC: 1201.9
##
## Number of Fisher Scoring iterations: 2

data$Country <- relevel(data$Country, ref= "GUI")
model1<-glm((Prev_Tsg) ~ Country,data=data, family=gaussian())
summary(model1)

##
## Call:
## glm(formula = (Prev_Tsg) ~ Country, family = gaussian(), data = data)
##
## Deviance Residuals:
## Min 1Q Median 3Q Max
## -16.34 -4.28 0.00 0.00 57.34
##
## Coefficients:
## Estimate Std. Error t value Pr(>|t|)
## (Intercept) 1.634e-14 2.794e+00 0.000 1.000000
## CountryGHA -1.959e-14 3.782e+00 0.000 1.000000
## CountryBKF 4.280e+00 3.244e+00 1.319 0.189268
## CountryZIM 1.634e+01 4.417e+00 3.700 0.000308 ***
## CountryZAM 3.493e+01 1.117e+01 3.126 0.002153 **
## CountrySAF 1.069e+01 4.737e+00 2.258 0.025505 *
## CountryUGA 5.028e+00 5.587e+00 0.900 0.369692
## CountryURT 6.803e+00 5.226e+00 1.302 0.195121
## CountrySWA 1.018e-14 1.117e+01 0.000 1.000000
## CountrySEN 5.853e-01 3.951e+00 0.148 0.882428
## CountryZAI 6.324e-15 1.117e+01 0.000 1.000000
## CountryMOZ 3.000e+00 8.145e+00 0.368 0.713168
## CountryMLI -1.717e-14 3.782e+00 0.000 1.000000
## CountryKEN 1.462e+01 4.190e+00 3.490 0.000645 ***
## CountryETH 1.046e+01 1.117e+01 0.936 0.350830
## ---
## Signif. codes: 0 '***' 0.001 '**' 0.01 '*' 0.05 '.' 0.1 ' ' 1
##
## (Dispersion parameter for gaussian family taken to be 117.0589)
##
## Null deviance: 21742 on 155 degrees of freedom
## Residual deviance: 16505 on 141 degrees of freedom
## AIC: 1201.9
##
## Number of Fisher Scoring iterations: 2

data$Country <- relevel(data$Country, ref= "ETH")
model1<-glm((Prev_Tsg) ~ Country,data=data, family=gaussian())
summary(model1)

##
## Call:
## glm(formula = (Prev_Tsg) ~ Country, family = gaussian(), data = data)
##
## Deviance Residuals:
## Min 1Q Median 3Q Max
## -16.34 -4.28 0.00 0.00 57.34
##
## Coefficients:
## Estimate Std. Error t value Pr(>|t|)
## (Intercept) 10.4600 10.8194 0.967 0.335
## CountryGUI -10.4600 11.1742 -0.936 0.351
## CountryGHA -10.4600 11.1159 -0.941 0.348
## CountryBKF -6.1802 10.9445 -0.565 0.573
## CountryZIM 5.8830 11.3475 0.518 0.605
## CountryZAM 24.4700 15.3009 1.599 0.112
## CountrySAF 0.2337 11.4757 0.020 0.984
## CountryUGA -5.4320 11.8520 -0.458 0.647
## CountryURT -3.6567 11.6863 -0.313 0.755
## CountrySWA -10.4600 15.3009 -0.684 0.495
## CountrySEN -9.8747 11.1742 -0.884 0.378
## CountryZAI -10.4600 15.3009 -0.684 0.495
## CountryMOZ -7.4600 13.2510 -0.563 0.574
## CountryMLI -10.4600 11.1159 -0.941 0.348
## CountryKEN 4.1633 11.2612 0.370 0.712
##
## (Dispersion parameter for gaussian family taken to be 117.0589)
##
## Null deviance: 21742 on 155 degrees of freedom
## Residual deviance: 16505 on 141 degrees of freedom
## AIC: 1201.9
##
## Number of Fisher Scoring iterations: 2

data$Country <- relevel(data$Country, ref= "KEN")
model1<-glm((Prev_Tsg) ~ Country,data=data, family=gaussian())
summary(model1)

##
## Call:
## glm(formula = (Prev_Tsg) ~ Country, family = gaussian(), data = data)
##
## Deviance Residuals:
## Min 1Q Median 3Q Max
## -16.34 -4.28 0.00 0.00 57.34
##
## Coefficients:
## Estimate Std. Error t value Pr(>|t|)
## (Intercept) 14.623 3.123 4.682 6.6e-06 ***
## CountryETH -4.163 11.261 -0.370 0.712155
## CountryGUI -14.623 4.190 -3.490 0.000645 ***
## CountryGHA -14.623 4.032 -3.627 0.000400 ***
## CountryBKF -10.344 3.532 -2.928 0.003976 **
## CountryZIM 1.720 4.633 0.371 0.711037
## CountryZAM 20.307 11.261 1.803 0.073485 .
## CountrySAF -3.930 4.938 -0.796 0.427528
## CountryUGA -9.595 5.759 -1.666 0.097908 .
## CountryURT -7.820 5.410 -1.446 0.150521
## CountrySWA -14.623 11.261 -1.299 0.196214
## CountrySEN -14.038 4.190 -3.350 0.001037 **
## CountryZAI -14.623 11.261 -1.299 0.196214
## CountryMOZ -11.623 8.263 -1.407 0.161747
## CountryMLI -14.623 4.032 -3.627 0.000400 ***
## ---
## Signif. codes: 0 '***' 0.001 '**' 0.01 '*' 0.05 '.' 0.1 ' ' 1
##
## (Dispersion parameter for gaussian family taken to be 117.0589)
##
## Null deviance: 21742 on 155 degrees of freedom
## Residual deviance: 16505 on 141 degrees of freedom
## AIC: 1201.9
##
## Number of Fisher Scoring iterations: 2

data$Country <- relevel(data$Country, ref= "MLI")
model1<-glm((Prev_Tsg) ~ Country,data=data, family=gaussian())
summary(model1)

##
## Call:
## glm(formula = (Prev_Tsg) ~ Country, family = gaussian(), data = data)
##
## Deviance Residuals:
## Min 1Q Median 3Q Max
## -16.34 -4.28 0.00 0.00 57.34
##
## Coefficients:
## Estimate Std. Error t value Pr(>|t|)
## (Intercept) 1.365e-14 2.550e+00 0.000 1.000000
## CountryKEN 1.462e+01 4.032e+00 3.627 0.000400 ***
## CountryETH 1.046e+01 1.112e+01 0.941 0.348315
## CountryGUI -3.449e-15 3.782e+00 0.000 1.000000
## CountryGHA -3.839e-15 3.606e+00 0.000 1.000000
## CountryBKF 4.280e+00 3.037e+00 1.409 0.161025
## CountryZIM 1.634e+01 4.267e+00 3.830 0.000192 ***
## CountryZAM 3.493e+01 1.112e+01 3.142 0.002043 **
## CountrySAF 1.069e+01 4.597e+00 2.326 0.021441 *
## CountryUGA 5.028e+00 5.469e+00 0.919 0.359516
## CountryURT 6.803e+00 5.100e+00 1.334 0.184385
## CountrySWA -2.645e-15 1.112e+01 0.000 1.000000
## CountrySEN 5.853e-01 3.782e+00 0.155 0.877241
## CountryZAI 3.185e-15 1.112e+01 0.000 1.000000
## CountryMOZ 3.000e+00 8.064e+00 0.372 0.710444
## ---
## Signif. codes: 0 '***' 0.001 '**' 0.01 '*' 0.05 '.' 0.1 ' ' 1
##
## (Dispersion parameter for gaussian family taken to be 117.0589)
##
## Null deviance: 21742 on 155 degrees of freedom
## Residual deviance: 16505 on 141 degrees of freedom
## AIC: 1201.9
##
## Number of Fisher Scoring iterations: 2

data$Country <- relevel(data$Country, ref= "MOZ")
model1<-glm((Prev_Tsg) ~ Country,data=data, family=gaussian())
summary(model1)

##
## Call:
## glm(formula = (Prev_Tsg) ~ Country, family = gaussian(), data = data)
##
## Deviance Residuals:
## Min 1Q Median 3Q Max
## -16.34 -4.28 0.00 0.00 57.34
##
## Coefficients:
## Estimate Std. Error t value Pr(>|t|)
## (Intercept) 3.000 7.650 0.392 0.6956
## CountryMLI -3.000 8.064 -0.372 0.7104
## CountryKEN 11.623 8.263 1.407 0.1617
## CountryETH 7.460 13.251 0.563 0.5743
## CountryGUI -3.000 8.145 -0.368 0.7132
## CountryGHA -3.000 8.064 -0.372 0.7104
## CountryBKF 1.280 7.826 0.164 0.8703
## CountryZIM 13.343 8.381 1.592 0.1136
## CountryZAM 31.930 13.251 2.410 0.0173 *
## CountrySAF 7.694 8.553 0.899 0.3699
## CountryUGA 2.028 9.052 0.224 0.8231
## CountryURT 3.803 8.834 0.431 0.6675
## CountrySWA -3.000 13.251 -0.226 0.8212
## CountrySEN -2.415 8.145 -0.296 0.7673
## CountryZAI -3.000 13.251 -0.226 0.8212
## ---
## Signif. codes: 0 '***' 0.001 '**' 0.01 '*' 0.05 '.' 0.1 ' ' 1
##
## (Dispersion parameter for gaussian family taken to be 117.0589)
##
## Null deviance: 21742 on 155 degrees of freedom
## Residual deviance: 16505 on 141 degrees of freedom
## AIC: 1201.9
##
## Number of Fisher Scoring iterations: 2

data$Country <- relevel(data$Country, ref= "ZAI")
model1<-glm((Prev_Tsg) ~ Country,data=data, family=gaussian())
summary(model1)

##
## Call:
## glm(formula = (Prev_Tsg) ~ Country, family = gaussian(), data = data)
##
## Deviance Residuals:
## Min 1Q Median 3Q Max
## -16.34 -4.28 0.00 0.00 57.34
##
## Coefficients:
## Estimate Std. Error t value Pr(>|t|)
## (Intercept) -4.883e-14 1.082e+01 0.000 1.0000
## CountryMOZ 3.000e+00 1.325e+01 0.226 0.8212
## CountryMLI 4.522e-14 1.112e+01 0.000 1.0000
## CountryKEN 1.462e+01 1.126e+01 1.299 0.1962
## CountryETH 1.046e+01 1.530e+01 0.684 0.4953
## CountryGUI 4.630e-14 1.117e+01 0.000 1.0000
## CountryGHA 3.256e-14 1.112e+01 0.000 1.0000
## CountryBKF 4.280e+00 1.094e+01 0.391 0.6964
## CountryZIM 1.634e+01 1.135e+01 1.440 0.1520
## CountryZAM 3.493e+01 1.530e+01 2.283 0.0239 *
## CountrySAF 1.069e+01 1.148e+01 0.932 0.3530
## CountryUGA 5.028e+00 1.185e+01 0.424 0.6720
## CountryURT 6.803e+00 1.169e+01 0.582 0.5614
## CountrySWA 6.981e-14 1.530e+01 0.000 1.0000
## CountrySEN 5.853e-01 1.117e+01 0.052 0.9583
## ---
## Signif. codes: 0 '***' 0.001 '**' 0.01 '*' 0.05 '.' 0.1 ' ' 1
##
## (Dispersion parameter for gaussian family taken to be 117.0589)
##
## Null deviance: 21742 on 155 degrees of freedom
## Residual deviance: 16505 on 141 degrees of freedom
## AIC: 1201.9
##
## Number of Fisher Scoring iterations: 2

data$Country <- relevel(data$Country, ref= "SEN")
model1<-glm((Prev_Tsg) ~ Country,data=data, family=gaussian())
summary(model1)

##
## Call:
## glm(formula = (Prev_Tsg) ~ Country, family = gaussian(), data = data)
##
## Deviance Residuals:
## Min 1Q Median 3Q Max
## -16.34 -4.28 0.00 0.00 57.34
##
## Coefficients:
## Estimate Std. Error t value Pr(>|t|)
## (Intercept) 0.5853 2.7936 0.210 0.834337
## CountryZAI -0.5853 11.1742 -0.052 0.958298
## CountryMOZ 2.4147 8.1445 0.296 0.767302
## CountryMLI -0.5853 3.7825 -0.155 0.877241
## CountryKEN 14.0380 4.1903 3.350 0.001037 **
## CountryETH 9.8747 11.1742 0.884 0.378361
## CountryGUI -0.5853 3.9507 -0.148 0.882428
## CountryGHA -0.5853 3.7825 -0.155 0.877241
## CountryBKF 3.6944 3.2444 1.139 0.256757
## CountryZIM 15.7577 4.4170 3.568 0.000493 ***
## CountryZAM 34.3447 11.1742 3.074 0.002540 **
## CountrySAF 10.1084 4.7367 2.134 0.034568 *
## CountryUGA 4.4427 5.5871 0.795 0.427854
## CountryURT 6.2180 5.2263 1.190 0.236140
## CountrySWA -0.5853 11.1742 -0.052 0.958298
## ---
## Signif. codes: 0 '***' 0.001 '**' 0.01 '*' 0.05 '.' 0.1 ' ' 1
##
## (Dispersion parameter for gaussian family taken to be 117.0589)
##
## Null deviance: 21742 on 155 degrees of freedom
## Residual deviance: 16505 on 141 degrees of freedom
## AIC: 1201.9
##
## Number of Fisher Scoring iterations: 2

data$Country <- relevel(data$Country, ref= "SWA")
model1<-glm((Prev_Tsg) ~ Country,data=data, family=gaussian())
summary(model1)

##
## Call:
## glm(formula = (Prev_Tsg) ~ Country, family = gaussian(), data = data)
##
## Deviance Residuals:
## Min 1Q Median 3Q Max
## -16.34 -4.28 0.00 0.00 57.34
##
## Coefficients:
## Estimate Std. Error t value Pr(>|t|)
## (Intercept) 1.065e-13 1.082e+01 0.000 1.0000
## CountrySEN 5.853e-01 1.117e+01 0.052 0.9583
## CountryZAI -9.969e-14 1.530e+01 0.000 1.0000
## CountryMOZ 3.000e+00 1.325e+01 0.226 0.8212
## CountryMLI -1.289e-13 1.112e+01 0.000 1.0000
## CountryKEN 1.462e+01 1.126e+01 1.299 0.1962
## CountryETH 1.046e+01 1.530e+01 0.684 0.4953
## CountryGUI -2.022e-13 1.117e+01 0.000 1.0000
## CountryGHA -9.150e-14 1.112e+01 0.000 1.0000
## CountryBKF 4.280e+00 1.094e+01 0.391 0.6964
## CountryZIM 1.634e+01 1.135e+01 1.440 0.1520
## CountryZAM 3.493e+01 1.530e+01 2.283 0.0239 *
## CountrySAF 1.069e+01 1.148e+01 0.932 0.3530
## CountryUGA 5.028e+00 1.185e+01 0.424 0.6720
## CountryURT 6.803e+00 1.169e+01 0.582 0.5614
## ---
## Signif. codes: 0 '***' 0.001 '**' 0.01 '*' 0.05 '.' 0.1 ' ' 1
##
## (Dispersion parameter for gaussian family taken to be 117.0589)
##
## Null deviance: 21742 on 155 degrees of freedom
## Residual deviance: 16505 on 141 degrees of freedom
## AIC: 1201.9
##
## Number of Fisher Scoring iterations: 2

data$Country <- relevel(data$Country, ref= "URT")
model1<-glm((Prev_Tsg) ~ Country,data=data, family=gaussian())
summary(model1)

##
## Call:
## glm(formula = (Prev_Tsg) ~ Country, family = gaussian(), data = data)
##
## Deviance Residuals:
## Min 1Q Median 3Q Max
## -16.34 -4.28 0.00 0.00 57.34
##
## Coefficients:
## Estimate Std. Error t value Pr(>|t|)
## (Intercept) 6.803 4.417 1.540 0.1257
## CountrySWA -6.803 11.686 -0.582 0.5614
## CountrySEN -6.218 5.226 -1.190 0.2361
## CountryZAI -6.803 11.686 -0.582 0.5614
## CountryMOZ -3.803 8.834 -0.431 0.6675
## CountryMLI -6.803 5.100 -1.334 0.1844
## CountryKEN 7.820 5.410 1.446 0.1505
## CountryETH 3.657 11.686 0.313 0.7548
## CountryGUI -6.803 5.226 -1.302 0.1951
## CountryGHA -6.803 5.100 -1.334 0.1844
## CountryBKF -2.524 4.715 -0.535 0.5933
## CountryZIM 9.540 5.587 1.707 0.0899 .
## CountryZAM 28.127 11.686 2.407 0.0174 *
## CountrySAF 3.890 5.843 0.666 0.5066
## CountryUGA -1.775 6.551 -0.271 0.7868
## ---
## Signif. codes: 0 '***' 0.001 '**' 0.01 '*' 0.05 '.' 0.1 ' ' 1
##
## (Dispersion parameter for gaussian family taken to be 117.0589)
##
## Null deviance: 21742 on 155 degrees of freedom
## Residual deviance: 16505 on 141 degrees of freedom
## AIC: 1201.9
##
## Number of Fisher Scoring iterations: 2

data$Country <- relevel(data$Country, ref= "UGA")
model1<-glm((Prev_Tsg) ~ Country,data=data, family=gaussian())
summary(model1)

##
## Call:
## glm(formula = (Prev_Tsg) ~ Country, family = gaussian(), data = data)
##
## Deviance Residuals:
## Min 1Q Median 3Q Max
## -16.34 -4.28 0.00 0.00 57.34
##
## Coefficients:
## Estimate Std. Error t value Pr(>|t|)
## (Intercept) 5.0280 4.8386 1.039 0.3005
## CountryURT 1.7753 6.5515 0.271 0.7868
## CountrySWA -5.0280 11.8520 -0.424 0.6720
## CountrySEN -4.4427 5.5871 -0.795 0.4279
## CountryZAI -5.0280 11.8520 -0.424 0.6720
## CountryMOZ -2.0280 9.0521 -0.224 0.8231
## CountryMLI -5.0280 5.4695 -0.919 0.3595
## CountryKEN 9.5953 5.7591 1.666 0.0979 .
## CountryETH 5.4320 11.8520 0.458 0.6474
## CountryGUI -5.0280 5.5871 -0.900 0.3697
## CountryGHA -5.0280 5.4695 -0.919 0.3595
## CountryBKF -0.7482 5.1122 -0.146 0.8838
## CountryZIM 11.3150 5.9260 1.909 0.0582 .
## CountryZAM 29.9020 11.8520 2.523 0.0127 *
## CountrySAF 5.6658 6.1680 0.919 0.3599
## ---
## Signif. codes: 0 '***' 0.001 '**' 0.01 '*' 0.05 '.' 0.1 ' ' 1
##
## (Dispersion parameter for gaussian family taken to be 117.0589)
##
## Null deviance: 21742 on 155 degrees of freedom
## Residual deviance: 16505 on 141 degrees of freedom
## AIC: 1201.9
##
## Number of Fisher Scoring iterations: 2

data$Country <- relevel(data$Country, ref= "SAF")
model1<-glm((Prev_Tsg) ~ Country,data=data, family=gaussian())
summary(model1)

##
## Call:
## glm(formula = (Prev_Tsg) ~ Country, family = gaussian(), data = data)
##
## Deviance Residuals:
## Min 1Q Median 3Q Max
## -16.34 -4.28 0.00 0.00 57.34
##
## Coefficients:
## Estimate Std. Error t value Pr(>|t|)
## (Intercept) 10.6938 3.8252 2.796 0.0059 **
## CountryUGA -5.6658 6.1680 -0.919 0.3599
## CountryURT -3.8904 5.8431 -0.666 0.5066
## CountrySWA -10.6938 11.4757 -0.932 0.3530
## CountrySEN -10.1084 4.7367 -2.134 0.0346 *
## CountryZAI -10.6937 11.4757 -0.932 0.3530
## CountryMOZ -7.6937 8.5535 -0.899 0.3699
## CountryMLI -10.6937 4.5974 -2.326 0.0214 *
## CountryKEN 3.9296 4.9383 0.796 0.4275
## CountryETH -0.2337 11.4757 -0.020 0.9838
## CountryGUI -10.6938 4.7367 -2.258 0.0255 *
## CountryGHA -10.6938 4.5974 -2.326 0.0214 *
## CountryBKF -6.4140 4.1659 -1.540 0.1259
## CountryZIM 5.6492 5.1321 1.101 0.2729
## CountryZAM 24.2362 11.4757 2.112 0.0365 *
## ---
## Signif. codes: 0 '***' 0.001 '**' 0.01 '*' 0.05 '.' 0.1 ' ' 1
##
## (Dispersion parameter for gaussian family taken to be 117.0589)
##
## Null deviance: 21742 on 155 degrees of freedom
## Residual deviance: 16505 on 141 degrees of freedom
## AIC: 1201.9
##
## Number of Fisher Scoring iterations: 2

data$Country <- relevel(data$Country, ref= "ZAM")
model1<-glm((Prev_Tsg) ~ Country,data=data, family=gaussian())
summary(model1)

##
## Call:
## glm(formula = (Prev_Tsg) ~ Country, family = gaussian(), data = data)
##
## Deviance Residuals:
## Min 1Q Median 3Q Max
## -16.34 -4.28 0.00 0.00 57.34
##
## Coefficients:
## Estimate Std. Error t value Pr(>|t|)
## (Intercept) 34.93 10.82 3.228 0.00155 **
## CountrySAF -24.24 11.48 -2.112 0.03645 *
## CountryUGA -29.90 11.85 -2.523 0.01275 *
## CountryURT -28.13 11.69 -2.407 0.01739 *
## CountrySWA -34.93 15.30 -2.283 0.02393 *
## CountrySEN -34.34 11.17 -3.074 0.00254 **
## CountryZAI -34.93 15.30 -2.283 0.02393 *
## CountryMOZ -31.93 13.25 -2.410 0.01726 *
## CountryMLI -34.93 11.12 -3.142 0.00204 **
## CountryKEN -20.31 11.26 -1.803 0.07348 .
## CountryETH -24.47 15.30 -1.599 0.11200
## CountryGUI -34.93 11.17 -3.126 0.00215 **
## CountryGHA -34.93 11.12 -3.142 0.00204 **
## CountryBKF -30.65 10.94 -2.801 0.00582 **
## CountryZIM -18.59 11.35 -1.638 0.10365
## ---
## Signif. codes: 0 '***' 0.001 '**' 0.01 '*' 0.05 '.' 0.1 ' ' 1
##
## (Dispersion parameter for gaussian family taken to be 117.0589)
##
## Null deviance: 21742 on 155 degrees of freedom
## Residual deviance: 16505 on 141 degrees of freedom
## AIC: 1201.9
##
## Number of Fisher Scoring iterations: 2

data$Country <- relevel(data$Country, ref= "ZIM")
model1<-glm((Prev_Tsg) ~ Country,data=data, family=gaussian())
summary(model1)

##
## Call:
## glm(formula = (Prev_Tsg) ~ Country, family = gaussian(), data = data)
##
## Deviance Residuals:
## Min 1Q Median 3Q Max
## -16.34 -4.28 0.00 0.00 57.34
##
## Coefficients:
## Estimate Std. Error t value Pr(>|t|)
## (Intercept) 16.343 3.421 4.777 4.42e-06 ***
## CountryZAM 18.587 11.347 1.638 0.103654
## CountrySAF -5.649 5.132 -1.101 0.272872
## CountryUGA -11.315 5.926 -1.909 0.058245 .
## CountryURT -9.540 5.587 -1.707 0.089940 .
## CountrySWA -16.343 11.347 -1.440 0.152018
## CountrySEN -15.758 4.417 -3.568 0.000493 ***
## CountryZAI -16.343 11.347 -1.440 0.152018
## CountryMOZ -13.343 8.381 -1.592 0.113597
## CountryMLI -16.343 4.267 -3.830 0.000192 ***
## CountryKEN -1.720 4.633 -0.371 0.711037
## CountryETH -5.883 11.347 -0.518 0.604963
## CountryGUI -16.343 4.417 -3.700 0.000308 ***
## CountryGHA -16.343 4.267 -3.830 0.000192 ***
## CountryBKF -12.063 3.798 -3.176 0.001835 **
## ---
## Signif. codes: 0 '***' 0.001 '**' 0.01 '*' 0.05 '.' 0.1 ' ' 1
##
## (Dispersion parameter for gaussian family taken to be 117.0589)
##
## Null deviance: 21742 on 155 degrees of freedom
## Residual deviance: 16505 on 141 degrees of freedom
## AIC: 1201.9
##
## Number of Fisher Scoring iterations: 2

#======== Glm TcTv per country
data$Country <- relevel(data$Country, ref= "BKF")
model1<-glm((Prev_TcTv) ~ Country,data=data, family=gaussian())
summary(model1)

##
## Call:
## glm(formula = (Prev_TcTv) ~ Country, family = gaussian(), data = data)
##
## Deviance Residuals:
## Min 1Q Median 3Q Max
## -2.0450 -0.2163 0.0000 0.0000 7.9550
##
## Coefficients:
## Estimate Std. Error t value Pr(>|t|)
## (Intercept) 0.21628 0.16487 1.312 0.192
## CountryZIM -0.21628 0.37956 -0.570 0.570
## CountryZAM -0.21628 1.09363 -0.198 0.844
## CountrySAF 0.01997 0.41628 0.048 0.962
## CountryUGA -0.21628 0.51083 -0.423 0.673
## CountryURT -0.21628 0.47116 -0.459 0.647
## CountrySWA -0.21628 1.09363 -0.198 0.844
## CountrySEN -0.21628 0.32420 -0.667 0.506
## CountryZAI -0.21628 1.09363 -0.198 0.844
## CountryMOZ -0.21628 0.78205 -0.277 0.783
## CountryMLI -0.21628 0.30351 -0.713 0.477
## CountryKEN 1.82872 0.35297 5.181 7.48e-07 ***
## CountryETH -0.21628 1.09363 -0.198 0.844
## CountryGUI -0.21628 0.32420 -0.667 0.506
## CountryGHA -0.21628 0.30351 -0.713 0.477
## ---
## Signif. codes: 0 '***' 0.001 '**' 0.01 '*' 0.05 '.' 0.1 ' ' 1
##
## (Dispersion parameter for gaussian family taken to be 1.168838)
##
## Null deviance: 209.26 on 155 degrees of freedom
## Residual deviance: 164.81 on 141 degrees of freedom
## AIC: 483.28
##
## Number of Fisher Scoring iterations: 2

data$Country <- relevel(data$Country, ref= "GHA")
model1<-glm((Prev_TcTv) ~ Country,data=data, family=gaussian())
summary(model1)

##
## Call:
## glm(formula = (Prev_TcTv) ~ Country, family = gaussian(), data = data)
##
## Deviance Residuals:
## Min 1Q Median 3Q Max
## -2.0450 -0.2163 0.0000 0.0000 7.9550
##
## Coefficients:
## Estimate Std. Error t value Pr(>|t|)
## (Intercept) -1.004e-15 2.548e-01 0.000 1.000
## CountryBKF 2.163e-01 3.035e-01 0.713 0.477
## CountryZIM 2.887e-15 4.264e-01 0.000 1.000
## CountryZAM 5.090e-16 1.111e+00 0.000 1.000
## CountrySAF 2.363e-01 4.594e-01 0.514 0.608
## CountryUGA -5.441e-15 5.465e-01 0.000 1.000
## CountryURT 2.003e-15 5.096e-01 0.000 1.000
## CountrySWA 2.463e-15 1.111e+00 0.000 1.000
## CountrySEN 1.552e-15 3.780e-01 0.000 1.000
## CountryZAI 2.394e-15 1.111e+00 0.000 1.000
## CountryMOZ 2.004e-15 8.058e-01 0.000 1.000
## CountryMLI 1.173e-15 3.604e-01 0.000 1.000
## CountryKEN 2.045e+00 4.029e-01 5.076 1.2e-06 ***
## CountryETH 4.605e-15 1.111e+00 0.000 1.000
## CountryGUI 1.950e-15 3.780e-01 0.000 1.000
## ---
## Signif. codes: 0 '***' 0.001 '**' 0.01 '*' 0.05 '.' 0.1 ' ' 1
##
## (Dispersion parameter for gaussian family taken to be 1.168838)
##
## Null deviance: 209.26 on 155 degrees of freedom
## Residual deviance: 164.81 on 141 degrees of freedom
## AIC: 483.28
##
## Number of Fisher Scoring iterations: 2

data$Country <- relevel(data$Country, ref= "GUI")
model1<-glm((Prev_TcTv) ~ Country,data=data, family=gaussian())
summary(model1)

##
## Call:
## glm(formula = (Prev_TcTv) ~ Country, family = gaussian(), data = data)
##
## Deviance Residuals:
## Min 1Q Median 3Q Max
## -2.0450 -0.2163 0.0000 0.0000 7.9550
##
## Coefficients:
## Estimate Std. Error t value Pr(>|t|)
## (Intercept) 4.237e-15 2.791e-01 0.000 1.000
## CountryGHA -4.980e-15 3.780e-01 0.000 1.000
## CountryBKF 2.163e-01 3.242e-01 0.667 0.506
## CountryZIM -3.309e-15 4.414e-01 0.000 1.000
## CountryZAM 2.952e-14 1.117e+00 0.000 1.000
## CountrySAF 2.362e-01 4.733e-01 0.499 0.618
## CountryUGA -6.271e-15 5.583e-01 0.000 1.000
## CountryURT -6.890e-15 5.222e-01 0.000 1.000
## CountrySWA -1.534e-14 1.117e+00 0.000 1.000
## CountrySEN -3.293e-15 3.948e-01 0.000 1.000
## CountryZAI -6.731e-15 1.117e+00 0.000 1.000
## CountryMOZ -1.760e-15 8.138e-01 0.000 1.000
## CountryMLI -4.293e-15 3.780e-01 0.000 1.000
## CountryKEN 2.045e+00 4.187e-01 4.884 2.78e-06 ***
## CountryETH 2.007e-15 1.117e+00 0.000 1.000
## ---
## Signif. codes: 0 '***' 0.001 '**' 0.01 '*' 0.05 '.' 0.1 ' ' 1
##
## (Dispersion parameter for gaussian family taken to be 1.168838)
##
## Null deviance: 209.26 on 155 degrees of freedom
## Residual deviance: 164.81 on 141 degrees of freedom
## AIC: 483.28
##
## Number of Fisher Scoring iterations: 2

data$Country <- relevel(data$Country, ref= "ETH")
model1<-glm((Prev_TcTv) ~ Country,data=data, family=gaussian())
summary(model1)

##
## Call:
## glm(formula = (Prev_TcTv) ~ Country, family = gaussian(), data = data)
##
## Deviance Residuals:
## Min 1Q Median 3Q Max
## -2.0450 -0.2163 0.0000 0.0000 7.9550
##
## Coefficients:
## Estimate Std. Error t value Pr(>|t|)
## (Intercept) -1.162e-14 1.081e+00 0.000 1.0000
## CountryGUI 1.175e-14 1.117e+00 0.000 1.0000
## CountryGHA 1.184e-14 1.111e+00 0.000 1.0000
## CountryBKF 2.163e-01 1.094e+00 0.198 0.8435
## CountryZIM 1.329e-14 1.134e+00 0.000 1.0000
## CountryZAM 2.493e-14 1.529e+00 0.000 1.0000
## CountrySAF 2.363e-01 1.147e+00 0.206 0.8371
## CountryUGA 1.201e-14 1.184e+00 0.000 1.0000
## CountryURT 1.389e-14 1.168e+00 0.000 1.0000
## CountrySWA 1.166e-14 1.529e+00 0.000 1.0000
## CountrySEN 1.064e-14 1.117e+00 0.000 1.0000
## CountryZAI 9.636e-15 1.529e+00 0.000 1.0000
## CountryMOZ 1.229e-14 1.324e+00 0.000 1.0000
## CountryMLI 1.196e-14 1.111e+00 0.000 1.0000
## CountryKEN 2.045e+00 1.125e+00 1.817 0.0713 .
## ---
## Signif. codes: 0 '***' 0.001 '**' 0.01 '*' 0.05 '.' 0.1 ' ' 1
##
## (Dispersion parameter for gaussian family taken to be 1.168838)
##
## Null deviance: 209.26 on 155 degrees of freedom
## Residual deviance: 164.81 on 141 degrees of freedom
## AIC: 483.28
##
## Number of Fisher Scoring iterations: 2

data$Country <- relevel(data$Country, ref= "KEN")
model1<-glm((Prev_TcTv) ~ Country,data=data, family=gaussian())
summary(model1)

##
## Call:
## glm(formula = (Prev_TcTv) ~ Country, family = gaussian(), data = data)
##
## Deviance Residuals:
## Min 1Q Median 3Q Max
## -2.0450 -0.2163 0.0000 0.0000 7.9550
##
## Coefficients:
## Estimate Std. Error t value Pr(>|t|)
## (Intercept) 2.0450 0.3121 6.552 9.86e-10 ***
## CountryETH -2.0450 1.1253 -1.817 0.071289 .
## CountryGUI -2.0450 0.4187 -4.884 2.78e-06 ***
## CountryGHA -2.0450 0.4029 -5.076 1.20e-06 ***
## CountryBKF -1.8287 0.3530 -5.181 7.48e-07 ***
## CountryZIM -2.0450 0.4629 -4.418 1.97e-05 ***
## CountryZAM -2.0450 1.1253 -1.817 0.071289 .
## CountrySAF -1.8087 0.4935 -3.665 0.000349 ***
## CountryUGA -2.0450 0.5755 -3.554 0.000517 ***
## CountryURT -2.0450 0.5406 -3.783 0.000228 ***
## CountrySWA -2.0450 1.1253 -1.817 0.071289 .
## CountrySEN -2.0450 0.4187 -4.884 2.78e-06 ***
## CountryZAI -2.0450 1.1253 -1.817 0.071289 .
## CountryMOZ -2.0450 0.8257 -2.477 0.014446 *
## CountryMLI -2.0450 0.4029 -5.076 1.20e-06 ***
## ---
## Signif. codes: 0 '***' 0.001 '**' 0.01 '*' 0.05 '.' 0.1 ' ' 1
##
## (Dispersion parameter for gaussian family taken to be 1.168838)
##
## Null deviance: 209.26 on 155 degrees of freedom
## Residual deviance: 164.81 on 141 degrees of freedom
## AIC: 483.28
##
## Number of Fisher Scoring iterations: 2

data$Country <- relevel(data$Country, ref= "MLI")
model1<-glm((Prev_TcTv) ~ Country,data=data, family=gaussian())
summary(model1)

##
## Call:
## glm(formula = (Prev_TcTv) ~ Country, family = gaussian(), data = data)
##
## Deviance Residuals:
## Min 1Q Median 3Q Max
## -2.0450 -0.2163 0.0000 0.0000 7.9550
##
## Coefficients:
## Estimate Std. Error t value Pr(>|t|)
## (Intercept) 1.333e-15 2.548e-01 0.000 1.000
## CountryKEN 2.045e+00 4.029e-01 5.076 1.2e-06 ***
## CountryETH -4.973e-15 1.111e+00 0.000 1.000
## CountryGUI -2.874e-16 3.780e-01 0.000 1.000
## CountryGHA 3.881e-15 3.604e-01 0.000 1.000
## CountryBKF 2.163e-01 3.035e-01 0.713 0.477
## CountryZIM -1.206e-15 4.264e-01 0.000 1.000
## CountryZAM -2.583e-15 1.111e+00 0.000 1.000
## CountrySAF 2.362e-01 4.594e-01 0.514 0.608
## CountryUGA -6.729e-16 5.465e-01 0.000 1.000
## CountryURT -2.343e-19 5.096e-01 0.000 1.000
## CountrySWA -2.345e-16 1.111e+00 0.000 1.000
## CountrySEN -1.728e-15 3.780e-01 0.000 1.000
## CountryZAI -6.743e-16 1.111e+00 0.000 1.000
## CountryMOZ -1.055e-15 8.058e-01 0.000 1.000
## ---
## Signif. codes: 0 '***' 0.001 '**' 0.01 '*' 0.05 '.' 0.1 ' ' 1
##
## (Dispersion parameter for gaussian family taken to be 1.168838)
##
## Null deviance: 209.26 on 155 degrees of freedom
## Residual deviance: 164.81 on 141 degrees of freedom
## AIC: 483.28
##
## Number of Fisher Scoring iterations: 2

data$Country <- relevel(data$Country, ref= "MOZ")
model1<-glm((Prev_TcTv) ~ Country,data=data, family=gaussian())
summary(model1)

##
## Call:
## glm(formula = (Prev_TcTv) ~ Country, family = gaussian(), data = data)
##
## Deviance Residuals:
## Min 1Q Median 3Q Max
## -2.0450 -0.2163 0.0000 0.0000 7.9550
##
## Coefficients:
## Estimate Std. Error t value Pr(>|t|)
## (Intercept) -1.042e-16 7.645e-01 0.000 1.0000
## CountryMLI -9.460e-16 8.058e-01 0.000 1.0000
## CountryKEN 2.045e+00 8.257e-01 2.477 0.0144 *
## CountryETH 1.538e-15 1.324e+00 0.000 1.0000
## CountryGUI -1.131e-14 8.138e-01 0.000 1.0000
## CountryGHA 2.303e-15 8.058e-01 0.000 1.0000
## CountryBKF 2.163e-01 7.820e-01 0.277 0.7825
## CountryZIM 1.313e-15 8.374e-01 0.000 1.0000
## CountryZAM 5.483e-16 1.324e+00 0.000 1.0000
## CountrySAF 2.363e-01 8.547e-01 0.276 0.7826
## CountryUGA -2.056e-16 9.045e-01 0.000 1.0000
## CountryURT 1.663e-15 8.827e-01 0.000 1.0000
## CountrySWA 9.890e-16 1.324e+00 0.000 1.0000
## CountrySEN 1.972e-15 8.138e-01 0.000 1.0000
## CountryZAI 5.439e-16 1.324e+00 0.000 1.0000
## ---
## Signif. codes: 0 '***' 0.001 '**' 0.01 '*' 0.05 '.' 0.1 ' ' 1
##
## (Dispersion parameter for gaussian family taken to be 1.168838)
##
## Null deviance: 209.26 on 155 degrees of freedom
## Residual deviance: 164.81 on 141 degrees of freedom
## AIC: 483.28
##
## Number of Fisher Scoring iterations: 2

data$Country <- relevel(data$Country, ref= "ZAI")
model1<-glm((Prev_TcTv) ~ Country,data=data, family=gaussian())
summary(model1)

##
## Call:
## glm(formula = (Prev_TcTv) ~ Country, family = gaussian(), data = data)
##
## Deviance Residuals:
## Min 1Q Median 3Q Max
## -2.0450 -0.2163 0.0000 0.0000 7.9550
##
## Coefficients:
## Estimate Std. Error t value Pr(>|t|)
## (Intercept) 5.182e-15 1.081e+00 0.000 1.0000
## CountryMOZ -7.572e-15 1.324e+00 0.000 1.0000
## CountryMLI -4.622e-15 1.111e+00 0.000 1.0000
## CountryKEN 2.045e+00 1.125e+00 1.817 0.0713 .
## CountryETH -3.152e-14 1.529e+00 0.000 1.0000
## CountryGUI -7.254e-15 1.117e+00 0.000 1.0000
## CountryGHA -4.887e-15 1.111e+00 0.000 1.0000
## CountryBKF 2.163e-01 1.094e+00 0.198 0.8435
## CountryZIM -5.336e-15 1.134e+00 0.000 1.0000
## CountryZAM 5.839e-16 1.529e+00 0.000 1.0000
## CountrySAF 2.362e-01 1.147e+00 0.206 0.8371
## CountryUGA -5.733e-15 1.184e+00 0.000 1.0000
## CountryURT -5.431e-15 1.168e+00 0.000 1.0000
## CountrySWA -7.103e-15 1.529e+00 0.000 1.0000
## CountrySEN -8.370e-15 1.117e+00 0.000 1.0000
## ---
## Signif. codes: 0 '***' 0.001 '**' 0.01 '*' 0.05 '.' 0.1 ' ' 1
##
## (Dispersion parameter for gaussian family taken to be 1.168838)
##
## Null deviance: 209.26 on 155 degrees of freedom
## Residual deviance: 164.81 on 141 degrees of freedom
## AIC: 483.28
##
## Number of Fisher Scoring iterations: 2

data$Country <- relevel(data$Country, ref= "SEN")
model1<-glm((Prev_TcTv) ~ Country,data=data, family=gaussian())
summary(model1)

##
## Call:
## glm(formula = (Prev_TcTv) ~ Country, family = gaussian(), data = data)
##
## Deviance Residuals:
## Min 1Q Median 3Q Max
## -2.0450 -0.2163 0.0000 0.0000 7.9550
##
## Coefficients:
## Estimate Std. Error t value Pr(>|t|)
## (Intercept) 1.988e-16 2.791e-01 0.000 1.000
## CountryZAI -4.742e-15 1.117e+00 0.000 1.000
## CountryMOZ -9.054e-16 8.138e-01 0.000 1.000
## CountryMLI 2.786e-17 3.780e-01 0.000 1.000
## CountryKEN 2.045e+00 4.187e-01 4.884 2.78e-06 ***
## CountryETH 1.336e-14 1.117e+00 0.000 1.000
## CountryGUI 1.923e-15 3.948e-01 0.000 1.000
## CountryGHA 2.425e-15 3.780e-01 0.000 1.000
## CountryBKF 2.163e-01 3.242e-01 0.667 0.506
## CountryZIM 2.211e-15 4.414e-01 0.000 1.000
## CountryZAM 3.167e-16 1.117e+00 0.000 1.000
## CountrySAF 2.363e-01 4.733e-01 0.499 0.618
## CountryUGA 2.782e-15 5.583e-01 0.000 1.000
## CountryURT 2.033e-15 5.222e-01 0.000 1.000
## CountrySWA 2.494e-15 1.117e+00 0.000 1.000
## ---
## Signif. codes: 0 '***' 0.001 '**' 0.01 '*' 0.05 '.' 0.1 ' ' 1
##
## (Dispersion parameter for gaussian family taken to be 1.168838)
##
## Null deviance: 209.26 on 155 degrees of freedom
## Residual deviance: 164.81 on 141 degrees of freedom
## AIC: 483.28
##
## Number of Fisher Scoring iterations: 2

data$Country <- relevel(data$Country, ref= "SWA")
model1<-glm((Prev_TcTv) ~ Country,data=data, family=gaussian())
summary(model1)

##
## Call:
## glm(formula = (Prev_TcTv) ~ Country, family = gaussian(), data = data)
##
## Deviance Residuals:
## Min 1Q Median 3Q Max
## -2.0450 -0.2163 0.0000 0.0000 7.9550
##
## Coefficients:
## Estimate Std. Error t value Pr(>|t|)
## (Intercept) 1.131e-14 1.081e+00 0.000 1.0000
## CountrySEN -1.177e-14 1.117e+00 0.000 1.0000
## CountryZAI -1.048e-14 1.529e+00 0.000 1.0000
## CountryMOZ -1.222e-14 1.324e+00 0.000 1.0000
## CountryMLI -1.773e-14 1.111e+00 0.000 1.0000
## CountryKEN 2.045e+00 1.125e+00 1.817 0.0713 .
## CountryETH -8.075e-15 1.529e+00 0.000 1.0000
## CountryGUI -8.970e-15 1.117e+00 0.000 1.0000
## CountryGHA -9.031e-15 1.111e+00 0.000 1.0000
## CountryBKF 2.163e-01 1.094e+00 0.198 0.8435
## CountryZIM -8.851e-15 1.134e+00 0.000 1.0000
## CountryZAM -6.940e-15 1.529e+00 0.000 1.0000
## CountrySAF 2.362e-01 1.147e+00 0.206 0.8371
## CountryUGA -8.357e-15 1.184e+00 0.000 1.0000
## CountryURT -8.574e-15 1.168e+00 0.000 1.0000
## ---
## Signif. codes: 0 '***' 0.001 '**' 0.01 '*' 0.05 '.' 0.1 ' ' 1
##
## (Dispersion parameter for gaussian family taken to be 1.168838)
##
## Null deviance: 209.26 on 155 degrees of freedom
## Residual deviance: 164.81 on 141 degrees of freedom
## AIC: 483.28
##
## Number of Fisher Scoring iterations: 2

data$Country <- relevel(data$Country, ref= "URT")
model1<-glm((Prev_TcTv) ~ Country,data=data, family=gaussian())
summary(model1)

##
## Call:
## glm(formula = (Prev_TcTv) ~ Country, family = gaussian(), data = data)
##
## Deviance Residuals:
## Min 1Q Median 3Q Max
## -2.0450 -0.2163 0.0000 0.0000 7.9550
##
## Coefficients:
## Estimate Std. Error t value Pr(>|t|)
## (Intercept) -8.621e-15 4.414e-01 0.000 1.000000
## CountrySWA 4.171e-15 1.168e+00 0.000 1.000000
## CountrySEN 9.215e-15 5.222e-01 0.000 1.000000
## CountryZAI 9.200e-15 1.168e+00 0.000 1.000000
## CountryMOZ 3.360e-15 8.827e-01 0.000 1.000000
## CountryMLI 7.216e-15 5.096e-01 0.000 1.000000
## CountryKEN 2.045e+00 5.406e-01 3.783 0.000228 ***
## CountryETH 5.840e-15 1.168e+00 0.000 1.000000
## CountryGUI 9.271e-15 5.222e-01 0.000 1.000000
## CountryGHA 9.174e-15 5.096e-01 0.000 1.000000
## CountryBKF 2.163e-01 4.712e-01 0.459 0.646913
## CountryZIM 8.736e-15 5.583e-01 0.000 1.000000
## CountryZAM 8.956e-15 1.168e+00 0.000 1.000000
## CountrySAF 2.363e-01 5.839e-01 0.405 0.686367
## CountryUGA 8.126e-15 6.547e-01 0.000 1.000000
## ---
## Signif. codes: 0 '***' 0.001 '**' 0.01 '*' 0.05 '.' 0.1 ' ' 1
##
## (Dispersion parameter for gaussian family taken to be 1.168838)
##
## Null deviance: 209.26 on 155 degrees of freedom
## Residual deviance: 164.81 on 141 degrees of freedom
## AIC: 483.28
##
## Number of Fisher Scoring iterations: 2

data$Country <- relevel(data$Country, ref= "UGA")
model1<-glm((Prev_TcTv) ~ Country,data=data, family=gaussian())
summary(model1)

##
## Call:
## glm(formula = (Prev_TcTv) ~ Country, family = gaussian(), data = data)
##
## Deviance Residuals:
## Min 1Q Median 3Q Max
## -2.0450 -0.2163 0.0000 0.0000 7.9550
##
## Coefficients:
## Estimate Std. Error t value Pr(>|t|)
## (Intercept) 2.629e-16 4.835e-01 0.000 1.000000
## CountryURT 1.053e-15 6.547e-01 0.000 1.000000
## CountrySWA -6.275e-16 1.184e+00 0.000 1.000000
## CountrySEN 9.718e-16 5.583e-01 0.000 1.000000
## CountryZAI 3.860e-14 1.184e+00 0.000 1.000000
## CountryMOZ 2.023e-15 9.045e-01 0.000 1.000000
## CountryMLI -5.353e-16 5.465e-01 0.000 1.000000
## CountryKEN 2.045e+00 5.755e-01 3.554 0.000517 ***
## CountryETH 1.094e-15 1.184e+00 0.000 1.000000
## CountryGUI -6.636e-16 5.583e-01 0.000 1.000000
## CountryGHA -1.739e-15 5.465e-01 0.000 1.000000
## CountryBKF 2.163e-01 5.108e-01 0.423 0.672659
## CountryZIM -7.119e-16 5.922e-01 0.000 1.000000
## CountryZAM -9.966e-15 1.184e+00 0.000 1.000000
## CountrySAF 2.362e-01 6.163e-01 0.383 0.702065
## ---
## Signif. codes: 0 '***' 0.001 '**' 0.01 '*' 0.05 '.' 0.1 ' ' 1
##
## (Dispersion parameter for gaussian family taken to be 1.168838)
##
## Null deviance: 209.26 on 155 degrees of freedom
## Residual deviance: 164.81 on 141 degrees of freedom
## AIC: 483.28
##
## Number of Fisher Scoring iterations: 2

data$Country <- relevel(data$Country, ref= "SAF")
model1<-glm((Prev_TcTv) ~ Country,data=data, family=gaussian())
summary(model1)

##
## Call:
## glm(formula = (Prev_TcTv) ~ Country, family = gaussian(), data = data)
##
## Deviance Residuals:
## Min 1Q Median 3Q Max
## -2.0450 -0.2163 0.0000 0.0000 7.9550
##
## Coefficients:
## Estimate Std. Error t value Pr(>|t|)
## (Intercept) 0.23625 0.38224 0.618 0.537524
## CountryUGA -0.23625 0.61634 -0.383 0.702065
## CountryURT -0.23625 0.58388 -0.405 0.686367
## CountrySWA -0.23625 1.14671 -0.206 0.837069
## CountrySEN -0.23625 0.47332 -0.499 0.618459
## CountryZAI -0.23625 1.14671 -0.206 0.837069
## CountryMOZ -0.23625 0.85471 -0.276 0.782637
## CountryMLI -0.23625 0.45939 -0.514 0.607870
## CountryKEN 1.80875 0.49347 3.665 0.000349 ***
## CountryETH -0.23625 1.14671 -0.206 0.837069
## CountryGUI -0.23625 0.47332 -0.499 0.618459
## CountryGHA -0.23625 0.45939 -0.514 0.607870
## CountryBKF -0.01997 0.41628 -0.048 0.961804
## CountryZIM -0.23625 0.51282 -0.461 0.645735
## CountryZAM -0.23625 1.14671 -0.206 0.837069
## ---
## Signif. codes: 0 '***' 0.001 '**' 0.01 '*' 0.05 '.' 0.1 ' ' 1
##
## (Dispersion parameter for gaussian family taken to be 1.168838)
##
## Null deviance: 209.26 on 155 degrees of freedom
## Residual deviance: 164.81 on 141 degrees of freedom
## AIC: 483.28
##
## Number of Fisher Scoring iterations: 2

data$Country <- relevel(data$Country, ref= "ZAM")
model1<-glm((Prev_TcTv) ~ Country,data=data, family=gaussian())
summary(model1)

##
## Call:
## glm(formula = (Prev_TcTv) ~ Country, family = gaussian(), data = data)
##
## Deviance Residuals:
## Min 1Q Median 3Q Max
## -2.0450 -0.2163 0.0000 0.0000 7.9550
##
## Coefficients:
## Estimate Std. Error t value Pr(>|t|)
## (Intercept) -3.848e-14 1.081e+00 0.000 1.0000
## CountrySAF 2.363e-01 1.147e+00 0.206 0.8371
## CountryUGA 3.913e-14 1.184e+00 0.000 1.0000
## CountryURT 3.890e-14 1.168e+00 0.000 1.0000
## CountrySWA 8.231e-15 1.529e+00 0.000 1.0000
## CountrySEN 3.836e-14 1.117e+00 0.000 1.0000
## CountryZAI 3.776e-14 1.529e+00 0.000 1.0000
## CountryMOZ 3.947e-14 1.324e+00 0.000 1.0000
## CountryMLI 3.869e-14 1.111e+00 0.000 1.0000
## CountryKEN 2.045e+00 1.125e+00 1.817 0.0713 .
## CountryETH 3.036e-14 1.529e+00 0.000 1.0000
## CountryGUI 3.866e-14 1.117e+00 0.000 1.0000
## CountryGHA 3.831e-14 1.111e+00 0.000 1.0000
## CountryBKF 2.163e-01 1.094e+00 0.198 0.8435
## CountryZIM 3.880e-14 1.134e+00 0.000 1.0000
## ---
## Signif. codes: 0 '***' 0.001 '**' 0.01 '*' 0.05 '.' 0.1 ' ' 1
##
## (Dispersion parameter for gaussian family taken to be 1.168838)
##
## Null deviance: 209.26 on 155 degrees of freedom
## Residual deviance: 164.81 on 141 degrees of freedom
## AIC: 483.28
##
## Number of Fisher Scoring iterations: 2

data$Country <- relevel(data$Country, ref= "ZIM")
model1<-glm((Prev_TcTv) ~ Country,data=data, family=gaussian())
summary(model1)

##
## Call:
## glm(formula = (Prev_TcTv) ~ Country, family = gaussian(), data = data)
##
## Deviance Residuals:
## Min 1Q Median 3Q Max
## -2.0450 -0.2163 0.0000 0.0000 7.9550
##
## Coefficients:
## Estimate Std. Error t value Pr(>|t|)
## (Intercept) 5.955e-15 3.419e-01 0.000 1.000
## CountryZAM -1.108e-14 1.134e+00 0.000 1.000
## CountrySAF 2.362e-01 5.128e-01 0.461 0.646
## CountryUGA -5.150e-15 5.922e-01 0.000 1.000
## CountryURT -2.163e-14 5.583e-01 0.000 1.000
## CountrySWA -1.589e-14 1.134e+00 0.000 1.000
## CountrySEN -5.951e-15 4.414e-01 0.000 1.000
## CountryZAI -1.082e-14 1.134e+00 0.000 1.000
## CountryMOZ -1.477e-14 8.374e-01 0.000 1.000
## CountryMLI -5.405e-15 4.264e-01 0.000 1.000
## CountryKEN 2.045e+00 4.629e-01 4.418 1.97e-05 ***
## CountryETH -6.762e-15 1.134e+00 0.000 1.000
## CountryGUI -5.751e-15 4.414e-01 0.000 1.000
## CountryGHA -5.573e-15 4.264e-01 0.000 1.000
## CountryBKF 2.163e-01 3.796e-01 0.570 0.570
## ---
## Signif. codes: 0 '***' 0.001 '**' 0.01 '*' 0.05 '.' 0.1 ' ' 1
##
## (Dispersion parameter for gaussian family taken to be 1.168838)
##
## Null deviance: 209.26 on 155 degrees of freedom
## Residual deviance: 164.81 on 141 degrees of freedom
## AIC: 483.28
##
## Number of Fisher Scoring iterations: 2

#======== Glm TcTz per country
data$Country <- relevel(data$Country, ref= "BKF")
model1<-glm((Prev_TcTz) ~ Country,data=data, family=gaussian())
summary(model1)

##
## Call:
## glm(formula = (Prev_TcTz) ~ Country, family = gaussian(), data = data)
##
## Deviance Residuals:
## Min 1Q Median 3Q Max
## -3.7894 -0.3586 0.0000 0.0000 18.4306
##
## Coefficients:
## Estimate Std. Error t value Pr(>|t|)
## (Intercept) 0.35860 0.40932 0.876 0.382
## CountryZIM -0.35860 0.94232 -0.381 0.704
## CountryZAM -0.35860 2.71512 -0.132 0.895
## CountrySAF -0.35860 1.03348 -0.347 0.729
## CountryUGA -0.35860 1.26823 -0.283 0.778
## CountryURT -0.12360 1.16973 -0.106 0.916
## CountrySWA -0.35860 2.71512 -0.132 0.895
## CountrySEN -0.35860 0.80488 -0.446 0.657
## CountryZAI -0.35860 2.71512 -0.132 0.895
## CountryMOZ -0.35860 1.94157 -0.185 0.854
## CountryMLI -0.35860 0.75351 -0.476 0.635
## CountryKEN -0.02444 0.87630 -0.028 0.978
## CountryETH -0.35860 2.71512 -0.132 0.895
## CountryGUI -0.35860 0.80488 -0.446 0.657
## CountryGHA 3.43084 0.75351 4.553 1.13e-05 ***
## ---
## Signif. codes: 0 '***' 0.001 '**' 0.01 '*' 0.05 '.' 0.1 ' ' 1
##
## (Dispersion parameter for gaussian family taken to be 7.204325)
##
## Null deviance: 1230.7 on 155 degrees of freedom
## Residual deviance: 1015.8 on 141 degrees of freedom
## AIC: 766.99
##
## Number of Fisher Scoring iterations: 2

data$Country <- relevel(data$Country, ref= "GHA")
model1<-glm((Prev_TcTz) ~ Country,data=data, family=gaussian())
summary(model1)

##
## Call:
## glm(formula = (Prev_TcTz) ~ Country, family = gaussian(), data = data)
##
## Deviance Residuals:
## Min 1Q Median 3Q Max
## -3.7894 -0.3586 0.0000 0.0000 18.4306
##
## Coefficients:
## Estimate Std. Error t value Pr(>|t|)
## (Intercept) 3.7894 0.6326 5.990 1.66e-08 ***
## CountryBKF -3.4308 0.7535 -4.553 1.13e-05 ***
## CountryZIM -3.7894 1.0586 -3.580 0.000472 ***
## CountryZAM -3.7894 2.7576 -1.374 0.171571
## CountrySAF -3.7894 1.1405 -3.323 0.001136 **
## CountryUGA -3.7894 1.3569 -2.793 0.005952 **
## CountryURT -3.5544 1.2653 -2.809 0.005672 **
## CountrySWA -3.7894 2.7576 -1.374 0.171571
## CountrySEN -3.7894 0.9384 -4.038 8.80e-05 ***
## CountryZAI -3.7894 2.7576 -1.374 0.171571
## CountryMOZ -3.7894 2.0006 -1.894 0.060253 .
## CountryMLI -3.7894 0.8947 -4.235 4.09e-05 ***
## CountryKEN -3.4553 1.0003 -3.454 0.000729 ***
## CountryETH -3.7894 2.7576 -1.374 0.171571
## CountryGUI -3.7894 0.9384 -4.038 8.80e-05 ***
## ---
## Signif. codes: 0 '***' 0.001 '**' 0.01 '*' 0.05 '.' 0.1 ' ' 1
##
## (Dispersion parameter for gaussian family taken to be 7.204325)
##
## Null deviance: 1230.7 on 155 degrees of freedom
## Residual deviance: 1015.8 on 141 degrees of freedom
## AIC: 766.99
##
## Number of Fisher Scoring iterations: 2

data$Country <- relevel(data$Country, ref= "GUI")
model1<-glm((Prev_TcTz) ~ Country,data=data, family=gaussian())
summary(model1)

##
## Call:
## glm(formula = (Prev_TcTz) ~ Country, family = gaussian(), data = data)
##
## Deviance Residuals:
## Min 1Q Median 3Q Max
## -3.7894 -0.3586 0.0000 0.0000 18.4306
##
## Coefficients:
## Estimate Std. Error t value Pr(>|t|)
## (Intercept) 1.209e-15 6.930e-01 0.000 1.000
## CountryGHA 3.789e+00 9.384e-01 4.038 8.8e-05 ***
## CountryBKF 3.586e-01 8.049e-01 0.446 0.657
## CountryZIM -2.041e-16 1.096e+00 0.000 1.000
## CountryZAM 5.004e-15 2.772e+00 0.000 1.000
## CountrySAF -4.096e-16 1.175e+00 0.000 1.000
## CountryUGA -5.811e-16 1.386e+00 0.000 1.000
## CountryURT 2.350e-01 1.297e+00 0.181 0.856
## CountrySWA -8.210e-16 2.772e+00 0.000 1.000
## CountrySEN -2.249e-16 9.801e-01 0.000 1.000
## CountryZAI 5.601e-16 2.772e+00 0.000 1.000
## CountryMOZ -5.156e-16 2.021e+00 0.000 1.000
## CountryMLI -3.186e-16 9.384e-01 0.000 1.000
## CountryKEN 3.342e-01 1.040e+00 0.321 0.748
## CountryETH -3.411e-15 2.772e+00 0.000 1.000
## ---
## Signif. codes: 0 '***' 0.001 '**' 0.01 '*' 0.05 '.' 0.1 ' ' 1
##
## (Dispersion parameter for gaussian family taken to be 7.204325)
##
## Null deviance: 1230.7 on 155 degrees of freedom
## Residual deviance: 1015.8 on 141 degrees of freedom
## AIC: 766.99
##
## Number of Fisher Scoring iterations: 2

data$Country <- relevel(data$Country, ref= "ETH")
model1<-glm((Prev_TcTz) ~ Country,data=data, family=gaussian())
summary(model1)

##
## Call:
## glm(formula = (Prev_TcTz) ~ Country, family = gaussian(), data = data)
##
## Deviance Residuals:
## Min 1Q Median 3Q Max
## -3.7894 -0.3586 0.0000 0.0000 18.4306
##
## Coefficients:
## Estimate Std. Error t value Pr(>|t|)
## (Intercept) -1.535e-16 2.684e+00 0.000 1.000
## CountryGUI 3.075e-15 2.772e+00 0.000 1.000
## CountryGHA 3.789e+00 2.758e+00 1.374 0.172
## CountryBKF 3.586e-01 2.715e+00 0.132 0.895
## CountryZIM 1.333e-15 2.815e+00 0.000 1.000
## CountryZAM 1.441e-15 3.796e+00 0.000 1.000
## CountrySAF -7.151e-16 2.847e+00 0.000 1.000
## CountryUGA -2.882e-16 2.940e+00 0.000 1.000
## CountryURT 2.350e-01 2.899e+00 0.081 0.936
## CountrySWA -1.979e-15 3.796e+00 0.000 1.000
## CountrySEN -8.517e-16 2.772e+00 0.000 1.000
## CountryZAI -1.156e-15 3.796e+00 0.000 1.000
## CountryMOZ 1.420e-15 3.287e+00 0.000 1.000
## CountryMLI -7.274e-16 2.758e+00 0.000 1.000
## CountryKEN 3.342e-01 2.794e+00 0.120 0.905
##
## (Dispersion parameter for gaussian family taken to be 7.204325)
##
## Null deviance: 1230.7 on 155 degrees of freedom
## Residual deviance: 1015.8 on 141 degrees of freedom
## AIC: 766.99
##
## Number of Fisher Scoring iterations: 2

data$Country <- relevel(data$Country, ref= "KEN")
model1<-glm((Prev_TcTz) ~ Country,data=data, family=gaussian())
summary(model1)

##
## Call:
## glm(formula = (Prev_TcTz) ~ Country, family = gaussian(), data = data)
##
## Deviance Residuals:
## Min 1Q Median 3Q Max
## -3.7894 -0.3586 0.0000 0.0000 18.4306
##
## Coefficients:
## Estimate Std. Error t value Pr(>|t|)
## (Intercept) 0.33417 0.77483 0.431 0.666925
## CountryETH -0.33417 2.79369 -0.120 0.904958
## CountryGUI -0.33417 1.03954 -0.321 0.748341
## CountryGHA 3.45528 1.00030 3.454 0.000729 ***
## CountryBKF 0.02444 0.87630 0.028 0.977791
## CountryZIM -0.33417 1.14926 -0.291 0.771656
## CountryZAM -0.33417 2.79369 -0.120 0.904958
## CountrySAF -0.33417 1.22511 -0.273 0.785434
## CountryUGA -0.33417 1.42871 -0.234 0.815407
## CountryURT -0.09917 1.34204 -0.074 0.941201
## CountrySWA -0.33417 2.79369 -0.120 0.904958
## CountrySEN -0.33417 1.03954 -0.321 0.748341
## CountryZAI -0.33417 2.79369 -0.120 0.904958
## CountryMOZ -0.33417 2.05001 -0.163 0.870746
## CountryMLI -0.33417 1.00030 -0.334 0.738826
## ---
## Signif. codes: 0 '***' 0.001 '**' 0.01 '*' 0.05 '.' 0.1 ' ' 1
##
## (Dispersion parameter for gaussian family taken to be 7.204325)
##
## Null deviance: 1230.7 on 155 degrees of freedom
## Residual deviance: 1015.8 on 141 degrees of freedom
## AIC: 766.99
##
## Number of Fisher Scoring iterations: 2

data$Country <- relevel(data$Country, ref= "MLI")
model1<-glm((Prev_TcTz) ~ Country,data=data, family=gaussian())
summary(model1)

##
## Call:
## glm(formula = (Prev_TcTz) ~ Country, family = gaussian(), data = data)
##
## Deviance Residuals:
## Min 1Q Median 3Q Max
## -3.7894 -0.3586 0.0000 0.0000 18.4306
##
## Coefficients:
## Estimate Std. Error t value Pr(>|t|)
## (Intercept) 2.089e-16 6.326e-01 0.000 1.000
## CountryKEN 3.342e-01 1.000e+00 0.334 0.739
## CountryETH 1.768e-15 2.758e+00 0.000 1.000
## CountryGUI -6.666e-16 9.384e-01 0.000 1.000
## CountryGHA 3.789e+00 8.947e-01 4.235 4.09e-05 ***
## CountryBKF 3.586e-01 7.535e-01 0.476 0.635
## CountryZIM -5.254e-16 1.059e+00 0.000 1.000
## CountryZAM -6.858e-16 2.758e+00 0.000 1.000
## CountrySAF -1.921e-16 1.141e+00 0.000 1.000
## CountryUGA -8.262e-16 1.357e+00 0.000 1.000
## CountryURT 2.350e-01 1.265e+00 0.186 0.853
## CountrySWA -1.292e-15 2.758e+00 0.000 1.000
## CountrySEN -9.176e-16 9.384e-01 0.000 1.000
## CountryZAI -1.251e-16 2.758e+00 0.000 1.000
## CountryMOZ -1.138e-16 2.001e+00 0.000 1.000
## ---
## Signif. codes: 0 '***' 0.001 '**' 0.01 '*' 0.05 '.' 0.1 ' ' 1
##
## (Dispersion parameter for gaussian family taken to be 7.204325)
##
## Null deviance: 1230.7 on 155 degrees of freedom
## Residual deviance: 1015.8 on 141 degrees of freedom
## AIC: 766.99
##
## Number of Fisher Scoring iterations: 2

data$Country <- relevel(data$Country, ref= "MOZ")
model1<-glm((Prev_TcTz) ~ Country,data=data, family=gaussian())
summary(model1)

##
## Call:
## glm(formula = (Prev_TcTz) ~ Country, family = gaussian(), data = data)
##
## Deviance Residuals:
## Min 1Q Median 3Q Max
## -3.7894 -0.3586 0.0000 0.0000 18.4306
##
## Coefficients:
## Estimate Std. Error t value Pr(>|t|)
## (Intercept) 2.451e-15 1.898e+00 0.000 1.0000
## CountryMLI -3.412e-15 2.001e+00 0.000 1.0000
## CountryKEN 3.342e-01 2.050e+00 0.163 0.8707
## CountryETH -2.103e-15 3.287e+00 0.000 1.0000
## CountryGUI -4.523e-15 2.021e+00 0.000 1.0000
## CountryGHA 3.789e+00 2.001e+00 1.894 0.0603 .
## CountryBKF 3.586e-01 1.942e+00 0.185 0.8537
## CountryZIM -2.962e-15 2.079e+00 0.000 1.0000
## CountryZAM -4.012e-15 3.287e+00 0.000 1.0000
## CountrySAF -3.407e-15 2.122e+00 0.000 1.0000
## CountryUGA -2.651e-15 2.246e+00 0.000 1.0000
## CountryURT 2.350e-01 2.192e+00 0.107 0.9148
## CountrySWA -7.183e-15 3.287e+00 0.000 1.0000
## CountrySEN -3.305e-15 2.021e+00 0.000 1.0000
## CountryZAI -1.700e-15 3.287e+00 0.000 1.0000
## ---
## Signif. codes: 0 '***' 0.001 '**' 0.01 '*' 0.05 '.' 0.1 ' ' 1
##
## (Dispersion parameter for gaussian family taken to be 7.204325)
##
## Null deviance: 1230.7 on 155 degrees of freedom
## Residual deviance: 1015.8 on 141 degrees of freedom
## AIC: 766.99
##
## Number of Fisher Scoring iterations: 2

data$Country <- relevel(data$Country, ref= "ZAI")
model1<-glm((Prev_TcTz) ~ Country,data=data, family=gaussian())
summary(model1)

##
## Call:
## glm(formula = (Prev_TcTz) ~ Country, family = gaussian(), data = data)
##
## Deviance Residuals:
## Min 1Q Median 3Q Max
## -3.7894 -0.3586 0.0000 0.0000 18.4306
##
## Coefficients:
## Estimate Std. Error t value Pr(>|t|)
## (Intercept) 2.585e-15 2.684e+00 0.000 1.000
## CountryMOZ 1.695e-16 3.287e+00 0.000 1.000
## CountryMLI -1.507e-15 2.758e+00 0.000 1.000
## CountryKEN 3.342e-01 2.794e+00 0.120 0.905
## CountryETH 8.687e-15 3.796e+00 0.000 1.000
## CountryGUI -9.789e-16 2.772e+00 0.000 1.000
## CountryGHA 3.789e+00 2.758e+00 1.374 0.172
## CountryBKF 3.586e-01 2.715e+00 0.132 0.895
## CountryZIM -3.095e-15 2.815e+00 0.000 1.000
## CountryZAM -4.302e-15 3.796e+00 0.000 1.000
## CountrySAF -4.216e-15 2.847e+00 0.000 1.000
## CountryUGA -4.170e-15 2.940e+00 0.000 1.000
## CountryURT 2.350e-01 2.899e+00 0.081 0.936
## CountrySWA -3.049e-15 3.796e+00 0.000 1.000
## CountrySEN -3.741e-15 2.772e+00 0.000 1.000
##
## (Dispersion parameter for gaussian family taken to be 7.204325)
##
## Null deviance: 1230.7 on 155 degrees of freedom
## Residual deviance: 1015.8 on 141 degrees of freedom
## AIC: 766.99
##
## Number of Fisher Scoring iterations: 2

data$Country <- relevel(data$Country, ref= "SEN")
model1<-glm((Prev_TcTz) ~ Country,data=data, family=gaussian())
summary(model1)

##
## Call:
## glm(formula = (Prev_TcTz) ~ Country, family = gaussian(), data = data)
##
## Deviance Residuals:
## Min 1Q Median 3Q Max
## -3.7894 -0.3586 0.0000 0.0000 18.4306
##
## Coefficients:
## Estimate Std. Error t value Pr(>|t|)
## (Intercept) -1.874e-15 6.930e-01 0.000 1.000
## CountryZAI 9.262e-16 2.772e+00 0.000 1.000
## CountryMOZ 3.636e-15 2.021e+00 0.000 1.000
## CountryMLI 1.727e-15 9.384e-01 0.000 1.000
## CountryKEN 3.342e-01 1.040e+00 0.321 0.748
## CountryETH 2.669e-15 2.772e+00 0.000 1.000
## CountryGUI 8.571e-16 9.801e-01 0.000 1.000
## CountryGHA 3.789e+00 9.384e-01 4.038 8.8e-05 ***
## CountryBKF 3.586e-01 8.049e-01 0.446 0.657
## CountryZIM 1.289e-15 1.096e+00 0.000 1.000
## CountryZAM -3.120e-14 2.772e+00 0.000 1.000
## CountrySAF 1.648e-15 1.175e+00 0.000 1.000
## CountryUGA 1.238e-15 1.386e+00 0.000 1.000
## CountryURT 2.350e-01 1.297e+00 0.181 0.856
## CountrySWA 9.110e-16 2.772e+00 0.000 1.000
## ---
## Signif. codes: 0 '***' 0.001 '**' 0.01 '*' 0.05 '.' 0.1 ' ' 1
##
## (Dispersion parameter for gaussian family taken to be 7.204325)
##
## Null deviance: 1230.7 on 155 degrees of freedom
## Residual deviance: 1015.8 on 141 degrees of freedom
## AIC: 766.99
##
## Number of Fisher Scoring iterations: 2

data$Country <- relevel(data$Country, ref= "SWA")
model1<-glm((Prev_TcTz) ~ Country,data=data, family=gaussian())
summary(model1)

##
## Call:
## glm(formula = (Prev_TcTz) ~ Country, family = gaussian(), data = data)
##
## Deviance Residuals:
## Min 1Q Median 3Q Max
## -3.7894 -0.3586 0.0000 0.0000 18.4306
##
## Coefficients:
## Estimate Std. Error t value Pr(>|t|)
## (Intercept) -7.527e-15 2.684e+00 0.000 1.000
## CountrySEN 1.102e-14 2.772e+00 0.000 1.000
## CountryZAI 6.513e-15 3.796e+00 0.000 1.000
## CountryMOZ 1.088e-14 3.287e+00 0.000 1.000
## CountryMLI 5.711e-15 2.758e+00 0.000 1.000
## CountryKEN 3.342e-01 2.794e+00 0.120 0.905
## CountryETH 1.209e-14 3.796e+00 0.000 1.000
## CountryGUI 8.285e-15 2.772e+00 0.000 1.000
## CountryGHA 3.789e+00 2.758e+00 1.374 0.172
## CountryBKF 3.586e-01 2.715e+00 0.132 0.895
## CountryZIM 8.210e-15 2.815e+00 0.000 1.000
## CountryZAM 5.515e-15 3.796e+00 0.000 1.000
## CountrySAF 9.175e-15 2.847e+00 0.000 1.000
## CountryUGA 6.696e-15 2.940e+00 0.000 1.000
## CountryURT 2.350e-01 2.899e+00 0.081 0.936
##
## (Dispersion parameter for gaussian family taken to be 7.204325)
##
## Null deviance: 1230.7 on 155 degrees of freedom
## Residual deviance: 1015.8 on 141 degrees of freedom
## AIC: 766.99
##
## Number of Fisher Scoring iterations: 2

data$Country <- relevel(data$Country, ref= "URT")
model1<-glm((Prev_TcTz) ~ Country,data=data, family=gaussian())
summary(model1)

##
## Call:
## glm(formula = (Prev_TcTz) ~ Country, family = gaussian(), data = data)
##
## Deviance Residuals:
## Min 1Q Median 3Q Max
## -3.7894 -0.3586 0.0000 0.0000 18.4306
##
## Coefficients:
## Estimate Std. Error t value Pr(>|t|)
## (Intercept) 0.23500 1.09577 0.214 0.83050
## CountrySWA -0.23500 2.89915 -0.081 0.93551
## CountrySEN -0.23500 1.29654 -0.181 0.85643
## CountryZAI -0.23500 2.89915 -0.081 0.93551
## CountryMOZ -0.23500 2.19155 -0.107 0.91476
## CountryMLI -0.23500 1.26529 -0.186 0.85292
## CountryKEN 0.09917 1.34204 0.074 0.94120
## CountryETH -0.23500 2.89915 -0.081 0.93551
## CountryGUI -0.23500 1.29654 -0.181 0.85643
## CountryGHA 3.55444 1.26529 2.809 0.00567 **
## CountryBKF 0.12360 1.16973 0.106 0.91599
## CountryZIM -0.23500 1.38606 -0.170 0.86561
## CountryZAM -0.23500 2.89915 -0.081 0.93551
## CountrySAF -0.23500 1.44957 -0.162 0.87145
## CountryUGA -0.23500 1.62530 -0.145 0.88524
## ---
## Signif. codes: 0 '***' 0.001 '**' 0.01 '*' 0.05 '.' 0.1 ' ' 1
##
## (Dispersion parameter for gaussian family taken to be 7.204325)
##
## Null deviance: 1230.7 on 155 degrees of freedom
## Residual deviance: 1015.8 on 141 degrees of freedom
## AIC: 766.99
##
## Number of Fisher Scoring iterations: 2

data$Country <- relevel(data$Country, ref= "UGA")
model1<-glm((Prev_TcTz) ~ Country,data=data, family=gaussian())
summary(model1)

##
## Call:
## glm(formula = (Prev_TcTz) ~ Country, family = gaussian(), data = data)
##
## Deviance Residuals:
## Min 1Q Median 3Q Max
## -3.7894 -0.3586 0.0000 0.0000 18.4306
##
## Coefficients:
## Estimate Std. Error t value Pr(>|t|)
## (Intercept) -1.222e-15 1.200e+00 0.000 1.00000
## CountryURT 2.350e-01 1.625e+00 0.145 0.88524
## CountrySWA 1.962e-15 2.940e+00 0.000 1.00000
## CountrySEN 4.112e-15 1.386e+00 0.000 1.00000
## CountryZAI -4.143e-15 2.940e+00 0.000 1.00000
## CountryMOZ -8.805e-17 2.246e+00 0.000 1.00000
## CountryMLI -3.170e-16 1.357e+00 0.000 1.00000
## CountryKEN 3.342e-01 1.429e+00 0.234 0.81541
## CountryETH -1.171e-15 2.940e+00 0.000 1.00000
## CountryGUI 1.119e-15 1.386e+00 0.000 1.00000
## CountryGHA 3.789e+00 1.357e+00 2.793 0.00595 **
## CountryBKF 3.586e-01 1.268e+00 0.283 0.77778
## CountryZIM 1.815e-15 1.470e+00 0.000 1.00000
## CountryZAM 9.729e-16 2.940e+00 0.000 1.00000
## CountrySAF 1.768e-15 1.530e+00 0.000 1.00000
## ---
## Signif. codes: 0 '***' 0.001 '**' 0.01 '*' 0.05 '.' 0.1 ' ' 1
##
## (Dispersion parameter for gaussian family taken to be 7.204325)
##
## Null deviance: 1230.7 on 155 degrees of freedom
## Residual deviance: 1015.8 on 141 degrees of freedom
## AIC: 766.99
##
## Number of Fisher Scoring iterations: 2

data$Country <- relevel(data$Country, ref= "SAF")
model1<-glm((Prev_TcTz) ~ Country,data=data, family=gaussian())
summary(model1)

##
## Call:
## glm(formula = (Prev_TcTz) ~ Country, family = gaussian(), data = data)
##
## Deviance Residuals:
## Min 1Q Median 3Q Max
## -3.7894 -0.3586 0.0000 0.0000 18.4306
##
## Coefficients:
## Estimate Std. Error t value Pr(>|t|)
## (Intercept) 1.492e-15 9.490e-01 0.000 1.00000
## CountryUGA -2.430e-15 1.530e+00 0.000 1.00000
## CountryURT 2.350e-01 1.450e+00 0.162 0.87145
## CountrySWA -7.619e-16 2.847e+00 0.000 1.00000
## CountrySEN -5.865e-16 1.175e+00 0.000 1.00000
## CountryZAI -4.824e-15 2.847e+00 0.000 1.00000
## CountryMOZ 8.483e-17 2.122e+00 0.000 1.00000
## CountryMLI -1.449e-15 1.141e+00 0.000 1.00000
## CountryKEN 3.342e-01 1.225e+00 0.273 0.78543
## CountryETH 5.042e-15 2.847e+00 0.000 1.00000
## CountryGUI 1.249e-16 1.175e+00 0.000 1.00000
## CountryGHA 3.789e+00 1.141e+00 3.323 0.00114 **
## CountryBKF 3.586e-01 1.033e+00 0.347 0.72912
## CountryZIM -1.675e-15 1.273e+00 0.000 1.00000
## CountryZAM -8.464e-16 2.847e+00 0.000 1.00000
## ---
## Signif. codes: 0 '***' 0.001 '**' 0.01 '*' 0.05 '.' 0.1 ' ' 1
##
## (Dispersion parameter for gaussian family taken to be 7.204325)
##
## Null deviance: 1230.7 on 155 degrees of freedom
## Residual deviance: 1015.8 on 141 degrees of freedom
## AIC: 766.99
##
## Number of Fisher Scoring iterations: 2

data$Country <- relevel(data$Country, ref= "ZAM")
model1<-glm((Prev_TcTz) ~ Country,data=data, family=gaussian())
summary(model1)

##
## Call:
## glm(formula = (Prev_TcTz) ~ Country, family = gaussian(), data = data)
##
## Deviance Residuals:
## Min 1Q Median 3Q Max
## -3.7894 -0.3586 0.0000 0.0000 18.4306
##
## Coefficients:
## Estimate Std. Error t value Pr(>|t|)
## (Intercept) -4.631e-15 2.684e+00 0.000 1.000
## CountrySAF 3.665e-15 2.847e+00 0.000 1.000
## CountryUGA 4.773e-15 2.940e+00 0.000 1.000
## CountryURT 2.350e-01 2.899e+00 0.081 0.936
## CountrySWA -3.759e-15 3.796e+00 0.000 1.000
## CountrySEN 3.169e-15 2.772e+00 0.000 1.000
## CountryZAI 6.544e-15 3.796e+00 0.000 1.000
## CountryMOZ 2.690e-15 3.287e+00 0.000 1.000
## CountryMLI 5.472e-15 2.758e+00 0.000 1.000
## CountryKEN 3.342e-01 2.794e+00 0.120 0.905
## CountryETH 8.794e-15 3.796e+00 0.000 1.000
## CountryGUI 5.672e-15 2.772e+00 0.000 1.000
## CountryGHA 3.789e+00 2.758e+00 1.374 0.172
## CountryBKF 3.586e-01 2.715e+00 0.132 0.895
## CountryZIM 4.105e-15 2.815e+00 0.000 1.000
##
## (Dispersion parameter for gaussian family taken to be 7.204325)
##
## Null deviance: 1230.7 on 155 degrees of freedom
## Residual deviance: 1015.8 on 141 degrees of freedom
## AIC: 766.99
##
## Number of Fisher Scoring iterations: 2

data$Country <- relevel(data$Country, ref= "ZIM")
model1<-glm((Prev_TcTz) ~ Country,data=data, family=gaussian())
summary(model1)

##
## Call:
## glm(formula = (Prev_TcTz) ~ Country, family = gaussian(), data = data)
##
## Deviance Residuals:
## Min 1Q Median 3Q Max
## -3.7894 -0.3586 0.0000 0.0000 18.4306
##
## Coefficients:
## Estimate Std. Error t value Pr(>|t|)
## (Intercept) 2.194e-15 8.488e-01 0.000 1.000000
## CountryZAM -5.394e-15 2.815e+00 0.000 1.000000
## CountrySAF -3.226e-16 1.273e+00 0.000 1.000000
## CountryUGA -2.595e-15 1.470e+00 0.000 1.000000
## CountryURT 2.350e-01 1.386e+00 0.170 0.865610
## CountrySWA -5.910e-15 2.815e+00 0.000 1.000000
## CountrySEN -3.719e-15 1.096e+00 0.000 1.000000
## CountryZAI 1.144e-16 2.815e+00 0.000 1.000000
## CountryMOZ 4.144e-15 2.079e+00 0.000 1.000000
## CountryMLI -5.334e-16 1.059e+00 0.000 1.000000
## CountryKEN 3.342e-01 1.149e+00 0.291 0.771656
## CountryETH -2.672e-15 2.815e+00 0.000 1.000000
## CountryGUI -3.155e-15 1.096e+00 0.000 1.000000
## CountryGHA 3.789e+00 1.059e+00 3.580 0.000472 ***
## CountryBKF 3.586e-01 9.423e-01 0.381 0.704107
## ---
## Signif. codes: 0 '***' 0.001 '**' 0.01 '*' 0.05 '.' 0.1 ' ' 1
##
## (Dispersion parameter for gaussian family taken to be 7.204325)
##
## Null deviance: 1230.7 on 155 degrees of freedom
## Residual deviance: 1015.8 on 141 degrees of freedom
## AIC: 766.99
##
## Number of Fisher Scoring iterations: 2

#======== Glm TcTsg per country
data$Country <- relevel(data$Country, ref= "BKF")
model1<-glm((Prev_TcTsg) ~ Country,data=data, family=gaussian())
summary(model1)

##
## Call:
## glm(formula = (Prev_TcTsg) ~ Country, family = gaussian(), data = data)
##
## Deviance Residuals:
## Min 1Q Median 3Q Max
## -4.6633 -0.1012 0.0000 0.0000 16.4667
##
## Coefficients:
## Estimate Std. Error t value Pr(>|t|)
## (Intercept) 0.1012 0.3926 0.258 0.79701
## CountryZIM 1.5788 0.9037 1.747 0.08281 .
## CountryZAM 7.4288 2.6039 2.853 0.00498 **
## CountrySAF 0.3538 0.9912 0.357 0.72163
## CountryUGA 0.1108 1.2163 0.091 0.92752
## CountryURT 4.5622 1.1218 4.067 7.89e-05 ***
## CountrySWA -0.1012 2.6039 -0.039 0.96906
## CountrySEN -0.1012 0.7719 -0.131 0.89592
## CountryZAI -0.1012 2.6039 -0.039 0.96906
## CountryMOZ -0.1012 1.8621 -0.054 0.95675
## CountryMLI -0.1012 0.7227 -0.140 0.88887
## CountryKEN 4.2872 0.8404 5.101 1.07e-06 ***
## CountryETH 0.5488 2.6039 0.211 0.83337
## CountryGUI -0.1012 0.7719 -0.131 0.89592
## CountryGHA -0.1012 0.7227 -0.140 0.88887
## ---
## Signif. codes: 0 '***' 0.001 '**' 0.01 '*' 0.05 '.' 0.1 ' ' 1
##
## (Dispersion parameter for gaussian family taken to be 6.626342)
##
## Null deviance: 1299.26 on 155 degrees of freedom
## Residual deviance: 934.31 on 141 degrees of freedom
## AIC: 753.94
##
## Number of Fisher Scoring iterations: 2

data$Country <- relevel(data$Country, ref= "GHA")
model1<-glm((Prev_TcTsg) ~ Country,data=data, family=gaussian())
summary(model1)

##
## Call:
## glm(formula = (Prev_TcTsg) ~ Country, family = gaussian(), data = data)
##
## Deviance Residuals:
## Min 1Q Median 3Q Max
## -4.6633 -0.1012 0.0000 0.0000 16.4667
##
## Coefficients:
## Estimate Std. Error t value Pr(>|t|)
## (Intercept) -5.756e-15 6.067e-01 0.000 1.000000
## CountryBKF 1.012e-01 7.227e-01 0.140 0.888870
## CountryZIM 1.680e+00 1.015e+00 1.655 0.100202
## CountryZAM 7.530e+00 2.645e+00 2.847 0.005070 **
## CountrySAF 4.550e-01 1.094e+00 0.416 0.678059
## CountryUGA 2.120e-01 1.301e+00 0.163 0.870820
## CountryURT 4.663e+00 1.213e+00 3.843 0.000183 ***
## CountrySWA 5.657e-15 2.645e+00 0.000 1.000000
## CountrySEN 1.014e-14 8.999e-01 0.000 1.000000
## CountryZAI 7.705e-15 2.645e+00 0.000 1.000000
## CountryMOZ 1.346e-14 1.919e+00 0.000 1.000000
## CountryMLI 4.198e-15 8.581e-01 0.000 1.000000
## CountryKEN 4.388e+00 9.593e-01 4.574 1.04e-05 ***
## CountryETH 6.500e-01 2.645e+00 0.246 0.806215
## CountryGUI 3.901e-15 8.999e-01 0.000 1.000000
## ---
## Signif. codes: 0 '***' 0.001 '**' 0.01 '*' 0.05 '.' 0.1 ' ' 1
##
## (Dispersion parameter for gaussian family taken to be 6.626342)
##
## Null deviance: 1299.26 on 155 degrees of freedom
## Residual deviance: 934.31 on 141 degrees of freedom
## AIC: 753.94
##
## Number of Fisher Scoring iterations: 2

data$Country <- relevel(data$Country, ref= "GUI")
model1<-glm((Prev_TcTsg) ~ Country,data=data, family=gaussian())
summary(model1)

##
## Call:
## glm(formula = (Prev_TcTsg) ~ Country, family = gaussian(), data = data)
##
## Deviance Residuals:
## Min 1Q Median 3Q Max
## -4.6633 -0.1012 0.0000 0.0000 16.4667
##
## Coefficients:
## Estimate Std. Error t value Pr(>|t|)
## (Intercept) 5.850e-15 6.646e-01 0.000 1.000000
## CountryGHA -5.634e-15 8.999e-01 0.000 1.000000
## CountryBKF 1.012e-01 7.719e-01 0.131 0.895920
## CountryZIM 1.680e+00 1.051e+00 1.599 0.112141
## CountryZAM 7.530e+00 2.659e+00 2.832 0.005298 **
## CountrySAF 4.550e-01 1.127e+00 0.404 0.687016
## CountryUGA 2.120e-01 1.329e+00 0.159 0.873517
## CountryURT 4.663e+00 1.243e+00 3.750 0.000257 ***
## CountrySWA -3.896e-15 2.659e+00 0.000 1.000000
## CountrySEN -4.078e-15 9.400e-01 0.000 1.000000
## CountryZAI -7.877e-15 2.659e+00 0.000 1.000000
## CountryMOZ -5.226e-15 1.938e+00 0.000 1.000000
## CountryMLI -1.878e-15 8.999e-01 0.000 1.000000
## CountryKEN 4.388e+00 9.970e-01 4.402 2.11e-05 ***
## CountryETH 6.500e-01 2.659e+00 0.244 0.807206
## ---
## Signif. codes: 0 '***' 0.001 '**' 0.01 '*' 0.05 '.' 0.1 ' ' 1
##
## (Dispersion parameter for gaussian family taken to be 6.626342)
##
## Null deviance: 1299.26 on 155 degrees of freedom
## Residual deviance: 934.31 on 141 degrees of freedom
## AIC: 753.94
##
## Number of Fisher Scoring iterations: 2

data$Country <- relevel(data$Country, ref= "ETH")
model1<-glm((Prev_TcTsg) ~ Country,data=data, family=gaussian())
summary(model1)

##
## Call:
## glm(formula = (Prev_TcTsg) ~ Country, family = gaussian(), data = data)
##
## Deviance Residuals:
## Min 1Q Median 3Q Max
## -4.6633 -0.1012 0.0000 0.0000 16.4667
##
## Coefficients:
## Estimate Std. Error t value Pr(>|t|)
## (Intercept) 0.6500 2.5742 0.253 0.8010
## CountryGUI -0.6500 2.6586 -0.244 0.8072
## CountryGHA -0.6500 2.6447 -0.246 0.8062
## CountryBKF -0.5488 2.6039 -0.211 0.8334
## CountryZIM 1.0300 2.6998 0.382 0.7034
## CountryZAM 6.8800 3.6404 1.890 0.0608 .
## CountrySAF -0.1950 2.7303 -0.071 0.9432
## CountryUGA -0.4380 2.8199 -0.155 0.8768
## CountryURT 4.0133 2.7804 1.443 0.1511
## CountrySWA -0.6500 3.6404 -0.179 0.8585
## CountrySEN -0.6500 2.6586 -0.244 0.8072
## CountryZAI -0.6500 3.6404 -0.179 0.8585
## CountryMOZ -0.6500 3.1527 -0.206 0.8370
## CountryMLI -0.6500 2.6447 -0.246 0.8062
## CountryKEN 3.7383 2.6793 1.395 0.1651
## ---
## Signif. codes: 0 '***' 0.001 '**' 0.01 '*' 0.05 '.' 0.1 ' ' 1
##
## (Dispersion parameter for gaussian family taken to be 6.626342)
##
## Null deviance: 1299.26 on 155 degrees of freedom
## Residual deviance: 934.31 on 141 degrees of freedom
## AIC: 753.94
##
## Number of Fisher Scoring iterations: 2

data$Country <- relevel(data$Country, ref= "KEN")
model1<-glm((Prev_TcTsg) ~ Country,data=data, family=gaussian())
summary(model1)

##
## Call:
## glm(formula = (Prev_TcTsg) ~ Country, family = gaussian(), data = data)
##
## Deviance Residuals:
## Min 1Q Median 3Q Max
## -4.6633 -0.1012 0.0000 0.0000 16.4667
##
## Coefficients:
## Estimate Std. Error t value Pr(>|t|)
## (Intercept) 4.3883 0.7431 5.905 2.51e-08 ***
## CountryETH -3.7383 2.6793 -1.395 0.16513
## CountryGUI -4.3883 0.9970 -4.402 2.11e-05 ***
## CountryGHA -4.3883 0.9593 -4.574 1.04e-05 ***
## CountryBKF -4.2872 0.8404 -5.101 1.07e-06 ***
## CountryZIM -2.7083 1.1022 -2.457 0.01522 *
## CountryZAM 3.1417 2.6793 1.173 0.24294
## CountrySAF -3.9333 1.1749 -3.348 0.00105 **
## CountryUGA -4.1763 1.3702 -3.048 0.00275 **
## CountryURT 0.2750 1.2871 0.214 0.83112
## CountrySWA -4.3883 2.6793 -1.638 0.10368
## CountrySEN -4.3883 0.9970 -4.402 2.11e-05 ***
## CountryZAI -4.3883 2.6793 -1.638 0.10368
## CountryMOZ -4.3883 1.9661 -2.232 0.02719 *
## CountryMLI -4.3883 0.9593 -4.574 1.04e-05 ***
## ---
## Signif. codes: 0 '***' 0.001 '**' 0.01 '*' 0.05 '.' 0.1 ' ' 1
##
## (Dispersion parameter for gaussian family taken to be 6.626342)
##
## Null deviance: 1299.26 on 155 degrees of freedom
## Residual deviance: 934.31 on 141 degrees of freedom
## AIC: 753.94
##
## Number of Fisher Scoring iterations: 2

data$Country <- relevel(data$Country, ref= "MLI")
model1<-glm((Prev_TcTz) ~ Country,data=data, family=gaussian())
summary(model1)

##
## Call:
## glm(formula = (Prev_TcTz) ~ Country, family = gaussian(), data = data)
##
## Deviance Residuals:
## Min 1Q Median 3Q Max
## -3.7894 -0.3586 0.0000 0.0000 18.4306
##
## Coefficients:
## Estimate Std. Error t value Pr(>|t|)
## (Intercept) 2.089e-16 6.326e-01 0.000 1.000
## CountryKEN 3.342e-01 1.000e+00 0.334 0.739
## CountryETH 1.768e-15 2.758e+00 0.000 1.000
## CountryGUI -6.666e-16 9.384e-01 0.000 1.000
## CountryGHA 3.789e+00 8.947e-01 4.235 4.09e-05 ***
## CountryBKF 3.586e-01 7.535e-01 0.476 0.635
## CountryZIM -5.254e-16 1.059e+00 0.000 1.000
## CountryZAM -6.858e-16 2.758e+00 0.000 1.000
## CountrySAF -1.921e-16 1.141e+00 0.000 1.000
## CountryUGA -8.262e-16 1.357e+00 0.000 1.000
## CountryURT 2.350e-01 1.265e+00 0.186 0.853
## CountrySWA -1.292e-15 2.758e+00 0.000 1.000
## CountrySEN -9.176e-16 9.384e-01 0.000 1.000
## CountryZAI -1.251e-16 2.758e+00 0.000 1.000
## CountryMOZ -1.138e-16 2.001e+00 0.000 1.000
## ---
## Signif. codes: 0 '***' 0.001 '**' 0.01 '*' 0.05 '.' 0.1 ' ' 1
##
## (Dispersion parameter for gaussian family taken to be 7.204325)
##
## Null deviance: 1230.7 on 155 degrees of freedom
## Residual deviance: 1015.8 on 141 degrees of freedom
## AIC: 766.99
##
## Number of Fisher Scoring iterations: 2

data$Country <- relevel(data$Country, ref= "MOZ")
model1<-glm((Prev_TcTsg) ~ Country,data=data, family=gaussian())
summary(model1)

##
## Call:
## glm(formula = (Prev_TcTsg) ~ Country, family = gaussian(), data = data)
##
## Deviance Residuals:
## Min 1Q Median 3Q Max
## -4.6633 -0.1012 0.0000 0.0000 16.4667
##
## Coefficients:
## Estimate Std. Error t value Pr(>|t|)
## (Intercept) -1.047e-14 1.820e+00 0.000 1.0000
## CountryMLI 1.063e-14 1.919e+00 0.000 1.0000
## CountryKEN 4.388e+00 1.966e+00 2.232 0.0272 *
## CountryETH 6.500e-01 3.153e+00 0.206 0.8370
## CountryGUI -5.284e-15 1.938e+00 0.000 1.0000
## CountryGHA 1.360e-14 1.919e+00 0.000 1.0000
## CountryBKF 1.012e-01 1.862e+00 0.054 0.9568
## CountryZIM 1.680e+00 1.994e+00 0.843 0.4009
## CountryZAM 7.530e+00 3.153e+00 2.388 0.0182 *
## CountrySAF 4.550e-01 2.035e+00 0.224 0.8234
## CountryUGA 2.120e-01 2.154e+00 0.098 0.9217
## CountryURT 4.663e+00 2.102e+00 2.219 0.0281 *
## CountrySWA 1.073e-14 3.153e+00 0.000 1.0000
## CountrySEN 1.232e-14 1.938e+00 0.000 1.0000
## CountryZAI 3.263e-15 3.153e+00 0.000 1.0000
## ---
## Signif. codes: 0 '***' 0.001 '**' 0.01 '*' 0.05 '.' 0.1 ' ' 1
##
## (Dispersion parameter for gaussian family taken to be 6.626342)
##
## Null deviance: 1299.26 on 155 degrees of freedom
## Residual deviance: 934.31 on 141 degrees of freedom
## AIC: 753.94
##
## Number of Fisher Scoring iterations: 2

data$Country <- relevel(data$Country, ref= "ZAI")
model1<-glm((Prev_TcTsg) ~ Country,data=data, family=gaussian())
summary(model1)

##
## Call:
## glm(formula = (Prev_TcTsg) ~ Country, family = gaussian(), data = data)
##
## Deviance Residuals:
## Min 1Q Median 3Q Max
## -4.6633 -0.1012 0.0000 0.0000 16.4667
##
## Coefficients:
## Estimate Std. Error t value Pr(>|t|)
## (Intercept) 2.621e-14 2.574e+00 0.000 1.0000
## CountryMOZ -2.354e-14 3.153e+00 0.000 1.0000
## CountryMLI -2.361e-14 2.645e+00 0.000 1.0000
## CountryKEN 4.388e+00 2.679e+00 1.638 0.1037
## CountryETH 6.500e-01 3.640e+00 0.179 0.8585
## CountryGUI -3.327e-14 2.659e+00 0.000 1.0000
## CountryGHA -2.543e-14 2.645e+00 0.000 1.0000
## CountryBKF 1.012e-01 2.604e+00 0.039 0.9691
## CountryZIM 1.680e+00 2.700e+00 0.622 0.5348
## CountryZAM 7.530e+00 3.640e+00 2.068 0.0404 *
## CountrySAF 4.550e-01 2.730e+00 0.167 0.8679
## CountryUGA 2.120e-01 2.820e+00 0.075 0.9402
## CountryURT 4.663e+00 2.780e+00 1.677 0.0957 .
## CountrySWA -2.526e-14 3.640e+00 0.000 1.0000
## CountrySEN -3.073e-14 2.659e+00 0.000 1.0000
## ---
## Signif. codes: 0 '***' 0.001 '**' 0.01 '*' 0.05 '.' 0.1 ' ' 1
##
## (Dispersion parameter for gaussian family taken to be 6.626342)
##
## Null deviance: 1299.26 on 155 degrees of freedom
## Residual deviance: 934.31 on 141 degrees of freedom
## AIC: 753.94
##
## Number of Fisher Scoring iterations: 2

data$Country <- relevel(data$Country, ref= "SEN")
model1<-glm((Prev_TcTsg) ~ Country,data=data, family=gaussian())
summary(model1)

##
## Call:
## glm(formula = (Prev_TcTsg) ~ Country, family = gaussian(), data = data)
##
## Deviance Residuals:
## Min 1Q Median 3Q Max
## -4.6633 -0.1012 0.0000 0.0000 16.4667
##
## Coefficients:
## Estimate Std. Error t value Pr(>|t|)
## (Intercept) -5.202e-15 6.646e-01 0.000 1.000000
## CountryZAI 4.809e-15 2.659e+00 0.000 1.000000
## CountryMOZ 3.179e-15 1.938e+00 0.000 1.000000
## CountryMLI 3.176e-15 8.999e-01 0.000 1.000000
## CountryKEN 4.388e+00 9.970e-01 4.402 2.11e-05 ***
## CountryETH 6.500e-01 2.659e+00 0.244 0.807206
## CountryGUI 4.652e-15 9.400e-01 0.000 1.000000
## CountryGHA 1.110e-14 8.999e-01 0.000 1.000000
## CountryBKF 1.012e-01 7.719e-01 0.131 0.895920
## CountryZIM 1.680e+00 1.051e+00 1.599 0.112141
## CountryZAM 7.530e+00 2.659e+00 2.832 0.005298 **
## CountrySAF 4.550e-01 1.127e+00 0.404 0.687016
## CountryUGA 2.120e-01 1.329e+00 0.159 0.873517
## CountryURT 4.663e+00 1.243e+00 3.750 0.000257 ***
## CountrySWA 5.504e-15 2.659e+00 0.000 1.000000
## ---
## Signif. codes: 0 '***' 0.001 '**' 0.01 '*' 0.05 '.' 0.1 ' ' 1
##
## (Dispersion parameter for gaussian family taken to be 6.626342)
##
## Null deviance: 1299.26 on 155 degrees of freedom
## Residual deviance: 934.31 on 141 degrees of freedom
## AIC: 753.94
##
## Number of Fisher Scoring iterations: 2

data$Country <- relevel(data$Country, ref= "SWA")
model1<-glm((Prev_TcTsg) ~ Country,data=data, family=gaussian())
summary(model1)

##
## Call:
## glm(formula = (Prev_TcTsg) ~ Country, family = gaussian(), data = data)
##
## Deviance Residuals:
## Min 1Q Median 3Q Max
## -4.6633 -0.1012 0.0000 0.0000 16.4667
##
## Coefficients:
## Estimate Std. Error t value Pr(>|t|)
## (Intercept) 3.185e-14 2.574e+00 0.000 1.0000
## CountrySEN -3.144e-14 2.659e+00 0.000 1.0000
## CountryZAI -3.168e-14 3.640e+00 0.000 1.0000
## CountryMOZ -2.996e-14 3.153e+00 0.000 1.0000
## CountryMLI -6.544e-14 2.645e+00 0.000 1.0000
## CountryKEN 4.388e+00 2.679e+00 1.638 0.1037
## CountryETH 6.500e-01 3.640e+00 0.179 0.8585
## CountryGUI -3.930e-14 2.659e+00 0.000 1.0000
## CountryGHA -2.563e-14 2.645e+00 0.000 1.0000
## CountryBKF 1.012e-01 2.604e+00 0.039 0.9691
## CountryZIM 1.680e+00 2.700e+00 0.622 0.5348
## CountryZAM 7.530e+00 3.640e+00 2.068 0.0404 *
## CountrySAF 4.550e-01 2.730e+00 0.167 0.8679
## CountryUGA 2.120e-01 2.820e+00 0.075 0.9402
## CountryURT 4.663e+00 2.780e+00 1.677 0.0957 .
## ---
## Signif. codes: 0 '***' 0.001 '**' 0.01 '*' 0.05 '.' 0.1 ' ' 1
##
## (Dispersion parameter for gaussian family taken to be 6.626342)
##
## Null deviance: 1299.26 on 155 degrees of freedom
## Residual deviance: 934.31 on 141 degrees of freedom
## AIC: 753.94
##
## Number of Fisher Scoring iterations: 2

data$Country <- relevel(data$Country, ref= "URT")
model1<-glm((Prev_TcTz) ~ Country,data=data, family=gaussian())
summary(model1)

##
## Call:
## glm(formula = (Prev_TcTz) ~ Country, family = gaussian(), data = data)
##
## Deviance Residuals:
## Min 1Q Median 3Q Max
## -3.7894 -0.3586 0.0000 0.0000 18.4306
##
## Coefficients:
## Estimate Std. Error t value Pr(>|t|)
## (Intercept) 0.23500 1.09577 0.214 0.83050
## CountrySWA -0.23500 2.89915 -0.081 0.93551
## CountrySEN -0.23500 1.29654 -0.181 0.85643
## CountryZAI -0.23500 2.89915 -0.081 0.93551
## CountryMOZ -0.23500 2.19155 -0.107 0.91476
## CountryMLI -0.23500 1.26529 -0.186 0.85292
## CountryKEN 0.09917 1.34204 0.074 0.94120
## CountryETH -0.23500 2.89915 -0.081 0.93551
## CountryGUI -0.23500 1.29654 -0.181 0.85643
## CountryGHA 3.55444 1.26529 2.809 0.00567 **
## CountryBKF 0.12360 1.16973 0.106 0.91599
## CountryZIM -0.23500 1.38606 -0.170 0.86561
## CountryZAM -0.23500 2.89915 -0.081 0.93551
## CountrySAF -0.23500 1.44957 -0.162 0.87145
## CountryUGA -0.23500 1.62530 -0.145 0.88524
## ---
## Signif. codes: 0 '***' 0.001 '**' 0.01 '*' 0.05 '.' 0.1 ' ' 1
##
## (Dispersion parameter for gaussian family taken to be 7.204325)
##
## Null deviance: 1230.7 on 155 degrees of freedom
## Residual deviance: 1015.8 on 141 degrees of freedom
## AIC: 766.99
##
## Number of Fisher Scoring iterations: 2

data$Country <- relevel(data$Country, ref= "UGA")
model1<-glm((Prev_TcTsg) ~ Country,data=data, family=gaussian())
summary(model1)

##
## Call:
## glm(formula = (Prev_TcTsg) ~ Country, family = gaussian(), data = data)
##
## Deviance Residuals:
## Min 1Q Median 3Q Max
## -4.6633 -0.1012 0.0000 0.0000 16.4667
##
## Coefficients:
## Estimate Std. Error t value Pr(>|t|)
## (Intercept) 0.2120 1.1512 0.184 0.85416
## CountryURT 4.4513 1.5587 2.856 0.00494 **
## CountrySWA -0.2120 2.8199 -0.075 0.94018
## CountrySEN -0.2120 1.3293 -0.159 0.87352
## CountryZAI -0.2120 2.8199 -0.075 0.94018
## CountryMOZ -0.2120 2.1537 -0.098 0.92173
## CountryMLI -0.2120 1.3013 -0.163 0.87082
## CountryKEN 4.1763 1.3702 3.048 0.00275 **
## CountryETH 0.4380 2.8199 0.155 0.87679
## CountryGUI -0.2120 1.3293 -0.159 0.87352
## CountryGHA -0.2120 1.3013 -0.163 0.87082
## CountryBKF -0.1108 1.2163 -0.091 0.92752
## CountryZIM 1.4680 1.4099 1.041 0.29957
## CountryZAM 7.3180 2.8199 2.595 0.01045 *
## CountrySAF 0.2430 1.4675 0.166 0.86872
## ---
## Signif. codes: 0 '***' 0.001 '**' 0.01 '*' 0.05 '.' 0.1 ' ' 1
##
## (Dispersion parameter for gaussian family taken to be 6.626342)
##
## Null deviance: 1299.26 on 155 degrees of freedom
## Residual deviance: 934.31 on 141 degrees of freedom
## AIC: 753.94
##
## Number of Fisher Scoring iterations: 2

data$Country <- relevel(data$Country, ref= "SAF")
model1<-glm((Prev_TcTsg) ~ Country,data=data, family=gaussian())
summary(model1)

##
## Call:
## glm(formula = (Prev_TcTsg) ~ Country, family = gaussian(), data = data)
##
## Deviance Residuals:
## Min 1Q Median 3Q Max
## -4.6633 -0.1012 0.0000 0.0000 16.4667
##
## Coefficients:
## Estimate Std. Error t value Pr(>|t|)
## (Intercept) 0.4550 0.9101 0.500 0.61790
## CountryUGA -0.2430 1.4675 -0.166 0.86872
## CountryURT 4.2083 1.3902 3.027 0.00294 **
## CountrySWA -0.4550 2.7303 -0.167 0.86789
## CountrySEN -0.4550 1.1270 -0.404 0.68702
## CountryZAI -0.4550 2.7303 -0.167 0.86789
## CountryMOZ -0.4550 2.0351 -0.224 0.82341
## CountryMLI -0.4550 1.0938 -0.416 0.67806
## CountryKEN 3.9333 1.1749 3.348 0.00105 **
## CountryETH 0.1950 2.7303 0.071 0.94316
## CountryGUI -0.4550 1.1270 -0.404 0.68702
## CountryGHA -0.4550 1.0938 -0.416 0.67806
## CountryBKF -0.3538 0.9912 -0.357 0.72163
## CountryZIM 1.2250 1.2210 1.003 0.31746
## CountryZAM 7.0750 2.7303 2.591 0.01057 *
## ---
## Signif. codes: 0 '***' 0.001 '**' 0.01 '*' 0.05 '.' 0.1 ' ' 1
##
## (Dispersion parameter for gaussian family taken to be 6.626342)
##
## Null deviance: 1299.26 on 155 degrees of freedom
## Residual deviance: 934.31 on 141 degrees of freedom
## AIC: 753.94
##
## Number of Fisher Scoring iterations: 2

data$Country <- relevel(data$Country, ref= "ZAM")
model1<-glm((Prev_TcTsg) ~ Country,data=data, family=gaussian())
summary(model1)

##
## Call:
## glm(formula = (Prev_TcTsg) ~ Country, family = gaussian(), data = data)
##
## Deviance Residuals:
## Min 1Q Median 3Q Max
## -4.6633 -0.1012 0.0000 0.0000 16.4667
##
## Coefficients:
## Estimate Std. Error t value Pr(>|t|)
## (Intercept) 7.530 2.574 2.925 0.00401 **
## CountrySAF -7.075 2.730 -2.591 0.01057 *
## CountryUGA -7.318 2.820 -2.595 0.01045 *
## CountryURT -2.867 2.780 -1.031 0.30430
## CountrySWA -7.530 3.640 -2.068 0.04043 *
## CountrySEN -7.530 2.659 -2.832 0.00530 **
## CountryZAI -7.530 3.640 -2.068 0.04043 *
## CountryMOZ -7.530 3.153 -2.388 0.01824 *
## CountryMLI -7.530 2.645 -2.847 0.00507 **
## CountryKEN -3.142 2.679 -1.173 0.24294
## CountryETH -6.880 3.640 -1.890 0.06083 .
## CountryGUI -7.530 2.659 -2.832 0.00530 **
## CountryGHA -7.530 2.645 -2.847 0.00507 **
## CountryBKF -7.429 2.604 -2.853 0.00498 **
## CountryZIM -5.850 2.700 -2.167 0.03193 *
## ---
## Signif. codes: 0 '***' 0.001 '**' 0.01 '*' 0.05 '.' 0.1 ' ' 1
##
## (Dispersion parameter for gaussian family taken to be 6.626342)
##
## Null deviance: 1299.26 on 155 degrees of freedom
## Residual deviance: 934.31 on 141 degrees of freedom
## AIC: 753.94
##
## Number of Fisher Scoring iterations: 2

data$Country <- relevel(data$Country, ref= "ZIM")
model1<-glm((Prev_TcTsg) ~ Country,data=data, family=gaussian())
summary(model1)

##
## Call:
## glm(formula = (Prev_TcTsg) ~ Country, family = gaussian(), data = data)
##
## Deviance Residuals:
## Min 1Q Median 3Q Max
## -4.6633 -0.1012 0.0000 0.0000 16.4667
##
## Coefficients:
## Estimate Std. Error t value Pr(>|t|)
## (Intercept) 1.6800 0.8140 2.064 0.0409 *
## CountryZAM 5.8500 2.6998 2.167 0.0319 *
## CountrySAF -1.2250 1.2210 -1.003 0.3175
## CountryUGA -1.4680 1.4099 -1.041 0.2996
## CountryURT 2.9833 1.3293 2.244 0.0264 *
## CountrySWA -1.6800 2.6998 -0.622 0.5348
## CountrySEN -1.6800 1.0509 -1.599 0.1121
## CountryZAI -1.6800 2.6998 -0.622 0.5348
## CountryMOZ -1.6800 1.9939 -0.843 0.4009
## CountryMLI -1.6800 1.0153 -1.655 0.1002
## CountryKEN 2.7083 1.1022 2.457 0.0152 *
## CountryETH -1.0300 2.6998 -0.382 0.7034
## CountryGUI -1.6800 1.0509 -1.599 0.1121
## CountryGHA -1.6800 1.0153 -1.655 0.1002
## CountryBKF -1.5788 0.9037 -1.747 0.0828 .
## ---
## Signif. codes: 0 '***' 0.001 '**' 0.01 '*' 0.05 '.' 0.1 ' ' 1
##
## (Dispersion parameter for gaussian family taken to be 6.626342)
##
## Null deviance: 1299.26 on 155 degrees of freedom
## Residual deviance: 934.31 on 141 degrees of freedom
## AIC: 753.94
##
## Number of Fisher Scoring iterations: 2

#======== Glm TvTz per country
data$Country <- relevel(data$Country, ref= "BKF")
model1<-glm((Prev_TvTz) ~ Country,data=data, family=gaussian())
summary(model1)

##
## Call:
## glm(formula = (Prev_TvTz) ~ Country, family = gaussian(), data = data)
##
## Deviance Residuals:
## Min 1Q Median 3Q Max
## -13.394 -0.480 -0.086 0.000 69.936
##
## Coefficients:
## Estimate Std. Error t value Pr(>|t|)
## (Intercept) 0.4800 1.3507 0.355 0.723
## CountryZIM -0.4800 3.1096 -0.154 0.878
## CountryZAM -0.4800 8.9598 -0.054 0.957
## CountrySAF -0.4800 3.4105 -0.141 0.888
## CountryUGA -0.4800 4.1851 -0.115 0.909
## CountryURT -0.2450 3.8601 -0.063 0.949
## CountrySWA -0.4800 8.9598 -0.054 0.957
## CountrySEN 1.0333 2.6561 0.389 0.698
## CountryZAI -0.4800 8.9598 -0.054 0.957
## CountryMOZ -0.4800 6.4071 -0.075 0.940
## CountryMLI -0.4800 2.4866 -0.193 0.847
## CountryKEN -0.3942 2.8918 -0.136 0.892
## CountryETH -0.4800 8.9598 -0.054 0.957
## CountryGUI -0.4800 2.6561 -0.181 0.857
## CountryGHA 12.9139 2.4866 5.193 7.08e-07 ***
## ---
## Signif. codes: 0 '***' 0.001 '**' 0.01 '*' 0.05 '.' 0.1 ' ' 1
##
## (Dispersion parameter for gaussian family taken to be 78.45348)
##
## Null deviance: 13808 on 155 degrees of freedom
## Residual deviance: 11062 on 141 degrees of freedom
## AIC: 1139.5
##
## Number of Fisher Scoring iterations: 2

data$Country <- relevel(data$Country, ref= "GHA")
model1<-glm((Prev_TvTz) ~ Country,data=data, family=gaussian())
summary(model1)

##
## Call:
## glm(formula = (Prev_TvTz) ~ Country, family = gaussian(), data = data)
##
## Deviance Residuals:
## Min 1Q Median 3Q Max
## -13.394 -0.480 -0.086 0.000 69.936
##
## Coefficients:
## Estimate Std. Error t value Pr(>|t|)
## (Intercept) 13.394 2.088 6.416 1.98e-09 ***
## CountryBKF -12.914 2.487 -5.193 7.08e-07 ***
## CountryZIM -13.394 3.493 -3.834 0.000189 ***
## CountryZAM -13.394 9.100 -1.472 0.143293
## CountrySAF -13.394 3.764 -3.559 0.000508 ***
## CountryUGA -13.394 4.478 -2.991 0.003280 **
## CountryURT -13.159 4.175 -3.152 0.001984 **
## CountrySWA -13.394 9.100 -1.472 0.143293
## CountrySEN -11.881 3.097 -3.837 0.000188 ***
## CountryZAI -13.394 9.100 -1.472 0.143293
## CountryMOZ -13.394 6.602 -2.029 0.044362 *
## CountryMLI -13.394 2.952 -4.537 1.21e-05 ***
## CountryKEN -13.308 3.301 -4.032 9.03e-05 ***
## CountryETH -13.394 9.100 -1.472 0.143293
## CountryGUI -13.394 3.097 -4.325 2.86e-05 ***
## ---
## Signif. codes: 0 '***' 0.001 '**' 0.01 '*' 0.05 '.' 0.1 ' ' 1
##
## (Dispersion parameter for gaussian family taken to be 78.45348)
##
## Null deviance: 13808 on 155 degrees of freedom
## Residual deviance: 11062 on 141 degrees of freedom
## AIC: 1139.5
##
## Number of Fisher Scoring iterations: 2

data$Country <- relevel(data$Country, ref= "GUI")
model1<-glm((Prev_TvTz) ~ Country,data=data, family=gaussian())
summary(model1)

##
## Call:
## glm(formula = (Prev_TvTz) ~ Country, family = gaussian(), data = data)
##
## Deviance Residuals:
## Min 1Q Median 3Q Max
## -13.394 -0.480 -0.086 0.000 69.936
##
## Coefficients:
## Estimate Std. Error t value Pr(>|t|)
## (Intercept) 1.991e-15 2.287e+00 0.000 1.000
## CountryGHA 1.339e+01 3.097e+00 4.325 2.86e-05 ***
## CountryBKF 4.800e-01 2.656e+00 0.181 0.857
## CountryZIM -9.384e-16 3.616e+00 0.000 1.000
## CountryZAM -9.099e-16 9.148e+00 0.000 1.000
## CountrySAF -1.854e-15 3.878e+00 0.000 1.000
## CountryUGA -2.235e-15 4.574e+00 0.000 1.000
## CountryURT 2.350e-01 4.279e+00 0.055 0.956
## CountrySWA -2.238e-16 9.148e+00 0.000 1.000
## CountrySEN 1.513e+00 3.234e+00 0.468 0.641
## CountryZAI -1.418e-15 9.148e+00 0.000 1.000
## CountryMOZ -1.136e-15 6.668e+00 0.000 1.000
## CountryMLI -1.794e-15 3.097e+00 0.000 1.000
## CountryKEN 8.583e-02 3.430e+00 0.025 0.980
## CountryETH -2.236e-15 9.148e+00 0.000 1.000
## ---
## Signif. codes: 0 '***' 0.001 '**' 0.01 '*' 0.05 '.' 0.1 ' ' 1
##
## (Dispersion parameter for gaussian family taken to be 78.45348)
##
## Null deviance: 13808 on 155 degrees of freedom
## Residual deviance: 11062 on 141 degrees of freedom
## AIC: 1139.5
##
## Number of Fisher Scoring iterations: 2

data$Country <- relevel(data$Country, ref= "ETH")
model1<-glm((Prev_TvTz) ~ Country,data=data, family=gaussian())
summary(model1)

##
## Call:
## glm(formula = (Prev_TvTz) ~ Country, family = gaussian(), data = data)
##
## Deviance Residuals:
## Min 1Q Median 3Q Max
## -13.394 -0.480 -0.086 0.000 69.936
##
## Coefficients:
## Estimate Std. Error t value Pr(>|t|)
## (Intercept) 9.020e-15 8.857e+00 0.000 1.000
## CountryGUI -5.066e-15 9.148e+00 0.000 1.000
## CountryGHA 1.339e+01 9.100e+00 1.472 0.143
## CountryBKF 4.800e-01 8.960e+00 0.054 0.957
## CountryZIM -9.356e-15 9.290e+00 0.000 1.000
## CountryZAM -9.787e-15 1.253e+01 0.000 1.000
## CountrySAF -1.187e-14 9.395e+00 0.000 1.000
## CountryUGA -1.089e-14 9.703e+00 0.000 1.000
## CountryURT 2.350e-01 9.567e+00 0.025 0.980
## CountrySWA -9.925e-15 1.253e+01 0.000 1.000
## CountrySEN 1.513e+00 9.148e+00 0.165 0.869
## CountryZAI -9.733e-15 1.253e+01 0.000 1.000
## CountryMOZ -1.060e-14 1.085e+01 0.000 1.000
## CountryMLI -9.415e-15 9.100e+00 0.000 1.000
## CountryKEN 8.583e-02 9.219e+00 0.009 0.993
##
## (Dispersion parameter for gaussian family taken to be 78.45348)
##
## Null deviance: 13808 on 155 degrees of freedom
## Residual deviance: 11062 on 141 degrees of freedom
## AIC: 1139.5
##
## Number of Fisher Scoring iterations: 2

data$Country <- relevel(data$Country, ref= "KEN")
model1<-glm((Prev_TvTz) ~ Country,data=data, family=gaussian())
summary(model1)

##
## Call:
## glm(formula = (Prev_TvTz) ~ Country, family = gaussian(), data = data)
##
## Deviance Residuals:
## Min 1Q Median 3Q Max
## -13.394 -0.480 -0.086 0.000 69.936
##
## Coefficients:
## Estimate Std. Error t value Pr(>|t|)
## (Intercept) 0.08583 2.55691 0.034 0.973
## CountryETH -0.08583 9.21907 -0.009 0.993
## CountryGUI -0.08583 3.43046 -0.025 0.980
## CountryGHA 13.30806 3.30096 4.032 9.03e-05 ***
## CountryBKF 0.39417 2.89176 0.136 0.892
## CountryZIM -0.08583 3.79251 -0.023 0.982
## CountryZAM -0.08583 9.21907 -0.009 0.993
## CountrySAF -0.08583 4.04283 -0.021 0.983
## CountryUGA -0.08583 4.71471 -0.018 0.986
## CountryURT 0.14917 4.42870 0.034 0.973
## CountrySWA -0.08583 9.21907 -0.009 0.993
## CountrySEN 1.42750 3.43046 0.416 0.678
## CountryZAI -0.08583 9.21907 -0.009 0.993
## CountryMOZ -0.08583 6.76495 -0.013 0.990
## CountryMLI -0.08583 3.30096 -0.026 0.979
## ---
## Signif. codes: 0 '***' 0.001 '**' 0.01 '*' 0.05 '.' 0.1 ' ' 1
##
## (Dispersion parameter for gaussian family taken to be 78.45348)
##
## Null deviance: 13808 on 155 degrees of freedom
## Residual deviance: 11062 on 141 degrees of freedom
## AIC: 1139.5
##
## Number of Fisher Scoring iterations: 2

data$Country <- relevel(data$Country, ref= "MLI")
model1<-glm((Prev_TvTz) ~ Country,data=data, family=gaussian())
summary(model1)

##
## Call:
## glm(formula = (Prev_TvTz) ~ Country, family = gaussian(), data = data)
##
## Deviance Residuals:
## Min 1Q Median 3Q Max
## -13.394 -0.480 -0.086 0.000 69.936
##
## Coefficients:
## Estimate Std. Error t value Pr(>|t|)
## (Intercept) 7.044e-16 2.088e+00 0.000 1.000
## CountryKEN 8.583e-02 3.301e+00 0.026 0.979
## CountryETH 2.829e-14 9.100e+00 0.000 1.000
## CountryGUI -4.363e-15 3.097e+00 0.000 1.000
## CountryGHA 1.339e+01 2.952e+00 4.537 1.21e-05 ***
## CountryBKF 4.800e-01 2.487e+00 0.193 0.847
## CountryZIM 1.089e-15 3.493e+00 0.000 1.000
## CountryZAM -1.762e-15 9.100e+00 0.000 1.000
## CountrySAF 8.197e-16 3.764e+00 0.000 1.000
## CountryUGA 1.967e-15 4.478e+00 0.000 1.000
## CountryURT 2.350e-01 4.175e+00 0.056 0.955
## CountrySWA 1.013e-15 9.100e+00 0.000 1.000
## CountrySEN 1.513e+00 3.097e+00 0.489 0.626
## CountryZAI 5.999e-16 9.100e+00 0.000 1.000
## CountryMOZ 3.103e-16 6.602e+00 0.000 1.000
## ---
## Signif. codes: 0 '***' 0.001 '**' 0.01 '*' 0.05 '.' 0.1 ' ' 1
##
## (Dispersion parameter for gaussian family taken to be 78.45348)
##
## Null deviance: 13808 on 155 degrees of freedom
## Residual deviance: 11062 on 141 degrees of freedom
## AIC: 1139.5
##
## Number of Fisher Scoring iterations: 2

data$Country <- relevel(data$Country, ref= "MOZ")
model1<-glm((Prev_TvTz) ~ Country,data=data, family=gaussian())
summary(model1)

##
## Call:
## glm(formula = (Prev_TvTz) ~ Country, family = gaussian(), data = data)
##
## Deviance Residuals:
## Min 1Q Median 3Q Max
## -13.394 -0.480 -0.086 0.000 69.936
##
## Coefficients:
## Estimate Std. Error t value Pr(>|t|)
## (Intercept) 2.569e-15 6.263e+00 0.000 1.0000
## CountryMLI -1.635e-15 6.602e+00 0.000 1.0000
## CountryKEN 8.583e-02 6.765e+00 0.013 0.9899
## CountryETH -2.195e-14 1.085e+01 0.000 1.0000
## CountryGUI -8.312e-15 6.668e+00 0.000 1.0000
## CountryGHA 1.339e+01 6.602e+00 2.029 0.0444 *
## CountryBKF 4.800e-01 6.407e+00 0.075 0.9404
## CountryZIM -3.063e-15 6.861e+00 0.000 1.0000
## CountryZAM -4.752e-15 1.085e+01 0.000 1.0000
## CountrySAF -3.912e-15 7.002e+00 0.000 1.0000
## CountryUGA -5.976e-15 7.411e+00 0.000 1.0000
## CountryURT 2.350e-01 7.232e+00 0.032 0.9741
## CountrySWA -3.878e-15 1.085e+01 0.000 1.0000
## CountrySEN 1.513e+00 6.668e+00 0.227 0.8208
## CountryZAI -4.147e-15 1.085e+01 0.000 1.0000
## ---
## Signif. codes: 0 '***' 0.001 '**' 0.01 '*' 0.05 '.' 0.1 ' ' 1
##
## (Dispersion parameter for gaussian family taken to be 78.45348)
##
## Null deviance: 13808 on 155 degrees of freedom
## Residual deviance: 11062 on 141 degrees of freedom
## AIC: 1139.5
##
## Number of Fisher Scoring iterations: 2

data$Country <- relevel(data$Country, ref= "ZAI")
model1<-glm((Prev_TvTz) ~ Country,data=data, family=gaussian())
summary(model1)

##
## Call:
## glm(formula = (Prev_TvTz) ~ Country, family = gaussian(), data = data)
##
## Deviance Residuals:
## Min 1Q Median 3Q Max
## -13.394 -0.480 -0.086 0.000 69.936
##
## Coefficients:
## Estimate Std. Error t value Pr(>|t|)
## (Intercept) 1.867e-14 8.857e+00 0.000 1.000
## CountryMOZ -1.760e-14 1.085e+01 0.000 1.000
## CountryMLI -1.961e-14 9.100e+00 0.000 1.000
## CountryKEN 8.583e-02 9.219e+00 0.009 0.993
## CountryETH -1.037e-14 1.253e+01 0.000 1.000
## CountryGUI -1.371e-14 9.148e+00 0.000 1.000
## CountryGHA 1.339e+01 9.100e+00 1.472 0.143
## CountryBKF 4.800e-01 8.960e+00 0.054 0.957
## CountryZIM -1.942e-14 9.290e+00 0.000 1.000
## CountryZAM -2.245e-14 1.253e+01 0.000 1.000
## CountrySAF -2.159e-14 9.395e+00 0.000 1.000
## CountryUGA -2.163e-14 9.703e+00 0.000 1.000
## CountryURT 2.350e-01 9.567e+00 0.025 0.980
## CountrySWA -2.266e-14 1.253e+01 0.000 1.000
## CountrySEN 1.513e+00 9.148e+00 0.165 0.869
##
## (Dispersion parameter for gaussian family taken to be 78.45348)
##
## Null deviance: 13808 on 155 degrees of freedom
## Residual deviance: 11062 on 141 degrees of freedom
## AIC: 1139.5
##
## Number of Fisher Scoring iterations: 2

data$Country <- relevel(data$Country, ref= "SEN")
model1<-glm((Prev_TvTz) ~ Country,data=data, family=gaussian())
summary(model1)

##
## Call:
## glm(formula = (Prev_TvTz) ~ Country, family = gaussian(), data = data)
##
## Deviance Residuals:
## Min 1Q Median 3Q Max
## -13.394 -0.480 -0.086 0.000 69.936
##
## Coefficients:
## Estimate Std. Error t value Pr(>|t|)
## (Intercept) 1.513 2.287 0.662 0.509231
## CountryZAI -1.513 9.148 -0.165 0.868843
## CountryMOZ -1.513 6.668 -0.227 0.820777
## CountryMLI -1.513 3.097 -0.489 0.625805
## CountryKEN -1.427 3.430 -0.416 0.677951
## CountryETH -1.513 9.148 -0.165 0.868843
## CountryGUI -1.513 3.234 -0.468 0.640574
## CountryGHA 11.881 3.097 3.837 0.000188 ***
## CountryBKF -1.033 2.656 -0.389 0.697830
## CountryZIM -1.513 3.616 -0.419 0.676212
## CountryZAM -1.513 9.148 -0.165 0.868843
## CountrySAF -1.513 3.878 -0.390 0.696932
## CountryUGA -1.513 4.574 -0.331 0.741241
## CountryURT -1.278 4.279 -0.299 0.765549
## CountrySWA -1.513 9.148 -0.165 0.868843
## ---
## Signif. codes: 0 '***' 0.001 '**' 0.01 '*' 0.05 '.' 0.1 ' ' 1
##
## (Dispersion parameter for gaussian family taken to be 78.45348)
##
## Null deviance: 13808 on 155 degrees of freedom
## Residual deviance: 11062 on 141 degrees of freedom
## AIC: 1139.5
##
## Number of Fisher Scoring iterations: 2

data$Country <- relevel(data$Country, ref= "SWA")
model1<-glm((Prev_TvTz) ~ Country,data=data, family=gaussian())
summary(model1)

##
## Call:
## glm(formula = (Prev_TvTz) ~ Country, family = gaussian(), data = data)
##
## Deviance Residuals:
## Min 1Q Median 3Q Max
## -13.394 -0.480 -0.086 0.000 69.936
##
## Coefficients:
## Estimate Std. Error t value Pr(>|t|)
## (Intercept) 1.476e-15 8.857e+00 0.000 1.000
## CountrySEN 1.513e+00 9.148e+00 0.165 0.869
## CountryZAI -4.630e-15 1.253e+01 0.000 1.000
## CountryMOZ -1.373e-15 1.085e+01 0.000 1.000
## CountryMLI 3.087e-16 9.100e+00 0.000 1.000
## CountryKEN 8.583e-02 9.219e+00 0.009 0.993
## CountryETH 4.353e-15 1.253e+01 0.000 1.000
## CountryGUI -3.957e-15 9.148e+00 0.000 1.000
## CountryGHA 1.339e+01 9.100e+00 1.472 0.143
## CountryBKF 4.800e-01 8.960e+00 0.054 0.957
## CountryZIM -2.172e-15 9.290e+00 0.000 1.000
## CountryZAM 7.878e-15 1.253e+01 0.000 1.000
## CountrySAF -6.047e-16 9.395e+00 0.000 1.000
## CountryUGA -3.640e-15 9.703e+00 0.000 1.000
## CountryURT 2.350e-01 9.567e+00 0.025 0.980
##
## (Dispersion parameter for gaussian family taken to be 78.45348)
##
## Null deviance: 13808 on 155 degrees of freedom
## Residual deviance: 11062 on 141 degrees of freedom
## AIC: 1139.5
##
## Number of Fisher Scoring iterations: 2

data$Country <- relevel(data$Country, ref= "URT")
model1<-glm((Prev_TvTz) ~ Country,data=data, family=gaussian())
summary(model1)

##
## Call:
## glm(formula = (Prev_TvTz) ~ Country, family = gaussian(), data = data)
##
## Deviance Residuals:
## Min 1Q Median 3Q Max
## -13.394 -0.480 -0.086 0.000 69.936
##
## Coefficients:
## Estimate Std. Error t value Pr(>|t|)
## (Intercept) 0.2350 3.6160 0.065 0.94828
## CountrySWA -0.2350 9.5671 -0.025 0.98044
## CountrySEN 1.2783 4.2785 0.299 0.76555
## CountryZAI -0.2350 9.5671 -0.025 0.98044
## CountryMOZ -0.2350 7.2320 -0.032 0.97412
## CountryMLI -0.2350 4.1754 -0.056 0.95520
## CountryKEN -0.1492 4.4287 -0.034 0.97318
## CountryETH -0.2350 9.5671 -0.025 0.98044
## CountryGUI -0.2350 4.2785 -0.055 0.95628
## CountryGHA 13.1589 4.1754 3.152 0.00198 **
## CountryBKF 0.2450 3.8601 0.063 0.94948
## CountryZIM -0.2350 4.5739 -0.051 0.95910
## CountryZAM -0.2350 9.5671 -0.025 0.98044
## CountrySAF -0.2350 4.7835 -0.049 0.96089
## CountryUGA -0.2350 5.3634 -0.044 0.96511
## ---
## Signif. codes: 0 '***' 0.001 '**' 0.01 '*' 0.05 '.' 0.1 ' ' 1
##
## (Dispersion parameter for gaussian family taken to be 78.45348)
##
## Null deviance: 13808 on 155 degrees of freedom
## Residual deviance: 11062 on 141 degrees of freedom
## AIC: 1139.5
##
## Number of Fisher Scoring iterations: 2

data$Country <- relevel(data$Country, ref= "UGA")
model1<-glm((Prev_TvTz) ~ Country,data=data, family=gaussian())
summary(model1)

##
## Call:
## glm(formula = (Prev_TvTz) ~ Country, family = gaussian(), data = data)
##
## Deviance Residuals:
## Min 1Q Median 3Q Max
## -13.394 -0.480 -0.086 0.000 69.936
##
## Coefficients:
## Estimate Std. Error t value Pr(>|t|)
## (Intercept) 5.677e-15 3.961e+00 0.000 1.00000
## CountryURT 2.350e-01 5.363e+00 0.044 0.96511
## CountrySWA -4.985e-15 9.703e+00 0.000 1.00000
## CountrySEN 1.513e+00 4.574e+00 0.331 0.74124
## CountryZAI -7.804e-15 9.703e+00 0.000 1.00000
## CountryMOZ -7.172e-15 7.411e+00 0.000 1.00000
## CountryMLI -8.049e-15 4.478e+00 0.000 1.00000
## CountryKEN 8.583e-02 4.715e+00 0.018 0.98550
## CountryETH -2.382e-15 9.703e+00 0.000 1.00000
## CountryGUI -3.980e-15 4.574e+00 0.000 1.00000
## CountryGHA 1.339e+01 4.478e+00 2.991 0.00328 **
## CountryBKF 4.800e-01 4.185e+00 0.115 0.90885
## CountryZIM -5.103e-15 4.851e+00 0.000 1.00000
## CountryZAM 3.876e-15 9.703e+00 0.000 1.00000
## CountrySAF -3.623e-15 5.049e+00 0.000 1.00000
## ---
## Signif. codes: 0 '***' 0.001 '**' 0.01 '*' 0.05 '.' 0.1 ' ' 1
##
## (Dispersion parameter for gaussian family taken to be 78.45348)
##
## Null deviance: 13808 on 155 degrees of freedom
## Residual deviance: 11062 on 141 degrees of freedom
## AIC: 1139.5
##
## Number of Fisher Scoring iterations: 2

data$Country <- relevel(data$Country, ref= "SAF")
model1<-glm((Prev_TvTz) ~ Country,data=data, family=gaussian())
summary(model1)

##
## Call:
## glm(formula = (Prev_TvTz) ~ Country, family = gaussian(), data = data)
##
## Deviance Residuals:
## Min 1Q Median 3Q Max
## -13.394 -0.480 -0.086 0.000 69.936
##
## Coefficients:
## Estimate Std. Error t value Pr(>|t|)
## (Intercept) 1.565e-15 3.132e+00 0.000 1.000000
## CountryUGA 1.221e-15 5.049e+00 0.000 1.000000
## CountryURT 2.350e-01 4.784e+00 0.049 0.960888
## CountrySWA 6.406e-16 9.395e+00 0.000 1.000000
## CountrySEN 1.513e+00 3.878e+00 0.390 0.696932
## CountryZAI 3.824e-15 9.395e+00 0.000 1.000000
## CountryMOZ -1.667e-16 7.002e+00 0.000 1.000000
## CountryMLI -2.586e-15 3.764e+00 0.000 1.000000
## CountryKEN 8.583e-02 4.043e+00 0.021 0.983091
## CountryETH 1.305e-14 9.395e+00 0.000 1.000000
## CountryGUI 3.499e-15 3.878e+00 0.000 1.000000
## CountryGHA 1.339e+01 3.764e+00 3.559 0.000508 ***
## CountryBKF 4.800e-01 3.410e+00 0.141 0.888273
## CountryZIM -2.427e-15 4.201e+00 0.000 1.000000
## CountryZAM 3.179e-15 9.395e+00 0.000 1.000000
## ---
## Signif. codes: 0 '***' 0.001 '**' 0.01 '*' 0.05 '.' 0.1 ' ' 1
##
## (Dispersion parameter for gaussian family taken to be 78.45348)
##
## Null deviance: 13808 on 155 degrees of freedom
## Residual deviance: 11062 on 141 degrees of freedom
## AIC: 1139.5
##
## Number of Fisher Scoring iterations: 2

data$Country <- relevel(data$Country, ref= "ZAM")
model1<-glm((Prev_TvTz) ~ Country,data=data, family=gaussian())
summary(model1)

##
## Call:
## glm(formula = (Prev_TvTz) ~ Country, family = gaussian(), data = data)
##
## Deviance Residuals:
## Min 1Q Median 3Q Max
## -13.394 -0.480 -0.086 0.000 69.936
##
## Coefficients:
## Estimate Std. Error t value Pr(>|t|)
## (Intercept) 3.835e-14 8.857e+00 0.000 1.000
## CountrySAF -3.678e-14 9.395e+00 0.000 1.000
## CountryUGA -3.386e-14 9.703e+00 0.000 1.000
## CountryURT 2.350e-01 9.567e+00 0.025 0.980
## CountrySWA -3.875e-14 1.253e+01 0.000 1.000
## CountrySEN 1.513e+00 9.148e+00 0.165 0.869
## CountryZAI -4.028e-14 1.253e+01 0.000 1.000
## CountryMOZ -4.177e-14 1.085e+01 0.000 1.000
## CountryMLI -3.875e-14 9.100e+00 0.000 1.000
## CountryKEN 8.583e-02 9.219e+00 0.009 0.993
## CountryETH -4.546e-14 1.253e+01 0.000 1.000
## CountryGUI -3.832e-14 9.148e+00 0.000 1.000
## CountryGHA 1.339e+01 9.100e+00 1.472 0.143
## CountryBKF 4.800e-01 8.960e+00 0.054 0.957
## CountryZIM -3.656e-14 9.290e+00 0.000 1.000
##
## (Dispersion parameter for gaussian family taken to be 78.45348)
##
## Null deviance: 13808 on 155 degrees of freedom
## Residual deviance: 11062 on 141 degrees of freedom
## AIC: 1139.5
##
## Number of Fisher Scoring iterations: 2

data$Country <- relevel(data$Country, ref= "ZIM")
model1<-glm((Prev_TvTz) ~ Country,data=data, family=gaussian())
summary(model1)

##
## Call:
## glm(formula = (Prev_TvTz) ~ Country, family = gaussian(), data = data)
##
## Deviance Residuals:
## Min 1Q Median 3Q Max
## -13.394 -0.480 -0.086 0.000 69.936
##
## Coefficients:
## Estimate Std. Error t value Pr(>|t|)
## (Intercept) -3.951e-16 2.801e+00 0.000 1.000000
## CountryZAM -6.294e-15 9.290e+00 0.000 1.000000
## CountrySAF -5.800e-16 4.201e+00 0.000 1.000000
## CountryUGA 1.167e-15 4.851e+00 0.000 1.000000
## CountryURT 2.350e-01 4.574e+00 0.051 0.959097
## CountrySWA 1.059e-15 9.290e+00 0.000 1.000000
## CountrySEN 1.513e+00 3.616e+00 0.419 0.676212
## CountryZAI -5.622e-15 9.290e+00 0.000 1.000000
## CountryMOZ 4.382e-15 6.861e+00 0.000 1.000000
## CountryMLI -1.316e-16 3.493e+00 0.000 1.000000
## CountryKEN 8.583e-02 3.793e+00 0.023 0.981976
## CountryETH -8.040e-15 9.290e+00 0.000 1.000000
## CountryGUI 0.000e+00 3.616e+00 0.000 1.000000
## CountryGHA 1.339e+01 3.493e+00 3.834 0.000189 ***
## CountryBKF 4.800e-01 3.110e+00 0.154 0.877548
## ---
## Signif. codes: 0 '***' 0.001 '**' 0.01 '*' 0.05 '.' 0.1 ' ' 1
##
## (Dispersion parameter for gaussian family taken to be 78.45348)
##
## Null deviance: 13808 on 155 degrees of freedom
## Residual deviance: 11062 on 141 degrees of freedom
## AIC: 1139.5
##
## Number of Fisher Scoring iterations: 2

#======== Glm TvTsg per country
data$Country <- relevel(data$Country, ref= "BKF")
model1<-glm((Prev_TvTsg) ~ Country,data=data, family=gaussian())
summary(model1)

##
## Call:
## glm(formula = (Prev_TvTsg) ~ Country, family = gaussian(), data = data)
##
## Deviance Residuals:
## Min 1Q Median 3Q Max
## -6.536 -0.104 0.000 0.000 60.134
##
## Coefficients:
## Estimate Std. Error t value Pr(>|t|)
## (Intercept) 6.536 1.141 5.729 5.87e-08 ***
## CountryZIM -6.536 2.626 -2.489 0.01399 *
## CountryZAM -6.536 7.567 -0.864 0.38921
## CountrySAF -6.536 2.880 -2.269 0.02478 *
## CountryUGA -6.536 3.535 -1.849 0.06654 .
## CountryURT -5.394 3.260 -1.655 0.10022
## CountrySWA -6.536 7.567 -0.864 0.38921
## CountrySEN -6.536 2.243 -2.914 0.00416 **
## CountryZAI -6.536 7.567 -0.864 0.38921
## CountryMOZ -5.536 5.411 -1.023 0.30804
## CountryMLI -6.536 2.100 -3.112 0.00225 **
## CountryKEN -6.432 2.442 -2.633 0.00940 **
## CountryETH -6.096 7.567 -0.806 0.42185
## CountryGUI -6.536 2.243 -2.914 0.00416 **
## CountryGHA -6.536 2.100 -3.112 0.00225 **
## ---
## Signif. codes: 0 '***' 0.001 '**' 0.01 '*' 0.05 '.' 0.1 ' ' 1
##
## (Dispersion parameter for gaussian family taken to be 55.96374)
##
## Null deviance: 9193.0 on 155 degrees of freedom
## Residual deviance: 7890.9 on 141 degrees of freedom
## AIC: 1086.8
##
## Number of Fisher Scoring iterations: 2

data$Country <- relevel(data$Country, ref= "GHA")
model1<-glm((Prev_TvTsg) ~ Country,data=data, family=gaussian())
summary(model1)

##
## Call:
## glm(formula = (Prev_TvTsg) ~ Country, family = gaussian(), data = data)
##
## Deviance Residuals:
## Min 1Q Median 3Q Max
## -6.536 -0.104 0.000 0.000 60.134
##
## Coefficients:
## Estimate Std. Error t value Pr(>|t|)
## (Intercept) 3.413e-15 1.763e+00 0.000 1.00000
## CountryBKF 6.536e+00 2.100e+00 3.112 0.00225 **
## CountryZIM -6.966e-15 2.951e+00 0.000 1.00000
## CountryZAM -4.867e-15 7.686e+00 0.000 1.00000
## CountrySAF -4.669e-15 3.179e+00 0.000 1.00000
## CountryUGA -4.758e-15 3.782e+00 0.000 1.00000
## CountryURT 1.142e+00 3.527e+00 0.324 0.74662
## CountrySWA -9.123e-15 7.686e+00 0.000 1.00000
## CountrySEN -5.532e-15 2.615e+00 0.000 1.00000
## CountryZAI -3.625e-14 7.686e+00 0.000 1.00000
## CountryMOZ 1.000e+00 5.576e+00 0.179 0.85793
## CountryMLI -6.984e-15 2.494e+00 0.000 1.00000
## CountryKEN 1.042e-01 2.788e+00 0.037 0.97025
## CountryETH 4.400e-01 7.686e+00 0.057 0.95443
## CountryGUI -5.822e-15 2.615e+00 0.000 1.00000
## ---
## Signif. codes: 0 '***' 0.001 '**' 0.01 '*' 0.05 '.' 0.1 ' ' 1
##
## (Dispersion parameter for gaussian family taken to be 55.96374)
##
## Null deviance: 9193.0 on 155 degrees of freedom
## Residual deviance: 7890.9 on 141 degrees of freedom
## AIC: 1086.8
##
## Number of Fisher Scoring iterations: 2

data$Country <- relevel(data$Country, ref= "GUI")
model1<-glm((Prev_TvTsg) ~ Country,data=data, family=gaussian())
summary(model1)

##
## Call:
## glm(formula = (Prev_TvTsg) ~ Country, family = gaussian(), data = data)
##
## Deviance Residuals:
## Min 1Q Median 3Q Max
## -6.536 -0.104 0.000 0.000 60.134
##
## Coefficients:
## Estimate Std. Error t value Pr(>|t|)
## (Intercept) 2.635e-15 1.932e+00 0.000 1.00000
## CountryGHA -3.116e-15 2.615e+00 0.000 1.00000
## CountryBKF 6.536e+00 2.243e+00 2.914 0.00416 **
## CountryZIM -2.345e-15 3.054e+00 0.000 1.00000
## CountryZAM -2.158e-15 7.726e+00 0.000 1.00000
## CountrySAF -1.999e-15 3.275e+00 0.000 1.00000
## CountryUGA -3.262e-15 3.863e+00 0.000 1.00000
## CountryURT 1.142e+00 3.614e+00 0.316 0.75252
## CountrySWA -5.779e-15 7.726e+00 0.000 1.00000
## CountrySEN -3.424e-15 2.732e+00 0.000 1.00000
## CountryZAI 1.445e-14 7.726e+00 0.000 1.00000
## CountryMOZ 1.000e+00 5.631e+00 0.178 0.85931
## CountryMLI 5.031e-16 2.615e+00 0.000 1.00000
## CountryKEN 1.042e-01 2.897e+00 0.036 0.97137
## CountryETH 4.400e-01 7.726e+00 0.057 0.95467
## ---
## Signif. codes: 0 '***' 0.001 '**' 0.01 '*' 0.05 '.' 0.1 ' ' 1
##
## (Dispersion parameter for gaussian family taken to be 55.96374)
##
## Null deviance: 9193.0 on 155 degrees of freedom
## Residual deviance: 7890.9 on 141 degrees of freedom
## AIC: 1086.8
##
## Number of Fisher Scoring iterations: 2

data$Country <- relevel(data$Country, ref= "ETH")
model1<-glm((Prev_TvTsg) ~ Country,data=data, family=gaussian())
summary(model1)

##
## Call:
## glm(formula = (Prev_TvTsg) ~ Country, family = gaussian(), data = data)
##
## Deviance Residuals:
## Min 1Q Median 3Q Max
## -6.536 -0.104 0.000 0.000 60.134
##
## Coefficients:
## Estimate Std. Error t value Pr(>|t|)
## (Intercept) 0.4400 7.4809 0.059 0.953
## CountryGUI -0.4400 7.7262 -0.057 0.955
## CountryGHA -0.4400 7.6859 -0.057 0.954
## CountryBKF 6.0960 7.5674 0.806 0.422
## CountryZIM -0.4400 7.8460 -0.056 0.955
## CountryZAM -0.4400 10.5796 -0.042 0.967
## CountrySAF -0.4400 7.9347 -0.055 0.956
## CountryUGA -0.4400 8.1949 -0.054 0.957
## CountryURT 0.7017 8.0803 0.087 0.931
## CountrySWA -0.4400 10.5796 -0.042 0.967
## CountrySEN -0.4400 7.7262 -0.057 0.955
## CountryZAI -0.4400 10.5796 -0.042 0.967
## CountryMOZ 0.5600 9.1622 0.061 0.951
## CountryMLI -0.4400 7.6859 -0.057 0.954
## CountryKEN -0.3358 7.7864 -0.043 0.966
##
## (Dispersion parameter for gaussian family taken to be 55.96374)
##
## Null deviance: 9193.0 on 155 degrees of freedom
## Residual deviance: 7890.9 on 141 degrees of freedom
## AIC: 1086.8
##
## Number of Fisher Scoring iterations: 2

data$Country <- relevel(data$Country, ref= "KEN")
model1<-glm((Prev_TvTsg) ~ Country,data=data, family=gaussian())
summary(model1)

##
## Call:
## glm(formula = (Prev_TvTsg) ~ Country, family = gaussian(), data = data)
##
## Deviance Residuals:
## Min 1Q Median 3Q Max
## -6.536 -0.104 0.000 0.000 60.134
##
## Coefficients:
## Estimate Std. Error t value Pr(>|t|)
## (Intercept) 0.1042 2.1595 0.048 0.9616
## CountryETH 0.3358 7.7864 0.043 0.9657
## CountryGUI -0.1042 2.8973 -0.036 0.9714
## CountryGHA -0.1042 2.7880 -0.037 0.9702
## CountryBKF 6.4319 2.4424 2.633 0.0094 **
## CountryZIM -0.1042 3.2031 -0.033 0.9741
## CountryZAM -0.1042 7.7864 -0.013 0.9893
## CountrySAF -0.1042 3.4145 -0.031 0.9757
## CountryUGA -0.1042 3.9820 -0.026 0.9792
## CountryURT 1.0375 3.7404 0.277 0.7819
## CountrySWA -0.1042 7.7864 -0.013 0.9893
## CountrySEN -0.1042 2.8973 -0.036 0.9714
## CountryZAI -0.1042 7.7864 -0.013 0.9893
## CountryMOZ 0.8958 5.7136 0.157 0.8756
## CountryMLI -0.1042 2.7880 -0.037 0.9702
## ---
## Signif. codes: 0 '***' 0.001 '**' 0.01 '*' 0.05 '.' 0.1 ' ' 1
##
## (Dispersion parameter for gaussian family taken to be 55.96374)
##
## Null deviance: 9193.0 on 155 degrees of freedom
## Residual deviance: 7890.9 on 141 degrees of freedom
## AIC: 1086.8
##
## Number of Fisher Scoring iterations: 2

data$Country <- relevel(data$Country, ref= "MLI")
model1<-glm((Prev_TvTsg) ~ Country,data=data, family=gaussian())
summary(model1)

##
## Call:
## glm(formula = (Prev_TvTsg) ~ Country, family = gaussian(), data = data)
##
## Deviance Residuals:
## Min 1Q Median 3Q Max
## -6.536 -0.104 0.000 0.000 60.134
##
## Coefficients:
## Estimate Std. Error t value Pr(>|t|)
## (Intercept) -2.697e-15 1.763e+00 0.000 1.00000
## CountryKEN 1.042e-01 2.788e+00 0.037 0.97025
## CountryETH 4.400e-01 7.686e+00 0.057 0.95443
## CountryGUI 6.453e-15 2.615e+00 0.000 1.00000
## CountryGHA 4.517e-15 2.494e+00 0.000 1.00000
## CountryBKF 6.536e+00 2.100e+00 3.112 0.00225 **
## CountryZIM 7.330e-15 2.951e+00 0.000 1.00000
## CountryZAM 7.386e-15 7.686e+00 0.000 1.00000
## CountrySAF 9.513e-15 3.179e+00 0.000 1.00000
## CountryUGA 5.246e-15 3.782e+00 0.000 1.00000
## CountryURT 1.142e+00 3.527e+00 0.324 0.74662
## CountrySWA 5.182e-15 7.686e+00 0.000 1.00000
## CountrySEN 5.356e-15 2.615e+00 0.000 1.00000
## CountryZAI 5.674e-15 7.686e+00 0.000 1.00000
## CountryMOZ 1.000e+00 5.576e+00 0.179 0.85793
## ---
## Signif. codes: 0 '***' 0.001 '**' 0.01 '*' 0.05 '.' 0.1 ' ' 1
##
## (Dispersion parameter for gaussian family taken to be 55.96374)
##
## Null deviance: 9193.0 on 155 degrees of freedom
## Residual deviance: 7890.9 on 141 degrees of freedom
## AIC: 1086.8
##
## Number of Fisher Scoring iterations: 2

data$Country <- relevel(data$Country, ref= "MOZ")
model1<-glm((Prev_TvTsg) ~ Country,data=data, family=gaussian())
summary(model1)

##
## Call:
## glm(formula = (Prev_TvTsg) ~ Country, family = gaussian(), data = data)
##
## Deviance Residuals:
## Min 1Q Median 3Q Max
## -6.536 -0.104 0.000 0.000 60.134
##
## Coefficients:
## Estimate Std. Error t value Pr(>|t|)
## (Intercept) 1.0000 5.2898 0.189 0.850
## CountryMLI -1.0000 5.5759 -0.179 0.858
## CountryKEN -0.8958 5.7136 -0.157 0.876
## CountryETH -0.5600 9.1622 -0.061 0.951
## CountryGUI -1.0000 5.6314 -0.178 0.859
## CountryGHA -1.0000 5.5759 -0.179 0.858
## CountryBKF 5.5360 5.4114 1.023 0.308
## CountryZIM -1.0000 5.7947 -0.173 0.863
## CountryZAM -1.0000 9.1622 -0.109 0.913
## CountrySAF -1.0000 5.9142 -0.169 0.866
## CountryUGA -1.0000 6.2590 -0.160 0.873
## CountryURT 0.1417 6.1081 0.023 0.982
## CountrySWA -1.0000 9.1622 -0.109 0.913
## CountrySEN -1.0000 5.6314 -0.178 0.859
## CountryZAI -1.0000 9.1622 -0.109 0.913
##
## (Dispersion parameter for gaussian family taken to be 55.96374)
##
## Null deviance: 9193.0 on 155 degrees of freedom
## Residual deviance: 7890.9 on 141 degrees of freedom
## AIC: 1086.8
##
## Number of Fisher Scoring iterations: 2

data$Country <- relevel(data$Country, ref= "ZAI")
model1<-glm((Prev_TvTsg) ~ Country,data=data, family=gaussian())
summary(model1)

##
## Call:
## glm(formula = (Prev_TvTsg) ~ Country, family = gaussian(), data = data)
##
## Deviance Residuals:
## Min 1Q Median 3Q Max
## -6.536 -0.104 0.000 0.000 60.134
##
## Coefficients:
## Estimate Std. Error t value Pr(>|t|)
## (Intercept) 6.801e-14 7.481e+00 0.000 1.000
## CountryMOZ 1.000e+00 9.162e+00 0.109 0.913
## CountryMLI -6.869e-14 7.686e+00 0.000 1.000
## CountryKEN 1.042e-01 7.786e+00 0.013 0.989
## CountryETH 4.400e-01 1.058e+01 0.042 0.967
## CountryGUI -6.621e-14 7.726e+00 0.000 1.000
## CountryGHA -6.834e-14 7.686e+00 0.000 1.000
## CountryBKF 6.536e+00 7.567e+00 0.864 0.389
## CountryZIM -6.617e-14 7.846e+00 0.000 1.000
## CountryZAM -1.372e-13 1.058e+01 0.000 1.000
## CountrySAF -6.079e-14 7.935e+00 0.000 1.000
## CountryUGA -6.251e-14 8.195e+00 0.000 1.000
## CountryURT 1.142e+00 8.080e+00 0.141 0.888
## CountrySWA -6.557e-14 1.058e+01 0.000 1.000
## CountrySEN -6.897e-14 7.726e+00 0.000 1.000
##
## (Dispersion parameter for gaussian family taken to be 55.96374)
##
## Null deviance: 9193.0 on 155 degrees of freedom
## Residual deviance: 7890.9 on 141 degrees of freedom
## AIC: 1086.8
##
## Number of Fisher Scoring iterations: 2

data$Country <- relevel(data$Country, ref= "SEN")
model1<-glm((Prev_TvTsg) ~ Country,data=data, family=gaussian())
summary(model1)

##
## Call:
## glm(formula = (Prev_TvTsg) ~ Country, family = gaussian(), data = data)
##
## Deviance Residuals:
## Min 1Q Median 3Q Max
## -6.536 -0.104 0.000 0.000 60.134
##
## Coefficients:
## Estimate Std. Error t value Pr(>|t|)
## (Intercept) -5.635e-15 1.932e+00 0.000 1.00000
## CountryZAI -4.202e-14 7.726e+00 0.000 1.00000
## CountryMOZ 1.000e+00 5.631e+00 0.178 0.85931
## CountryMLI 8.876e-15 2.615e+00 0.000 1.00000
## CountryKEN 1.042e-01 2.897e+00 0.036 0.97137
## CountryETH 4.400e-01 7.726e+00 0.057 0.95467
## CountryGUI 7.783e-15 2.732e+00 0.000 1.00000
## CountryGHA 5.509e-15 2.615e+00 0.000 1.00000
## CountryBKF 6.536e+00 2.243e+00 2.914 0.00416 **
## CountryZIM 8.371e-15 3.054e+00 0.000 1.00000
## CountryZAM -1.819e-14 7.726e+00 0.000 1.00000
## CountrySAF 1.109e-16 3.275e+00 0.000 1.00000
## CountryUGA 1.001e-14 3.863e+00 0.000 1.00000
## CountryURT 1.142e+00 3.614e+00 0.316 0.75252
## CountrySWA 5.275e-15 7.726e+00 0.000 1.00000
## ---
## Signif. codes: 0 '***' 0.001 '**' 0.01 '*' 0.05 '.' 0.1 ' ' 1
##
## (Dispersion parameter for gaussian family taken to be 55.96374)
##
## Null deviance: 9193.0 on 155 degrees of freedom
## Residual deviance: 7890.9 on 141 degrees of freedom
## AIC: 1086.8
##
## Number of Fisher Scoring iterations: 2

data$Country <- relevel(data$Country, ref= "SWA")
model1<-glm((Prev_TvTsg) ~ Country,data=data, family=gaussian())
summary(model1)

##
## Call:
## glm(formula = (Prev_TvTsg) ~ Country, family = gaussian(), data = data)
##
## Deviance Residuals:
## Min 1Q Median 3Q Max
## -6.536 -0.104 0.000 0.000 60.134
##
## Coefficients:
## Estimate Std. Error t value Pr(>|t|)
## (Intercept) 5.742e-14 7.481e+00 0.000 1.000
## CountrySEN -4.577e-14 7.726e+00 0.000 1.000
## CountryZAI -4.629e-14 1.058e+01 0.000 1.000
## CountryMOZ 1.000e+00 9.162e+00 0.109 0.913
## CountryMLI -5.995e-14 7.686e+00 0.000 1.000
## CountryKEN 1.042e-01 7.786e+00 0.013 0.989
## CountryETH 4.400e-01 1.058e+01 0.042 0.967
## CountryGUI -5.630e-14 7.726e+00 0.000 1.000
## CountryGHA -6.022e-14 7.686e+00 0.000 1.000
## CountryBKF 6.536e+00 7.567e+00 0.864 0.389
## CountryZIM -5.496e-14 7.846e+00 0.000 1.000
## CountryZAM -5.324e-14 1.058e+01 0.000 1.000
## CountrySAF -4.672e-14 7.935e+00 0.000 1.000
## CountryUGA -5.084e-14 8.195e+00 0.000 1.000
## CountryURT 1.142e+00 8.080e+00 0.141 0.888
##
## (Dispersion parameter for gaussian family taken to be 55.96374)
##
## Null deviance: 9193.0 on 155 degrees of freedom
## Residual deviance: 7890.9 on 141 degrees of freedom
## AIC: 1086.8
##
## Number of Fisher Scoring iterations: 2

data$Country <- relevel(data$Country, ref= "URT")
model1<-glm((Prev_TvTsg) ~ Country,data=data, family=gaussian())
summary(model1)

##
## Call:
## glm(formula = (Prev_TvTsg) ~ Country, family = gaussian(), data = data)
##
## Deviance Residuals:
## Min 1Q Median 3Q Max
## -6.536 -0.104 0.000 0.000 60.134
##
## Coefficients:
## Estimate Std. Error t value Pr(>|t|)
## (Intercept) 1.1417 3.0541 0.374 0.709
## CountrySWA -1.1417 8.0803 -0.141 0.888
## CountrySEN -1.1417 3.6136 -0.316 0.753
## CountryZAI -1.1417 8.0803 -0.141 0.888
## CountryMOZ -0.1417 6.1081 -0.023 0.982
## CountryMLI -1.1417 3.5265 -0.324 0.747
## CountryKEN -1.0375 3.7404 -0.277 0.782
## CountryETH -0.7017 8.0803 -0.087 0.931
## CountryGUI -1.1417 3.6136 -0.316 0.753
## CountryGHA -1.1417 3.5265 -0.324 0.747
## CountryBKF 5.3944 3.2602 1.655 0.100
## CountryZIM -1.1417 3.8631 -0.296 0.768
## CountryZAM -1.1417 8.0803 -0.141 0.888
## CountrySAF -1.1417 4.0401 -0.283 0.778
## CountryUGA -1.1417 4.5299 -0.252 0.801
##
## (Dispersion parameter for gaussian family taken to be 55.96374)
##
## Null deviance: 9193.0 on 155 degrees of freedom
## Residual deviance: 7890.9 on 141 degrees of freedom
## AIC: 1086.8
##
## Number of Fisher Scoring iterations: 2

data$Country <- relevel(data$Country, ref= "UGA")
model1<-glm((Prev_TvTsg) ~ Country,data=data, family=gaussian())
summary(model1)

##
## Call:
## glm(formula = (Prev_TvTsg) ~ Country, family = gaussian(), data = data)
##
## Deviance Residuals:
## Min 1Q Median 3Q Max
## -6.536 -0.104 0.000 0.000 60.134
##
## Coefficients:
## Estimate Std. Error t value Pr(>|t|)
## (Intercept) 2.818e-14 3.346e+00 0.000 1.0000
## CountryURT 1.142e+00 4.530e+00 0.252 0.8014
## CountrySWA -2.422e-14 8.195e+00 0.000 1.0000
## CountrySEN -2.735e-14 3.863e+00 0.000 1.0000
## CountryZAI -3.795e-14 8.195e+00 0.000 1.0000
## CountryMOZ 1.000e+00 6.259e+00 0.160 0.8733
## CountryMLI -2.995e-14 3.782e+00 0.000 1.0000
## CountryKEN 1.042e-01 3.982e+00 0.026 0.9792
## CountryETH 4.400e-01 8.195e+00 0.054 0.9573
## CountryGUI -2.101e-14 3.863e+00 0.000 1.0000
## CountryGHA -2.641e-14 3.782e+00 0.000 1.0000
## CountryBKF 6.536e+00 3.535e+00 1.849 0.0665 .
## CountryZIM -2.864e-14 4.097e+00 0.000 1.0000
## CountryZAM -1.832e-15 8.195e+00 0.000 1.0000
## CountrySAF -2.544e-14 4.265e+00 0.000 1.0000
## ---
## Signif. codes: 0 '***' 0.001 '**' 0.01 '*' 0.05 '.' 0.1 ' ' 1
##
## (Dispersion parameter for gaussian family taken to be 55.96374)
##
## Null deviance: 9193.0 on 155 degrees of freedom
## Residual deviance: 7890.9 on 141 degrees of freedom
## AIC: 1086.8
##
## Number of Fisher Scoring iterations: 2

data$Country <- relevel(data$Country, ref= "SAF")
model1<-glm((Prev_TvTsg) ~ Country,data=data, family=gaussian())
summary(model1)

##
## Call:
## glm(formula = (Prev_TvTsg) ~ Country, family = gaussian(), data = data)
##
## Deviance Residuals:
## Min 1Q Median 3Q Max
## -6.536 -0.104 0.000 0.000 60.134
##
## Coefficients:
## Estimate Std. Error t value Pr(>|t|)
## (Intercept) 4.031e-15 2.645e+00 0.000 1.0000
## CountryUGA 1.815e-14 4.265e+00 0.000 1.0000
## CountryURT 1.142e+00 4.040e+00 0.283 0.7779
## CountrySWA -1.675e-14 7.935e+00 0.000 1.0000
## CountrySEN -5.181e-15 3.275e+00 0.000 1.0000
## CountryZAI -4.942e-15 7.935e+00 0.000 1.0000
## CountryMOZ 1.000e+00 5.914e+00 0.169 0.8660
## CountryMLI -5.386e-15 3.179e+00 0.000 1.0000
## CountryKEN 1.042e-01 3.415e+00 0.031 0.9757
## CountryETH 4.400e-01 7.935e+00 0.055 0.9559
## CountryGUI 8.498e-15 3.275e+00 0.000 1.0000
## CountryGHA -6.658e-15 3.179e+00 0.000 1.0000
## CountryBKF 6.536e+00 2.880e+00 2.269 0.0248 *
## CountryZIM -1.167e-14 3.548e+00 0.000 1.0000
## CountryZAM -2.237e-15 7.935e+00 0.000 1.0000
## ---
## Signif. codes: 0 '***' 0.001 '**' 0.01 '*' 0.05 '.' 0.1 ' ' 1
##
## (Dispersion parameter for gaussian family taken to be 55.96374)
##
## Null deviance: 9193.0 on 155 degrees of freedom
## Residual deviance: 7890.9 on 141 degrees of freedom
## AIC: 1086.8
##
## Number of Fisher Scoring iterations: 2

data$Country <- relevel(data$Country, ref= "ZAM")
model1<-glm((Prev_TvTsg) ~ Country,data=data, family=gaussian())
summary(model1)

##
## Call:
## glm(formula = (Prev_TvTsg) ~ Country, family = gaussian(), data = data)
##
## Deviance Residuals:
## Min 1Q Median 3Q Max
## -6.536 -0.104 0.000 0.000 60.134
##
## Coefficients:
## Estimate Std. Error t value Pr(>|t|)
## (Intercept) 2.302e-13 7.481e+00 0.000 1.000
## CountrySAF -2.087e-13 7.935e+00 0.000 1.000
## CountryUGA -2.168e-13 8.195e+00 0.000 1.000
## CountryURT 1.142e+00 8.080e+00 0.141 0.888
## CountrySWA -2.309e-13 1.058e+01 0.000 1.000
## CountrySEN -2.346e-13 7.726e+00 0.000 1.000
## CountryZAI -2.390e-13 1.058e+01 0.000 1.000
## CountryMOZ 1.000e+00 9.162e+00 0.109 0.913
## CountryMLI -2.276e-13 7.686e+00 0.000 1.000
## CountryKEN 1.042e-01 7.786e+00 0.013 0.989
## CountryETH 4.400e-01 1.058e+01 0.042 0.967
## CountryGUI -2.317e-13 7.726e+00 0.000 1.000
## CountryGHA -2.330e-13 7.686e+00 0.000 1.000
## CountryBKF 6.536e+00 7.567e+00 0.864 0.389
## CountryZIM -2.336e-13 7.846e+00 0.000 1.000
##
## (Dispersion parameter for gaussian family taken to be 55.96374)
##
## Null deviance: 9193.0 on 155 degrees of freedom
## Residual deviance: 7890.9 on 141 degrees of freedom
## AIC: 1086.8
##
## Number of Fisher Scoring iterations: 2

data$Country <- relevel(data$Country, ref= "ZIM")
model1<-glm((Prev_TvTsg) ~ Country,data=data, family=gaussian())
summary(model1)

##
## Call:
## glm(formula = (Prev_TvTsg) ~ Country, family = gaussian(), data = data)
##
## Deviance Residuals:
## Min 1Q Median 3Q Max
## -6.536 -0.104 0.000 0.000 60.134
##
## Coefficients:
## Estimate Std. Error t value Pr(>|t|)
## (Intercept) -2.773e-15 2.366e+00 0.000 1.000
## CountryZAM -6.818e-14 7.846e+00 0.000 1.000
## CountrySAF -1.283e-14 3.548e+00 0.000 1.000
## CountryUGA -7.956e-16 4.097e+00 0.000 1.000
## CountryURT 1.142e+00 3.863e+00 0.296 0.768
## CountrySWA 7.575e-15 7.846e+00 0.000 1.000
## CountrySEN 2.589e-15 3.054e+00 0.000 1.000
## CountryZAI -2.430e-14 7.846e+00 0.000 1.000
## CountryMOZ 1.000e+00 5.795e+00 0.173 0.863
## CountryMLI 1.225e-14 2.951e+00 0.000 1.000
## CountryKEN 1.042e-01 3.203e+00 0.033 0.974
## CountryETH 4.400e-01 7.846e+00 0.056 0.955
## CountryGUI -3.846e-15 3.054e+00 0.000 1.000
## CountryGHA 8.723e-15 2.951e+00 0.000 1.000
## CountryBKF 6.536e+00 2.626e+00 2.489 0.014 *
## ---
## Signif. codes: 0 '***' 0.001 '**' 0.01 '*' 0.05 '.' 0.1 ' ' 1
##
## (Dispersion parameter for gaussian family taken to be 55.96374)
##
## Null deviance: 9193.0 on 155 degrees of freedom
## Residual deviance: 7890.9 on 141 degrees of freedom
## AIC: 1086.8
##
## Number of Fisher Scoring iterations: 2

#======== Glm TzTsg per country
data$Country <- relevel(data$Country, ref= "BKF")
model1<-glm((Prev_TzTsg) ~ Country,data=data, family=gaussian())
summary(model1)

##
## Call:
## glm(formula = (Prev_TzTsg) ~ Country, family = gaussian(), data = data)
##
## Deviance Residuals:
## Min 1Q Median 3Q Max
## -1.8402 -0.8751 0.0000 0.0000 31.4898
##
## Coefficients:
## Estimate Std. Error t value Pr(>|t|)
## (Intercept) 1.8402 0.5987 3.074 0.00254 **
## CountryZIM -1.8402 1.3784 -1.335 0.18401
## CountryZAM -1.8402 3.9716 -0.463 0.64383
## CountrySAF -1.8402 1.5117 -1.217 0.22552
## CountryUGA -1.8402 1.8551 -0.992 0.32291
## CountryURT -1.2869 1.7110 -0.752 0.45323
## CountrySWA -1.8402 3.9716 -0.463 0.64383
## CountrySEN -1.8402 1.1773 -1.563 0.12029
## CountryZAI -1.8402 3.9716 -0.463 0.64383
## CountryMOZ -1.8402 2.8401 -0.648 0.51807
## CountryMLI -1.8402 1.1022 -1.670 0.09722 .
## CountryKEN -1.5652 1.2818 -1.221 0.22408
## CountryETH -1.1902 3.9716 -0.300 0.76486
## CountryGUI -1.8402 1.1773 -1.563 0.12029
## CountryGHA -1.8402 1.1022 -1.670 0.09722 .
## ---
## Signif. codes: 0 '***' 0.001 '**' 0.01 '*' 0.05 '.' 0.1 ' ' 1
##
## (Dispersion parameter for gaussian family taken to be 15.41486)
##
## Null deviance: 2274.4 on 155 degrees of freedom
## Residual deviance: 2173.5 on 141 degrees of freedom
## AIC: 885.65
##
## Number of Fisher Scoring iterations: 2

data$Country <- relevel(data$Country, ref= "GHA")
model1<-glm((Prev_TzTsg) ~ Country,data=data, family=gaussian())
summary(model1)

##
## Call:
## glm(formula = (Prev_TzTsg) ~ Country, family = gaussian(), data = data)
##
## Deviance Residuals:
## Min 1Q Median 3Q Max
## -1.8402 -0.8751 0.0000 0.0000 31.4898
##
## Coefficients:
## Estimate Std. Error t value Pr(>|t|)
## (Intercept) 1.778e-15 9.254e-01 0.000 1.0000
## CountryBKF 1.840e+00 1.102e+00 1.670 0.0972 .
## CountryZIM 2.017e-15 1.549e+00 0.000 1.0000
## CountryZAM -1.941e-15 4.034e+00 0.000 1.0000
## CountrySAF -1.194e-15 1.668e+00 0.000 1.0000
## CountryUGA -1.697e-15 1.985e+00 0.000 1.0000
## CountryURT 5.533e-01 1.851e+00 0.299 0.7654
## CountrySWA -2.506e-15 4.034e+00 0.000 1.0000
## CountrySEN -1.966e-15 1.373e+00 0.000 1.0000
## CountryZAI -1.072e-15 4.034e+00 0.000 1.0000
## CountryMOZ 1.358e-16 2.926e+00 0.000 1.0000
## CountryMLI -3.025e-15 1.309e+00 0.000 1.0000
## CountryKEN 2.750e-01 1.463e+00 0.188 0.8512
## CountryETH 6.500e-01 4.034e+00 0.161 0.8722
## CountryGUI -1.650e-15 1.373e+00 0.000 1.0000
## ---
## Signif. codes: 0 '***' 0.001 '**' 0.01 '*' 0.05 '.' 0.1 ' ' 1
##
## (Dispersion parameter for gaussian family taken to be 15.41486)
##
## Null deviance: 2274.4 on 155 degrees of freedom
## Residual deviance: 2173.5 on 141 degrees of freedom
## AIC: 885.65
##
## Number of Fisher Scoring iterations: 2

data$Country <- relevel(data$Country, ref= "GUI")
model1<-glm((Prev_TzTsg) ~ Country,data=data, family=gaussian())
summary(model1)

##
## Call:
## glm(formula = (Prev_TzTsg) ~ Country, family = gaussian(), data = data)
##
## Deviance Residuals:
## Min 1Q Median 3Q Max
## -1.8402 -0.8751 0.0000 0.0000 31.4898
##
## Coefficients:
## Estimate Std. Error t value Pr(>|t|)
## (Intercept) 7.092e-16 1.014e+00 0.000 1.000
## CountryGHA -2.448e-15 1.373e+00 0.000 1.000
## CountryBKF 1.840e+00 1.177e+00 1.563 0.120
## CountryZIM -2.792e-16 1.603e+00 0.000 1.000
## CountryZAM -2.010e-16 4.055e+00 0.000 1.000
## CountrySAF 7.995e-16 1.719e+00 0.000 1.000
## CountryUGA -1.490e-15 2.027e+00 0.000 1.000
## CountryURT 5.533e-01 1.897e+00 0.292 0.771
## CountrySWA 1.732e-16 4.055e+00 0.000 1.000
## CountrySEN 5.485e-16 1.434e+00 0.000 1.000
## CountryZAI -1.067e-15 4.055e+00 0.000 1.000
## CountryMOZ -8.111e-16 2.956e+00 0.000 1.000
## CountryMLI -5.870e-16 1.373e+00 0.000 1.000
## CountryKEN 2.750e-01 1.521e+00 0.181 0.857
## CountryETH 6.500e-01 4.055e+00 0.160 0.873
##
## (Dispersion parameter for gaussian family taken to be 15.41486)
##
## Null deviance: 2274.4 on 155 degrees of freedom
## Residual deviance: 2173.5 on 141 degrees of freedom
## AIC: 885.65
##
## Number of Fisher Scoring iterations: 2

data$Country <- relevel(data$Country, ref= "ETH")
model1<-glm((Prev_TzTsg) ~ Country,data=data, family=gaussian())
summary(model1)

##
## Call:
## glm(formula = (Prev_TzTsg) ~ Country, family = gaussian(), data = data)
##
## Deviance Residuals:
## Min 1Q Median 3Q Max
## -1.8402 -0.8751 0.0000 0.0000 31.4898
##
## Coefficients:
## Estimate Std. Error t value Pr(>|t|)
## (Intercept) 0.65000 3.92618 0.166 0.869
## CountryGUI -0.65000 4.05494 -0.160 0.873
## CountryGHA -0.65000 4.03376 -0.161 0.872
## CountryBKF 1.19023 3.97157 0.300 0.765
## CountryZIM -0.65000 4.11781 -0.158 0.875
## CountryZAM -0.65000 5.55245 -0.117 0.907
## CountrySAF -0.65000 4.16434 -0.156 0.876
## CountryUGA -0.65000 4.30091 -0.151 0.880
## CountryURT -0.09667 4.24076 -0.023 0.982
## CountrySWA -0.65000 5.55245 -0.117 0.907
## CountrySEN -0.65000 4.05494 -0.160 0.873
## CountryZAI -0.65000 5.55245 -0.117 0.907
## CountryMOZ -0.65000 4.80856 -0.135 0.893
## CountryMLI -0.65000 4.03376 -0.161 0.872
## CountryKEN -0.37500 4.08649 -0.092 0.927
##
## (Dispersion parameter for gaussian family taken to be 15.41486)
##
## Null deviance: 2274.4 on 155 degrees of freedom
## Residual deviance: 2173.5 on 141 degrees of freedom
## AIC: 885.65
##
## Number of Fisher Scoring iterations: 2

data$Country <- relevel(data$Country, ref= "KEN")
model1<-glm((Prev_TzTsg) ~ Country,data=data, family=gaussian())
summary(model1)

##
## Call:
## glm(formula = (Prev_TzTsg) ~ Country, family = gaussian(), data = data)
##
[truncated: 343,606 more chars]
