# Supplementary material for: Prevalence of Trypanosoma and Sodalis in wild populations of tsetse flies and their impact on sterile insect technique programmes for tsetse eradication
Source: Sci Rep. 2022 Feb 28;12:3322. doi: 10.1038/s41598-022-06699-2 (PMC8885713; doi:10.1038/s41598-022-06699-2)
Supplement: Supplementary file 4 — Supplementary Information 4. [file 41598_2022_6699_MOESM4_ESM.docx]

**Supplementary File Legends**

**Supplementary Figure 1**. **Prevalence of the *Sodalis* and *Trypanosoma* (single and multiple) infection per country-species.** Prevalence data where square root transformed and averaged based by country-species and the matrix display was conducted in PRIMER version 7+ software. Country abbreviations follow the UNDP list of country codes <https://web.archive.org/web/20060713221355/http://refgat.undp.org/genericlist.cfm?entid=82&pagenumber=1&requesttimeout=360> as following: BKF: Burkina Faso; ETH: Ethiopia; GHA: Ghana; GUI: Guinea; KEN: Kenya; MLI: Mali; MOZ: Mozambique; SAF: South Africa; SWA: Eswatini; ZAI: Democratic Republic of the Congo; ZAM: Zambia; ZIM: Zimbabwe. Tsetse species was abbreviated as following: Ga: *Glossina austeni*; Gb: *G. brevipalpis*; Gff: *G. fuscipes fuscipes*, Gmm: *G. morsitans morsitans*; Gmsm: *G. m. submorsitans*; Gpg: *G. palpalis gambiensis*; Gpp: *G. p. palpalis*. Sod: Sodalis, Tc: *Trypanosoma. congolense* savannah, *T. congolense* Kilifi; *T. congolense* forest, Tsg: *T. simiae*; *T. simiae* Tsavo; *T. godfreyi*, Tv: *T. vivax,* Tz: T*. b. brucei, T. b. gambiense, T. b. rhodesiense.*

**Supplementary Figure 2**. **Correlation between** ***Trypanosoma* and *Sodalis* infection density in different tsetse species (A) and different infection type (B)**. Coloured circles indicate the normalised *Sodalis* and Trypanosome density.
