# Supplementary material for: Prevalence of Trypanosoma and Sodalis in wild populations of tsetse flies and their impact on sterile insect technique programmes for tsetse eradication
Source: Sci Rep. 2022 Feb 28;12:3322. doi: 10.1038/s41598-022-06699-2 (PMC8885713; doi:10.1038/s41598-022-06699-2)
Supplement: Supplementary file 5 — Supplementary Information 5. [file 41598_2022_6699_MOESM5_ESM.docx]

**Supplementary Table 1:** Prevalence of Trypanosome single and multiple infection in tsetse samples analyzed per country

| **Region** | **Country** | **Prevalence of trypanaosome species/subspecies and mixed infection (%)*** | | | | | | | | | | |
| --- | --- | --- | --- | --- | --- | --- | --- | --- | --- | --- | --- | --- |
|  |  | **Tc** | **Tv** | **Tz** | **Tsg** | **TcTv** | **TcTz** | **TcTsg** | **TvTz** | **TvTsg** | **TzTsg** | **TcTvTz** |
| East and south Africa | Ethiopia | 6.10^ab^ | 0.65 ^ab^ | 1.09 ^a^ | 10.46 ^ab^ | 0 ^ab^ | 0 ^ab^ | 0.65 ^ab^ | 0 ^ab^ | 0.44 ^ab^ | 0.65 | 0.00 |
|  | Kenya | 13.00 ^b^ | 5.65 ^ac^ | 0.60 ^a^ | 15.87 ^ab^ | 2.08 ^b^ | 0.50 ^a^ | 6.05 ^b^ | 0.20 ^a^ | 0.10 ^b^ | 0.40 | 0.00 |
|  | Democratic R. of Congo | 0.00 ^ab^ | 2.86 ^ab^ | 0.00 ^a^ | 0.00 ^ab^ | 0.00 ^ab^ | 0.00 ^ab^ | 0.00 ^ab^ | 0.00 ^ab^ | 0.00 ^ab^ | 0.00 | 0.00 |
|  | Mozambique | 3.00 ^a^ | 1.00 ^acd^ | 0.00 ^a^ | 3.00 ^ab^ | 0.00 ^a^ | 0.00 ^ab^ | 0.00 ^a^ | 0.00 ^a^ | 1.00 ^ab^ | 0.00 | 0.00 |
|  | South Africa | 3.99 ^a^ | 0.57 ^bc^ | 3.04 ^a^ | 7.03 ^ab^ | 0.19 ^a^ | 0.00 ^a^ | 0.38 ^a^ | 0.00 ^a^ | 0.00 ^b^ | 0.00 | 0.00 |
|  | Eswatini | 0.00 ^ab^ | 0.00 ^ab^ | 0.00 ^a^ | 0.00 ^ab^ | 0.00 ^ab^ | 0.00 ^ab^ | 0.00 ^ab^ | 0.00 ^ab^ | 0.00 ^ab^ | 0.00 | 0.00 |
|  | Tanzania | **14.20** ^b^ | 2.07 ^ab^ | 1.18 ^a^ | 10.95 ^ab^ | 0.00 ^a^ | 0.30 ^a^ | 6.21 ^b^ | 0.30 ^a^ | 1.78 ^ab^ | 0.89 | 0.00 |
|  | Uganda | 2.86 ^b^ | 0.95 ^ab^ | 0.95 ^a^ | 3.81 ^ab^ | 0.00 ^a^ | 0.00 ^a^ | 0.48 ^a^ | 0.00 ^a^ | 0.00 ^ab^ | 0.00 | 0.00 |
|  | Zambia | 4.76 ^b^ | 0.48 ^abd^ | 0.95 ^a^ | **30.95** ^ab^ | 0.00 ^ab^ | 0.00 ^ab^ | **9.05** ^b^ | 0.00 ^ab^ | 0.00 ^ab^ | 0.00 | 0.00 |
|  | Zimbabwe | 7.11 ^b^ | 0.47 ^bc^ | 1.90 ^a^ | 39.81 ^ab^ | 0.00 ^a^ | 0.00 ^a^ | 4.27 ^a^ | 0.00 ^a^ | 0.00 ^b^ | 0.00 | 0.00 |
|  | **Subtotal (Average%)** | **8.38** | **2.43** | **1.25** | **14.13** | **0.70** | **0.19** | **3.71** | **0.10** | **0.32** | **0.32** | **0.00** |
| West Africa | Burkina Faso | 2.55 ^a^ | 12.14 ^a^ | 2.68 ^a^ | 1.41 ^ac^ | 0.26 ^a^ | 0.53 ^a^ | 0.04 ^a^ | 0.70 ^a^ | 1.19 ^a^ | 0.26 | 0.09 |
|  | Ghana | 1.71 ^a^ | **14.10** ^a^ | **19.66** ^b^ | 0.00 ^ac^ | 0.00 ^a^ | **10.68** ^b^ | 0.00 ^a^ | **12.39** ^b^ | 0.00 ^b^ | 0.00 | 1.71 |
|  | Guinea | 0.00 ^a^ | 2.23 ^bc^ | 0.00 ^a^ | 0.00 ^ac^ | 0.00 ^a^ | 0.00 ^a^ | 0.00 ^a^ | 0.00 ^a^ | 0.00 ^b^ | 0.00 | 0.00 |
|  | Mali | 0.27 ^a^ | 5.49 ^ac^ | 1.10 ^a^ | 0.00 ^ac^ | 0.00 ^a^ | 0.00 ^a^ | 0.00 ^a^ | 0.00 ^a^ | 0.00 ^b^ | 0.00 | 0.00 |
|  | Senegal | 0.55 ^a^ | 8.96 ^acd^ | 1.28 ^a^ | 1.83 ^ac^ | 0.00 ^a^ | 0.00 ^a^ | 0.00 ^a^ | 1.65 ^a^ | 0.00 ^b^ | 0.00 | 0.00 |
|  | **Subtotal (Average%)** | **1.77** | **10.37** | **3.16** | **1.13** | **0.16** | **0.99** | **0.03** | **1.45** | **0.72** | **0.16** | **0.16** |
|  | **Total (Average%)** | **4.78** | **6.75** | **2.29** | **7.06** | **0.41** | **0.63** | **1.71** | **0.83** | **0.54** | **0.23** | **0.09** |

* Tc: *T. congolense* savannah, *T. congolense* Kilifi; *T. congolense* forest, Tsg: *T. simiae*; *T. simiae* Tsavo; *T. godfreyi*, Tv: *T. vivax,* Tz: T*. b. brucei, T. b. gambiense, T. b. rhodensiense, T. evansi*,

**Supplementary Table 2:** Prevalence of Trypanosome single and multiple infection in tsetse samples analyzed per tsetse species

| **Species** | **Prevalence of trypanaosome species/subspecies and mixed infection (%)*** | | | | | | | | | | |
| --- | --- | --- | --- | --- | --- | --- | --- | --- | --- | --- | --- |
|  | **Tc** | **Tv** | **Tz** | **Tsg** | **TcTv** | **TcTz** | **TcTsg** | **TvTz** | **TvTsg** | **TzTsg** | **TcTvTz** |
| *G. austeni* | 4.91^a^ | 0.87^a^ | 2.60 ^a^ | 7.23 ^a^ | 0.29 ^a^ | 0.29 ^a^ | 0.58^a^ | 0.00 ^a^ | 0.00 ^a^ | 0.00 | 0.00 ^a^ |
| *G. brevipalpis* | 2.00^a^ | 0.86^ab^ | 2.29 ^a^ | 4.29 ^ab^ | 0.00 ^a^ | 0.00 ^a^ | 0.00 ^a^ | 0.00 ^a^ | 0.29^a^ | 0.00 | 0.00 ^a^ |
| *G. f. fuscipes* | 2.73^ab^ | 4.37^ab^ | 0.55 ^a^ | 7.10 ^ab^ | 1.09 ^ab^ | 0.55 ^ab^ | 0.55^a^ | 0.00 ^ab^ | 0.00 ^a^ | 0.00 | 0.00 ^a^ |
| *G. medicorum* | 9.09^ab^ | 11.69^ab^ | 13.64 ^ab^ | 0.00 ^a^ | 1.30 ^b^ | 1.30 ^ab^ | 0.00 ^a^ | 0.65^ab^ | 0.00 ^a^ | 0.00 | 1.30 ^b^ |
| *G. m. morsitans* | 9.76^ab^ | 0.54^ac^ | 1.08 ^ac^ | 22.76 ^ae^ | 0.00 ^ad^ | 0.00 ^a^ | 5.15^a^ | 0.00 ^a^ | 1.36^a^ | 0.27 | 0.00 ^a^ |
| *G.m. submorsitans* | 5.25^abc^ | 11.37^ab^ | 0.58 ^ac^ | 0.00 ^ad^ | 0.00 ^a^ | 0.87 ^ab^ | 0.00 ^a^ | 0.00 ^ab^ | 0.00 ^a^ | 0.00 | 0.00 ^a^ |
| *G. pallidipes* | 10.68^b^ | 3.20^a^ | 0.87 ^ac^ | 16.54 ^b^ | 1.03 ^ab^ | 0.27 ^a^ | 5.10^b^ | 0.16^a^ | 0.22^a^ | 0.49 | 0.00 ^a^ |
| *G. p. gambiensis* | 0.46^ac^ | 8.86^b^ | 1.89 ^ac^ | 1.94 ^ac^ | 0.00 ^ac^ | 0.09 ^a^ | 0.05^a^ | 1.06^a^ | 1.20^ab^ | 0.28 | 0.00 ^ac^ |
| *G. p. palpalis* | 0.00 ^abc^ | 2.86^ab^ | 0.00 ^abc^ | 0.00 ^abc^ | 0.00 ^abcd^ | 0.00 ^ab^ | 0.00 ^ab^ | 0.00 ^a^ | 0.00 ^a^ | 0.00 | 0.00 ^a^ |
| *G. tachinoides* | 2.25^ac^ | 12.92^bc^ | 4.96 ^b^ | 0.00 ^ac^ | 0.37 ^ae^ | 2.81 ^b^ | 0.00 ^a^ | 2.81^b^ | 0.00 ^ac^ | 0.00 | 0.37 ^ad^ |
| **Total (Average%)** | **4.78** | **6.75** | **2.29** | **7.06** | **0.41** | **0.63** | **1.71** | **0.83** | **0.54** | **0.23** | **0.09** |

* Tc: *T. congolense* savannah, *T. congolense* Kilifi; *T. congolense* forest, Tsg: *T. simiae*; *T. simiae* Tsavo; *T. godfreyi*, Tv: *T. vivax,* Tz: T*. b. brucei, T. b. gambiense, T. b. rhodensiense, T. evansi*,

**Supplementary Table 3**: Prevalence of Trypanosome single and multiple infection in tsetse samples analyzed per tsetse species and per country

| **Species** | **Country** | **Prevalence of trypanaosome species/subspecies and mixed infection (%)*** | | | | | | | | | | |
| --- | --- | --- | --- | --- | --- | --- | --- | --- | --- | --- | --- | --- |
|  |  | **Tc** | **Tv** | **Tz** | **Tsg** | **TcTv** | **TcTz** | **TcTsg** | **TvTz** | **TvTsg** | **TzTsg** | **TcTvTz** |
| *G. austeni* | Mozambique | 6.00 | 0.00 | 0.00 | 4.00 | 0.00 | 0.00 | 0.00 | 0.00 | 0.00 | 0.00 | 0.00 |
|  | South Africa | 6.19 | 0.44 | 3.54 | 10.18 | 0.44 | 0.00 | 0.88 | 0.00 | 0.00 | 0.00 | 0.00 |
|  | Eswatini | 0.00 | 0.00 | 0.00 | 0.00 | 0.00 | 0.00 | 0.00 | 0.00 | 0.00 | 0.00 | 0.00 |
|  | Tanzania | 0.00 | 5.00 | 5.00 | 0.00 | 0.00 | 0.00 | 0.00 | 0.00 | 0.00 | 0.00 | 0.00 |
| *G. brevipalpis* | Mozambique | 0.00 | 2.00 | 0.00 ^a^ | 2.00 | 0.00 | 0.00 | 0.00 | 0.00 | 2.00 | 0.00 | 0.00 |
|  | South Africa | 2.33 | 0.67 | 2.67 ^b^ | 4.67 | 0.00 | 0.00 | 0.00 | 0.00 | 0.00 | 0.00 | 0.00 |
| *G. f. fuscipes* | Kenya | 2.25 | 6.74 | 0.00 ^a^ | **11.24** | 2.25 | 1.12 | 0.00 ^a^ | 0.00 | 0.00 | 0.00 | 0.00 |
|  | Uganda | 3.19 | 2.13 | 1.06 ^b^ | 3.19 | 0.00 | 0.00 | 1.06 ^b^ | 0.00 | 0.00 | 0.00 | 0.00 |
| *G. medicorum* | Burkina Faso | 9.09 | **11.69** | **13.64** ^a^ | 0.00 | 1.30 | 1.30 | 0.00 ^a^ | 0.65 | 0.00 | 0.00 | 1.30 |
| *G. m. morsitans* | Kenya | 2.35 | 0.00 | 0.00 | 0.00 | 0.00 | 0.00 | 0.00 | 0.00 | 0.00 ^a^ | 0.00 | 0.00 |
|  | Tanzania | **18.52** | 1.23 | 1.23 | **18.52** | 0.00 | 0.00 | **6.17** | 0.00 | **6.17** ^b^ | 1.23 ^b^ | 0.00 |
|  | Zambia | **12.5** | 0.00 | 1.56 | **21.88** | 0.00 | 0.00 | **12.5** | 0.00 | 0.00 ^a^ | 0.00 ^a^ | 0.00 |
|  | Zimbabwe | 7.91 | 0.72 | 1.44 | **39.57** | 0.00 | 0.00 | 4.32 | 0.00 | 0.00 ^a^ | 0.00 ^a^ | 0.00 |
| *G.m. submorsitans* | Burkina Faso | 5.25 | **11.37** | 0.58 | 0.00 | 0.00 | 0.87 | 0.00 | 0.00 | 0.00 | 0.00 | 0.00 |
| *G. pallidipes* | Ethiopia | 6.10 | 0.65 ^a^ | 1.09 | **10.46** | 0.00 | 0.00 | 0.65 | 0.00 ^a^ | 0.44 | 0.65 | 0.00 |
|  | Kenya | **15.23** | 6.12 ^ab^ | 0.72 | **17.99** | 2.28 | 0.48 | 7.31 | 0.24 ^a^ | 0.12 | 0.48 | 0.00 |
|  | Tanzania | **15.21** | 1.84 ^a^ | 0.46 | **10.14** | 0.00 | 0.46 | 7.37 | 0.46 ^ab^ | 0.46 | 0.92 | 0.00 |
|  | Uganda | 2.59 | 0.00 ^ac^ | 0.86 | 4.31 | 0.00 | 0.00 | 0.00 | 0.00 ^ac^ | 0.00 | 0.00 | 0.00 |
|  | Zambia | 1.37 | 0.68 ^a^ | 0.68 | **34.93** | 0.00 | 0.00 | 7.53 | 0.00 ^a^ | 0.00 | 0.00 | 0.00 |
|  | Zimbabwe | 5.56 | 0.00 ^ac^ | 2.78 | **40.28** | 0.00 | 0.00 | 4.17 | 0.00 ^ab^ | 0.00 | 0.00 | 0.00 |
| *G.p. palpalis* | Democratic R. of Congo | 0.00 | 2.86 | 0.00 | 0.00 | 0.00 | 0.00 | 0.00 | 0.00 | 0.00 | 0.00 | 0.00 |

**Supplementary Table 3 cont.**: Prevalence of Trypanosome single and multiple infection in tsetse samples analyzed per tsetse species and per country

| **Species** | **Country** | **Prevalence of trypanaosome species/subspecies and mixed infection (%)*** | | | | | | | | | | |
| --- | --- | --- | --- | --- | --- | --- | --- | --- | --- | --- | --- | --- |
|  |  | **Tc** | **Tv** | **Tz** | **Tsg** | **TcTv** | **TcTz** | **TcTsg** | **TvTz** | **TvTsg** | **TzTsg** | **TcTvTz** |
| *G. p. gambiensis* | Burkina Faso | 0.64 | 12.09 ^a^ | 3.50 | 3.39 ^a^ | 0.00 | 0.21 | 0.11 | 1.48 | 2.86 ^a^ | 0.64 | 0.00 |
|  | Guinea | 0.00 | 2.23 ^b^ | 0.00 | 0.00 ^b^ | 0.00 | 0.00 | 0.00 | 0.00 | 0.00 ^b^ | 0.00 | 0.00 |
|  | Mali | 0.27 | 5.49 ^ab^ | 1.10 | 0.00 ^b^ | 0.00 | 0.00 | 0.00 | 0.00 | 0.00 ^b^ | 0.00 | 0.00 |
|  | Senegal | 0.55 | 8.96 ^a^ | 1.28 | 1.83 ^b^ | 0.00 | 0.00 | 0.00 | 1.65 | 0.00 ^b^ | 0.00 | 0.00 |
| *G. tachinoides* | Burkina Faso | 2.40 ^a^ | **12.59** | 0.60 | 0.00 | 0.48 ^a^ | 0.60 | 0.00 | 0.12 | 0.00 | 0.00 | 0.00 |
|  | Ghana | 1.71 ^b^ | **14.10** | **19.66** | 0.00 | 0.00 ^b^ | **10.68** | 0.00 | **12.39** | 0.00 | 0.00 | 1.71 |
| **Total (Average%)** |  | **4.78** | **6.75** | **2.29** | **7.06** | **0.41** | **0.63** | **1.71** | **0.83** | **0.54** | **0.23** | **0.09** |

* Tc: *T. congolense* savannah, *T. congolense* Kilifi; *T. congolense* forest, Tsg: *T. simiae*; *T. simiae* Tsavo; *T. godfreyi*, Tv: *T. vivax,* Tz: T*. b. brucei, T. b. gambiense, T. b. rhodensiense, T. evansi*,

**Supplementary Table 5.** List of Primers used for PCR and quantitative PCR (qPCR) analyses of microbiome in *Glossina* species

| **Target Gene** | **Primer Name** | **Primer Sequence (Listed 5’- to -3’)** | **Annealing Temperature (^°^C)** | **Amplicon Size (bp)** | **References** |
| --- | --- | --- | --- | --- | --- |
| fliC (flagellin) (Sodalis) | sod-FliCF | GCA GTT TCA GGA TAC CC | 52.5 | 508 | ^1^ |
|  | sod-FliCR | GGC GGA AAA TGG TAT AG |  |  |  |
| GpCAG133 | GpCAG133-F | ATT TTT GCG TCA ACG TGA | 52.5 | 185-205 | ^2^ |
|  | GpCAG133-R | ATG AGG ATG TTG TCC AGT TT |  |  |  |
| fliC (flagellin) (Sodalis) | sodqPCR-FliCF | GAA GCC ACC GAT CCT GTA AC | 55 | 508 | ^3^ |
|  | sodqPCR-FliCR | CAT CTT TGC CCG TAG AAA TCA C |  |  |  |
| ITS 1 | ITS 1-CF | CCG GAA GTT CAC CGA TAT TG | 60 | 250-710 | ^4^ |
|  | ITS 1-BR | TTG CTG CGT TCT TCA ACG AA |  |  |  |
| 18S rDNA Trypanosomatidae | 18S_Typ_F | CGCCAAGCTAATACATGAACCAA | 60 | 106 | ^5^ |
|  | 18S_Tryp_R | TAATTTCATTCATTCGCTGGACG |  |  |  |

**Reference list**

1. Toh, H. *et al.* Massive genome erosion and functional adaptations provide insights into the symbiotic lifestyle of Sodalis glossinidius in the tsetse host. *Genome Res.* **16**, 149–156 (2006).

2. Baker, M. D. & Krafsur, E. S. Identification and properties of microsatellite markers in tsetse flies *Glossina morsitans* sensu lato (Diptera: Glossinidae). *Mol. Ecol. Notes* **1**, 234–236 (2001).

3. Weiss, B. L., Maltz, M. & Aksoy, S. Obligate symbionts activate immune system development in the tsetse fly. *J Immunol* **188**, 3395–3403 (2012).

4. Njiru, Z. K. *et al.* The use of ITS1 rDNA PCR in detecting pathogenic African trypanosomes. *Parasitol. Res.* **95**, 186–192 (2005).

5. Deborggraeve, S. *et al.* Molecular dipstick test for diagnosis of sleeping sickness. *J. Clin. Microbiol.* **44**, 2884–2889 (2006).
